# Promiscuity in Molecular Mimics of the Cysteine Dioxygenase: Effects of Selenium in the Substrate and Cobalt as the Central Metal Ion

**DOI:** 10.1002/anie.202507578

**Published:** 2025-08-19

**Authors:** Kilian Weißer, Edgar T. K. Weber, Gunasekaran Velmurugan, Beatrice Cula, Konstantin B. Krause, Siad Wolff, M. Qadri. E. Mubarak, Peter Comba, Sam P. de Visser, Christian Limberg

**Affiliations:** ^1^ Institut für Chemie Humboldt‐Universität zu Berlin Brook‐Taylor Straße 2 12489 Berlin Germany; ^2^ Institut für Anorganische Chemie and Interdisciplinary Center for Scientific Computing (IWR) Universität Heidelberg Im Neuenheimer Feld 270 69120 Heidelberg Germany; ^3^ Manchester Institute of Biotechnology The University of Manchester 131 Princess Street Manchester M1 7DN UK; ^4^ Department of Chemical Engineering The University of Manchester Oxford Road Manchester M13 9PL UK; ^5^ Program Teknologi Kimia Industri Universiti Sains Islam Malaysia Bandar Baru Nilai 71800 Malaysia

**Keywords:** Cysteine dioxygenase, Dioxygenase model, Nonheme iron enzymes, O_2_ activation, Selenium

## Abstract

Cysteine dioxygenase (CDO) catalyzes the conversion of cysteine with dioxygen to yield cysteine sulfinic acid, which lies at the branching point of cysteine catabolism. Despite many years of research there are still many questions related to its functioning. Thus, CDO is inactive with selenocysteine (Sec) or when the central iron ion is replaced by cobalt. In this context, we report here biomimetic CDO models with bound selenocysteamine substrate ligands, namely [Tp^Mes^Fe(Se‐CH_2_‐CH_2_‐NH_2_)] and [Tp^Mes*^Fe(Se‐CH_2_‐CH_2_‐NH_2_)] (with Tp^Mes^ = hydrotris(3‐mesitylpyrazol‐1‐yl)borate, Tp^Mes*^ = hydrobis((3‐mesitylpyrazol‐1‐yl)(5‐mesitylpyrazol‐1‐yl)borate) and in addition a cobalt‐analogue [Tp^Mes^Co(Se‐CH_2_‐CH_2_‐NH_2_)]. Upon treatment of the Fe/Se homologues with O_2_ – as in case of the parent cysteamine‐bound complexes – the dioxygenation of the chalcogen atoms was observed. This suggests that the lack in reactivity of CDO‐Sec toward O_2_ does not originate in the electronic situation but in the surrounding protein matrix. Subsequent DFT calculations indeed showed lower initial barriers for selenocysteamine than for cysteamine in support of the experimental work. The complex [Tp^Mes^Co(Se‐CH_2_‐CH_2_‐NH_2_)], where Fe is formally replaced by Co, also reacts with dioxygen – more slowly but selectively – to give [Tp^Mes^Co(O_2_Se‐CH_2_‐CH_2_‐NH_2_)]. Hence, this is an experimental observation of a dioxygenation with O_2_ mediated by a cobalt center in a molecular compound, which is so far without precedence.

## Graphical abstract

A biomimetic model for the cysteine dioxygenase (CDO) was found to be also capable of selectively dioxygenating selenocysteamine, suggesting that the lack of reactivity found for the CDO in combination with selenocysteine originates in the protein matrix. The corresponding cobalt complex mediates the dioxygenation of selenocysteamine, too, and thus represents the first example of a molecular cobalt complex that is potent to activate O_2_ for the dioxygenation of a substrate.

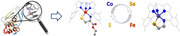

## Introduction

**Scheme 1:**
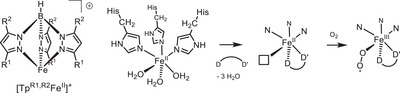
The [Tp^R1,R2^Fe]^+^ moiety in comparison with the binding of iron(II) within 3‐His amino acid environments in oxygenases; for the latter also the substrate binding and subsequent superoxide formation is shown.

**Scheme 2:**
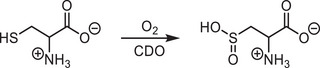
Conversion of cysteine to cysteine sulfinic acid via O_2_ oxidation catalyzed by the CDO.

Mononuclear non‐heme iron enzymes activate dioxygen for the subsequent oxygenation of substrates and are a fascinating class of metalloproteins.^[^ ^1^, ^2^, ^3^, ^4^, ^5^, ^6^, ^7^, ^8^, ^9^, ^10^, ^11^ ^]^ The majority of the representatives contains a deceptively simple active site with a single iron(II) ion anchored by three protein‐based ligands: typically, two imidazole donors of His residues and one carboxylate function (the so‐called 2‐His/1‐carboxylate facial triad)^[^ ^12^, ^13^ ^]^ or three histidine‐based imidazole units (3‐His)^[^ ^14^ ^]^ In consequence, three additional facial sites remain for the binding of exogenous ligands, for instance, a bidentate substrate and O_2_ as shown for the (3‐His) case in Scheme 1. One method to understand the effects of the coordination environment in enzymes on structure and reactivity is the creation of biomimetic models that replicate various features of the active sites but lack the protein environment.^[^ ^15^, ^16^, ^17^, ^18^ ^]^ A representative of the (His)_3_Fe‐enzyme family that in the past has motivated our modelling studies is the cysteine dioxygenase (CDO).^[^ ^19^, ^20^ ^]^ CDO catalyzes the reaction between cysteine (Cys) and dioxygen to yield cysteine sulfinic acid (Scheme 2), which is an intermediate along the cysteine catabolism process in nature.

The enzymatic system has been well studied and crystal structures have been reported of the resting state and substrate bound forms, which were further characterized by spectroscopic means.^[^ ^19^, ^20^, ^21^, ^22^, ^23^, ^24^ ^]^ The aminoethanethiol dioxygenase (ADO) is a (also structurally) related enzyme that dioxygenates the substrate cysteamine (CysAm).^[^ ^25^, ^26^ ^]^ However, recent reports indicate that the more important function may be the dioxygenation of the N‐terminal Cys of proteins.^[^ ^27^ ^]^ While the CDO works exclusively with iron – as a matter of fact the addition of cobalt inhibits its function – it has been recently shown that when iron(II) in the ADO is replaced by cobalt(II) or nickel(II) a small fraction of the activity is retained (rates decrease in the series Fe >>> Co > Ni).^[^ ^28^ ^]^ The CDO does not convert selenocysteine,^[^ ^29^ ^]^ while the activity of the ADO toward selenocysteamine has not been reported yet.

**Scheme 3:**
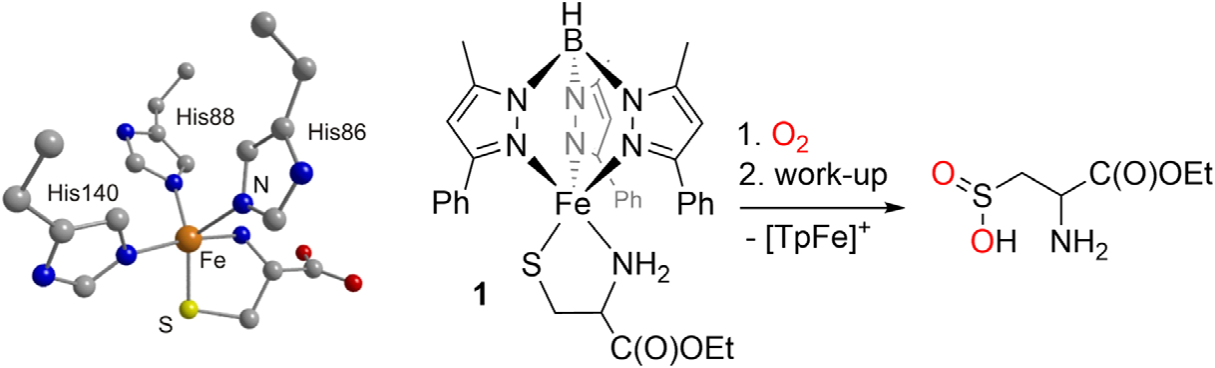
A Tp‐based functional model (right) for the CDO‐substrate complex, the structure of which is depicted for comparison on the left as taken from the 2IC1 pdb file.

To learn what key features (structural, electronic) influence and control the reactivity, several groups, including us, have pursued the design of synthetic model complexes that mimic the active sites of CDO/ADO enzymes.^[^ ^30^, ^31^, ^32^, ^33^, ^34^, ^35^, ^36^, ^37^, ^38^, ^39^, ^40^, ^41^, ^42^, ^43^, ^44^ ^]^ This is a challenging feat, since iron oxidation instead of S‐oxygenation as well as overoxidation at the S atom has to be avoided. For the CDO enzyme it has been shown^[^ ^19^, ^45^ ^]^ that in the first step of the conversion the deprotonated cysteine binds as a bidentate ligand at the (His)_3_Fe site, leaving one coordination site open for the potential binding of O_2_ as shown for the general case in Scheme 1. The active site structure of the CDO‐cysteine substrate complex (CDO‐Cys) is shown in Scheme 3 alongside a corresponding structural and functional model (**1**) that has been developed in our laboratory, using the Tp ligand (Tp = tris(pyrazolyl)borate, see Scheme 1) to simulate the (His)_3_ coordination sphere and a cysteine ester as the substrate ligand.^[^ ^31^ ^]^ While **1** contained phenyl residues at the pyrazolyl donors, in subsequent work we have also tested mesityl residues for our next generation of models.^[^ ^34^ ^]^ This seemingly minor change to the ligand framework proved to bring about major advantages. Unlike in the case of the complex with phenyl substituents, the isolation and crystallization of the sulfinate product complex [Tp^Mes^Fe(O_2_S‐CH_2_‐CH(CO_2_Et)‐NH_2_)], **2**, was successful. This has led to the first structural characterization of a sulfinate product with a *κ* ^2^‐*O*,*O′*‐binding mode of the sulfinate group in the solid state^[^ ^34^ ^]^ that has also been suggested for the enzymatic product complex.^[^ ^46^ ^]^ At the same time in comparison with **1** the reaction rate was increased due to a more spacious reaction pocket. Having found that the [Tp^Mes^Fe]^+^ entity appropriately simulates the active site of the CDO we have now employed it to examine further interesting features researchers have found investigating the CDO, namely, the lack of reactivity toward selenocysteine and the inhibiting effect of cobalt(II),^[^ ^47^, ^48^, ^49^, ^50^ ^]^ to see how this reflects in the model chemistry. We show here that selenocysteamine is rapidly dioxygenated when bound to the [Tp^Mes^Fe]^+^ moiety with much faster rates than cysteamine. Remarkably, the Tp^Mes^ cobalt(II) complex activated O_2_ for the dioxygenation of selenocysteamine as well, albeit much more slowly than the corresponding iron complex.

## Results and Discussion

In work by Brunold and coworkers, it was reported that, like cysteine, also selenocysteine (Sec) can bind to the iron center of the CDO but that it cannot be oxygenated subsequently, as according to calculations the initial activation of O_2_ at the iron center is endothermic.^[^ ^47^ ^]^ We were interested to test this with our model system both with the symmetric scorpionate ligand [Tp^Mes^] (Tp^Mes^ = hydrotris(3‐mesitylpyrazol‐1‐yl)borate) and with a more open asymmetric isomer [Tp^Mes*^] (Tp^Mes*^ = hydrobis((3‐mesitylpyrazol‐1‐yl)(5‐mesitylpyrazol‐1‐yl), that had been already applied in some of our previous studies.^[^ ^34^ ^]^

### Synthesis and Characterization of Tp^Mes^/Tp^Mes^*‐Iron(II)‐Selenocysteaminate Complexes

As an analogue of selenocysteine, we envisaged using selenocysteamine (SeCysAm), which is reasonable based on the results of our previous work: models feature a reduced complexity so that the bare reactivity of the metal center surrounded by its primary coordination sphere can be investigated. Cysteine and cysteamine vary from each other by the carboxylate group and while this can be an important difference for enzymes and their tailored binding pockets, our studies have shown that the carboxylate residue has hardly any influence on the reaction rates in model chemistry that lacks the second coordination sphere of enzymes.^[^ ^34^ ^]^

**Scheme 4:**
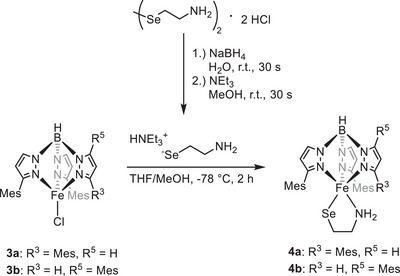
Synthesis of the complexes [Tp^Mes^FeSeCysAm] (**4a**) and [Tp^Mes*^FeSeCysAm] (**4b**).

The same can be assumed for selenocysteine and selenocysteamine and insofar selenocysteamine should represent an adequate surrogate for selenocysteine. However, we first of all had to develop a method for the generation of the free aminoethaneselenolate (selenocysteaminate) by reduction of the corresponding diselenide with NaBH_4_ in water. The reduction in non‐protic solvents was not successful due to the rapid formation of elemental selenium. Even when performing the reaction in water the subsequent work‐up had to be quick, due to the instability of the free selenol. The deprotonation was carried out with NEt_3_ and the addition of the selenolate to a cooled solution of [Tp^Mes^FeCl], **3a**, and [Tp^Mes*^FeCl], **3b**,^[^ ^34^ ^]^ respectively, led to the formation of the target complexes [Tp^Mes^FeSeCysAm], **4a**, and [Tp^Mes*^FeSeCysAm], **4b** (Scheme 4, yields: 61% and 56%, respectively). The employment of selenocysteamine in CDO/ADO model chemistry is so far unprecedented, likely also due to the synthetic challenges encountered as described above.

**Figure 1:**
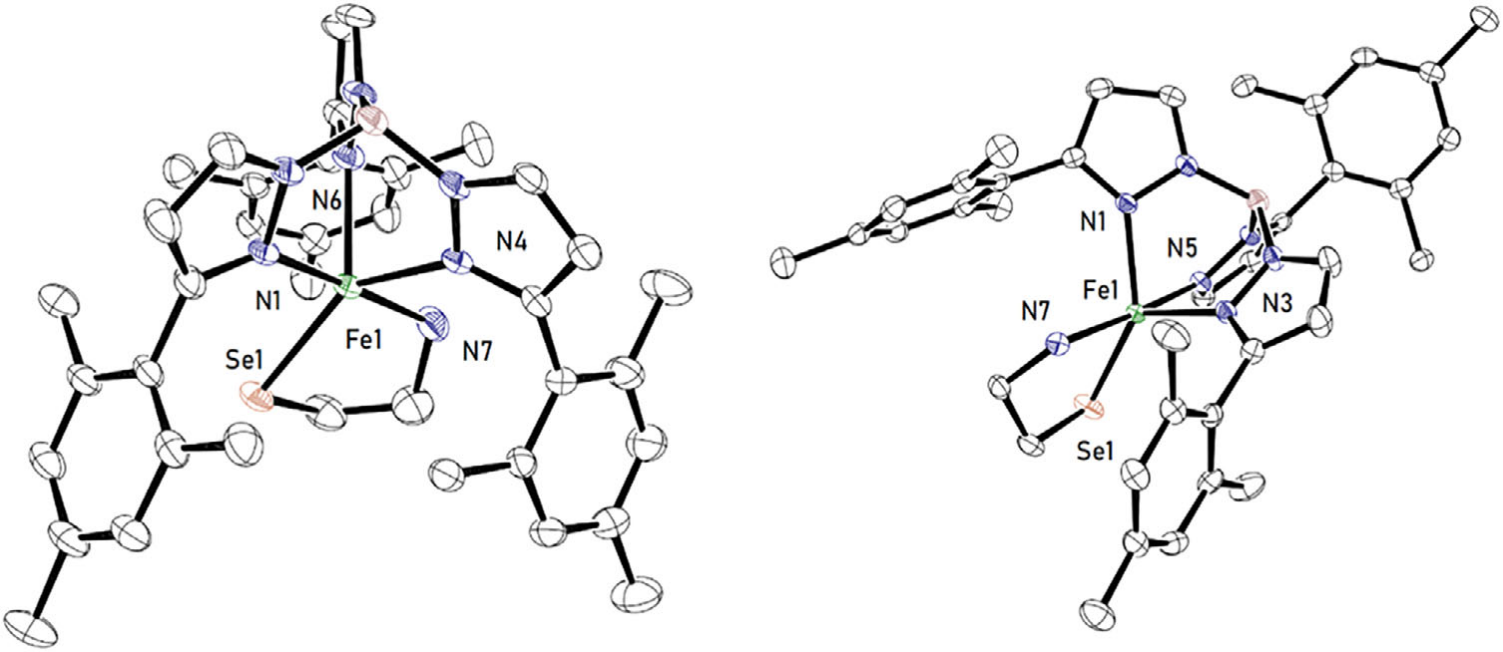
Molecular structure of **4a** (left) and **4b** (right). Hydrogen atoms and solvent molecules have been omitted for clarity. Selected bond lengths in [Å] for **4a** Fe1‐Se1 = 2.4366(6), Fe1‐N7 = 2.250(3) and **4b**: Fe1‐Se1 = 2.4510(5), Fe1‐N7 = 2.213(1).

**Figure 2:**
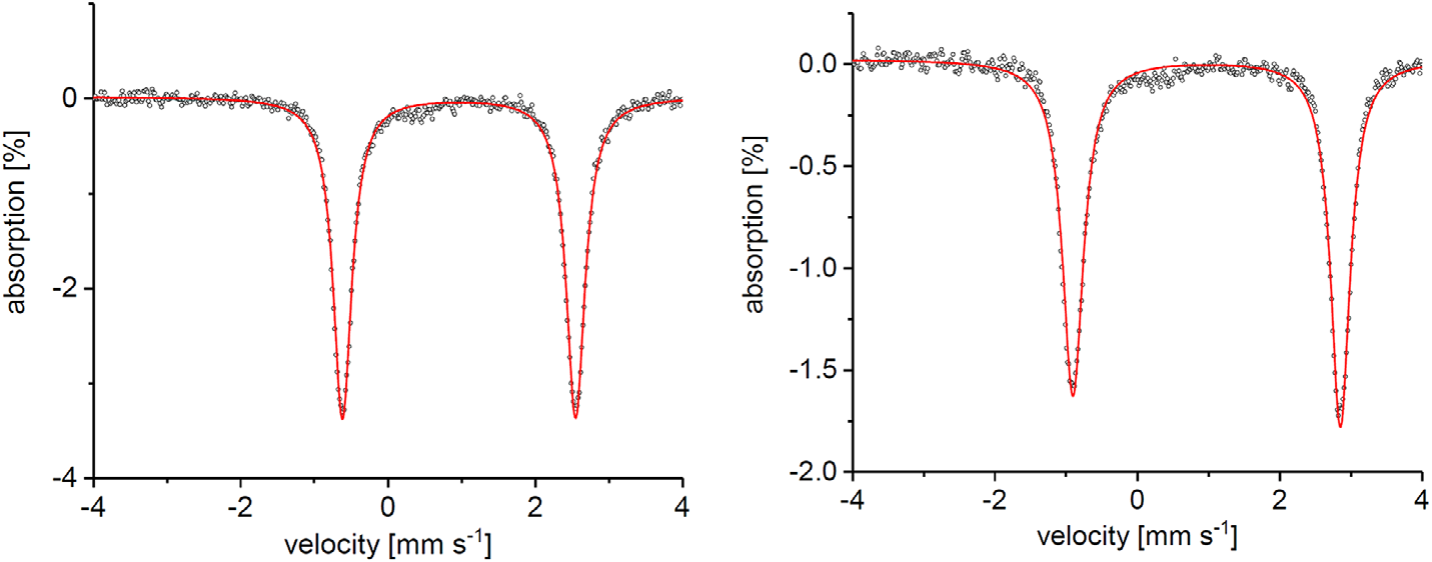
Mössbauer spectra of [Tp^Mes^FeSeCysAm] **4a** (left), with δ = 1.00 mm s^−1^ and Δ*E*_Q_ = 3.19 mm s^−1^, and [Tp^Mes*^FeSeCysAm] **4b** (right) with δ = 0.97 mm s^−1^ and Δ*E*_Q_ = 3.76 mm s^−1^.

Single crystals suitable for X‐ray diffraction could be grown by slow solvent evaporation from concentrated solutions in benzene, and the results of X‐ray diffraction analyses are shown in Figure 1. As observed for the CDO, the three nitrogen atoms are coordinated facially and the substrate binds to the metal center in a chelating fashion. The resulting coordination spheres of the iron(II) atoms in compounds **4a** and **4b** are in‐between square pyramidal and trigonal bipyramidal. The τ‐values (τ_4a_ = 0.52 and τ_4b_ = 0.53) are in good agreement with the values reported for the CDO‐Cys found in nature, which range between τ = 0.46^[^ ^23^ ^]^ and 0.71.^[^ ^19^ ^]^ Not unexpectedly, the Fe‐Se bonds of complexes **4a** and **4b** are slightly elongated (2.44 and 2.45 Å) compared to the Fe‐S bond found in the corresponding sulfur‐analogue [Tp^Mes^FeCysAm] (2.29 Å).^[^ ^34^ ^]^ A comparison of the Mössbauer spectrum of **4a** (see Figure 2) with the one of [Tp^Mes^FeCysAm] shows a large difference in the quadrupole splittings of ΔΔ*E*_Q_ = +0.41 mm s^−1^ (compare Table 1, entry 1 vs. 2) upon replacement of sulfur by selenium, despite a very similar electronegativity of selenium compared to sulfur (χ_M_(Se) = 2.43 $\sqrt{eV}$ and χ_M_(S) = 2.49 $\sqrt{eV}$).^[^ ^51^ ^]^ The difference is thus likely due to the larger covalent radius of selenium with respect to sulfur. This is further supported by the Wiberg bond index (0.686 and 0.688 for selenocysteamine and 0.642 and 0.651 for cysteamine) and a quantum theory of atoms in molecule (QTAIM) analysis.^[^ ^52^ ^]^ The occupancy and the natural atomic hybrid orbital composition of the calculated Fe ─ Se and Fe ─ S bonds confirm the difference in the bond orbitals (see Table S15). The QTAIM‐defined topological properties at the Fe ─ Se bond critical point (BCP) indicate a significantly more covalent character compared to Fe ─ S, and the contour plot Laplacian function $\nabla_{\rho_{\left(r\right)}}^{2}$ through the Fe–Se/S planes shows that the valence shell charge concentration zone (VSCC) of the Se atom is more diffused toward the Fe atom (see Table S16 and Figure S54). The isomer shift, in contrast, has basically the same value for both compounds (δ_Se_ = 1.00 mm s^−1^ and δ_S_ = 0.99 mm s^−1^), confirming that the complexes are in the same oxidation and spin states (S = 2). The same phenomenon was observed for the asymmetric systems [Tp^Mes*^FeCysAm] and [Tp^Mes*^FeSeCysAm] **4b**, for which an even larger difference in the quadrupole splittings (ΔΔ*E*_Q_ = +0.50 mm s^−1^) was found (entry 3 and 4 in Table 1). Similarly to the symmetric systems, the isomer shifts observed for those compounds are almost identical (δ_Se_ = 0.97 mm s^−1^ and δ_S_ = 0.96 mm s^−1^). The computed Mössbauer spectral parameters agree well with the experimental ones (see Table 1).^[^ ^53^ ^]^

**Table 1:** Comparison of the isomer shifts δ and quadrupole splittings Δ*E*_Q_ for the TpFe‐Se‐, and ‐S‐bound systems, the CDO‐Cys found in *Rattus norvegicus* as well as the ADO‐CysAm.

	Exp.		DFT	
	δ (mm s^−1^)	Δ*E*_Q_ (mm s^−1^)	δ (mm s^−1^)	Δ*E*_Q_ (mm s^−1^)
[Tp^Mes^FeSeCysAm]^a)^	1.00	3.19	0.98	3.12
[Tp^Mes^FeCysAm]^b)^	0.99	2.78	0.97	2.71
[Tp^Mes*^FeSeCysAm]^a)^	0.97	3.76	0.99	3.71
[Tp^Mes*^FeCysAm]^b)^	0.96	3.26	0.88	3.16
[Tp^Mes^FeO_2_SeCysAm]^a)^	1.19	3.52	1.15	3.48
[Tp^Mes^FeO_2_CysAm]^b)^	1.18	3.43	1.14	3.40
[Tp^Mes*^FeO_2_SeCysAm]^a)^	1.19	3.51	1.15	3.49
[Tp^Mes*^FeO_2_CysAm]^b)^	1.17	3.47	1.16	3.43
CDO‐Cys^c)^	0.80	2.80		
ADO‐CysAm^d)^	1.19	2.85		

^a)^ This work, measured as an amorphous solid at 14 K.

^b)^ Measured as a solid at 13 K.^[^ ^34^ ^]^

^c)^ ^57^Fe‐enriched protein, measured as isolated without any information on the temperature.^[^ ^54^ ^]^

^d)^ ^57^Fe‐enriched protein, measured as isolated at 4.2 K.^[^ ^55^ ^]^

### Reactivity of the Iron(II) Selenocysteaminate Complexes Toward Dioxygen – Selective Dioxygenation

**Scheme 5:**
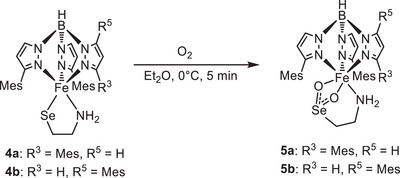
Synthesis of the seleninate complexes [Tp^Mes^FeO_2_SeCysAm] **5a** and [Tp^Mes*^FeO_2_SeCysAm] **5b**.

**Figure 3:**
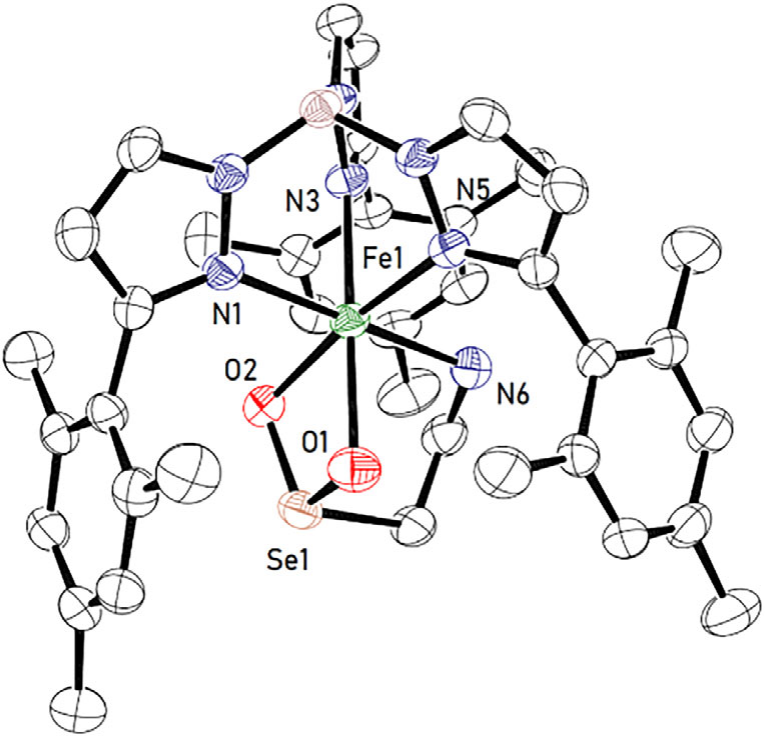
Crystal structure of **5a** (hydrogen atoms and solvent molecules have been omitted for clarity). Selected bond lengths in [Å]: **5a**: Fe1─O1 = 2.167(3), Fe1─O2 = 2.177(3), Fe1─N6 = 2.257(4), Se1 ─ O1 = 1.691(5), Se1 ─ O2 = 1.685(4).

**Figure 4:**
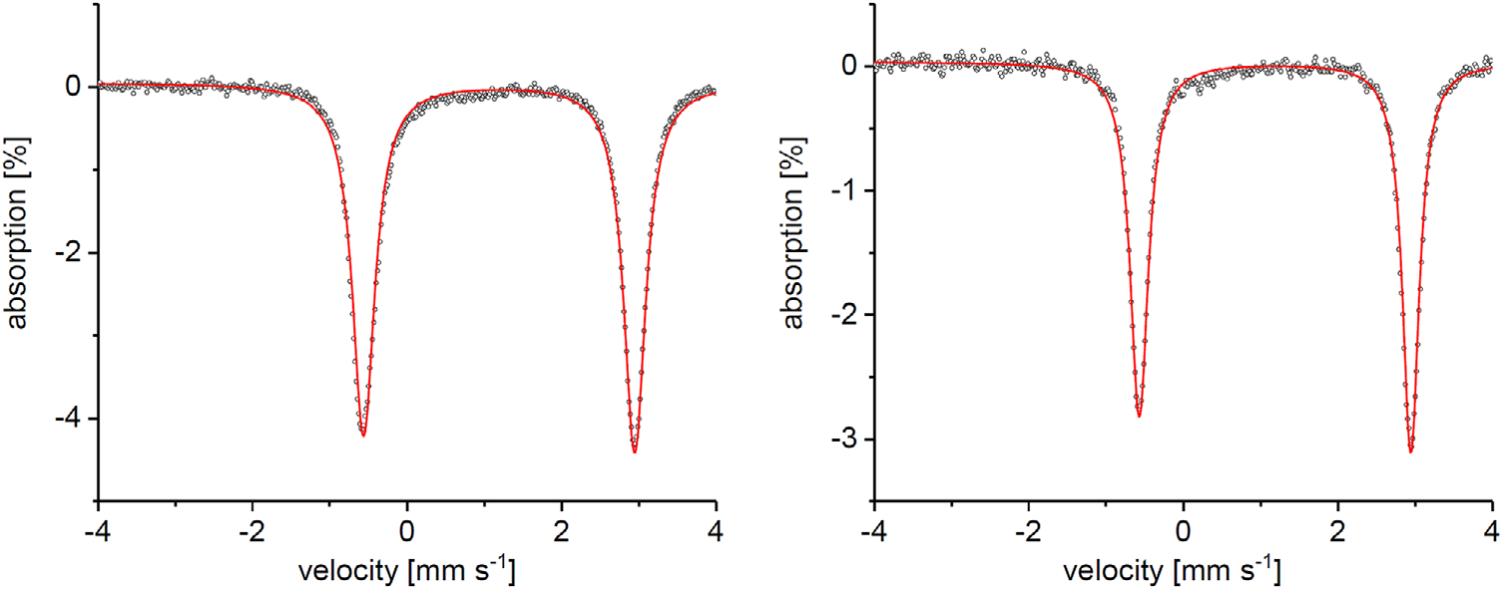
Mössbauer spectra of [Tp^Mes^FeO_2_SeCysAm] **5a** (left) with δ = 1.19 mm s^−1^ and Δ*E*_Q_ = 3.52 mm s^−1^ and [Tp^Mes*^FeO_2_SeCysAm] **5b** with δ = 1.19 mm s^−1^ and Δ*E*_Q_ = 3.51 mm s^−1^ (right).

Bearing the experimental and theoretical findings reported for CDO‐Sec as outlined above in mind, we decided to study the O_2_‐reactivity of our models **4a** and **4b**. Remarkably, unlike CDO‐Sec, both complexes [Tp^Mes^FeSeCysAm] **4a** and [Tp^Mes*^FeSeCysAm] **4b** dissolved in Et_2_O react with dioxygen readily and selectively (single product, no overoxidation), even at −80 °C, resulting in the formation of the corresponding complexes [Tp^Mes^FeO_2_SeCysAm] **5a**, and [Tp^Mes*^FeO_2_SeCysAm] **5b** (see Scheme 5). This has been shown spectroscopically, by elemental analysis and also by an X‐ray‐diffraction analysis of single crystals grown by slow solvent evaporation from a saturated solution of **5a** in chlorobenzene and DCM (see Figure 3). The result shows the dioxygenated aminoethaneselenolate being bound as a chelating ligand to the iron center, which after the reaction is again present in the oxidation state of + 2 (confirmed by Mössbauer spectroscopy, Figure 4), as expected for a dioxygenase reactivity.

The iron(II) ions are thus coordinated pseudo‐octahedrally by three nitrogen atoms of the Tp ligand, one amino function of the substrate and the seleninate function in a *κ* ^2^‐*O*,*O*'‐coordination mode. The selenium─oxygen bonds are almost identical in length within **5a** [Se1 − O1: 1.691(5), Se1 − O2: 1.685(4) Å]. Likewise, the iron─oxygen bonds are similar [Fe1‐O1 = 2.167(3), Fe1‐O2 = 2.177(3)]. The oxygen‐selenium‐oxygen angle is with 99.3(2) smaller than the ideal tetrahedral angle of 109.5°, likely due to a small extent of hybridization.

When the complexes **5a** and **5b** are compared with their S‐analogues, it can be seen that they also share some spectroscopic similarities. The isomer shifts in the Mössbauer spectra (Figure 4) are nearly identical, and also the values for the quadrupole splittings are rather similar, as now there is no contact with Se anymore and the Fe‐O distances are comparable (see Table 1). They clearly indicate a high‐spin state also in the product complexes.

We conclude at this point that in contrast to the CDO, which mediates the dioxygenation of Cys but not of Sec, replacement of S by Se in our models does not lead to an inhibition of reactivity. In fact, the selenocysteamine complex reacts more rapidly [experimentally determined half‐life of the reaction: $t_{1/2}^{0^{\circ }C}$(**4a**+O_2_) = 13 s, see SI) than the corresponding cysteamine complex ($t_{1/2}^{25^{\circ }C}$([Tp^Mes^FeCysAm]+O_2_) = 40 s, calculated from $k_{S}^{25^{\circ }C}$([Tp^Mes^FeCysAm]+O_2_) = 1.74 · 10^−2^ s^−1^) in the presence of excess (> 10 eq.) O_2_.^[^ ^34^ ^]^

### The Cobalt(II)/Selenocysteaminate/O_2_ System – an Unprecedented Case of O_2_ Activation at a Cobalt Center for a Subsequent Dioxygenation of a Substrate

We next turned our attention to a further characteristic of the CDO for a comparison with model chemistry. Considering non‐heme iron dioxygenases more generally, it has been found that some of them, like the quercetin dioxygenase or the extradiol catechol dioxygenase,^[^ ^56^ ^]^ are promiscuous, as they do not only function with iron but are also capable of using different metal cofactors for catalysis, including cobalt. By contrast, the CDO functions exclusively with iron, while cobalt is in fact inhibiting the enzymes’ activity by displacing the iron from the active site.^[^ ^48^ ^]^ In biomimetic oxidation chemistry, cobalt is often used as a substitute for iron for various reasons. First, the complexes often remain prone to react with O_2_ and compared to iron(III) superoxides, cobalt(III) superoxides are more stable, thus allowing for a mimicking of the primary step of the reaction sequence. Moreover, non‐heme high‐spin Co(II) systems are spectroscopically more accessible, for example, they have intense absorption bands in the visible region, which facilitates reaction monitoring. Fiedler and coworkers have synthesized a [Tp^Ph2^Co(S‐CH_2_‐CH(CO_2_Et)‐NH_2_)] complex and observed reversible O_2_‐binding as indicated by a new band in the electronic spectra at 483 nm.^[^ ^44^ ^]^ Goldberg and coworkers accessed the complex [Co(Me_3_TACN)(S_2_SiMe_2_)], which formed a stable oxygen adduct featuring bands at 445 and 550 nm.^[^ ^57^ ^]^ None of these complexes, however, showed subsequent dioxygenase reactivity.

**Scheme 6:**
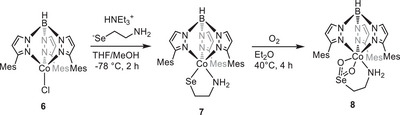
Synthesis of the selenocysteamine complex **7** from [Tp^Mes^CoCl] **6** and the respective seleninate complex [Tp^Mes^CoO_2_SeCysAm] **8**.

We have already previously reported, that – like in case of the enzyme – replacement of the iron(II) central metal ion against cobalt(II) in [Tp^Mes^FeCysAm] **4a** and [Tp^Mes*^FeCysAm] **4b** stops the dioxygenase reactivity, although [Tp^Mes*^CoCysAm] was shown to form a superoxide in contact with O_2_ at low temperatures, featuring a characteristic absorption at 482 nm.^[^ ^34^ ^]^ With the background of this result and the observations made upon employment of selenocysteamine as described above, we then performed an experiment that has not yet been tested for the CDO, namely the exchange of *both* Fe and S in our parent model complex by Co and Se, respectively, and study the effects on the O_2_ reactivity. We have thus prepared the cobalt analogue of complex **4a** [Tp^Mes^CoSeCysAm], **7**, applying similar procedures as for **4a** starting from [Tp^Mes^CoCl], **6** (see Scheme 6); both crystallize in the same space group with comparable bond lengths and angles (see Supporting Information). Remarkably, **7** *does* react with O_2_ – significantly slower than **4a** [$t_{1/2}^{0^{\circ }C}$(**7 **+ O_2_) = 51 min], but rather selectively – to finally yield solely the dioxygenated product [Tp^Mes^CoSeO_2_CysAm], **8**. At room temperature, the reaction was completed after ca. 18 h, but it was possible to accelerate the conversion through warming to 40 °C (Scheme 6). When the reaction was monitored at temperatures between RT and ‐80 °C with time‐resolved UV/Vis spectroscopy, unlike in case of the aforementioned systems, no bands appeared that could have been assigned to a reaction intermediate, such as a superoxide. This is likely due to the fact that the barrier after the superoxide – unlike in other cases – is not sufficiently high to enrich it (vide infra).

**Figure 5:**
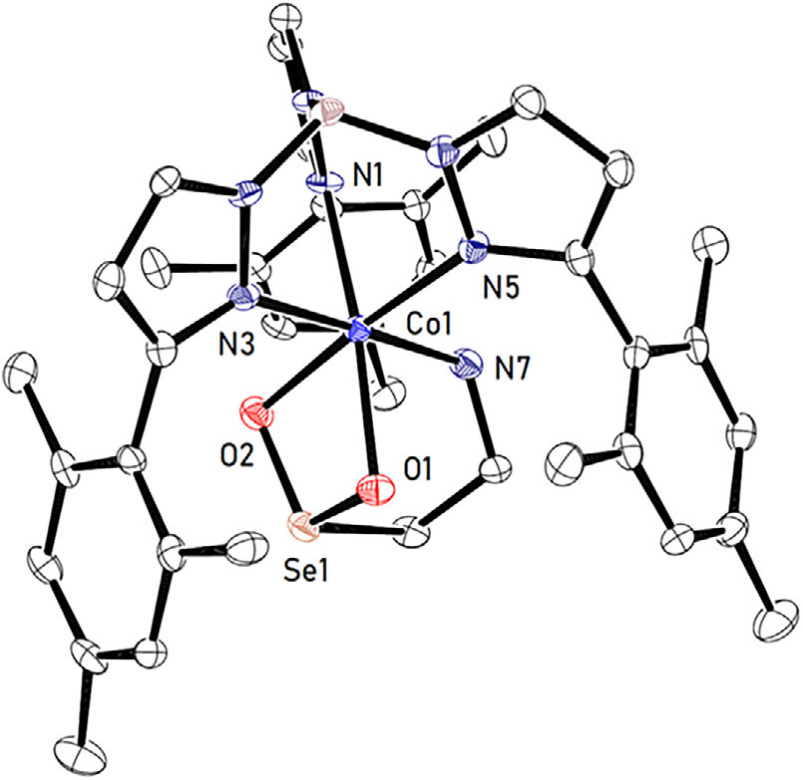
Molecular structure of **8** (hydrogen atoms and solvent molecules have been omitted for clarity). Selected bond lengths in [Å]: Co1─O1 = 2.166(5), Co1─O2 = 2.137(4), Co1─N7 = 2.203(6), Se1 ─ O1 = 1.696(4), Se1 ─ O2 = 1.6898(5).

Slow evaporation of the volatiles from a saturated solution of **8** in chlorobenzene resulted in the formation of single crystals suitable for X‐ray diffraction (see Figure 5). The molecular structure determined for the product complex **8** resembles the one of **5a**, with the seleninate bound to the metal center in a κ^3^−N,O,O‐fashion via the two oxygen atoms and the amine.

A clean four‐electron oxidation of a substrate is a quite rare observation in O_2_ activation processes, in particular for low‐molecular weight complexes. While there are a few instances known for iron complexes,^[^ ^58^ ^]^ cobalt complexes capable of activating O_2_ for the dioxygenation of any substrate are so far unknown.^[^ ^59^ ^]^ Within molecular model chemistry, complex **7** therefore shows a unique behavior toward dioxygen: while a H_2_O_2_‐derived peroxide cobalt complex has been reported to transfer both O atoms to a substrate,^[^ ^60^ ^]^ **7** represents the first case of a cobalt(II) compound that activates O_2_ yielding dioxygenase reactivity. Hence, in contrast to published complexes, the activation barrier after initial dioxygen adduct formation (see above) must be substantially lower, as we will discuss in the following section.

It has been demonstrated for the [Tp^Ph,Me^FeCys] complex **1** through employment of a mixture of ^18^O_2_/^16^O_2_ and subsequent ESI‐MS analysis that both oxygen atoms transferred to the substrate in fact originated from the same dioxygen molecule: no m/z signal had been observed for the mixed ^18^O/^16^O isotopologue.^[^ ^31^ ^]^ In an attempt to substantiate this assertion also for compound **7**, analogous experiments were conducted. However, due to the large number of Co and Se isotopes the signals of protonated [Tp^Mes^Co^16^O_2_SeCysAm] **8** and [Tp^Mes^Co^18^O_2_SeCysAm] **^18^O‐8** are strongly overlapping, so that – although the resulting signal can be fitted to the exclusive presence of only the pure isotopologues – the presence also of a mixed isotopologue cannot be rigorously excluded.

### DFT Analysis of the Relevant Reaction Paths

**Figure 6:**
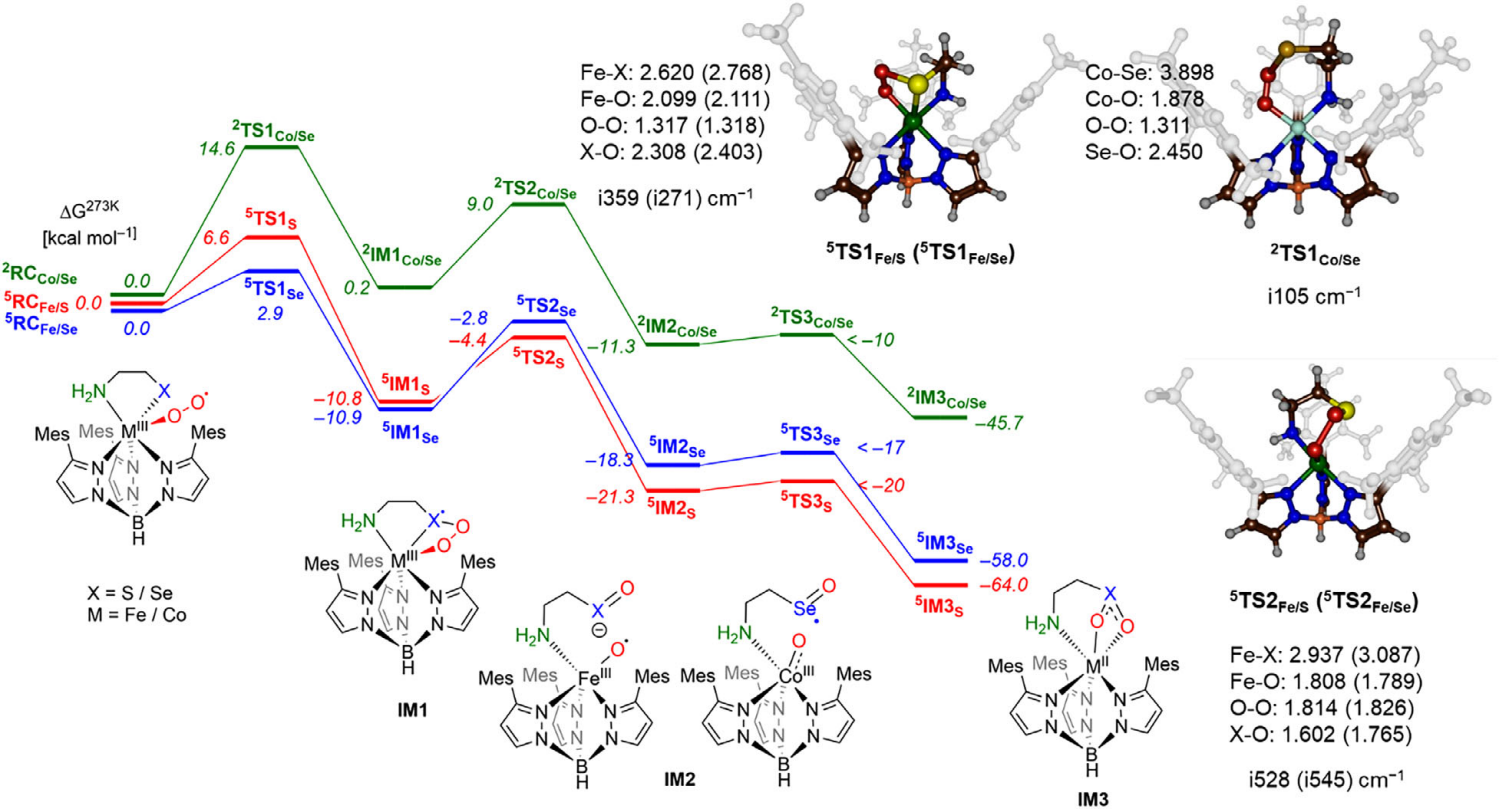
Free energy landscape for cysteamine/selenocysteamine dioxygenation by a biomimetic metal(III)‐superoxide complex (M = Fe/Co). Free energies are in kcal mol^−1^ and contain, zero‐point, solvent, thermal and entropic corrections at 273 K. Optimized transition state geometries give bond lengths in Å and the imaginary frequency in cm^−1^.

Subsequently, we performed DFT calculations on [Tp^Mes^Fe(O_2_)CysAm], [Tp^Mes^Fe(O_2_)SeCysAm] and [Tp^Mes^Co(O_2_)SeCysAm] and studied the intramolecular dioxygen transfer to the substrate. The majority of computational studies concerning the CDO and low‐molecular weight analogues suggest that the conversion occurs along the *S* = 2 potential energy surface (PES),^[^ ^33^, ^46^, ^56^ ^]^ which is the case for **4a**, too, considering that both **4a** and the product complex **5a** have high‐spin configurations and the calculations predict that also the path between them is lowest in energy on the quintet surface, which is thus shown in Figure 6. In case of the Co/Se system, **7** and **8** have high‐spin configurations (S = 3/2), too, as demonstrated experimentally by SQUID measurements (see SI). However, for comparable Co/S complexes, it has been shown (experimentally and theoretically) that upon O_2_ binding (superoxide formation) the S = ½ state becomes significantly more stable.^[^ ^34^, ^44^ ^]^ Indeed, DFT calculations on the [Tp^Mes^CoSeCysAm] and [Tp^Mes^Co(O_2_)SeCysAm] complexes gave different spin state orderings and ground states in line with the Co/S results. We have therefore performed the mechanistic calculations for the doublet surface only.

Figure 6 displays the stepwise reaction mechanism after dioxygen activation to give a superoxide (reacting complex, **RC**). It starts with the attack of the terminal oxygen atom of the metal‐superoxide entity on the S/Se atom of the substrate. This mechanism was previously shown by computational studies to be the preferred mechanism for CDO and MDO enzymes.^[^ ^33^, ^46^, ^61^, ^62^, ^63^, ^64^, ^65^, ^66^, ^67^, ^68^ ^]^ Based on the results of some recent experimental studies concerning the Co‐ADO, an alternative mechanism via attack of the proximal oxygen atom of the metal‐superoxide moiety at the chalcogen atom of the substrate to form a perchalcogenate intermediate has been suggested.^[^ ^28^ ^]^ To test whether proximal attack is a viable mechanism to generate structures **5** and **8**, we ran constraint geometry scans for those pathways, see Figure S53 in the Supporting Information. The calculations never led to a persulfenate/perselenate local minimum but instead the geometry scans show the continuous increase in energy, and the products of the scan were metal‐cysteamineperoxo/selenocysteamineperoxo complexes, with cleaved metal‐oxygen bonds. As such, the persulfenate/perselenate structure is not a catalytic cycle intermediate on the way to complexes **5** and **8**. Similar to previous studies on CDO and CDO model complexes,^[^ ^33^, ^46^, ^61^, ^62^ ^]^ the reaction proceeds via the initial O‐X (X = S/Se) bond formation to form metal(III)‐peroxochalcogenate intermediate **IM1** via transition state **TS1**. In the next step the dioxygen bond cleaves via transition state **TS2** to form the sulfoxide/selenoxide metal oxido intermediate **IM2**. The final oxygen atom transfer via transition state **TS3** then gives the sulfinate and seleninate product complexes either with just one (**IM3**) or both oxygen atoms bound to iron (**IM3’**). The overall free energy profile in Figure 6 shows that each subsequent intermediate is more stable than its precursor for both the iron‐cysteamine and iron‐selenocysteamine conversions, while the first step is thermoneutral for the cobalt‐selenocysteamine system, but exergonic thereafter. Therefore, the reactions are all thermodynamically feasible in the biomimetic model in agreement with experimental observation of products. The fact that the selenocysteine reaction does not proceed in the enzyme, therefore, will relate to the substrate binding and positioning in the active site of the enzyme that is accommodated for cysteine rather than selenocysteine.

Interestingly, the first barrier is small for both Fe systems, namely ^5^ **TS1**_Fe/S_ is *ΔG* = 6.6 kcal mol^−1^ above reactants, while ^5^ **TS1**_Fe/Se_ is at *ΔG* = 2.9 kcal mol^−1^. As such, dioxygen will thus react rapidly with substrate to form the persulfenate/perselenate complex **IM2**. These barriers also suggest that the superoxide will react more rapidly with selenocysteamine than with cysteamine, which aligns with the documented redox potentials of the respective species (*E^0^* (CysAm^−^) = −416 mV (vs. SHE)^[^ ^69^ ^]^ and *E^0^* (SeCysAm^−^) = −513 mV (vs. SHE)^[^ ^70^ ^]^) as well as with the experimental data presented above. To further understand the origin of the variation of the barriers, we analyzed the unpaired spin density distributions of the two complexes. Thus, in ^5^ **RC**_Fe/S_, the ground state has four unpaired electrons resulting in a spin of 4.08 on iron, 0.61 on the substrate and ‐0.74 on the dioxygen group. By contrast, in ^5^ **RC**_Fe/Se_, the dioxygen groups and the iron center have slightly more unpaired spin density of −0.77 and 4.17, respectively. This small change in radical character may enhance the reactivity and trigger lower barriers for ^5^ **TS1**_Fe/Se_ with respect to ^5^ **TS1**_Fe/S_. A further contribution has structural origins: the longer Fe─Se bond positions the Se atom slightly better for superoxide attack and the resulting peroxide has to bend less. As the two **TS2** barriers (^5^ **TS2**_Fe/S_ and ^5^ **TS2**_Fe/Se_) are much less affected by the replacement of sulfur by selenium, the variations in ^5^ **TS1** are responsible for the rate differences. The transition state structures are shown in Figure 6. Both have an imaginary mode for O─X (X = S/Se) formation and a relatively long Fe‐X distance, while the Fe─O and O─O distances are very similar. Replacement of iron by cobalt leads to major structural changes during the reaction mechanism and the selenium atom loses its contact with the cobalt center rapidly during the oxygen activation process. In particular, in ^2^ **TS1**_Co/Se_ the Co─Se distance is significantly lengthened to 4 Å upon O─Se bond formation. This leads to a shortening of the Co‐O bond to 1.878 Å in ^2^ **TS1**_Co/Se_ as compared to the Fe‐O distances of well over 2 Å in the first transition state structures with iron.

In the next reaction step, the dioxygen bond breaks, in case of Fe via ^5^ **TS2**_Fe/S_ or ^5^ **TS2**_Fe/Se_. This leads to O atom transfers to the chalcogenate and iron atoms. Formally, this would lead to Fe^IV ^= O units, but the electronic structure is more consistent with an iron(III)‐oxyl species as often seen in biomimetic model complexes.^[^ ^71^, ^72^, ^73^ ^]^ In case of Co/Se O─O, bond cleavage would lead via ^2^ **TS1**_Co/Se_ to an O atom transfer to the Co^II^ center and, thus, by analogy to the iron case, to a Co^IV ^= O or a cobalt(III)‐oxyl unit. However, the ^2^ **IM2**_Co/Se_ complex has only little spin density on the oxo group, but there is significant spin density on the selenium atom and almost two unpaired electrons on Co. This indicates that a tentative Co^IV ^═ O formed has received an electron form the oxy‐selenolate moiety, causing radical character for the latter and the generation of a Co^III ^═ O entity. The short Co─O bond length calculated matches nicely to a corresponding distance recently found for an isolated Co^III^O complex.^[^ ^74^ ^]^ For cysteamine a similar free energy for ^5^ **TS1**_Fe/S_ and ^5^ **TS2**_Fe/S_ is found (6.6 vs. 6.4 kcal mol^−1^), while for selenocysteamine the second step is somewhat higher in free energy than the first one. By contrast, for the Co/Se system the first barrier, that is, via ^2^ **TS1**_Co/Se_, is rate‐determining. Therefore, the kinetic profiles of the Fe/S, Fe/Se and Co/Se systems are different and dependent on the central metal ion and the chalcogen atom bound to it.

The final oxygen atom transfer to form the product complexes is highly exergonic (by > 50 kcal mol^−1^ for both Fe complexes, and 45.7 kcal mol^−1^ for Co) and has negligible barriers. The optimized geometry of ^5^ **IM3**_Fe/Se_ (Figure S44) matches the experimental crystal structure in Figure 3 well. The calculated Fe─O distances in ^5^ **IM3**_Fe/Se_ are 2.19 and 2.23 Å compared to 2.17 and 2.18 Å in the crystal structure, while the Fe─N distance is 2.27 Å (calculated) versus 2.26 Å (crystal structure). Consequently, the calculations compare well with experimental observation. The fact that ^2^ **TS1**_Co/Se_ lies 11.7 kcal mol^−1^ higher than ^5^ **TS1**_Fe/Se_ explains that the Fe/Se system reacts much faster than the one with Co/Se. At the same time the overall value of 14.6 kcal mol^−1^ rationalizes that the dioxygenation reaction proceeds – in contrast to the one of the abovementioned [Tp^Ph2^Co(S‐CH_2_‐CH(CO_2_Et)‐NH_2_)] complex, for which a barrier higher than 30 kcal mol^−1^ was calculated that prohibits any further reaction after cobalt(III) superoxide formation.^[^ ^44^ ^]^ This also explains that **RC**_Co/Se_ cannot be sufficiently enriched for characterization.

## Conclusion

We have for the first time accessed and introduced selenocysteamine into CDO/ADO model chemistry as a mimic of selenocysteine. While the CDO is not capable of dioxygenating selenocysteine, a corresponding transformation is observed with selenocysteamine as the substrate for Tp^Mes^/Tp^Mes*^Fe^II^‐based models, demonstrating that it is thermodynamically and kinetically feasible. DFT calculations showed that the mechanism by which selenocysteamine is converted is analogous to the one of cysteamine, but the first step after superoxide formation has a lower barrier, so that this reaction step is faster. However, in consequence the highest barrier in the Fe/Se profile after superoxide formation is the O‐O cleavage transition state via **TS2**, whereas the S─O bond formation is associated with the highest barrier in the Fe/S system. Replacing iron(II) by cobalt(II) in the complexes leads to a deceleration of the reaction, which again supports previous proposals that O_2_ activation proceeds at the metal center. Remarkably, the model Co‐analogue was found to react via dioxygenation of selenocysteamine, which is a unique reactivity since O_2_ activation by cobalt complexes published so far failed to show dioxygenation reactivity. However, it is consistent with the recent finding that the cobalt‐substituted ADO dioxygenates cysteamine.^[^ ^28^ ^]^

## Supporting Information

The authors have cited additional references within the Supporting Information.^[^ ^75^, ^76^, ^77^, ^78^, ^79^, ^80^, ^81^, ^82^, ^83^, ^84^, ^85^, ^86^, ^87^, ^88^, ^89^, ^90^, ^91^, ^92^, ^93^ ^]^

## Conflict of Interests

The authors declare no conflict of interest.

## Supporting information



Supporting Information

Supporting Information

## Data Availability

The data that support the findings of this study are available in the Supporting Information of this article.

## References

[1] C. J. Schofield , Z. Zhang , Curr. Opin. Struct. Biol. 1999, 9, 722–731. 10607676 10.1016/s0959-440x(99)00036-6

[2] E. I. Solomon , T. C. Brunold , M. I. Davis , J. N. Kemsley , S. K. Lee , N. Lehnert , F. Neese , A. J. Skulan , Y. S. Yang , J. Zhou , Chem. Rev. 2000, 100, 235–350. 11749238 10.1021/cr9900275

[3] T. D. H. Bugg , Curr. Opin. Chem. Biol. 2001, 5, 550–555. 11578928 10.1016/s1367-5931(00)00236-2

[4] M. Costas , M. P. Mehn , M. P. Jensen , L. Que , Chem. Rev. 2004, 104, 939–986. 14871146 10.1021/cr020628n

[5] M. M. Abu‐Omar , A. Loaiza , N. Hontzeas , Chem. Rev. 2005, 105, 2227–2252. 15941213 10.1021/cr040653o

[6] E. G. Kovaleva , J. D. Lipscomb , Nat. Chem. Biol. 2008, 4, 186–193. 18277980 10.1038/nchembio.71 PMC2720164

[7] S. P. De Visser , D. Kumar in , Iron‐Containing Enzymes: Versatile Catalysts of Hydroxylation Reactions in Nature, RSC Publishing, Cambridge (UK), 2011.

[8] A. R. McDonald , L. Que , Coord. Chem. Rev. 2013, 257, 414–428.

[9] K. Ray , F. F. Pfaff , B. Wang , W. Nam , J. Am. Chem. Soc. 2014, 136, 13942–13958. 25215462 10.1021/ja507807v

[10] M. D. White , E. Flashman , Curr. Opin. Chem. Biol. 2016, 31, 126–135. 27015291 10.1016/j.cbpa.2016.02.017 PMC4879150

[11] C. Q. Herr , R. P. Hausinger , Trends Biochem. Sci. 2018, 43, 517–532. 29709390 10.1016/j.tibs.2018.04.002 PMC6014900

[12] S. Kal , L. Que , J. Biol. Inorg. Chem. 2017, 22, 339–365. 28074299 10.1007/s00775-016-1431-2

[13] P. C. A. Bruijnincx , G. van Koten , R. J. M. Klein Gebbink , Chem. Soc. Rev. 2008, 37, 2716. 19020684 10.1039/b707179p

[14] D. Buongiorno , G. D. Straganz , Coord. Chem. Rev. 2013, 257, 541–563. 24850951 10.1016/j.ccr.2012.04.028 PMC4019311

[15] W. Nam , Y. M. Lee , S. Fukuzumi , Acc. Chem. Res. 2018, 51, 2014–2022. 30179459 10.1021/acs.accounts.8b00299

[16] L. Vicens , G. Olivo , M. Costas , ACS Catal. 2020, 10, 8611–8631.

[17] G. Mukherjee , J. K. Satpathy , U. K. Bagha , M. Q. E. Mubarak , C. V. Sastri , S. P. De Visser , ACS Catal. 2021, 11, 9761–9797.

[18] I. Siewert , C. Limberg , Chem. ‐ Eur. J. 2009, 15, 10316–10328. 19780121 10.1002/chem.200901910

[19] S. Ye , X. Wu , L. Wei , D. Tang , P. Sun , M. Bartlam , Z. Rao , J. Biol. Chem. 2007, 282, 3391–3402. 17135237 10.1074/jbc.M609337200

[20] C. A. Joseph , M. J. Maroney , Chem. Comm. 2007, 41, 3338–3349. 10.1039/b702158e 18019494

[21] M. H. Stipanuk , C. R. Simmons , P. A. Karplus , J. E. Dominy , Amino Acids 2011, 41, 91–102. 20195658 10.1007/s00726-010-0518-2 PMC3136866

[22] M. Fellner , E. Siakkou , A. S. Faponle , E. P. Tchesnokov , S. P. de Visser , S. M. Wilbanks , G. N. L. Jameson , J. Biol. Inorg. Chem. 2016, 21, 501–510. 27193596 10.1007/s00775-016-1360-0

[23] C. M. Driggers , R. B. Cooley , B. Sankaran , L. L. Hirschberger , M. H. Stipanuk , P. A. Karplus , J. Mol. Biol. 2013, 425, 3121–3136. 23747973 10.1016/j.jmb.2013.05.028 PMC3744157

[24] J. Li , W. P. Griffith , I. Davis , I. Shin , J. Wang , F. Li , Y. Wang , D. J. Wherritt , A. Liu , Nat. Chem. Biol. 2018, 14, 853–860. 29942080 10.1038/s41589-018-0085-5 PMC6103799

[25] Y. Wang , I. Shin , J. Li , A. Liu , J. Biol. Chem. 2021, 297, 101176. 34508780 10.1016/j.jbc.2021.101176 PMC8503633

[26] J. E. Dominy , C. R. Simmons , L. L. Hirschberger , J. Hwang , R. M. Coloso , M. H. Stipanuk , J. Biol. Chem. 2007, 282, 25189–25198. 17581819 10.1074/jbc.M703089200

[27] N. Masson , T. P. Keeley , B. Giuntoli , M. D. White , M. Lavilla Puerta , P. Perata , R. J. Hopkinson , E. Flashman , F. Licausi , P. J. Ratcliffe , Science 2019, 365, 65–69. 31273118 10.1126/science.aaw0112 PMC6715447

[28] J. Li , R. Duan , A. Liu , J. Am. Chem. Soc. 2024, 146, 18292–18297. 38941563 10.1021/jacs.4c01871 PMC11608028

[29] E. J. Blaesi , B. G. Fox , T. C. Brunold , Biochem 2015, 54, 2874–2884. 25897562 10.1021/acs.biochem.5b00171 PMC4642275

[30] M. Sallmann , C. Limberg , Acc. Chem. Res. 2015, 48, 2734–2743. 26305516 10.1021/acs.accounts.5b00148

[31] M. Sallmann , I. Siewert , L. Fohlmeister , C. Limberg , C. Knispel , Angew. Chem., Int. Ed. 2012, 51, 2234–2237. 10.1002/anie.201107345 22287034

[32] M. Sallmann , B. Braun , C. Limberg , Chem. Comm. 2015, 51, 6785–6787. 25786780 10.1039/c5cc01083g

[33] M. Sallmann , S. Kumar , P. Chernev , J. Nehrkorn , A. Schnegg , D. Kumar , H. Dau , C. Limberg , S. P. de Visser , Chem. ‐ Eur. J. 2015, 21, 7470–7479. 25823421 10.1002/chem.201500644

[34] L. Müller , S. Hoof , M. Keck , C. Herwig , C. Limberg , Chem. ‐ Eur. J. 2020, 26, 11851–11861. 32432367 10.1002/chem.202001818 PMC7540079

[35] A. C. McQuilken , D. P. Goldberg , Dalton Trans. 2012, 41, 10883. 22814765 10.1039/c2dt30806a PMC3454461

[36] A. C. McQuilken , Y. Jiang , M. A. Siegler , D. P. Goldberg , J. Am. Chem. Soc. 2012, 134, 8758–8761. 22578255 10.1021/ja302112y PMC3403739

[37] Y. M. Badiei , M. A. Siegler , D. P. Goldberg , J. Am. Chem. Soc. 2011, 133, 1274–1277. 21207980 10.1021/ja109923a PMC3224977

[38] Y. Jiang , L. R. Widger , G. D. Kasper , M. A. Siegler , D. P. Goldberg , J. Am. Chem. Soc. 2010, 132, 12214–12215. 20712312 10.1021/ja105591q PMC2938176

[39] S. Yadav , V. Yadav , M. A. Siegler , P. Moënne‐Loccoz , G. N. L. Jameson , D. P. Goldberg , J. Am. Chem. Soc. 2024, 146, 7915–7921. 38488295 10.1021/jacs.3c12337 PMC11318076

[40] K. Anandababu , R. Ramasubramanian , H. Wadepohl , P. Comba , N. J. Britto , M. Jaccob , R. A. Mayilmurugan , Chem. ‐ Eur. J. 2019, 25 9540–9547. 31090109 10.1002/chem.201901005

[41] G. Villar‐Acevedo , P. Lugo‐Mas , M. N. Blakely , J. A. Rees , A. S. Ganas , E. M. Hanada , W. Kaminsky , J. A. Kovacs , J. Am. Chem. Soc. 2017, 139, 119–129. 28033001 10.1021/jacs.6b03512 PMC5262503

[42] A. A. Fischer , N. Stracey , S. V. Lindeman , T. C. Brunold , A. T. Fiedler , Inorg. Chem. 2016, 55, 11839–11853. 27801576 10.1021/acs.inorgchem.6b01931 PMC5548292

[43] A. A. Fischer , J. R. Miller , R. J. Jodts , D. M. Ekanayake , S. V. Lindeman , T. C. Brunold , A. T. Fiedler , Inorg. Chem. 2019, 58, 16487–16499. 31789510 10.1021/acs.inorgchem.9b02432 PMC6956612

[44] A. A. Fischer , S. V. Lindeman , A. T. Fiedler , Dalton Trans. 2017, 46, 13229–13241. 28686274 10.1039/c7dt01600j

[45] C. R. Simmons , Q. Liu , Q. Huang , Q. Hao , T. P. Begley , P. A. Karplus , M. H. Stipanuk , J. Biol. Chem. 2006, 281, 18723–18733. 16611640 10.1074/jbc.M601555200

[46] D. Kumar , W. Thiel , S. P. de Visser , J. Am. Chem. Soc. 2011, 133, 3869–3882. 21344861 10.1021/ja107514f

[47] E. J. Blaesi , J. D. Gardner , B. G. Fox , T. C. Brunold , Biochem 2013, 52, 6040–6051. 23906193 10.1021/bi400825c PMC3974268

[48] S. C. Chai , A. A. Jerkins , J. J. Banik , I. Shalev , J. L. Pinkham , P. C. Uden , M. J. Maroney , J. Biol. Chem. 2005, 280, 9865–9869. 15623508 10.1074/jbc.M413733200

[49] R. L. Fernandez , N. D. Juntunen , T. C. Brunold , Acc. Chem. Res. 2022, 55, 2480–2490. 35994511 10.1021/acs.accounts.2c00359 PMC9583696

[50] J. D. Gardner , B. S. Pierce , B. G. Fox , T. C. Brunold , Biochem 2010, 49, 6033–6041. 20397631 10.1021/bi100189h PMC2914100

[51] J. C. A. Boeyens , Z. Naturforsch. 2008, 63, 199–209.

[52] F. Biegler‐König , J. Schönbohm , J. Comput. Chem. 2002, 23, 1489–1494. 12370951 10.1002/jcc.10085

[53] F. Neese , WIREs Comput. Mol. Sci. 2012, 2, 73–78.

[54] E. P. Tchesnokov , S. M. Wilbanks , G. N. L. Jameson , Biochem 2012, 51, 257–264. 22122511 10.1021/bi201597w

[55] Y. Wang , I. Davis , Y. Chan , S. G. Naik , W. P. Griffith , A. Liu , J. Biol. Chem. 2020, 295, 11789–11802. 32601061 10.1074/jbc.RA120.013915 PMC7450127

[56] A. T. Fiedler , A. A. Fischer , J. Biol. Inorg. Chem. 2017, 22, 407–424. 27853875 10.1007/s00775-016-1402-7

[57] J. B. Gordon , A. C. Vilbert , M. A. Siegler , K. M. Lancaster , P. Moënne‐Loccoz , D. P. Goldberg , J. Am. Chem. Soc. 2019, 141, 3641–3653. 30776222 10.1021/jacs.8b13134 PMC6556537

[58] S. Sahu , D. P. Goldberg , J. Am. Chem. Soc. 2016, 138, 11410–11428. 27576170 10.1021/jacs.6b05251 PMC5228556

[59] There are Co‐based quercetin dioxygenase models known, which, however, function via substrate activation (free superoxide).^[^ ^94^, ^95^ ^]^

[60] H. Noh , D. Jeong , T. Ohta , T. Ogura , J. S. Valentine , J. Cho , J. Am. Chem. Soc. 2017, 139, 10960–10963. 28758392 10.1021/jacs.7b04479

[61] C. C. G. Yeh , C. Pierides , G. N. L. Jameson , S. P. de Visser , Chem. ‐ Eur. J. 2021, 27, 13793–13806. 34310770 10.1002/chem.202101878

[62] A. S. Faponle , F. P. Seebeck , S. P. de Visser , J. Am. Chem. Soc. 2017, 139, 9259–9270. 28602090 10.1021/jacs.7b04251

[63] S. Aluri , S. P. de Visser , J. Am. Chem. Soc. 2007, 129, 14846–14847. 17994747 10.1021/ja0758178

[64] P. Wu , Y. Gu , L. Liao , Y. Wu , J. Jin , Z. Wang , J. Zhou , S. Shaik , B. Wang , Angew. Chem., Int. Ed. 2022, 61, e202214235. 10.1002/anie.202214235 36259368

[65] C. A. Brown , M. A. Pavlovsky , T. E. Westre , Y. Zhang , B. Hedman , K. O. Hodgson , E. I. Solomon , J. Am. Chem. Soc. 1995, 117, 577–840.

[66] Y. Wang , L. Yan , X. Li , S. Zhang , J. Wei , Y. Liu , Inorg. Chem. 2021, 60, 7844–7856. 34008401 10.1021/acs.inorgchem.1c00340

[67] G. Tian , H. Su , Y. Liu , ACS Catal. 2018, 8, 5875–5889.

[68] W. J. Wei , P. E. M. Siegbahn , R. Z. Liao , Inorg. Chem. 2017, 56, 3589–3599. 28277674 10.1021/acs.inorgchem.6b03177

[69] A. Mirzahosseini , B. Noszál , Sci. Rep. 2016, 6, 37596. 27869189 10.1038/srep37596 PMC5116634

[70] T. Palla , A. Mirzahosseini , B. Noszál , Antioxidants 2020, 9, 465. 32492814 10.3390/antiox9060465 PMC7346207

[71] Y. Cao , S. Hay , S. P. de Visser , J. Am. Chem. Soc. 2024, 146, 11726–11739. 38636166 10.1021/jacs.3c14574 PMC11066847

[72] H. S. Ali , S. P. de Visser , Front. Chem. 2024, 12, 1365494. 38406558 10.3389/fchem.2024.1365494 PMC10884159

[73] S. Goudarzi , J. T. Babicz Jr. , O. Kabil , R. Banerjee , E. I. Solomon , J. Am. Chem. Soc. 2018, 140, 14887–14902. 30362717 10.1021/jacs.8b09022 PMC6221977

[74] M. K. Goetz , E. A. Hill , A. S. Filatov , J. S. Anderson , J. Am. Chem. Soc. 2018, 140, 13176–13180. 30078327 10.1021/jacs.8b07399

[75] A. L. Rheingold , C. B. White , S. Trofimenko , Inorg. Chem. 1993, 32, 3471–3477.

[76] R. J. C. Dubey , R. J. Comito , Z. Wu , G. Zhang , A. J. Rieth , C. H. Hendon , J. T. Miller , M. Dincǎ , J. Am. Chem. Soc. 2017, 139, 12664–12669. 28783434 10.1021/jacs.7b06841

[77] H. Myamoto , Y. Yampolski , C. L. Young , J. Phys. Chem. Ref. Data 2014, 43, 1–209.

[78] D. Kass , T. Corona , K. Warm , B. Braun‐Cula , U. Kuhlmann , E. Bill , S. Mebs , M. Swart , H. Dau , M. Haumann , P. Hildebrandt , K. Ray , J. Am. Chem. Soc. 2020, 142, 5924–5928. 32168447 10.1021/jacs.9b13756

[79] G. A. Bain , J. F. Berry , J. Chem. Educ. 2008, 85, 532–536.

[80] B. N. Figgis , Nature 1958, 182, 1568–1570.

[81] G. M. Sheldrick , SADABS, Program for Empirical Absorption Correction of Area Detector Data, University of Göttingen, Göttingen (Germany) 1996.

[82] G. M. Sheldrick , Acta Crystallogr. A 2015, 71, 3–8. 10.1107/S2053273314026370 PMC4283466 25537383

[83] G. M. Sheldrick , Acta Crystallogr. C Struct. Chem. 2015, 71, 3–8. 25567568 10.1107/S2053229614024218 PMC4294323

[84] C. B. Hübschle , G. M. Sheldrick , B. Dittrich , J. Appl. Crystallogr. 2011, 44, 1281–1284. 22477785 10.1107/S0021889811043202 PMC3246833

[85] A. L. Spek , Acta Crystallogr. C Struct. Chem. 2015, 71, 9–18. 25567569 10.1107/S2053229614024929

[86] M. J. Frisch , G. W. Trucks , H. B. Schlegel , G. E. Scuseria , M. A. Robb , J. R. Cheeseman , G. Scalmani , V. Barone , B. Mennucci , G. A. Petersson , H. Nakatsuji , M. Caricato , X. Li , H. P. Hratchian , A. F. Izmaylov , Y. Honda , O. Kitao , H. Nakai , T. Vreven , J. A. Montgomery Jr. , J. E. Peralta , F. Ogliaro , M. Bearpark , J. J. Heyd , E. Brothers , K. N. Kudin , V. N. Staroverov , R. Kobayashi , J. Normand , K. Raghavachari , et al., Gaussian 09, Gaussian Inc., Wallingford CT (USA) 2009.

[87] A. D. Becke , J. Chem. Phys. 1993, 98, 5648–5652.

[88] C. Lee , W. Yang , R. G. Parr , Phys. Rev. B 1988, 37, 785–789. 10.1103/physrevb.37.785 9944570

[89] P. J. Hay , W. R. Wadt , J. Chem. Phys. 1985, 82, 270–283.

[90] R. Ditchfield , W. J. Hehre , J. A. Pople , J. Chem. Phys. 1971, 54, 724–728.

[91] M. M. Francl , W. J. Pietro , W. J. Hehre , J. S. Binkley , M. S. Gordon , D. J. DeFrees , J. A. Pople , J. Chem. Phys. 1982, 77, 3654–3665.

[92] J. Tomasi , B. Mennucci , R. Cammi , Chem. Rev. 2005, 105, 2999–3094. 16092826 10.1021/cr9904009

[93] M. Römelt , S. Ye , F. Neese , Inorg. Chem. 2009, 48, 784–785. 19102678 10.1021/ic801535v

[94] S. Hoof , C. Limberg , Inorg. Chem. 2019, 58, 12843–12853. 31502453 10.1021/acs.inorgchem.9b01795

[95] N. Podder , A. Saha , S. K. Barman , S. Mandal , Dalton Trans. 2023, 52, 11465–11480. 37466296 10.1039/d3dt00833a

