# Supplementary material for: Promiscuity in Molecular Mimics of the Cysteine Dioxygenase: Effects of Selenium in the Substrate and Cobalt as the Central Metal Ion
Source: Angew Chem Int Ed Engl. 2025 Aug 19;64(40):e202507578. doi: 10.1002/anie.202507578 (PMC12462750; doi:10.1002/anie.202507578)
Supplement: Supplementary file 1 — Supporting Information [file ANIE-64-e202507578-s002.docx]

***Supporting Information***

Promiscuity in Molecular Mimics of the Cysteine Dioxygenase: Effects of Selenium in the Substrate and Cobalt as the Central Metal Ion

Kilian Weißer,^[a]^ Edgar T. K. Weber,^[a]^ Gunasekaran Velmurugan,^[b]^ Beatrice Cula,^[a]^ Konstantin B. Krause,^[a]^ Siad Wolff,^[a]^ M. Qadri. E. Mubarak,^[c,d,e]^, Peter Comba,^[b]^ Sam P. de Visser,*^[c,d]^ and Christian Limberg*^[a]^

[a] K. Weißer, E. T. K. Weber, Dr. B. Cula, Dr. K. B. Krause, S. Wolff, Prof. Dr. C. Limberg
Institut für Chemie
Humboldt-Universität zu Berlin
Brook-Taylor Straße 2, 12489 Berlin (Germany)
E-mail: christian.limberg@hu-berlin.de

[b] Dr. G. Velmurugan, Prof. Dr. P. Comba
Institut für Anorganische Chemie
Universität Heidelberg
Im Neuenheimer Feld 270, 69120 Heidelberg (Germany)

[c] Dr. M. Q. E. Mubarak, Dr. S. P. de Visser
Manchester Institute of Biotechnology
The University of Manchester
131 Princess Street, Manchester M1 7DN (United Kingdom)
E-mail: sam.devisser@manchester.ac.uk

[d] Dr. M. Q. E. Mubarak, Dr. S. P. de Visser
Department of Chemical Engineering
The University of Manchester
Oxford Road, Manchester M13 9PL (United Kingdom)
E-mail: sam.devisser@manchester.ac.uk

[e] Dr. M. Q. E. Mubarak (present address)
Program Teknologi Kimia Industri
Universiti Sains Islam Malaysia
71800 Bandar Baru Nilai, Malaysia

***Experimental procedures***

**General methods**

All experiments were carried out in a dry argon atmosphere using a *MBraun* or *GS* glovebox and/or standard *Schlenk*-techniques. Et_2_O, hexane, DCM, chlorobenzene, MeCN and THF were purified employing a *MBraun* Solvent Purification System SPS, degassed *via* freeze-pump-thaw and stored in *Young*-ampules over MS 3 Å or MS 4 Å (for MeCN). Methanol was dried with CaH_2_, distilled, and stored in a *Young*-ampule over MS 4 Å. NEt_3_ was dried with KOH, distilled and stored in a *Young*-ampule over MS 3 Å. Deuterated solvents were stored over MS 3 Å for 48 h before use. All materials were obtained from commercial vendors as ACS reagent-grade or better and used as received. NMR spectra were recorded with a *Bruker* Avance DPX 300 spectrometer at room temperature. Mößbauer spectra were recorded at 14 K with a *SeeCo* MS6 spectrometer and a *Janis* CCS-850 cryostat with a *CTI-Cryogenics* 8200 helium compressor. The temperature was controlled with a *LakeShore* 335 thermocontrol. UV-Vis spectra were obtained at variable temperatures with an *Agilent* 8453 UV-Vis spectrophotometer equipped with a *Unisoku* USP-203-A cryostat. SUPRASIL Quartz cells from *Hellma Analytics* with a 10 mm path length were used. Microanalyses were performed with a HEKAtech Euro EA 3000 elemental analyzer. ATR-infrared (IR) spectra were recorded with a *Bruker* alpha FTIR spectrometer in the region 4000–400 cm^–1^. High resolution mass spectra were recorded with an *Agilent Technologies* 6230 LC/TOF instrument with electro-spray ionization (ESI-MS). The complex precursors Tp^Mes^FeCl, Tp^Mes*^FeCl^[1]^ and Tp^Mes^K∙Et_2_O^[2]^ were prepared as published.

**Selenocysteamine, triethylammonium salt**

Selenocysteamine dihydrochloride (12.5 mg, 0.039 mmol, 1 eq.) was suspended in water (0.5 mL) and NaBH_4_ (1.03 mg, 0.058, 1.5 eq.) was added. The mixture was stirred for 30 s, and all volatiles were subsequently removed at 80 °C under reduced pressure. The obtained white solid was dissolved in MeOH (5 mL) and NEt_3_ (0.1 mL) was added. The free selenolate was used in the subsequent reaction without further purification.

^77^Se-NMR (95 MHz, MeOD-d_4_): δ = -253.57 ppm.

**Tp^Mes/*^FeSeCysAm (4a/b)**

The free selenolate (29 mg, 0.182 mmol, 1.2 eq.) in MeOH was used after the reduction without further purification and slowly added to a solution of Tp^Mes/*^FeCl (100 mg, 0.152 mmol, 1 eq.) in THF (5 mL) at -78 °C. The mixture was stirred for 2 h and all volatiles were removed under reduced pressure. The crude product was dissolved in Et_2_O (10 mL) and subsequently filtered at -78 °C. The desired product was obtained in moderate yields (**4a**: 69.2 mg, 0.093 mmol, 61% and **4b**: 57.9 mg, 0.776 mmol, 56%) as a light-yellow solid.

**4a:** **^1^H-NMR** (300 MHz, C_6_D_6_): δ = 49.66, 41.19, 16.92, 8.10, 3.58, -0.48, -22.09 ppm. The signal of the borate-*H* is too broad to be observable.

**ATR-IR (solid):** $\tilde{\nu}$ = 3357.44, 3281.45 ([ν(N-H)]), 2475.90, 2451.42 [ν(B-H)] cm^-1^.

**EA:** calculated for C_38_H_46_BFeN_7_Se (M = 746.46 g/mol): C: 61.14, H: 6.21, N: 13.14; measured: C: 61.25, H: 6.33, N: 13.23.

**ESI-MS:** m/z calculated for [C_38_H_46_BFeN_7_Se]^+^ 747.2422; found 747.2423.

**4b**: **^1^H-NMR** (300 MHz, C_6_D_6_): δ = 69.74, 50.85, 46.07, 31.62, 19.14, 10.59, 1.04, 0.26, -0.44, -2.26, -5.24, -8.90, -30.84, -33.56 ppm.

**ATR-IR (solid):** $\tilde{\nu}$ = 3351.40, 3275.04 ([ν(N-H)]), 2494.99, 2485.44 [ν(B-H)] cm^-1^.

**EA**: calculated for C_38_H_46_BFeN_7_Se (M = 746.46 g/mol): C: 61.14, H: 6.21, N: 13.14; measured: C: 61.44, H: 6.24, N: 12.98.

**ESI-MS:** m/z calculated [C_38_H_46_BFeN_7_Se + C_36_H_40_BFeN_6_ + MeOH]^+^ 1402.5441; found 1402.5111.

**Tp^Mes^FeO_2_SeCysAm (5a)**

Complex **4a** (25.9 mg, 0.035 mmol) was dissolved in Et_2_O (5 mL) and the solution was degassed twice *via* freeze-pump-thaw. The evacuated flask was cooled in an ice bath, filled with 1.5 bar of dry oxygen, and the reaction mixture was subsequently stirred for 15 min. All volatiles were removed *in vacuo*, the remaining solid was washed with cold toluene (2 mL) and the product isolated as a colorless solid in good yields (26.8 mg, 0.034 mmol, 98%). Crystals suitable for X-ray diffraction could be grown by the evaporation of DCM from a solution with chlorobenzene.

**^1^H-NMR** (300 MHz, CDCl_3_): δ = 48.45, 12.23, 10.27, 7.26, 2.54, -16.31, -34.75 ppm. The signal of the borate-*H* is too broad to be observed.

**ATR-IR (solid):** $\tilde{\nu}$ = 3361.75, 3283.64 ([ν(N-H)]), 2461.19, 2440.94 [ν(B-H)] cm^-1^.

**EA:** calculated for C_38_H_46_BFeN_7_O_2_Se (M = 778.46 g/mol): C: 58.63, H: 5.96, N: 12.60; measured: C: 58.98, H: 6.06, N: 12.18.

**ESI-MS:** m/z calculated [C_38_H_46_BFeN_7_O_2_Se]^+^ 779.2321; found 779.2324.

**Tp^Mes*^FeO_2_SeCysAm (5b)**

Complex **4b** (50 mg, 0.07 mmol, 1.0 eq.) was dissolved in Et_2_O and the solution was cooled inside an ice bath. A two-neck flask was attached to an oxygen-line with a bubbler to provide atmospheric pressure. To avoid further oxidation of the product complex **5b** to the oxidation state +III, a stochiometric amount of oxygen gas was withdrawn with a syringe (1.79 ml, 0.07 mmol, 1.0 eq.) and added to the stirred solution of **4b**. After one hour all volatiles were removed under reduced pressure and the product was obtained in quantitative yields (50.1 mg, 0.0.64 mmol, 96%) as a colorless solid.

**^1^H-NMR** (300 MHz, C_6_D_6_): δ = 50.26, 47.20, 12.17, 10.64, 9.59, 8.67, 5.52, 3.58, 2.14, 0.97, -2.28, -14.39, -33.49 ppm.

**ATR-IR (solid):** $\tilde{\nu}$ = 3356.51, 3283.45 ([ν(N-H)]), 2483.27 [ν(B-H)] cm^-1^.

**EA:** calculated for C_38_H_46_BFeN_7_O_2_Se (M = 778.46 g/mol): C: 58.63, H: 5.96, N: 12.60; measured C: 59.10, H: 6.15, N: 12.27.

**ESI-MS:** m/z calculated [C_38_H_46_BFeN_7_O_2_Se]^+^ 779.2321; found 779.2321.

**Tp^Mes^CoCl (6)**

The precursor complex KTp^Mes^∙Et_2_O (173 mg, 0.285 mmol, 1 eq.) and CoCl_2_ (37 mg, 0.285 mmol, 1 eq.) were suspended in DCM and stirred overnight. All insoluble residues were filtered off and the clear solution was subsequently reduced to dryness. The resulting blue solid was washed twice with MeCN, and all volatiles were removed under reduced pressure. The desired complex was isolated in good yields (158.7 mg, 0.238 mmol, 84 %).

The obtained analytical data are in good agreement with data reported in the literature.^[3]^

**^1^H-NMR (300 MHz, CDCl_3_):** δ = 72.47, 37.45, 11.12, 6.54, 1.48, -29.13 ppm.

**Tp^Mes^CoSeCysAm (7)**

The free selenolate (1.2 eq.) in MeOH was used after the reduction without further purification and slowly added dropwise to a solution of Tp^Mes^CoCl **6** (50.0 mg, 0.076 mmol, 1 eq.) in THF (5 mL) at -78 °C. During the addition, the color changed from deep blue to bright orange. The mixture was stirred for 2 h and all volatiles were removed under reduced pressure. The crude product was dissolved in cold Et_2_O (10 mL) and subsequently filtered at -78 °C. The desired product **7** was obtained in moderate yields (33.3 mg, 0.043 mmol, 64%) as a red solid.

**^1^H-NMR (300 MHz, C_6_D_6_):** δ = 106.56, 79.64, 60.75, 43.92, 1.65, -6.14, -50.04 ppm. The signal of the borate-*H* is too broad to be observable.

**ATR-IR (solid):** $\tilde{\nu}$ = 3359.33, 3282.32 ([ν(N-H)]), 2479.76, 2453.50 [ν(B-H)] cm^-1^.

**EA:** calculated for C_38_H_46_BCoN_7_Se (M = 749.55 g/mol): C: 60.89, H: 6.19, N: 13.08; measured C: 60.50, H: 6.27, N: 12.54.

**ESI-MS:** m/z calculated [C_38_H_476_BCoN_7_Se + H^+^] 751.2478; found 751.2473.

**Tp^Mes^CoO_2_SeCysAm (8)**

Complex **7** (20 mg, 0.026 mmol) was dissolved in Et_2_O and the solution was degassed twice *via* freeze-pump-thaw. The evacuated flask was filled with 1.5 bar of dry oxygen, and the reaction mixture was subsequently stirred for 4 hours at 40 °C. All volatiles were removed *in vacuo*, the resulting solid was washed with cold toluene (2 mL) and the desired product was isolated as a light pink solid in very good yields (17.6 mg, 0.023 mmol, 88%). Crystals suitable for X-ray diffraction could be grown by the evaporation of DCM from a solution with chlorobenzene.

**^1^H-NMR** (300 MHz, CDCl_3_): δ = 102.64, 73.78, 43.94, 2.63, -4.71, -48.26 ppm. The signal of the borate-*H* is too broad to be observable.

**ATR-IR (solid):** $\tilde{\nu}$ = 3363.33, 3284.01 ([ν(N-H)]), 2464.19, 2442.01 [ν(B-H)] cm^-1^.

**EA:** calculated for C_38_H_46_BFeN_7_O_2_Se (M = 781.55 g/mol): C: 58.40, H: 5.93, N: 12.55, measured C: 58.23, H: 5.23, N: 12.38.

**ESI-MS:** m/z calculated [C_38_H_46_BFeN_7_SeO_2_ + C_36_H_40_BCoN_6_] 1408.5043; found 1408.5100.

**NMR-spectroscopy**

**
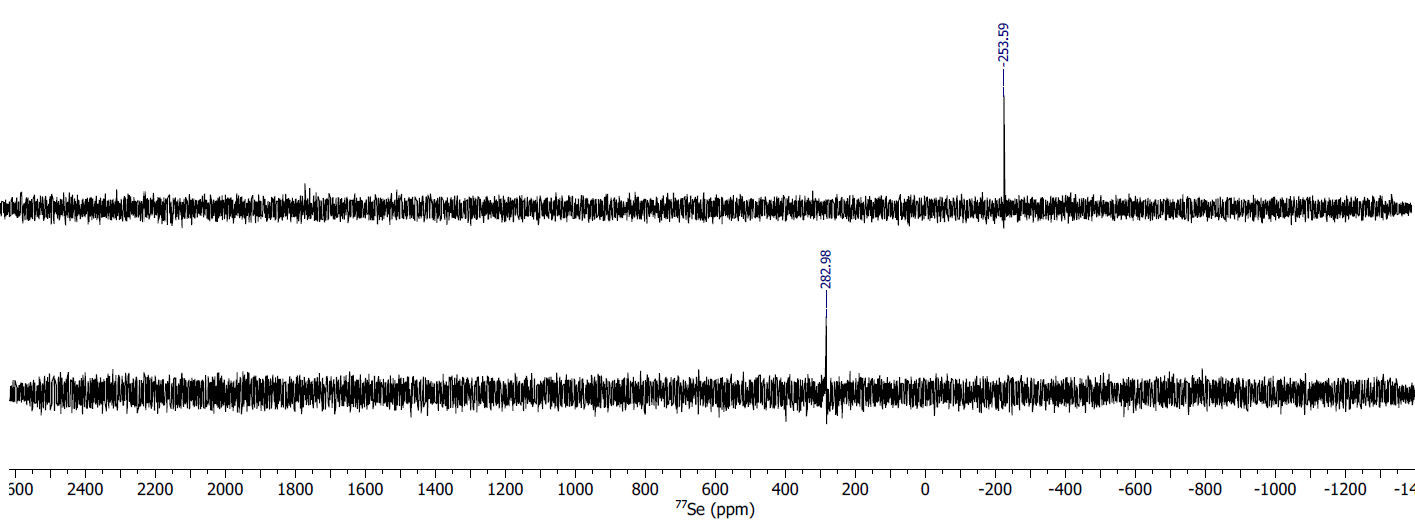
**

Figure S1. ^77^Se NMR spectrum (95 MHz) of selenocysteamine dihydrochloride (bottom) and selenocysteamine, triethylammonium salt (top) dissolved in MeOD-d_4_.


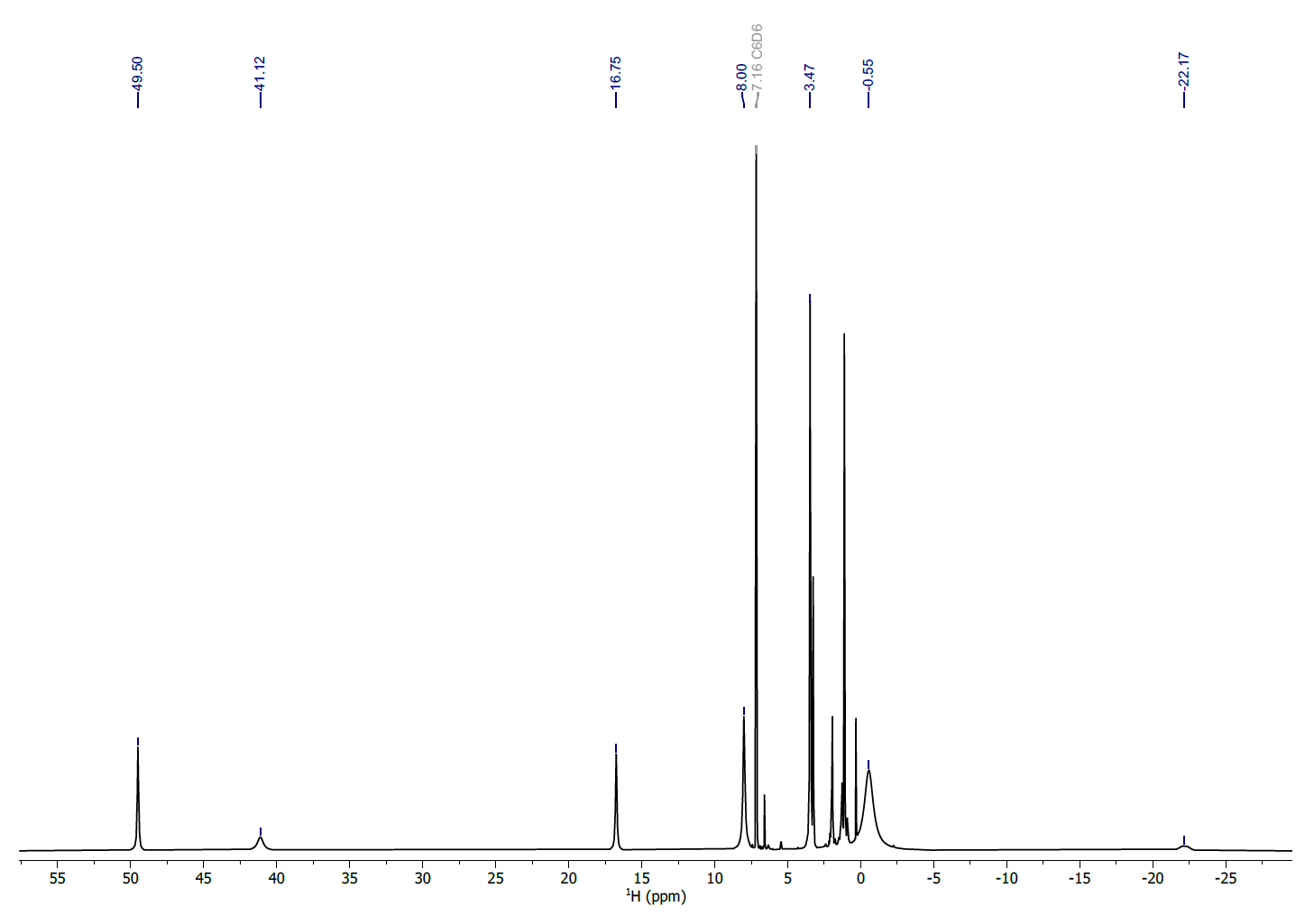


#

#

**Figure S2.** Paramagnetic ^1^H NMR spectrum (300 MHz) of [Tp^Mes^FeSeCysAm] **4a** dissolved in C_6_D_6_, residual solvent (Et_2_O) is marked with #.

***
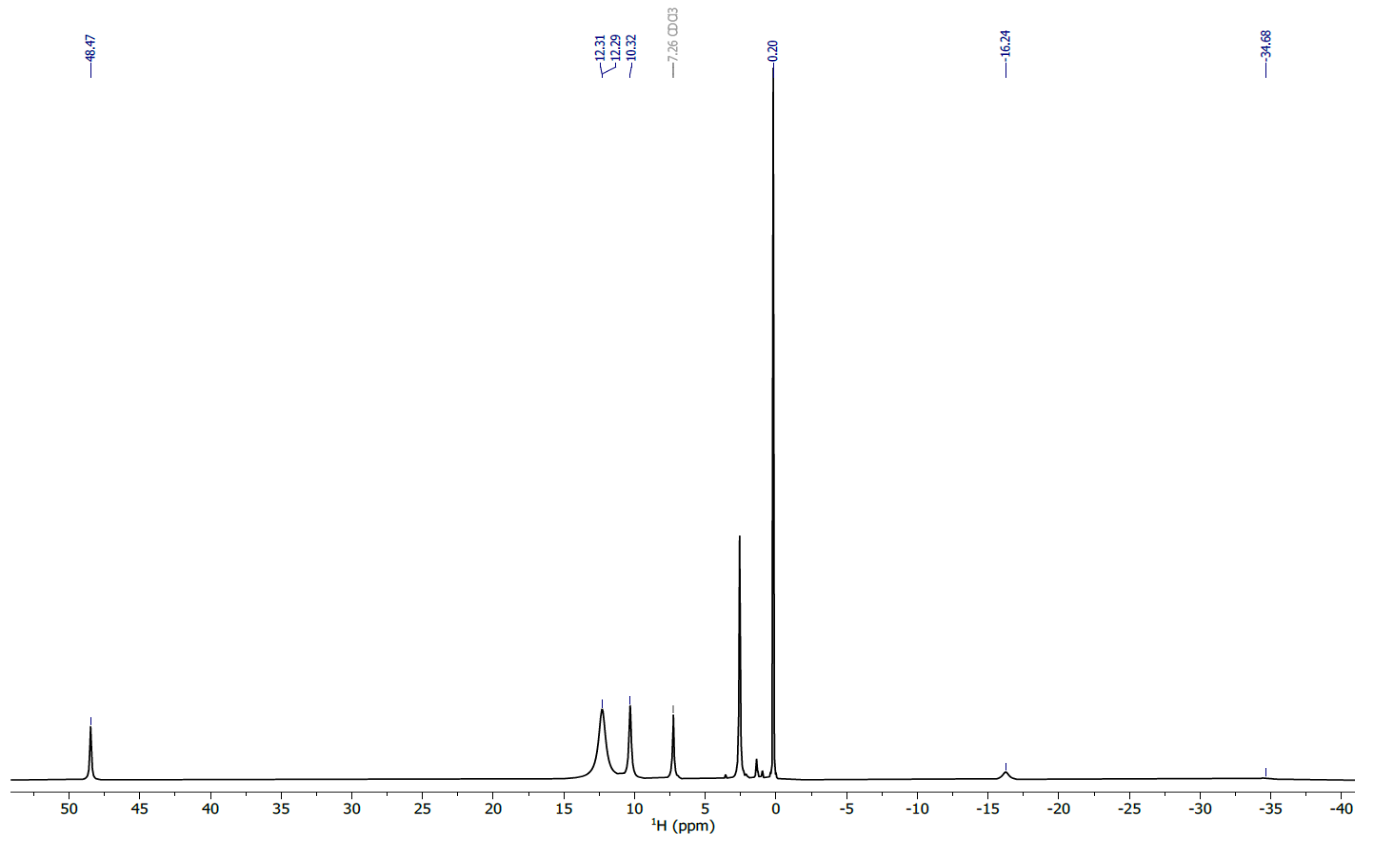
***

#

Figure S3. Paramagnetic ^1^H NMR spectrum (300 MHz) of [Tp^Mes^FeO_2_SeCysAm] 5a dissolved in C_6_D_6_, residual solvent (hexane) is marked with #.

***
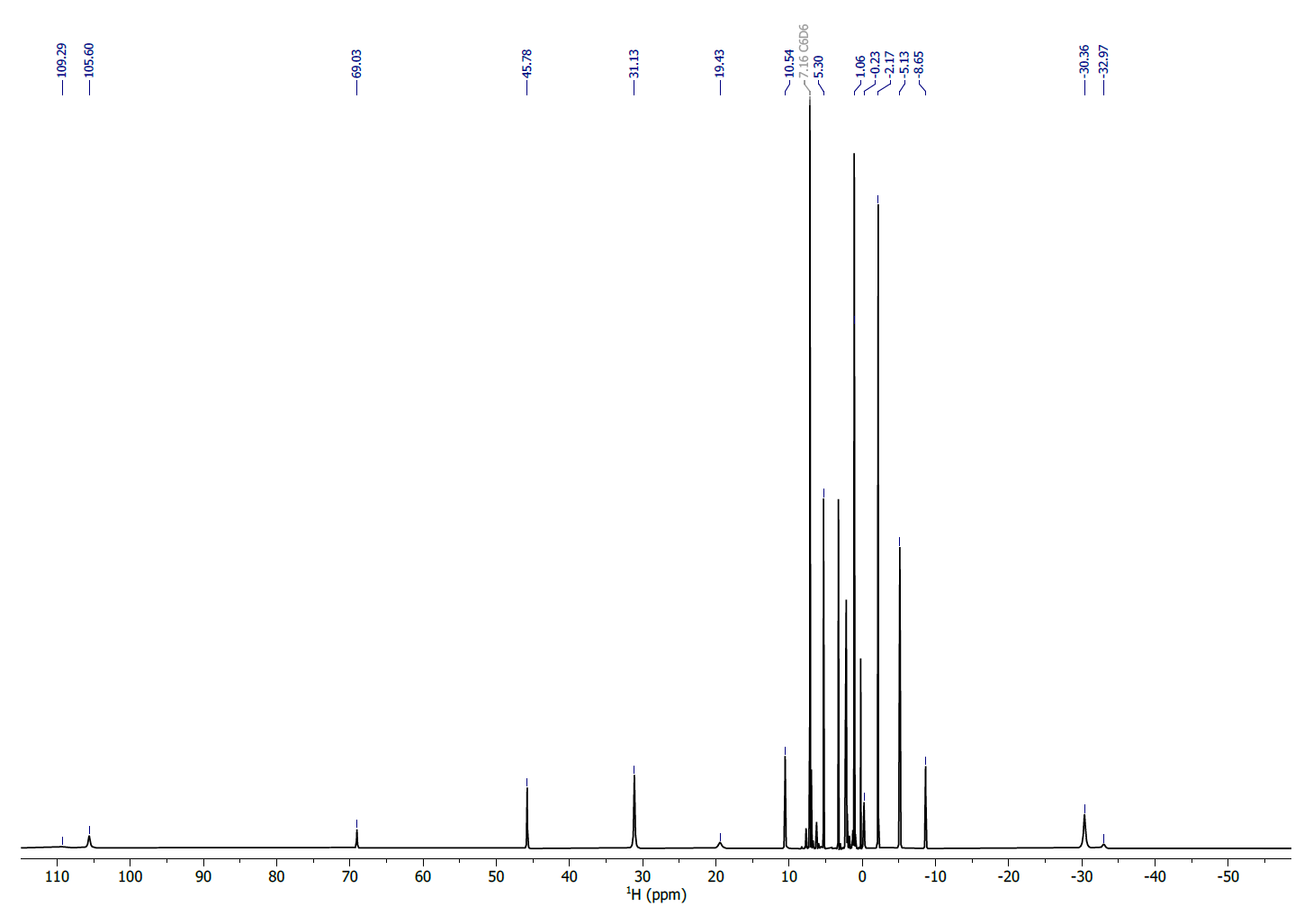
***

#

#

Figure S4. Paramagnetic ^1^H NMR spectrum (300 MHz) of [Tp^Mes*^FeSeCysAm] 4b dissolved in C_6_D_6_, residual solvent (Et_2_O) is marked with #.


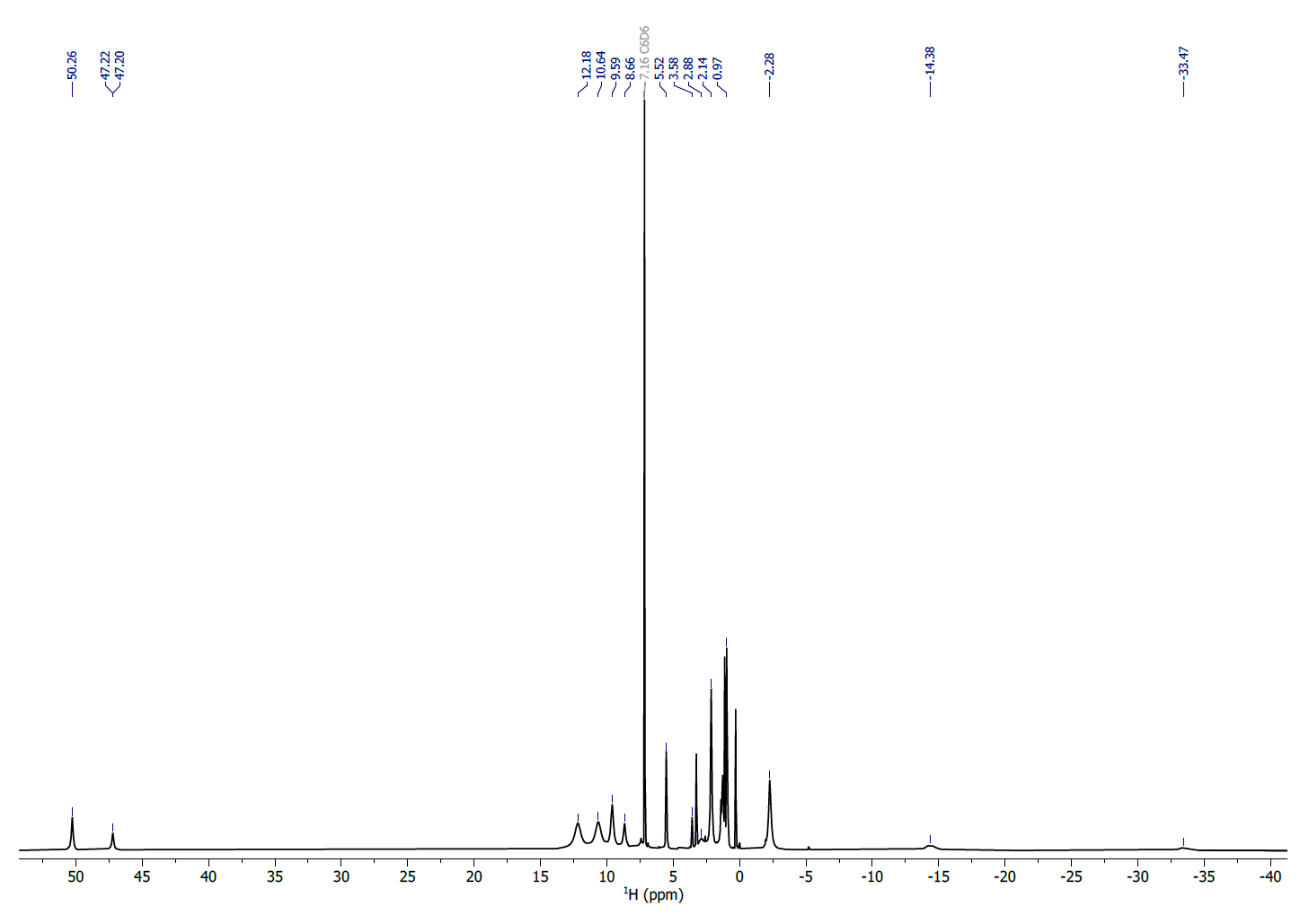


# /#’

#’

#

Figure S5. Paramagnetic ^1^H NMR spectrum (300 MHz) of [Tp^Mes*^FeO_2_SeCysAm] 5b dissolved in C_6_D_6_, residual solvent is marked with # (Et_2_O) and #’ (hexane).


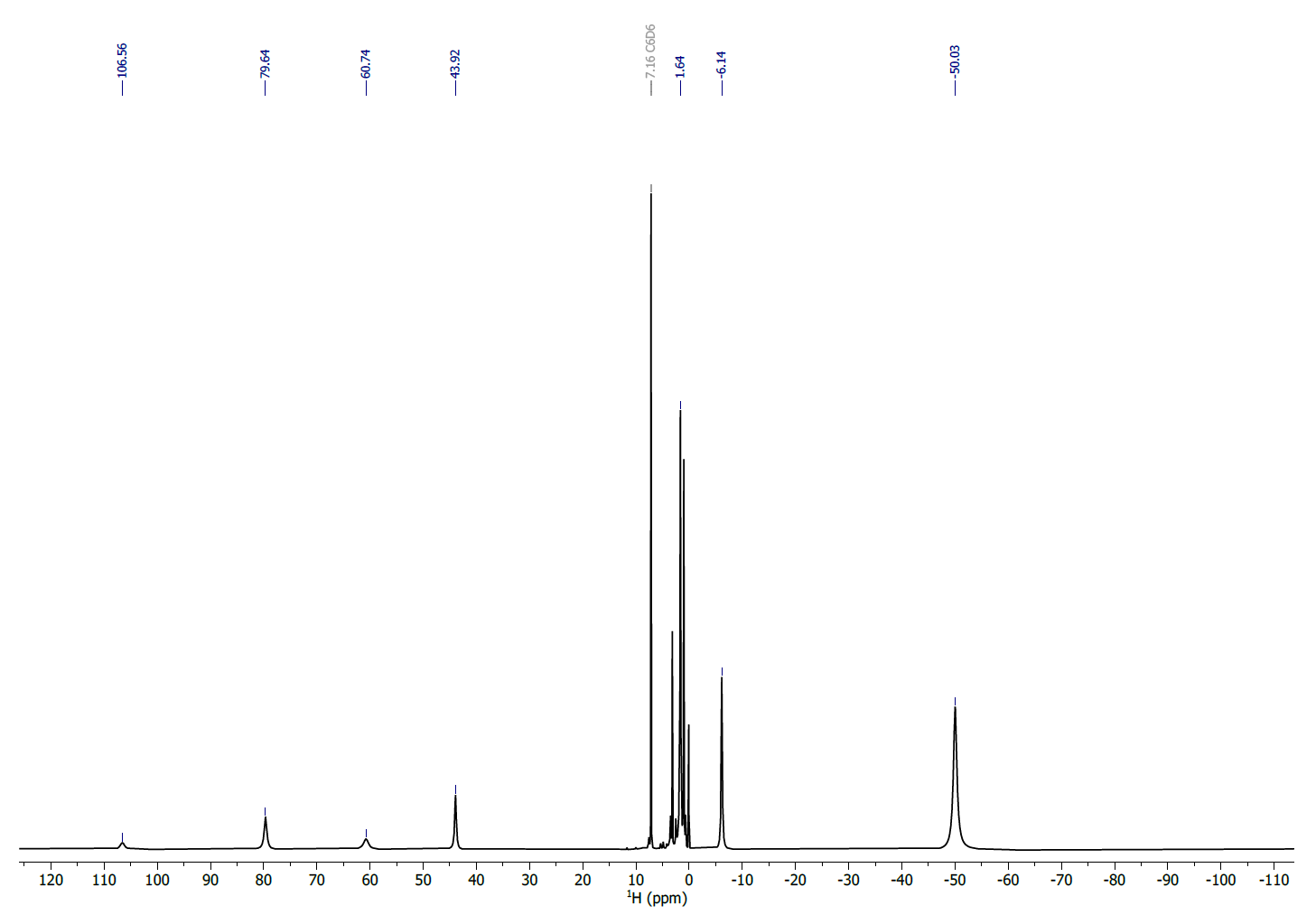


#

#

Figure S6. Paramagnetic ^1^H NMR spectrum (300 MHz) of [Tp^Mes^CoSeCysAm] 7 dissolved in C_6_D_6_, residual solvent (Et_2_O) is marked with #.


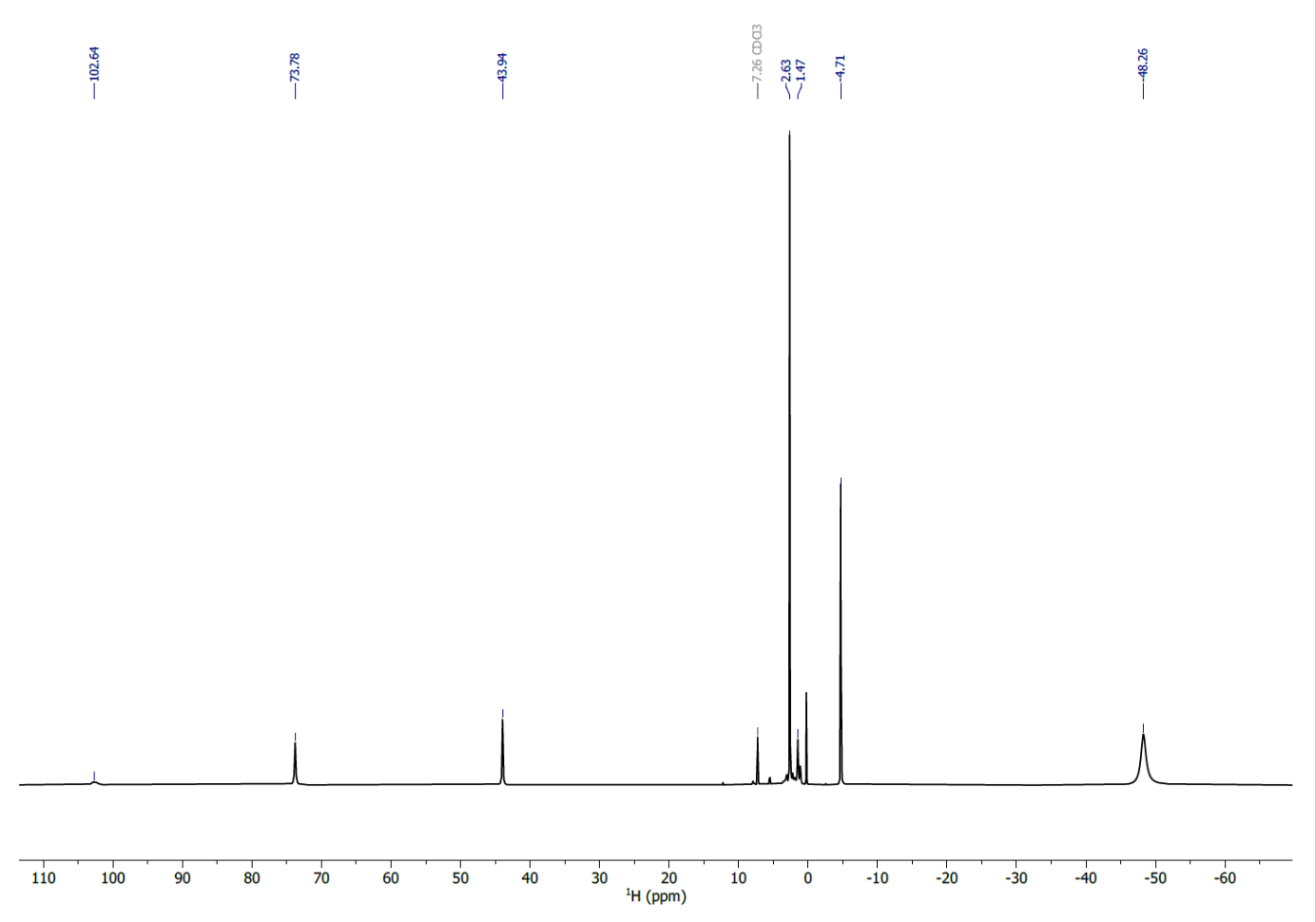


#

Figure S7. Paramagnetic ^1^H NMR spectrum (300 MHz) of [Tp^Mes^CoO_2_SeCysAm] dissolved 8 in CDCl_3_, residual solvent (MeOH) is marked with # (MeOH).

**ATR-IR spectroscopy**

**
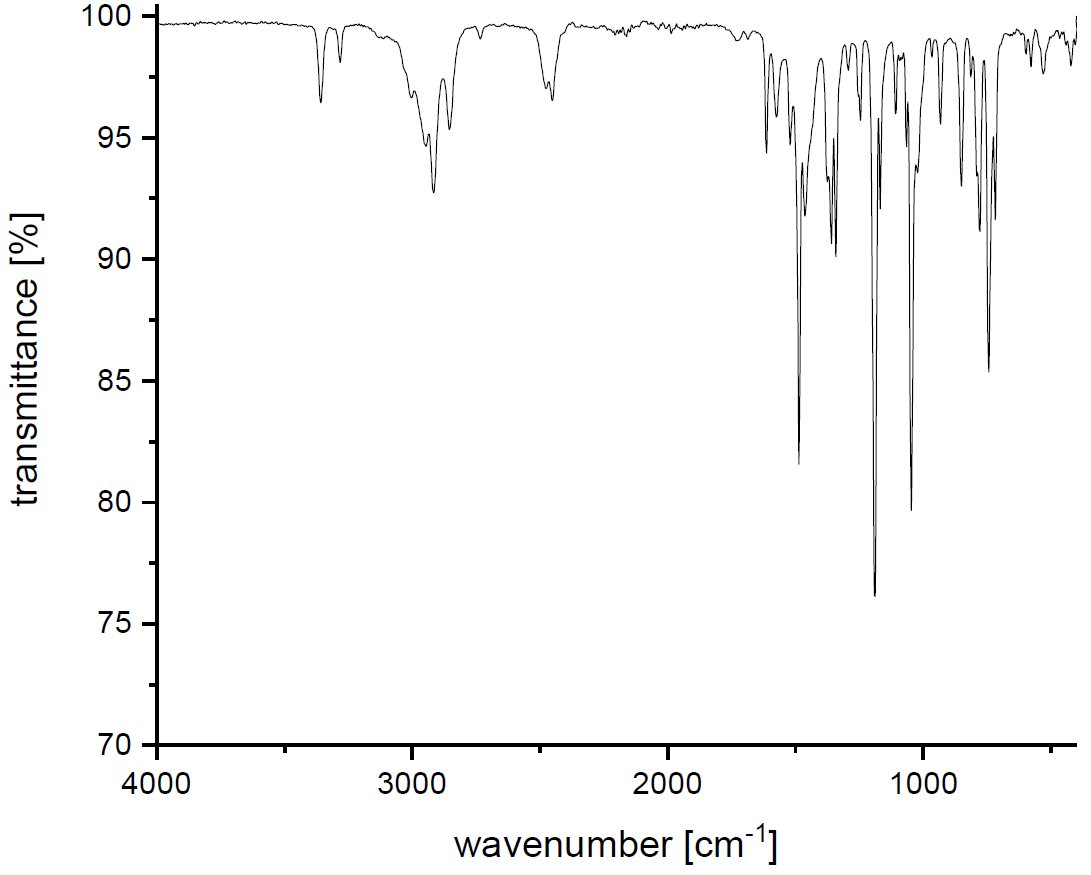
**

Figure S8. ATR-IR spectra (solid) of [Tp^Mes^FeSeCysAm] 4a.


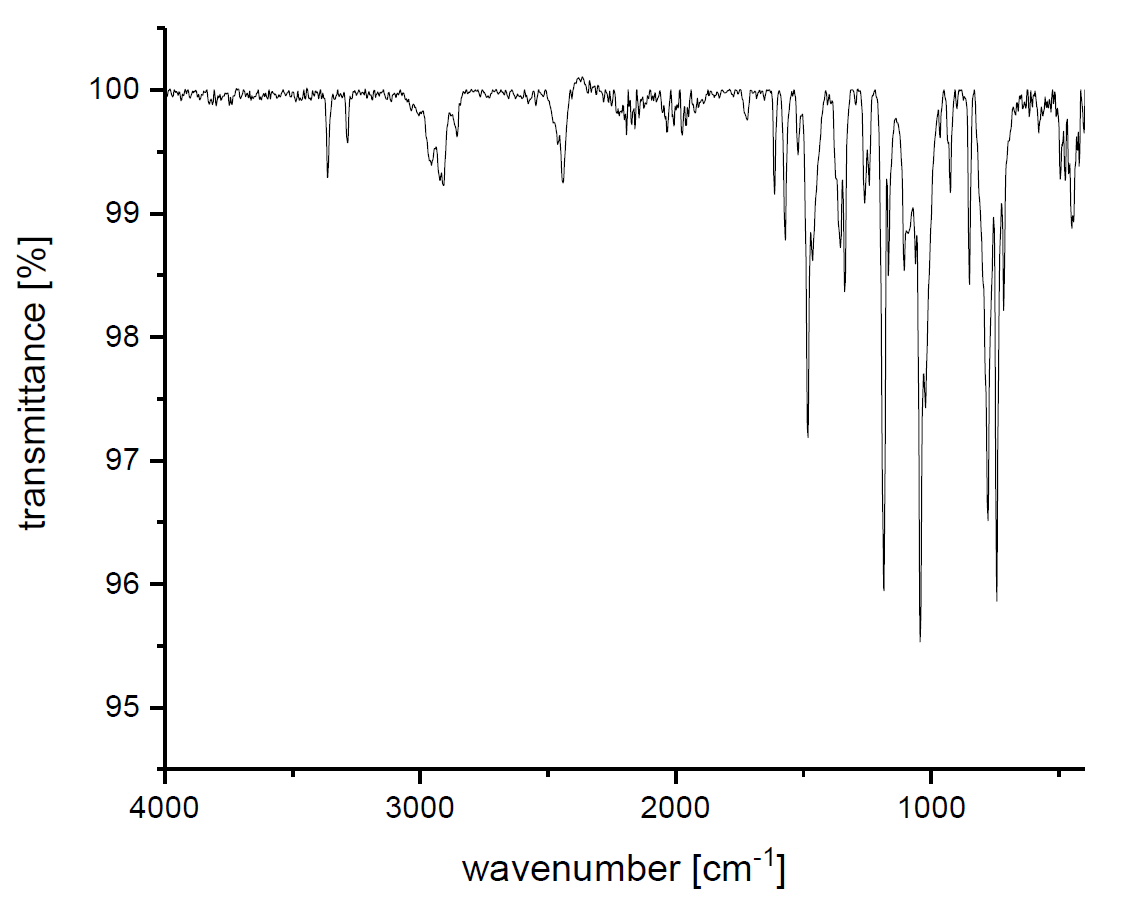


Figure S9. ATR-IR spectra (solid) of [Tp^Mes^FeO_2_SeCysAm] 5a.


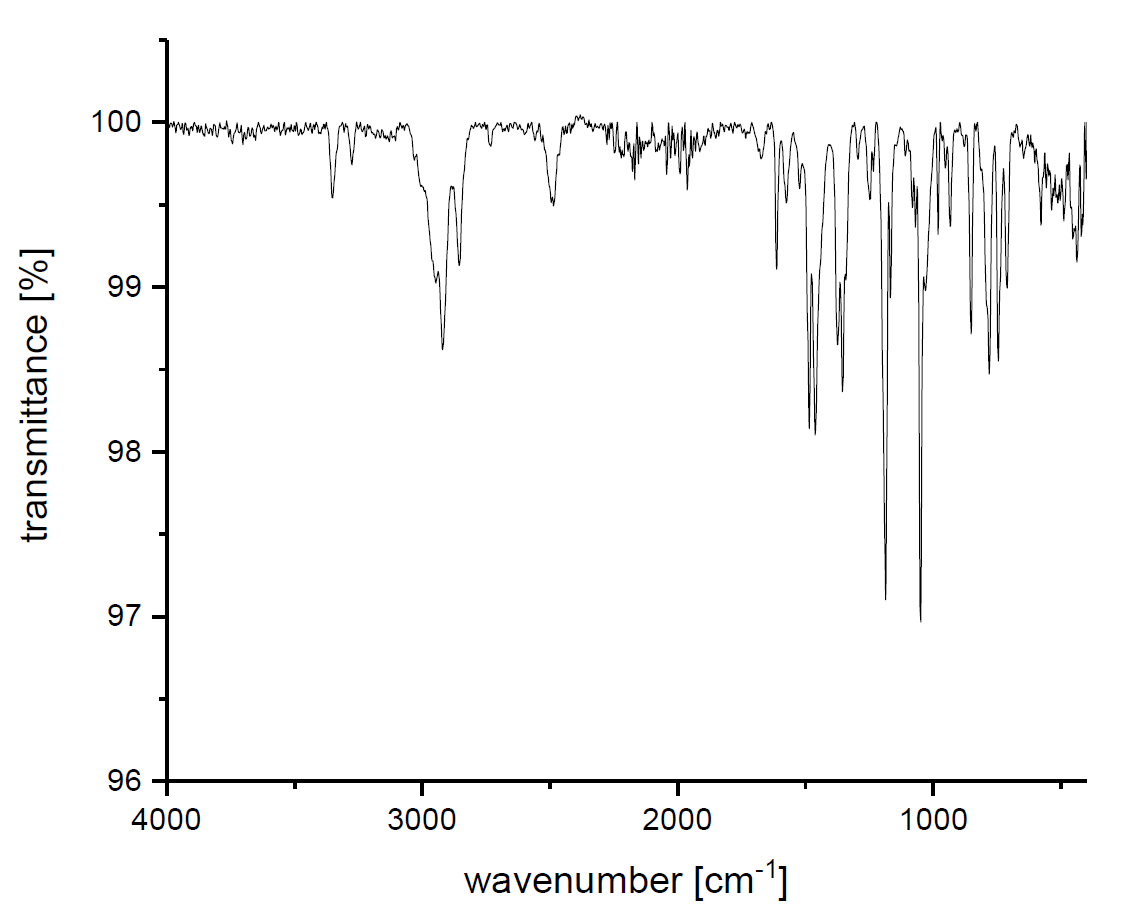


Figure S10. ATR-IR spectra (solid) of [Tp^Mes*^FeSeCysAm] 4b.


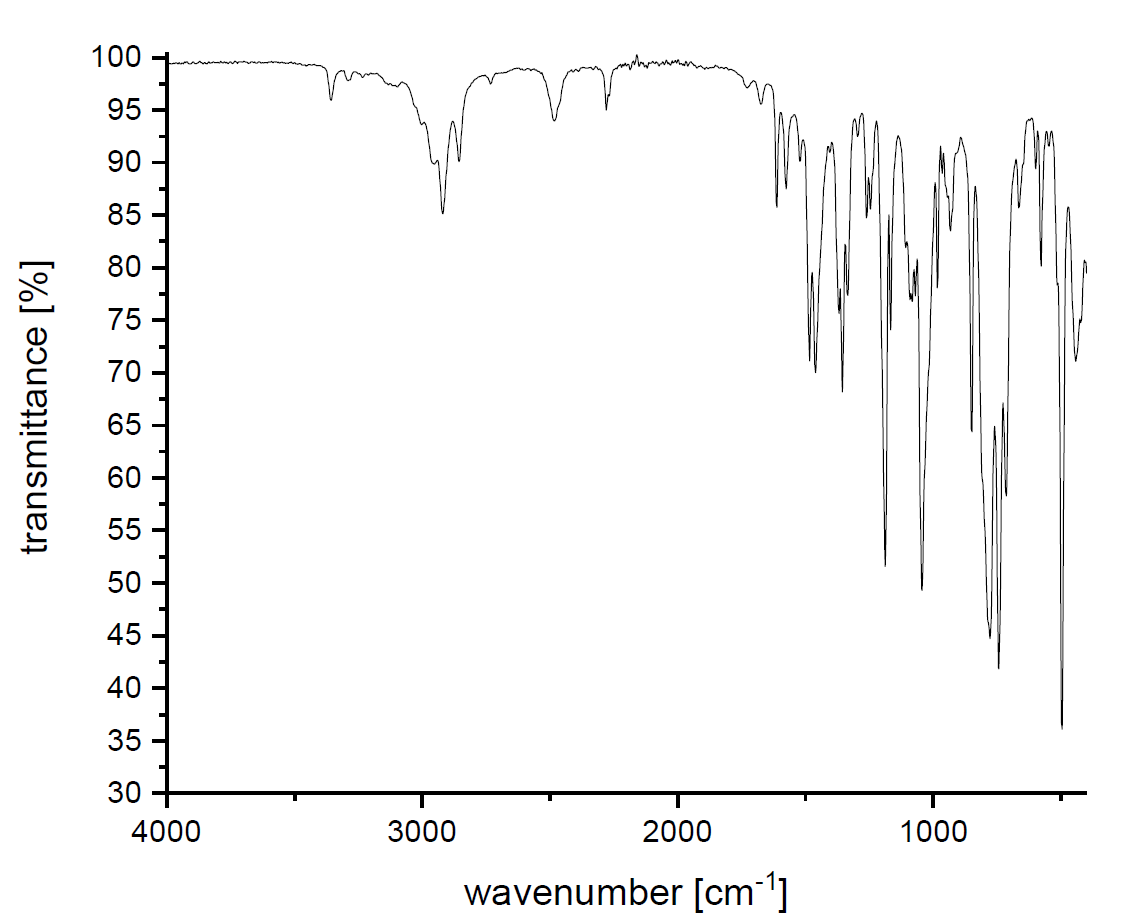


Figure S11. ATR-IR spectra (solid) of [Tp^Mes*^FeO_2_SeCysAm] 5b.


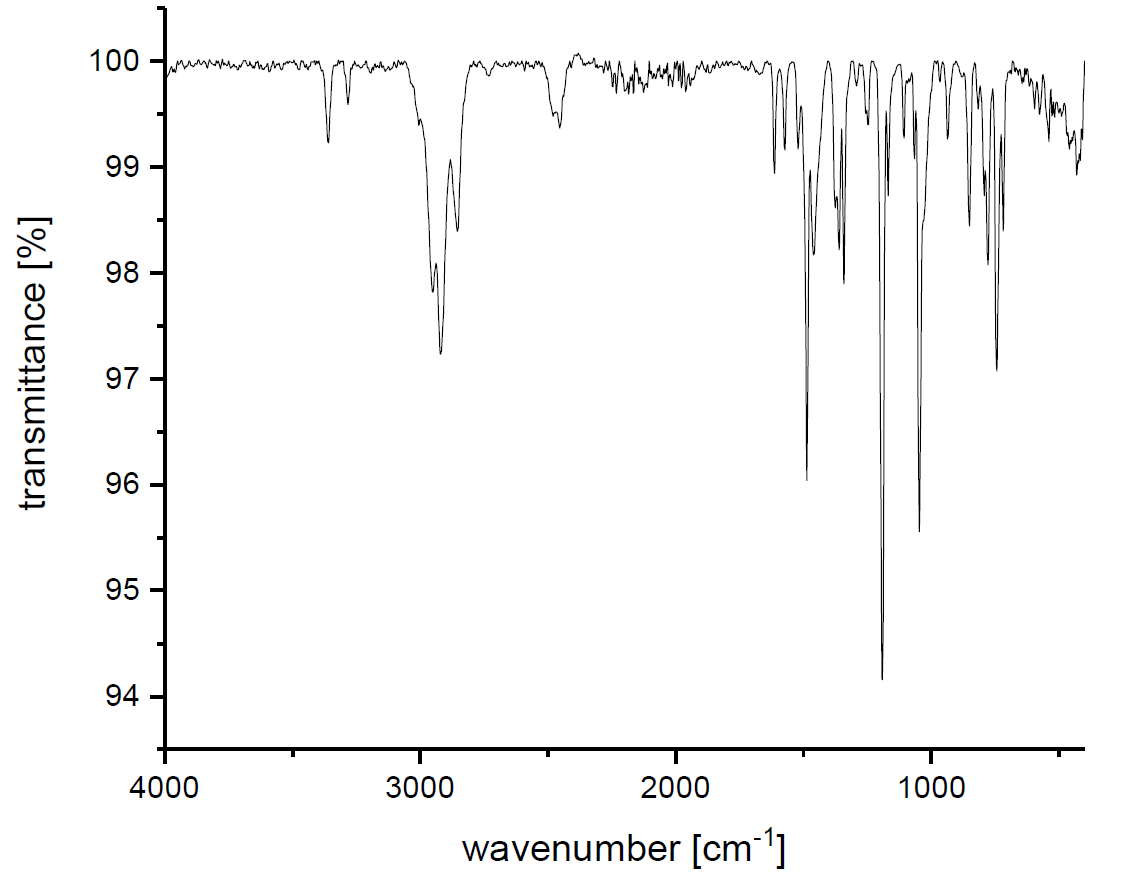


Figure S12. ATR-IR spectra (solid) of [Tp^Mes^CoSeCysAm] 7.


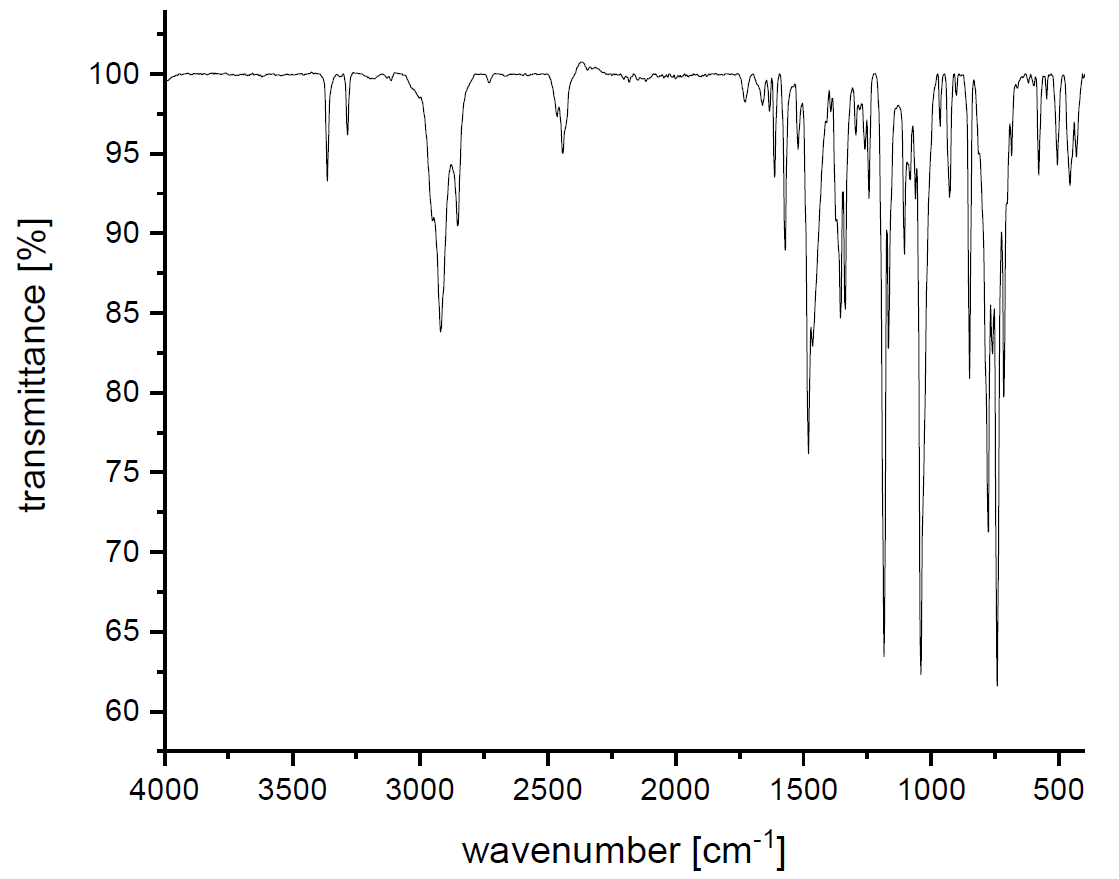


Figure S13. ATR-IR spectra (solid) of [Tp^Mes^CoO_2_SeCysAm] 8.

**ESI-MS spectrometry**


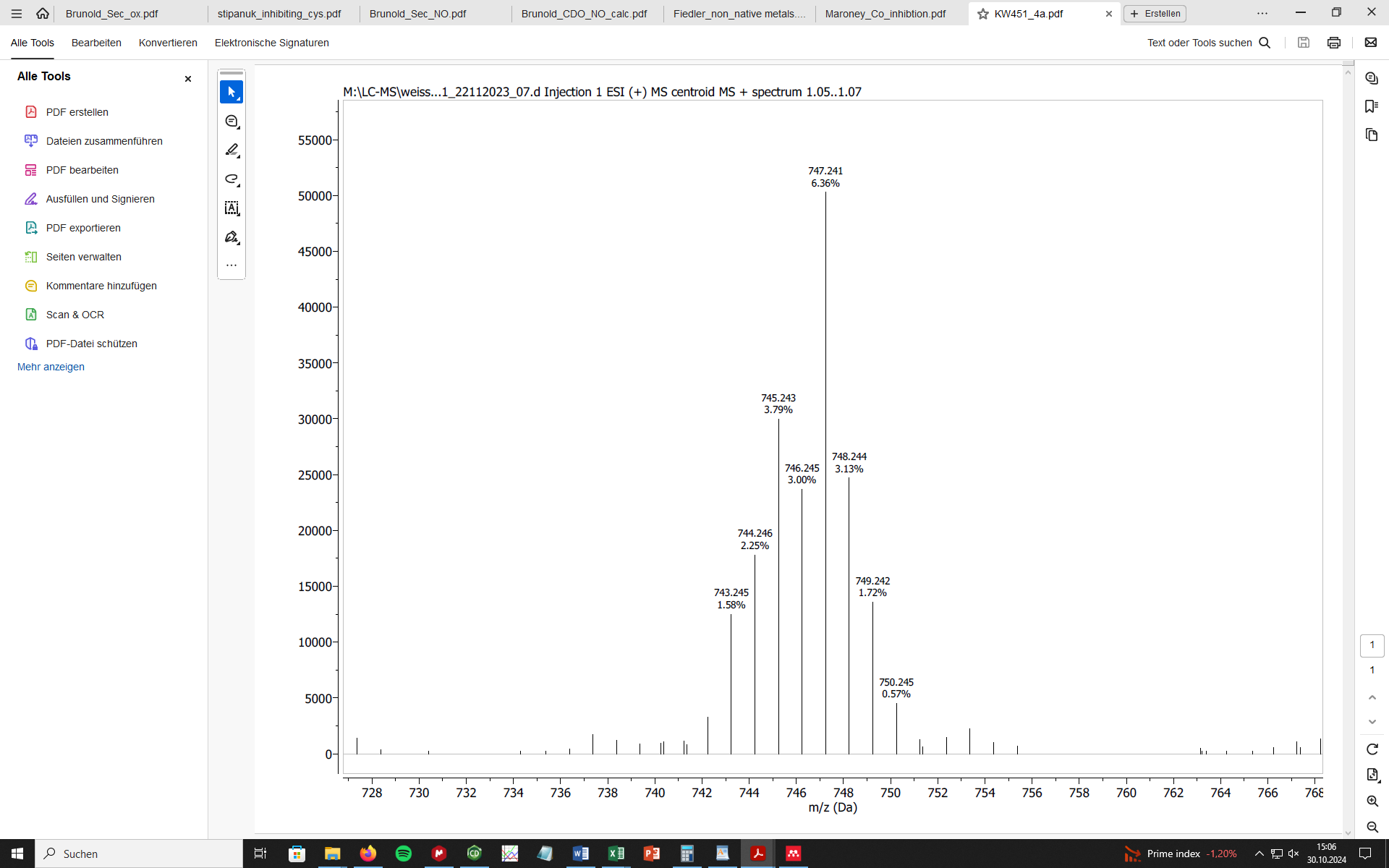


Figure S14. Section of the ESI-MS spectrum of [Tp^Mes^FeSeCysAm] 4a, showing the isotopic pattern attributed to [C_38_H_46_BFeN_7_Se]^+^.


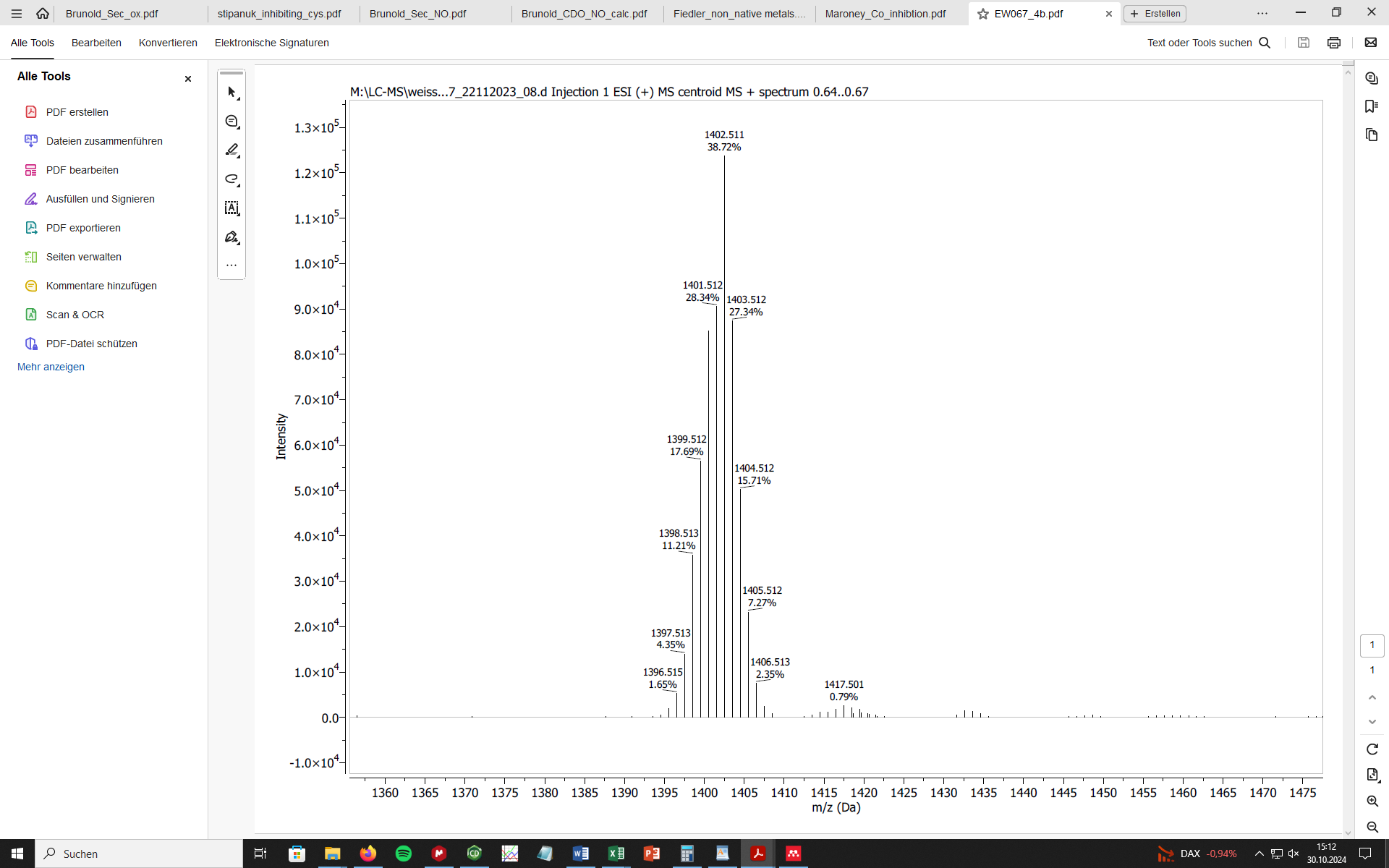


Figure S15. Section of the ESI-MS spectrum of [Tp^Mes*^FeSeCysAm] 4b, showing the isotopic pattern attributed to [C_38_H_46_BFeN_7_Se + C_36_H_40_BFeN_6_ + MeOH]^+^.


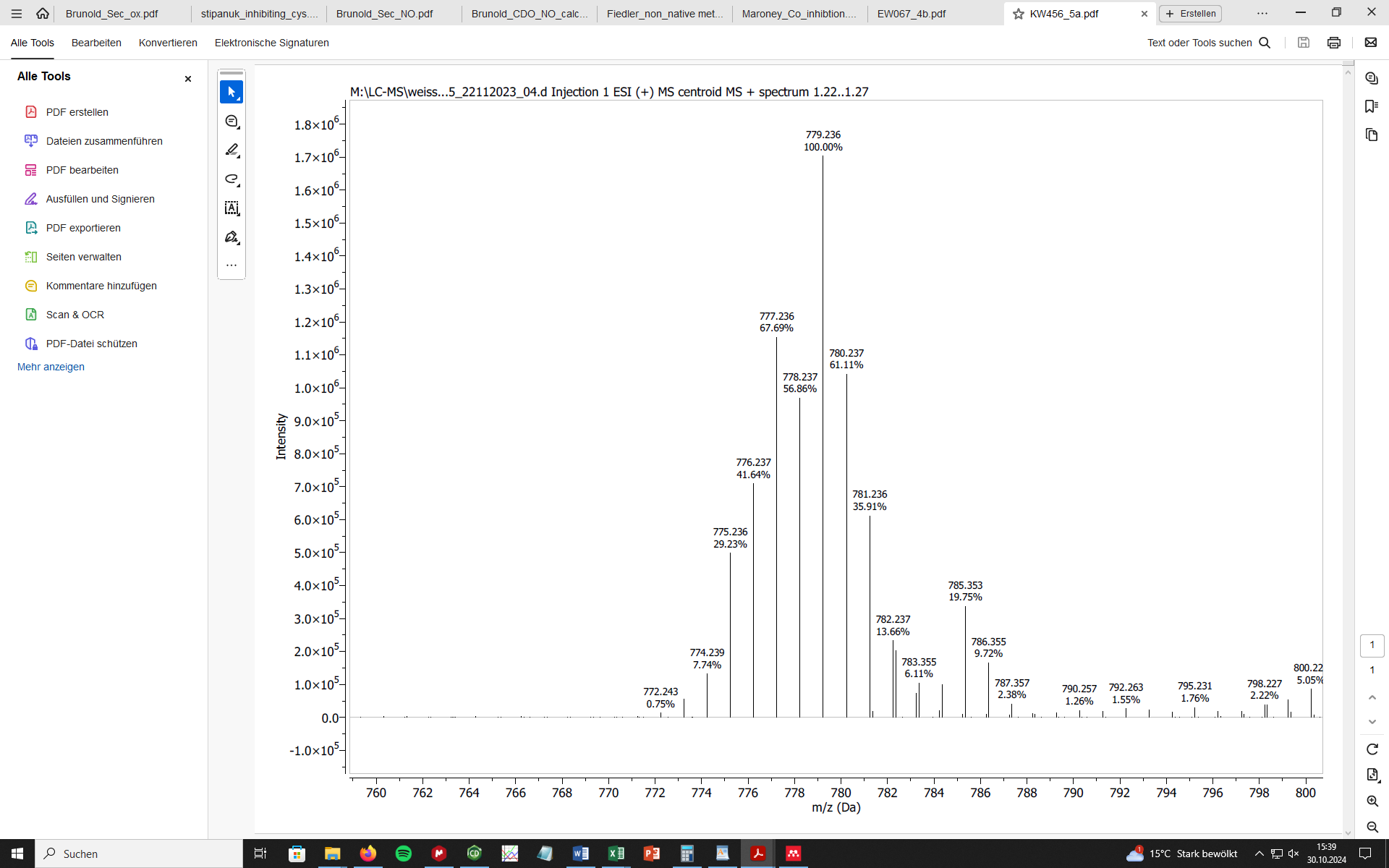


Figure S16. Section of the ESI-MS spectrum of [Tp^Mes^FeO_2_SeCysAm] 5a, showing the isotopic pattern attributed to [C_38_H_46_BFeN_7_O_2_Se]^+^.


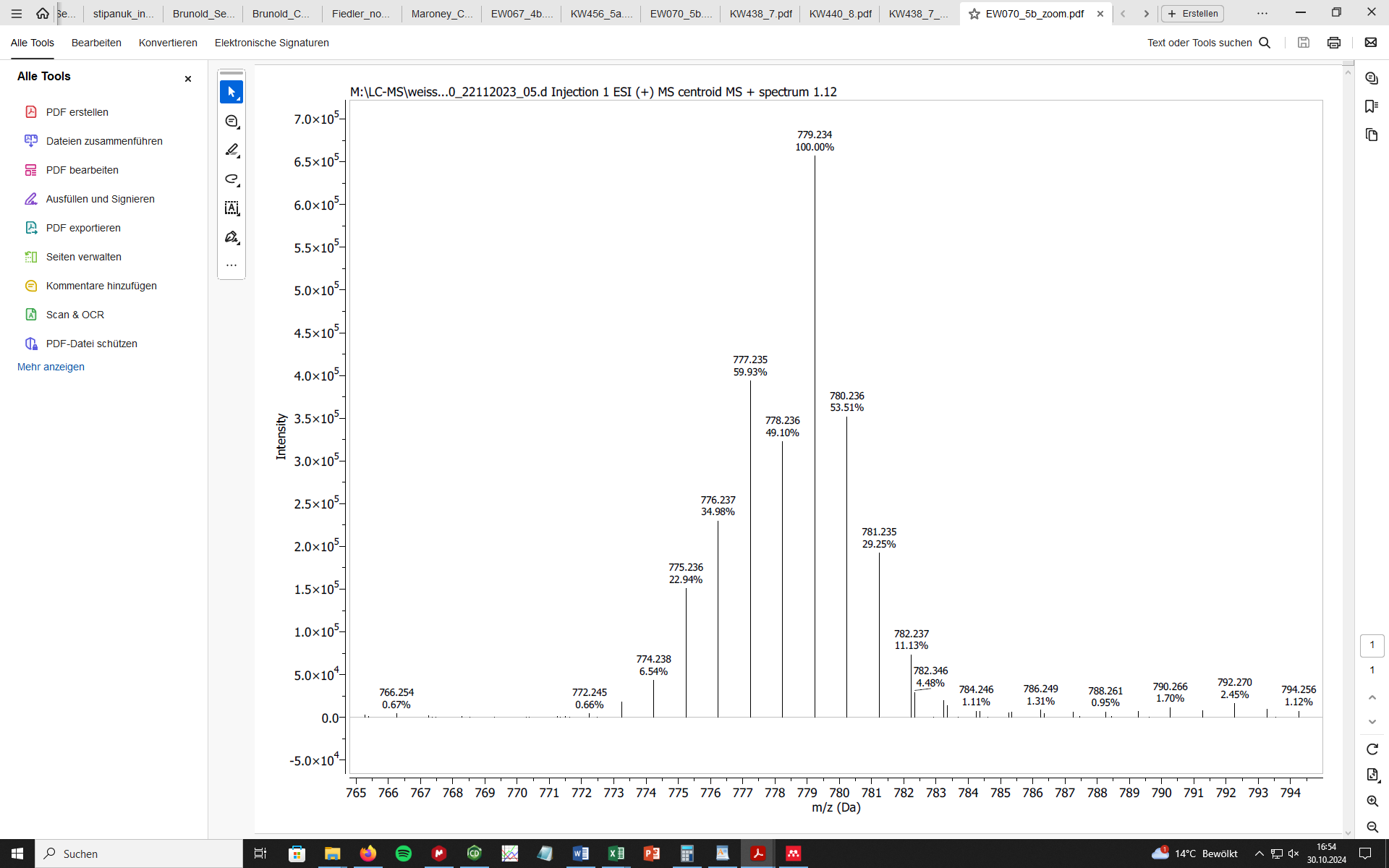


Figure S17. Section of the ESI-MS spectrum of [Tp^Mes*^FeO_2_SeCysAm] 5b, showing the isotopic pattern attributed to [C_38_H_46_BFeN_7_O_2_Se]^+^.


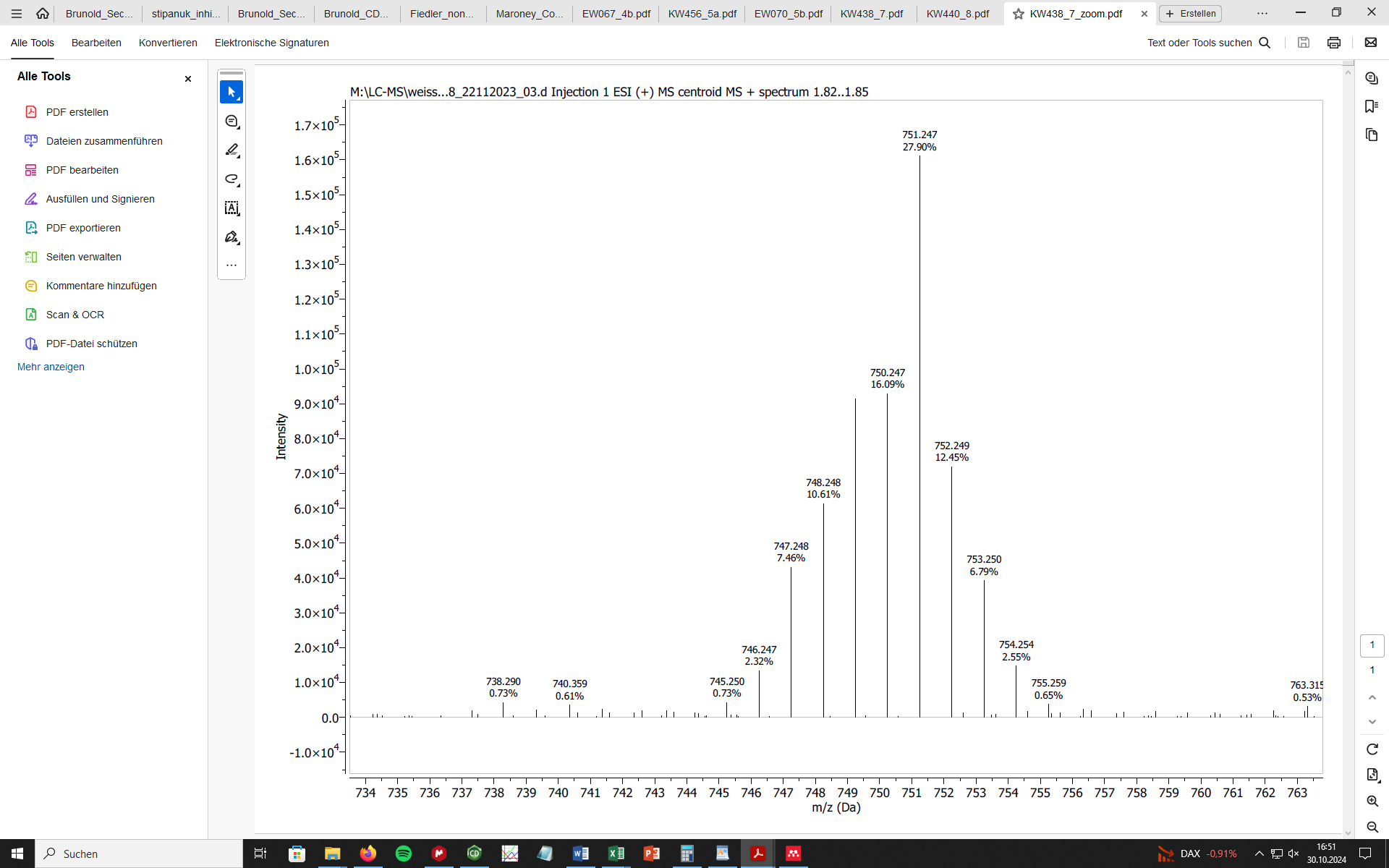


Figure S18. Section of the ESI-MS spectrum of [Tp^Mes^CoSeCysAm] 7, showing the isotopic pattern attributed to [C_38_H_46_BCoN_7_Se]^+^.


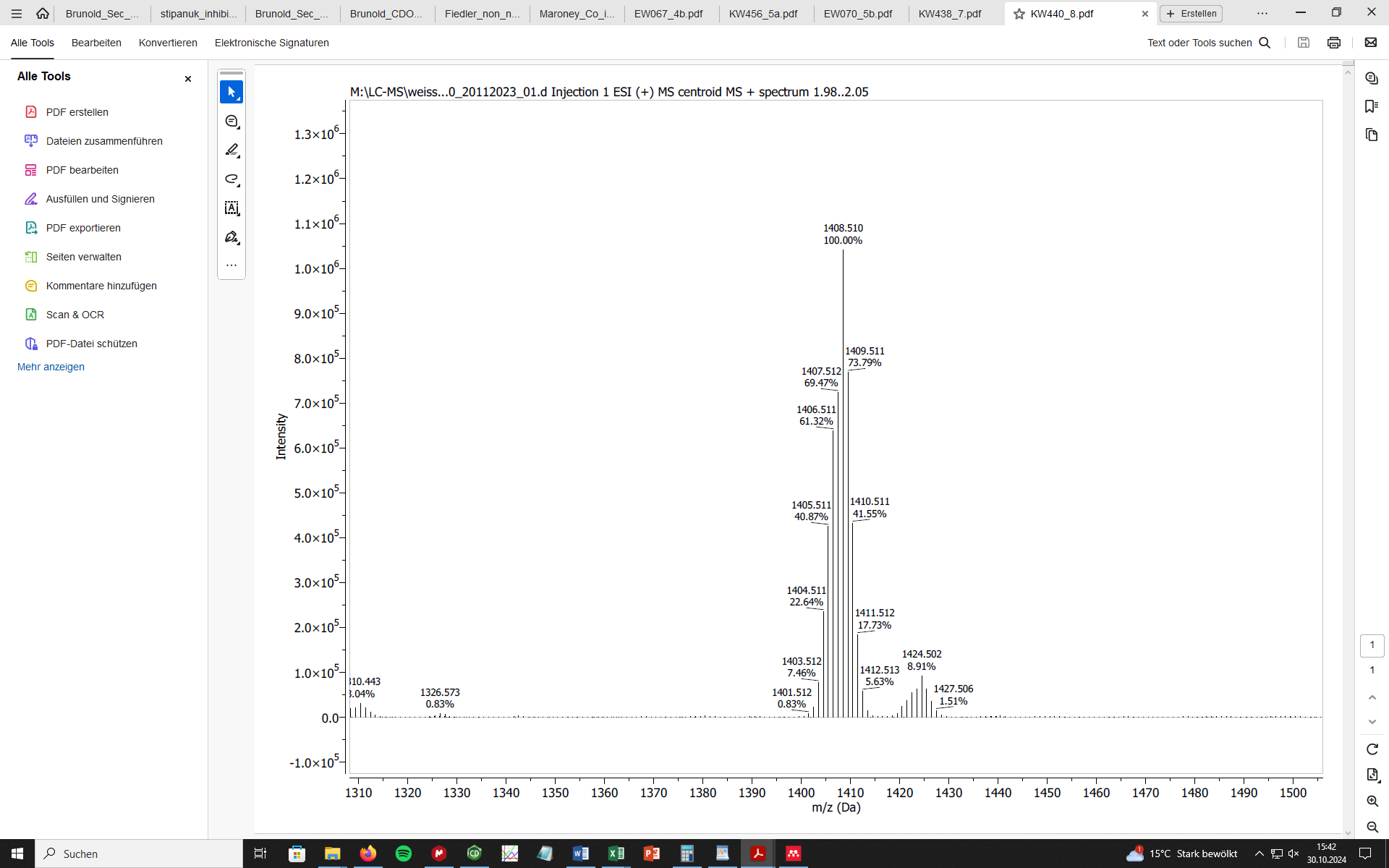


Figure S19. Section of the ESI-MS spectrum of [Tp^Mes^CoO_2_SeCysAm] 8, showing the isotopic pattern attributed to [C_38_H_46_BCoN_7_O_2_Se + C_36_H_40_BCoN_6_]^+^.

**UV/Vis spectroscopy**


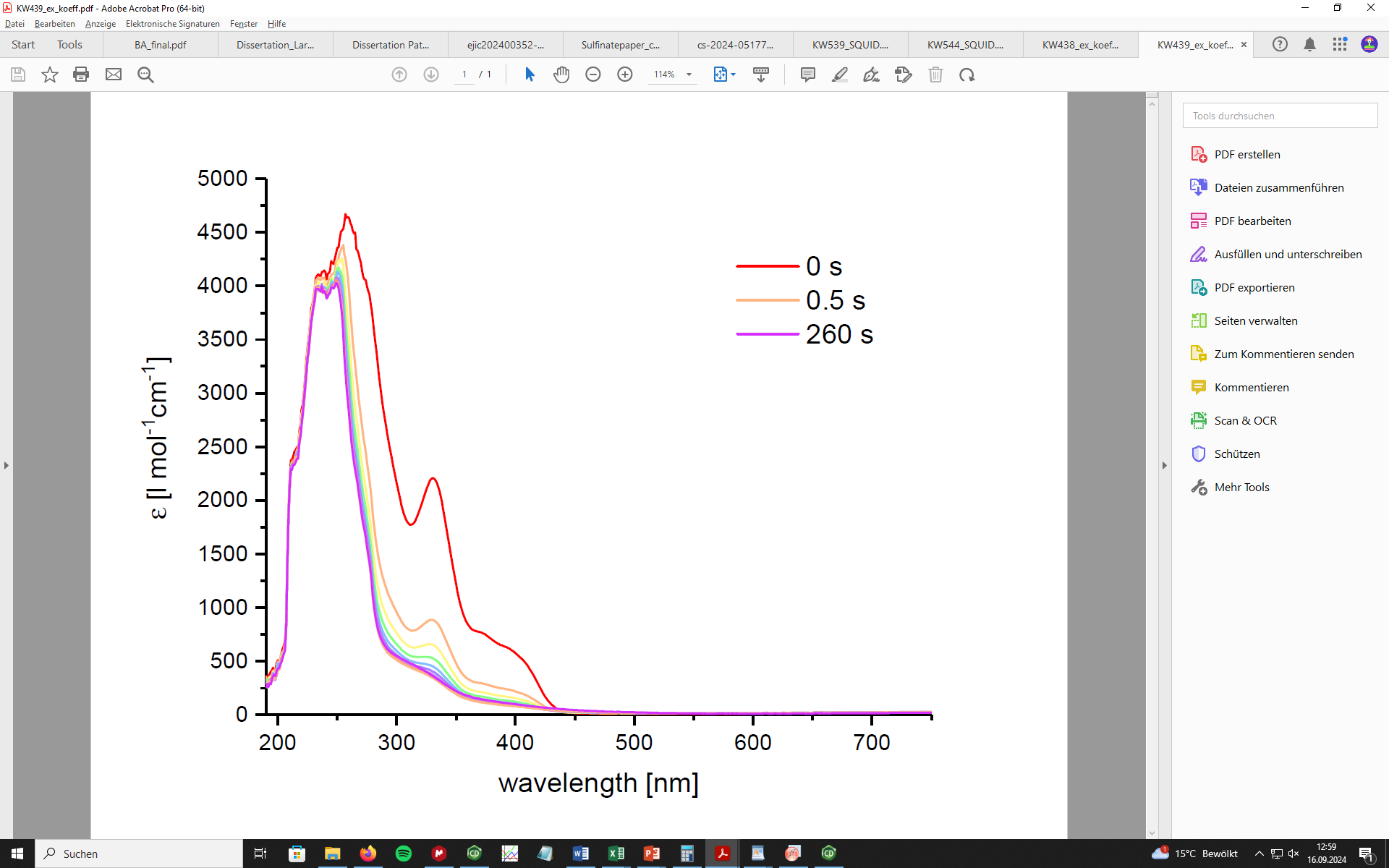


Figure S20. Monitoring the reaction of [Tp^Mes^FeSeCysAm] 4a (THF, 0.66 mM, 0 °C) with O_2_ by UV/Vis spectroscopy.


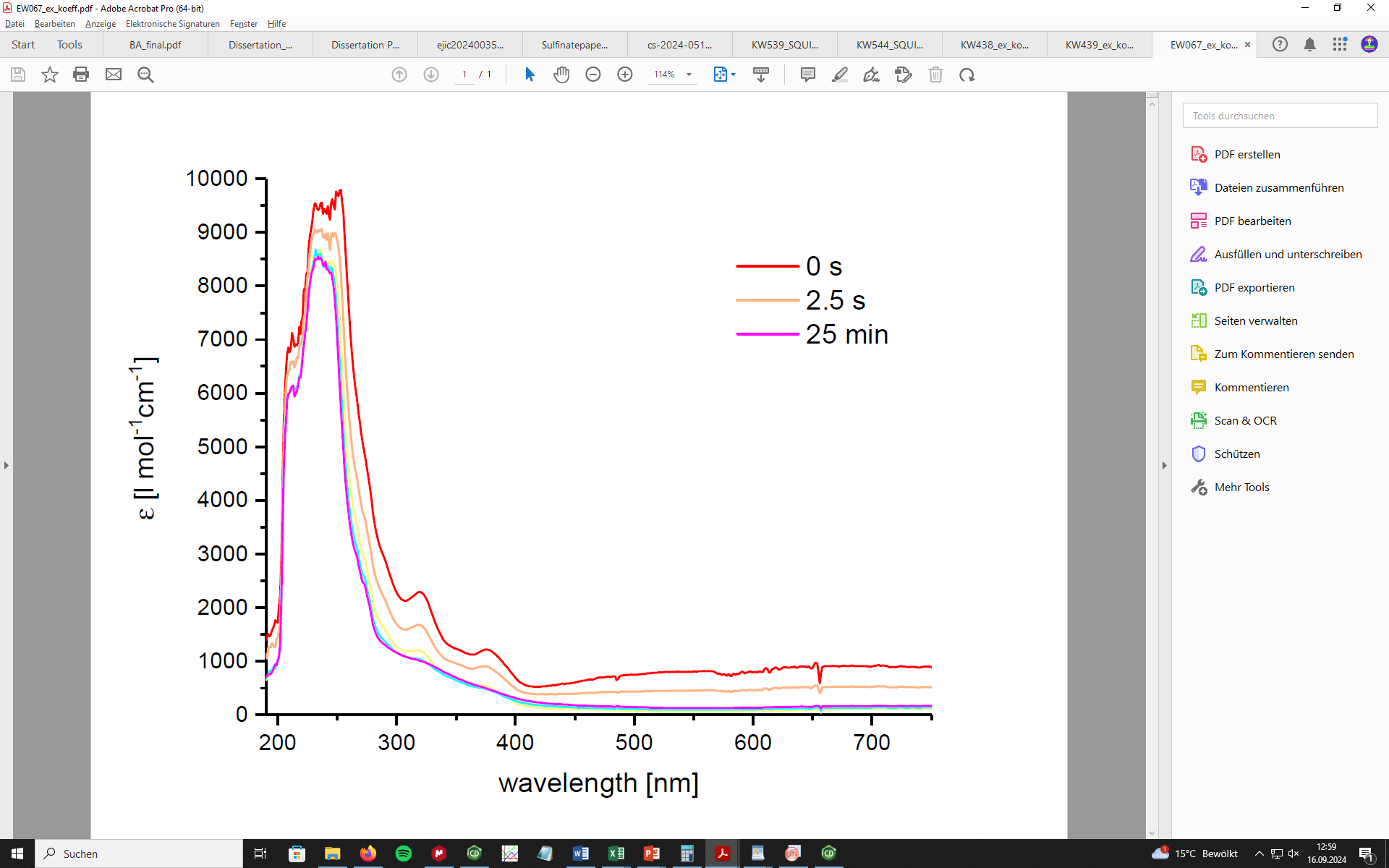


Figure S21. Monitoring the reaction of [Tp^Mes*^FeSeCysAm] 4b (THF, 0.33 mM, -80 °C) with O_2_ by UV/Vis spectroscopy.


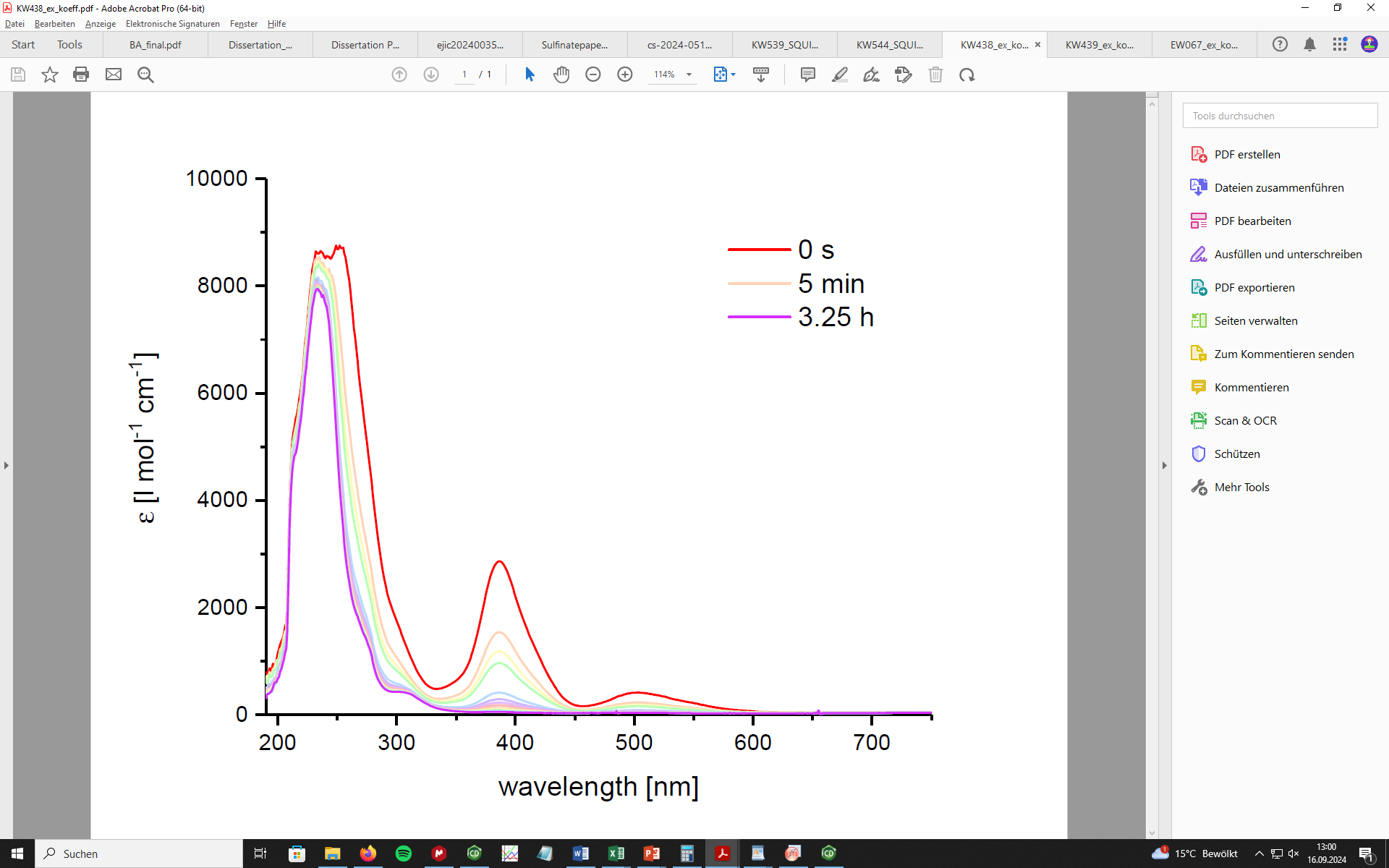


Figure S22. Monitoring the reaction of [Tp^Mes^CoSeCysAm] 7 (THF, 0.33 mM, 40 °C) with O_2_ with UV/Vis spectroscopy.

**Kinetic investigations**

All solutions were prepared freshly prior to measurement. In order to achieve the desired concentration, 1 mL of the dioxygen-saturated solvent was added to a 2 mL solution of the investigated complex. The oxygen-saturated solution was prepared by purging the respective solvent with dry oxygen for a period of 10 minutes. In order to guarantee a consistent oxygen supply, an O_2_-filled balloon was connected to the cuvette *via* a septum. The data were collected following the addition of the oxygen-saturated solution.

In the course of the kinetic investigations, the decay of the selenium-metal charge-transfer band was monitored. The observed time window corresponds to two half-lives, which were calculated by employing an exponential fit of the form $f\left( t \right)=y_{0}+A_{0}e^{-R_{0}t}$ to the measured absorbance, plotted against time. The first and second order plots were fitted by linear functions of the form $f\left( t \right)=a+kt$ with $\left| k \right|$ denoting the observed rate constant.

*[Tp^Mes^FeSeCysAm] (****4a****)*

The kinetic investigations for [Tp^Mes^FeSeCysAm] (**4a**) were carried out in THF. The Ostwald coefficient for dioxygen in THF is 0.2467^[4]^, therefore an excess of 11 equivalents of oxygen is employed when 1 mL of saturated solution is added to a 0.66 mM solution of **4a** in THF. The reaction follows second order kinetics with $k^{0^{\circ}C}$ = 6.01∙10^-2^ L∙mol^-1^∙s^-1^. A second order reaction rate under pseudo first order conditions can have various reasons, depending, for instance, on the barrier height of the superoxide formation.^[5]^


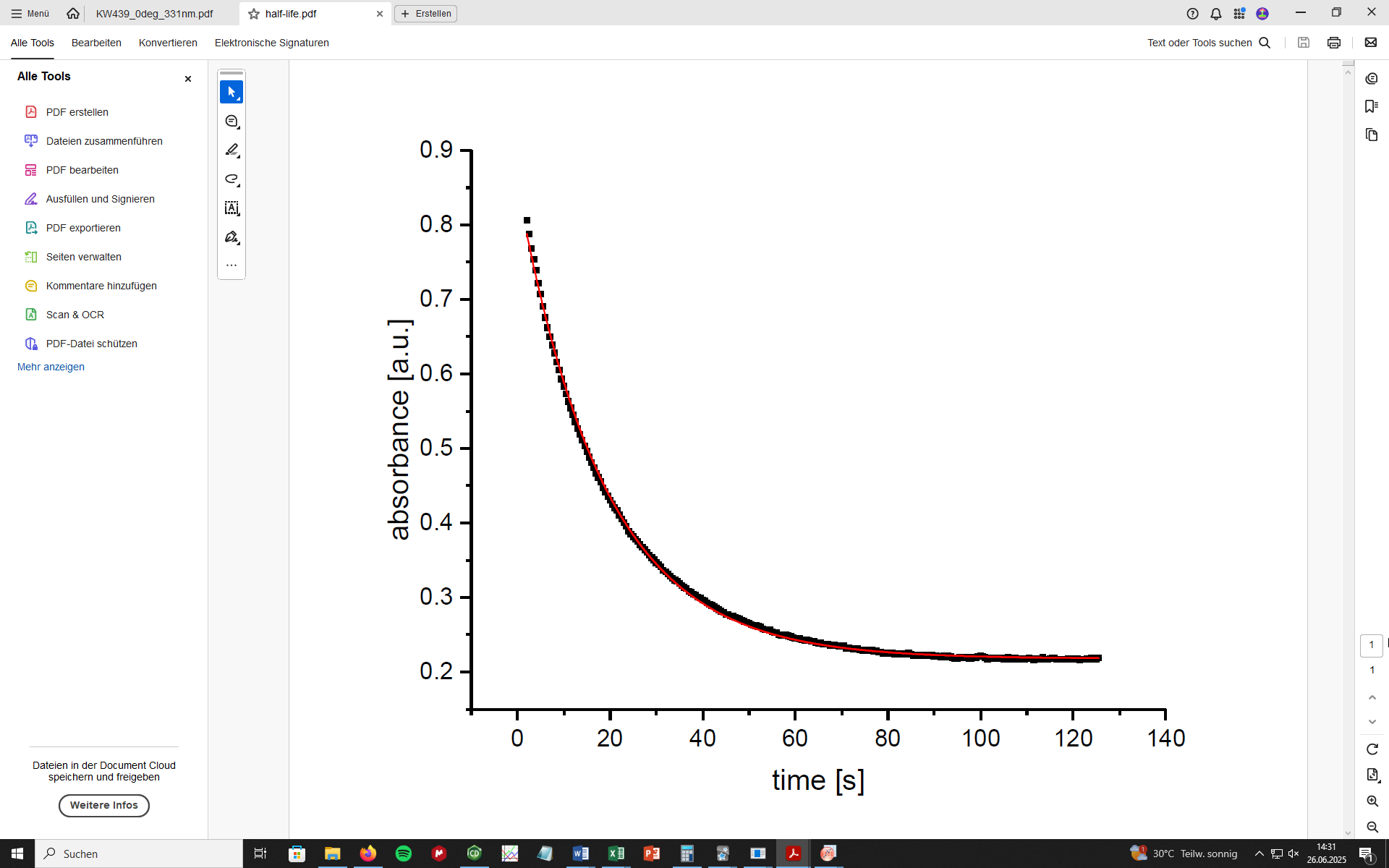


Figure S23. Trace of the relative absorption at 331 nm in the UV-Vis-spectra of [Tp^Mes^FeSeCysAm] (4a) in THF (0.66 mM) after the addition of 1 mL oxygen saturated THF at 0 °C. The red line represents an exponential fit for the determination of the half-life of 4a in the presence of O_2_.

The regression of the data using an exponential function results in the following equation: $f\left( t \right)=0,63464\times e^{-0.054 \frac{1}{s} \times t}+0,21794$ .

The half-life of the decay is determined *via* $t_{1/2}=\frac{\ln\left( 2 \right)}{0.054} s=12.83 s\approx13 s$.

The half-life time that can be deduced based on the rate constant determined would be 19 s.


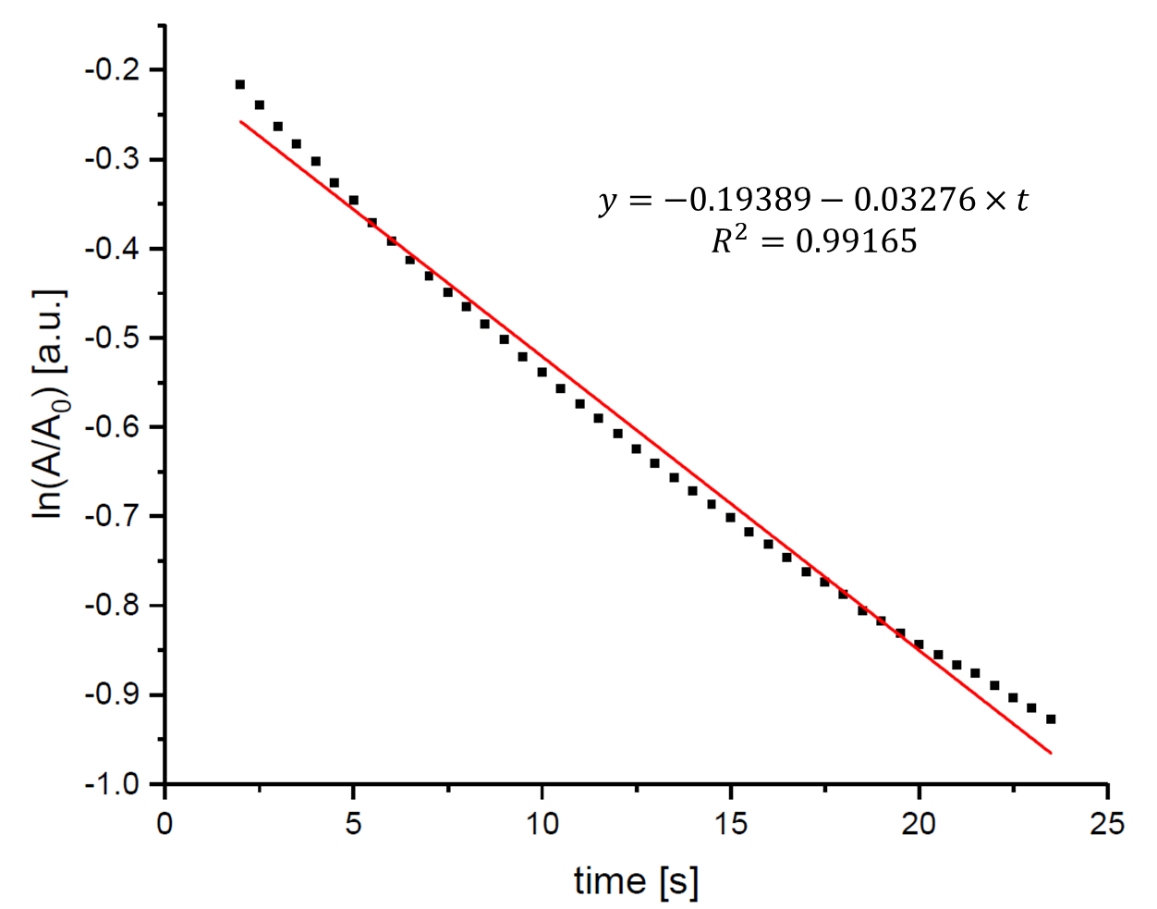


Figure S24. Plot of the natural logarithm of the relative absorption at 331 nm against the time (first order plot) in the UV-Vis-spectra of [Tp^Mes^FeSeCysAm] (4a) in THF (0.66 mM) after the addition of 1 mL oxygen saturated THF at 0 °C with linear regression (red).


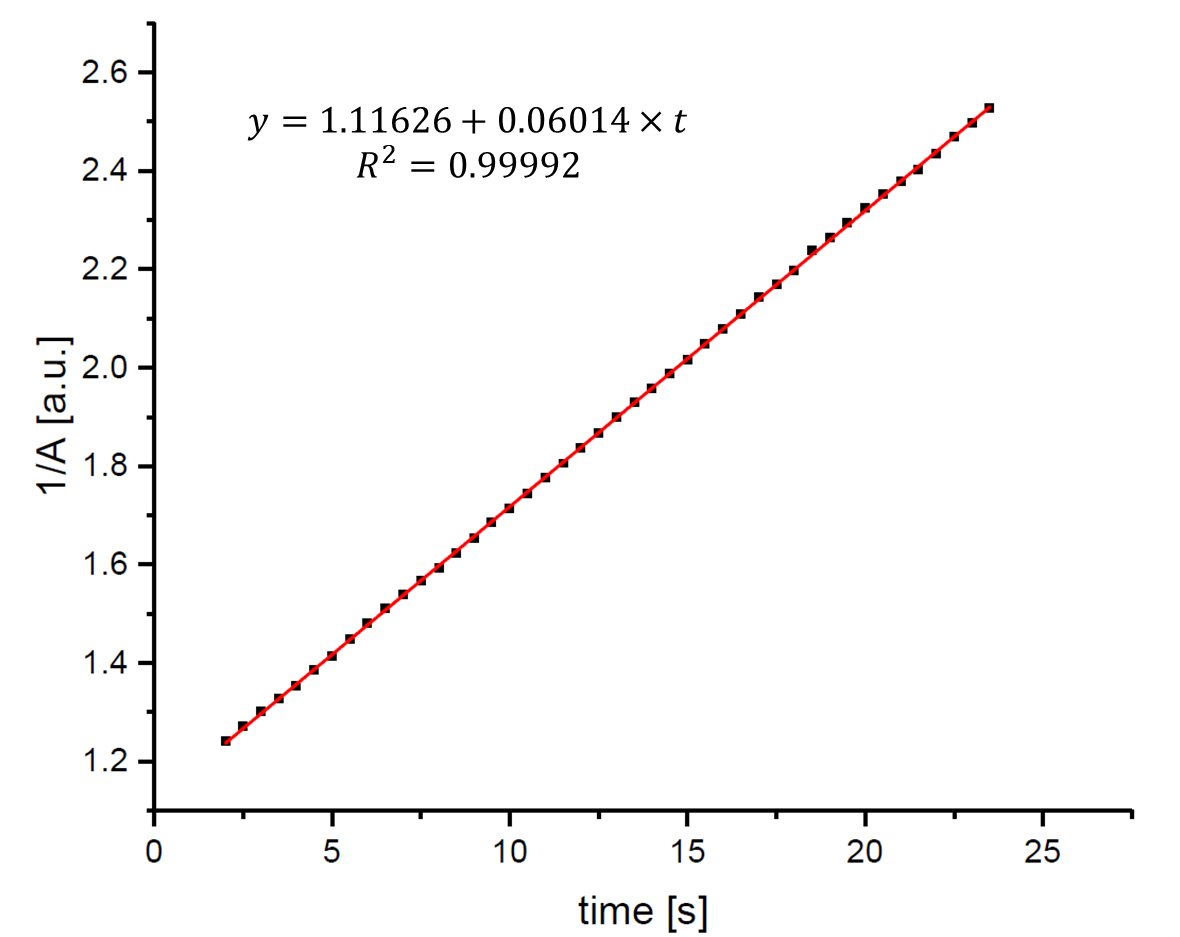


Figure S25. Plot of the reciprocal relative absorption at 331 nm against the time (second order plot) in the UV-Vis-spectra of [Tp^Mes^FeSeCysAm] (4a) in THF (0.66 mM) after the addition of 1 mL oxygen saturated THF at 0 °C with linear regression (red).

*[Tp^Mes^CoSeCysAm] (****7****)*

The kinetic investigations of complex **7** were carried out in MeCN. The solubility of dioxygen in MeCN at 24 °C is 4.4∙10^-4^ $x$_1_ ^[4]^, therefore an excess of 13 equivalents of oxygen is employed when 1 mL of saturated solution is added to a 0.2 mM solution of **7** in MeCN. As illustrated in Figure S27 and Figure S28, the reaction order is dependent on temperature, which is typical for such kind of reactions (changes in entropic contributions, O_2_ solubilities, barriers). For temperatures exceeding 0 °C, the kinetics are classified as pseudo first order, given that the oxygen concentration is sufficiently high to be regarded as constant.


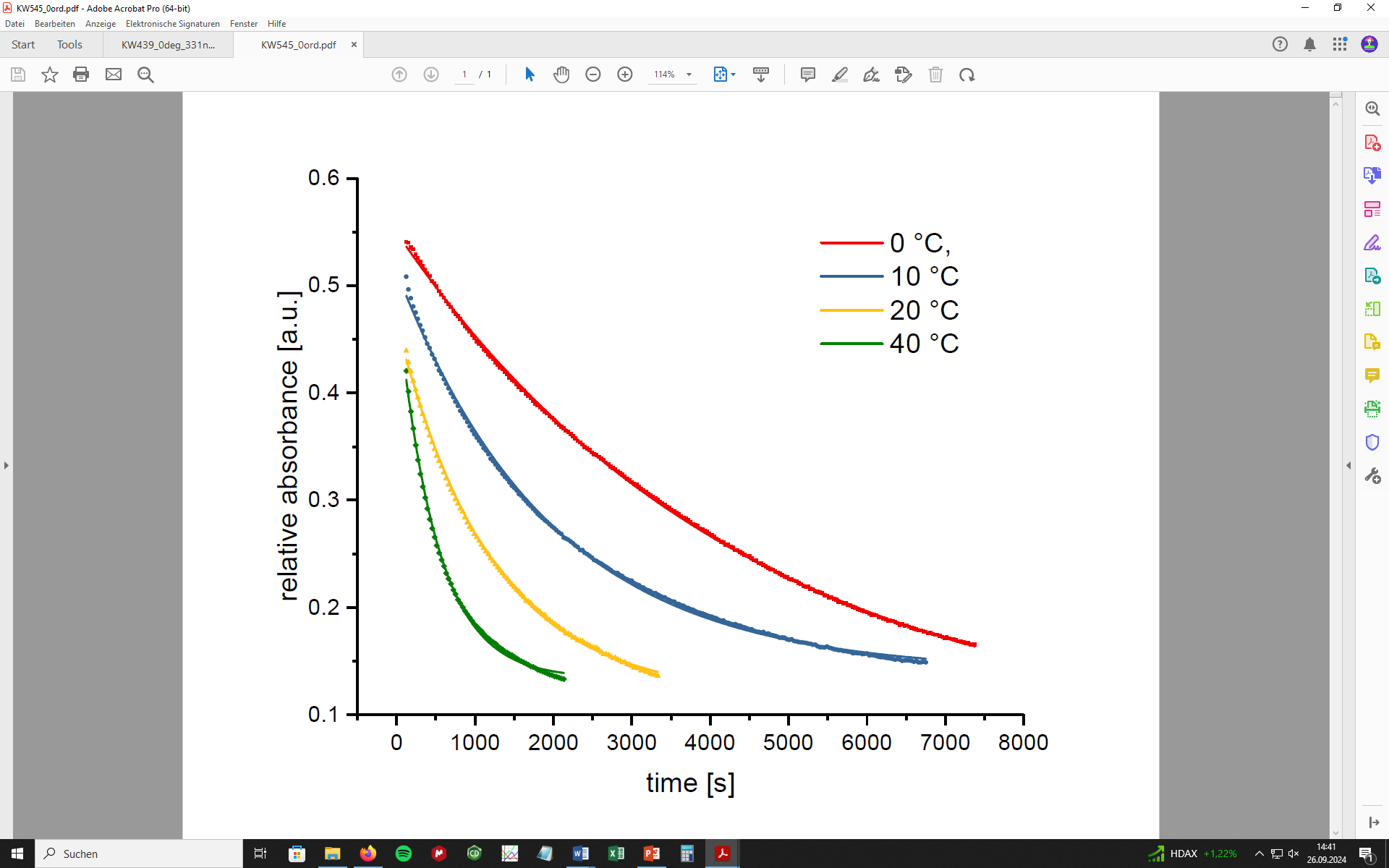


Figure S26. Trace of the relative absorption at 378 nm in the UV-Vis-spectra of [Tp^Mes^CoSeCysAm] (7) in MeCN (0.2 mM) after the addition of 1 mL of dioxygen-saturated MeCN at different temperatures with exponential regression.

The regression of the data was carried out using an exponential function of the form:

$f\left( t \right)=A_{0}\times e^{-k \times t}+y_{0}$ . The half-life is calculated *via* $t_{1/2}=\frac{\ln\left( 2 \right)}{k}$.

Table S1. Results of the regression of the decays shown in Figure S26, with the time constants and the calculated half-life.

|  | *k* [1/s] | *t_1/2_* [s] |
| --- | --- | --- |
| 0 °C | 2,25837·10^-4^ | 3068 |
| 10°C | 5.07722·10^-4^ | 1367 |
| 20 °C | 8.31402·10^-4^ | 833 |
| 40 °C | 2.01·10^-3^ | 344 |


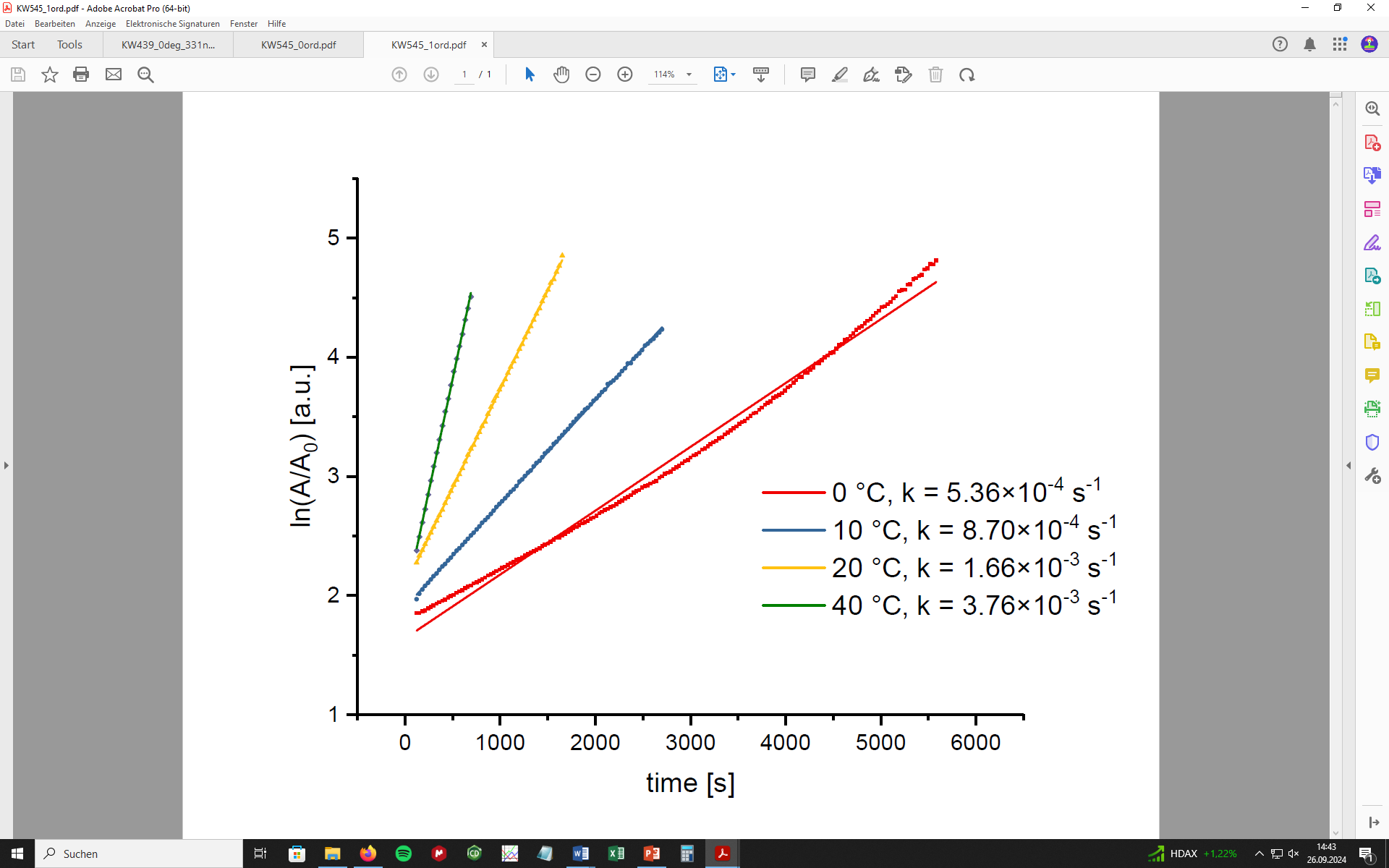


Figure S27. Plot of the natural logarithm of the relative absorption at 378 nm against the time (first order plot) in the UV-Vis-spectra of [Tp^Mes^CoSeCysAm] (7) in MeCN (0.2 mM) after the addition of 1 mL oxygen saturated MeCN at different temperatures with linear regression and the resulting rate constants.


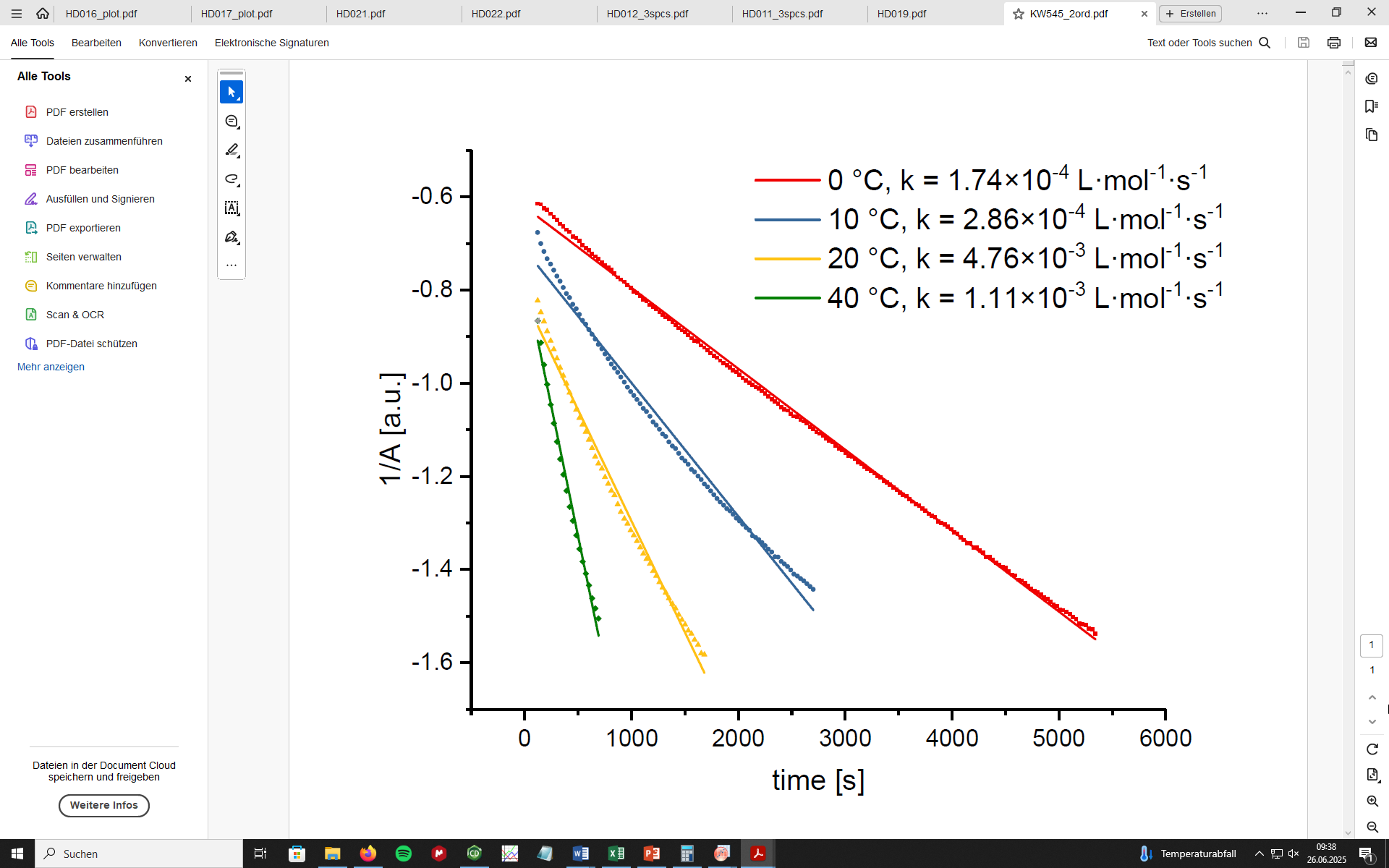


Figure S28. Plot of the reciprocal relative absorption at 378 nm against the time (second order plot) in the UV-Vis-spectra of [Tp^Mes^CoSeCysAm] (7) in MeCN (0.2 mM) after the addition of 1 mL oxygen saturated MeCN at different temperatures with linear regression and the resulting rate constants.

To unambiguously determine the reaction order for the reaction of Tp^Mes^CoSeCysAm with an excess of dioxygen, the **method of initial rates** was applied. The experiments were conducted in THF, varying the concentration of the complex (0.05 mmol/L, 0.1 mmol/L, and 0.2 mmol/L) while maintaining a constant oxygen concentration (using an O₂-saturated solution in THF). The reaction rate was monitored during the first 50 seconds after adding the O₂-saturated solution at 40 °C. The **initial rate (*v₀*)** was determined by applying a tangent at the beginning of the concentration *versus* time plot, where the slope of the linear section corresponds to *v₀*. Plotting the logarithm of the initial rates (log(*v₀*)) against the logarithm of the corresponding initial complex concentrations (log(*A₀*)) yields a linear relationship, where the **slope (*m*) represents the reaction order.**

The results of these studies, shown in Figure S29 and Figure S30, indicate a reaction order of 0.9 with respect to the complex, in agreement with the linear correlation observed for the ln(*A*/*A_0_*) *versus t* plots in Figure S27.


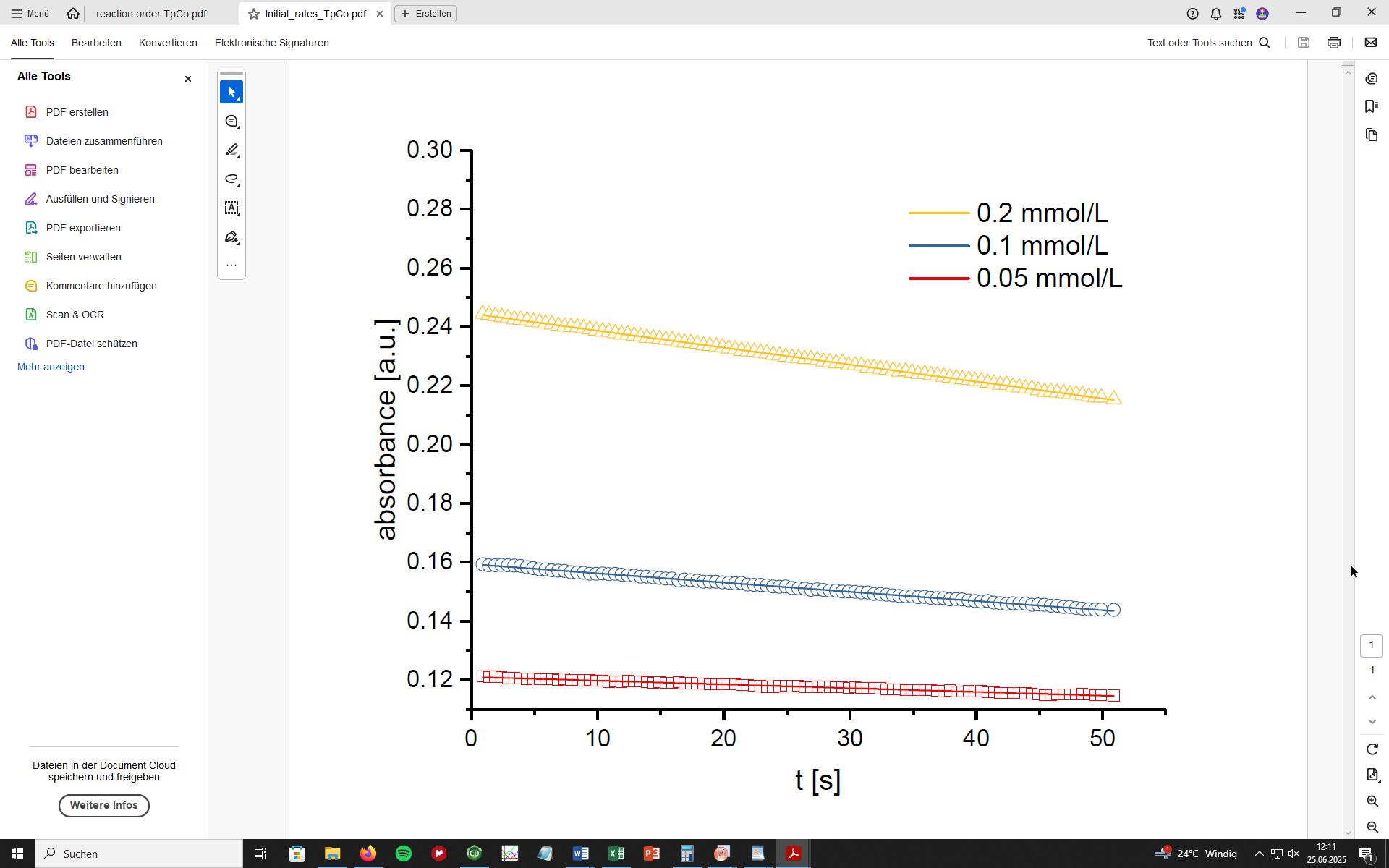


Figure S29. Trace of the relative absorption at 387 nm in the UV-Vis-spectra of [Tp^Mes^CoSeCysAm] (7) in THF with varying concentrations of the complex, after the addition of 1 mL oxygen saturated THF at 40 °C with linear regression.

Rate law with respect to complex **7**: $v=k{[A]}^{m}$

Logarithmic representation: $\log\left( v \right)=\log\left( k \right)+m\times log(A)$


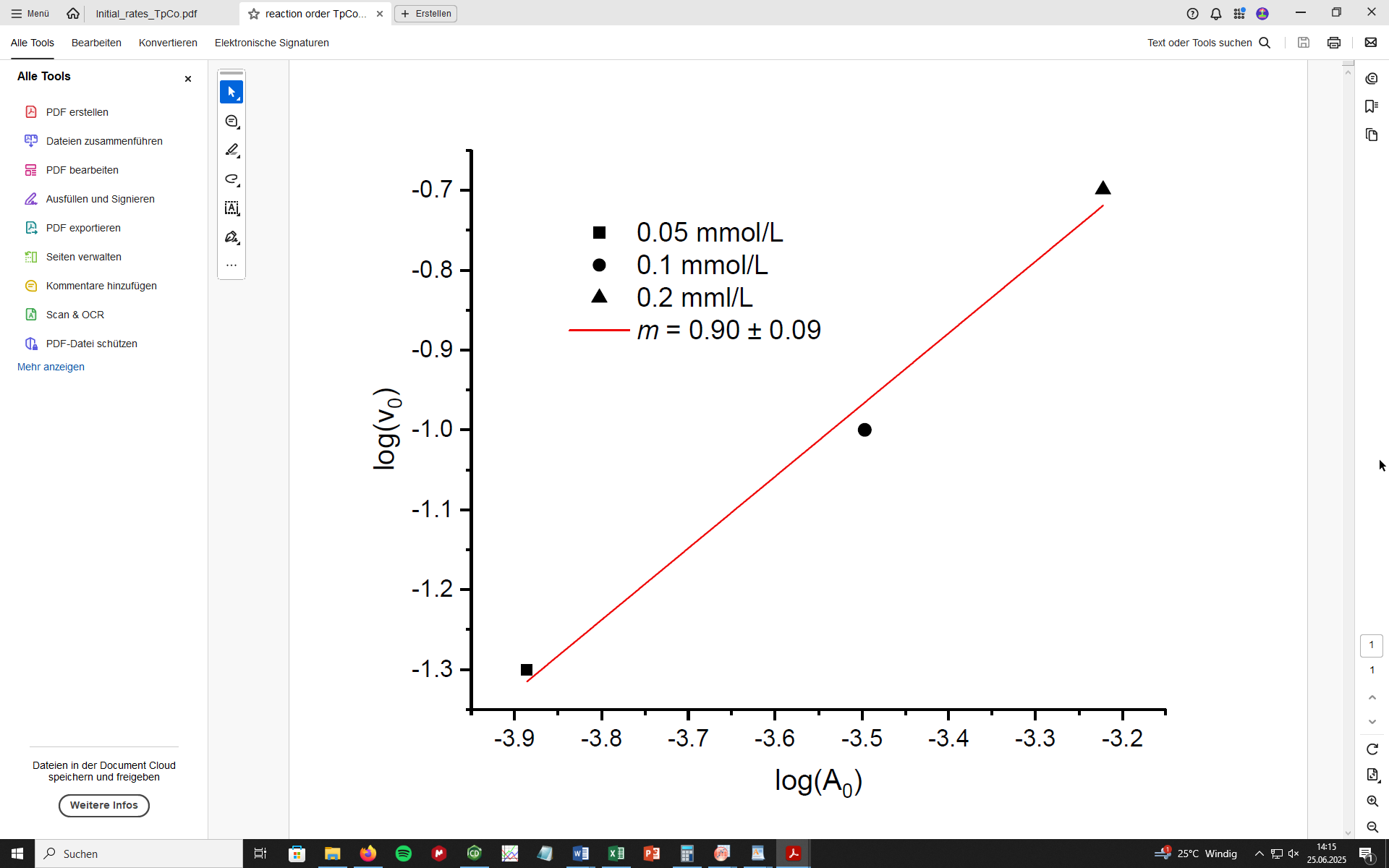


Figure S30. Logarithmic plot of the initial rates *v_0_* derived from Figure S29 against the starting concentration *A_0_* (black) with linear regression (red).

**Magnetic susceptibility measurements**

Magnetic measurements were performed with a QuantumDesign MPMS3 SQUID magnetometer. The samples were prepared in a glovebox in VSM powder capsules which were sealed with a piece of Teflon tape. Both the capsules and the Teflon tape were dried in a Schlenk flask under vacuum at 110 °C for five days. A brass sample holder was used. The measurement was carried out in VSM mode from 300 K to 2 K in a magnetic field of 7 T. A Background correction was applied subtracting the magnetic moment of an empty capsule sealed with a piece of Teflon tape using the same measurement sequence as for the sample. A diamagnetic correction was performed using Pascal’s constants.^[6]^ The effective magnetic moments of the compounds are 4.57 µ_B_ (**7**) and 4.80 µ_B_ (**8**) respectively, which is above the expected spin-only value of 3.88 µ_B_ for both complexes. This can be attributed to orbital contributions to the magnetic moment, derived from the orbital mixing (spin–orbit coupling constant λ = −170 cm^–1^ for Co^2+^), which are neglected in the spin-only formula.^[7]^


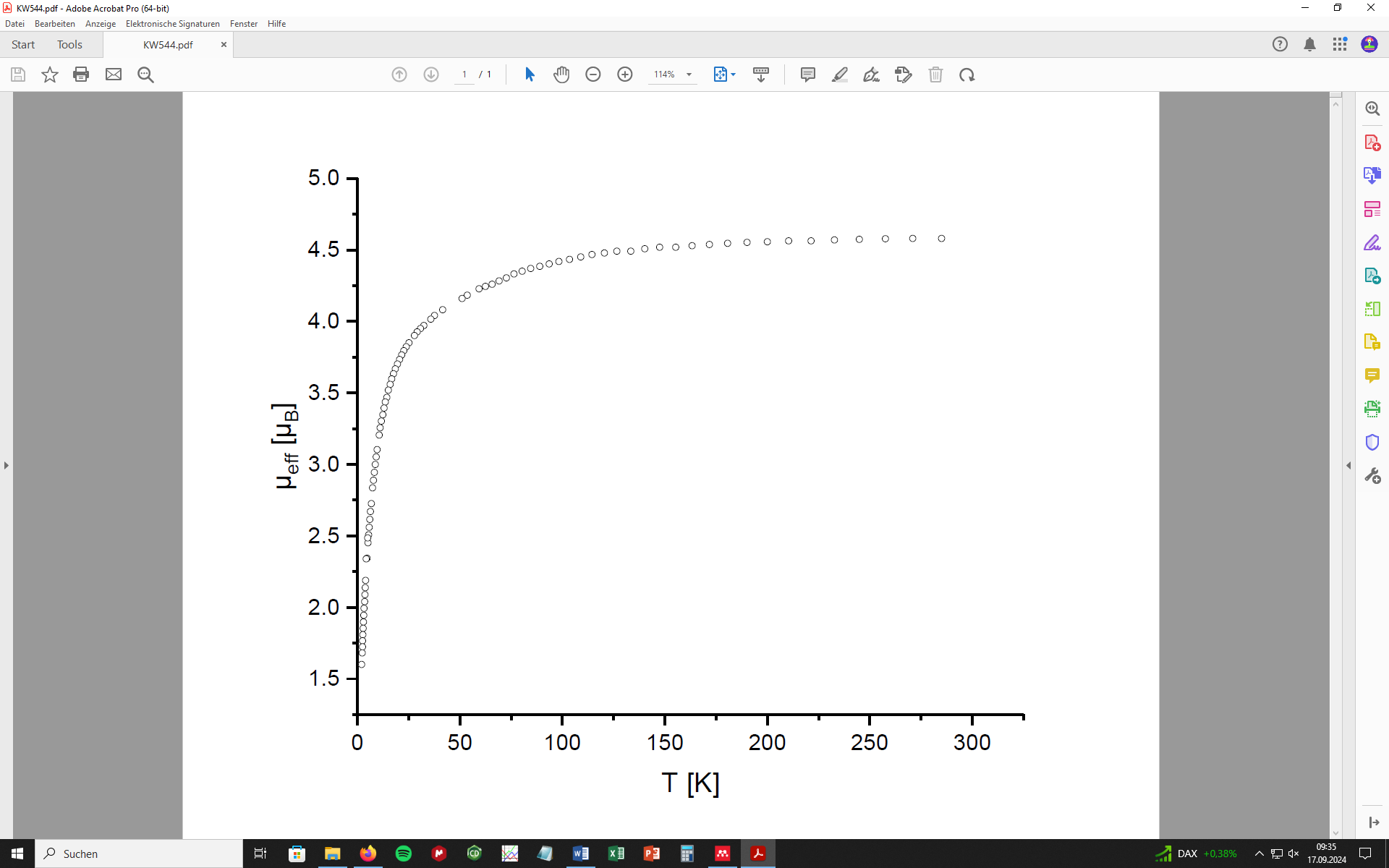


Figure S31. Experimental µ_eff_/µ_B_ *versus* T plot for [Tp^Mes^CoSeCysAm] (7).

Table S2. Experimental magnetic data for [Tp^Mes^CoSeCysAm] (7) obtained from the SQUID measurement.^[a]^

| Temperature [K] | Magnetic Field [Oe] | Magnetic Moment [emu] | χ_mol_ [cm^3^∙mol^-1^] | χ_p_ [cm^3^∙mol^-1^] | μ_eff_ [µ_B_] |
| --- | --- | --- | --- | --- | --- |
| 300.000 | 69999.59 | 0.00649 | 0.00827 | 0.00866 | 4.55931 |
| 285.025 | 69999.59 | 0.00691 | 0.00881 | 0.00920 | 4.58043 |
| 270.989 | 69999.59 | 0.00728 | 0.00928 | 0.00967 | 4.57899 |
| 257.612 | 69999.59 | 0.00767 | 0.00978 | 0.01017 | 4.57704 |
| 244.890 | 69999.59 | 0.00807 | 0.01029 | 0.01068 | 4.57351 |
| 232.813 | 69999.59 | 0.00849 | 0.01082 | 0.01121 | 4.56910 |
| 221.336 | 69999.59 | 0.00892 | 0.01137 | 0.01176 | 4.56345 |
| 210.367 | 69999.59 | 0.00940 | 0.01198 | 0.01237 | 4.56258 |
| 199.970 | 69999.59 | 0.00988 | 0.01260 | 0.01299 | 4.55735 |
| 190.092 | 69999.59 | 0.01038 | 0.01324 | 0.01363 | 4.55166 |
| 180.702 | 69999.59 | 0.01091 | 0.01391 | 0.01430 | 4.54594 |
| 171.790 | 69999.59 | 0.01145 | 0.01460 | 0.01499 | 4.53789 |
| 163.312 | 69999.59 | 0.01202 | 0.01532 | 0.01571 | 4.52922 |
| 155.249 | 69999.59 | 0.01259 | 0.01605 | 0.01644 | 4.51771 |
| 147.406 | 69999.59 | 0.01328 | 0.01693 | 0.01732 | 4.51912 |
| 140.234 | 69999.59 | 0.01390 | 0.01772 | 0.01811 | 4.50667 |
| 133.383 | 69999.59 | 0.01453 | 0.01852 | 0.01891 | 4.49125 |
| 126.641 | 69999.59 | 0.01531 | 0.01952 | 0.01991 | 4.49037 |
| 120.487 | 69999.59 | 0.01602 | 0.02043 | 0.02082 | 4.47866 |
| 114.414 | 69999.59 | 0.01680 | 0.02142 | 0.02181 | 4.46709 |
| 108.915 | 69999.59 | 0.01753 | 0.02235 | 0.02274 | 4.45082 |
| 103.439 | 69999.59 | 0.01834 | 0.02338 | 0.02377 | 4.43403 |
| 98.385 | 69999.59 | 0.01916 | 0.02443 | 0.02482 | 4.41897 |
| 93.527 | 69999.59 | 0.02001 | 0.02550 | 0.02589 | 4.40089 |
| 88.917 | 69999.59 | 0.02091 | 0.02666 | 0.02705 | 4.38548 |
| 84.541 | 69999.59 | 0.02185 | 0.02785 | 0.02824 | 4.36994 |
| 80.397 | 69999.59 | 0.02279 | 0.02905 | 0.02944 | 4.35055 |
| 76.434 | 69999.59 | 0.02376 | 0.03029 | 0.03068 | 4.33057 |
| 72.682 | 69999.59 | 0.02468 | 0.03146 | 0.03185 | 4.30302 |
| 69.106 | 69999.59 | 0.02572 | 0.03279 | 0.03318 | 4.28225 |
| 65.690 | 69999.59 | 0.02679 | 0.03415 | 0.03454 | 4.25989 |
| 62.452 | 69999.59 | 0.02798 | 0.03567 | 0.03606 | 4.24372 |
| 59.363 | 69999.59 | 0.02922 | 0.03725 | 0.03764 | 4.22711 |
| 56.428 | 69999.59 | 0.04120 | 0.05252 | 0.05291 | 4.88635 |
| 53.658 | 69999.59 | 0.03169 | 0.04040 | 0.04079 | 4.18386 |
| 51.009 | 69999.59 | 0.03297 | 0.04203 | 0.04241 | 4.15968 |
| 48.484 | 69999.59 | 0.04098 | 0.05224 | 0.05263 | 4.51727 |
| 46.098 | 69999.59 | 0.04205 | 0.05361 | 0.05400 | 4.46168 |
| 43.821 | 69999.59 | 0.04911 | 0.06260 | 0.06299 | 4.69841 |
| 41.657 | 69999.59 | 0.03893 | 0.04963 | 0.05002 | 4.08211 |
| 39.595 | 69999.59 | 0.05107 | 0.06510 | 0.06549 | 4.55406 |
| 37.665 | 69999.59 | 0.04220 | 0.05380 | 0.05419 | 4.04014 |
| 35.805 | 69999.59 | 0.04388 | 0.05594 | 0.05633 | 4.01610 |
| 34.038 | 69999.59 | 0.05495 | 0.07005 | 0.07044 | 4.37884 |
| 32.357 | 69999.59 | 0.04747 | 0.06051 | 0.06090 | 3.97000 |
| 30.760 | 69999.59 | 0.04946 | 0.06304 | 0.06343 | 3.95034 |
| 29.242 | 69999.59 | 0.05141 | 0.06554 | 0.06593 | 3.92668 |
| 27.799 | 69999.59 | 0.05339 | 0.06806 | 0.06845 | 3.90107 |
| 26.427 | 69999.59 | 0.06609 | 0.08425 | 0.08464 | 4.22942 |
| 25.123 | 69999.59 | 0.05757 | 0.07338 | 0.07377 | 3.84997 |
| 23.883 | 69999.59 | 0.05971 | 0.07611 | 0.07650 | 3.82263 |
| 22.704 | 69999.59 | 0.06187 | 0.07887 | 0.07926 | 3.79360 |
| 21.584 | 69999.59 | 0.06408 | 0.08169 | 0.08208 | 3.76397 |
| 20.518 | 69999.59 | 0.06634 | 0.08456 | 0.08495 | 3.73366 |
| 19.506 | 69999.59 | 0.06859 | 0.08744 | 0.08783 | 3.70148 |
| 18.543 | 69999.59 | 0.07083 | 0.09028 | 0.09067 | 3.66701 |
| 17.628 | 69999.59 | 0.07315 | 0.09325 | 0.09363 | 3.63328 |
| 16.758 | 69999.59 | 0.07540 | 0.09612 | 0.09651 | 3.59642 |
| 15.931 | 69999.59 | 0.07764 | 0.09897 | 0.09936 | 3.55800 |
| 15.145 | 69999.59 | 0.07987 | 0.10182 | 0.10221 | 3.51841 |
| 14.397 | 69999.59 | 0.08166 | 0.10410 | 0.10449 | 3.46861 |
| 13.687 | 69999.59 | 0.08430 | 0.10746 | 0.10785 | 3.43583 |
| 13.011 | 69999.59 | 0.08646 | 0.11022 | 0.11061 | 3.39260 |
| 12.369 | 69999.59 | 0.08854 | 0.11287 | 0.11326 | 3.34719 |
| 11.759 | 69999.59 | 0.09060 | 0.11550 | 0.11589 | 3.30123 |
| 11.178 | 69999.59 | 0.09270 | 0.11816 | 0.11855 | 3.25555 |
| 10.627 | 69999.59 | 0.09451 | 0.12048 | 0.12087 | 3.20509 |
| 10.103 | 69999.59 | 0.10694 | 0.13632 | 0.13671 | 3.32350 |
| 9.604 | 69999.59 | 0.09806 | 0.12500 | 0.12539 | 3.10336 |
| 9.129 | 69999.59 | 0.09971 | 0.12710 | 0.12749 | 3.05099 |
| 8.679 | 69999.59 | 0.10127 | 0.12910 | 0.12949 | 2.99795 |
| 8.251 | 69999.59 | 0.10272 | 0.13094 | 0.13133 | 2.94376 |
| 7.844 | 69999.59 | 0.10405 | 0.13264 | 0.13303 | 2.88874 |
| 7.456 | 69999.59 | 0.10537 | 0.13432 | 0.13471 | 2.83430 |
| 7.088 | 69999.59 | 0.11657 | 0.14860 | 0.14899 | 2.90619 |
| 6.738 | 69999.59 | 0.10778 | 0.13740 | 0.13779 | 2.72497 |
| 6.406 | 69999.59 | 0.10884 | 0.13875 | 0.13914 | 2.66981 |
| 6.090 | 69999.59 | 0.10983 | 0.14001 | 0.14040 | 2.61496 |
| 5.789 | 69999.59 | 0.11072 | 0.14114 | 0.14153 | 2.55975 |
| 5.503 | 69999.59 | 0.11157 | 0.14222 | 0.14261 | 2.50537 |
| 5.232 | 69999.59 | 0.11233 | 0.14319 | 0.14358 | 2.45112 |
| 4.974 | 69999.59 | 0.12145 | 0.15482 | 0.15521 | 2.48488 |
| 4.728 | 69999.59 | 0.11363 | 0.14484 | 0.14523 | 2.34351 |
| 4.495 | 69999.59 | 0.12383 | 0.15786 | 0.15825 | 2.38513 |
| 4.273 | 69999.59 | 0.12525 | 0.15966 | 0.16005 | 2.33881 |
| 4.061 | 69999.59 | 0.11536 | 0.14705 | 0.14744 | 2.18838 |
| 3.862 | 69999.59 | 0.11579 | 0.14760 | 0.14799 | 2.13786 |
| 3.670 | 69999.59 | 0.11624 | 0.14818 | 0.14857 | 2.08831 |
| 3.490 | 69999.59 | 0.11664 | 0.14868 | 0.14907 | 2.03980 |
| 3.318 | 69999.59 | 0.11699 | 0.14913 | 0.14952 | 1.99192 |
| 3.154 | 69999.59 | 0.11730 | 0.14953 | 0.14992 | 1.94471 |
| 2.999 | 69999.59 | 0.11757 | 0.14987 | 0.15026 | 1.89826 |
| 2.850 | 69999.59 | 0.11790 | 0.15030 | 0.15069 | 1.85328 |
| 2.710 | 69999.59 | 0.11811 | 0.15055 | 0.15094 | 1.80859 |
| 2.576 | 69999.59 | 0.11833 | 0.15085 | 0.15124 | 1.76505 |
| 2.449 | 69999.59 | 0.11857 | 0.15114 | 0.15153 | 1.72268 |
| 2.328 | 69999.59 | 0.11878 | 0.15142 | 0.15181 | 1.68122 |
| 2.213 | 69999.59 | 0.12863 | 0.16398 | 0.16437 | 1.70558 |
| 2.103 | 69999.59 | 0.11910 | 0.15183 | 0.15222 | 1.60023 |
| 2.000 | 69999.59 | 0.12767 | 0.16275 | 0.16314 | 1.61533 |

[a] m = 8.4 mg, [b] χ_p_ molar magnetic susceptibility after correction for the diamagnetism of the sample (χ_dia_ = –3.8962∙10^-4^ emu∙mol^-1^, calculated according to Bain *et al.*^[6]^).


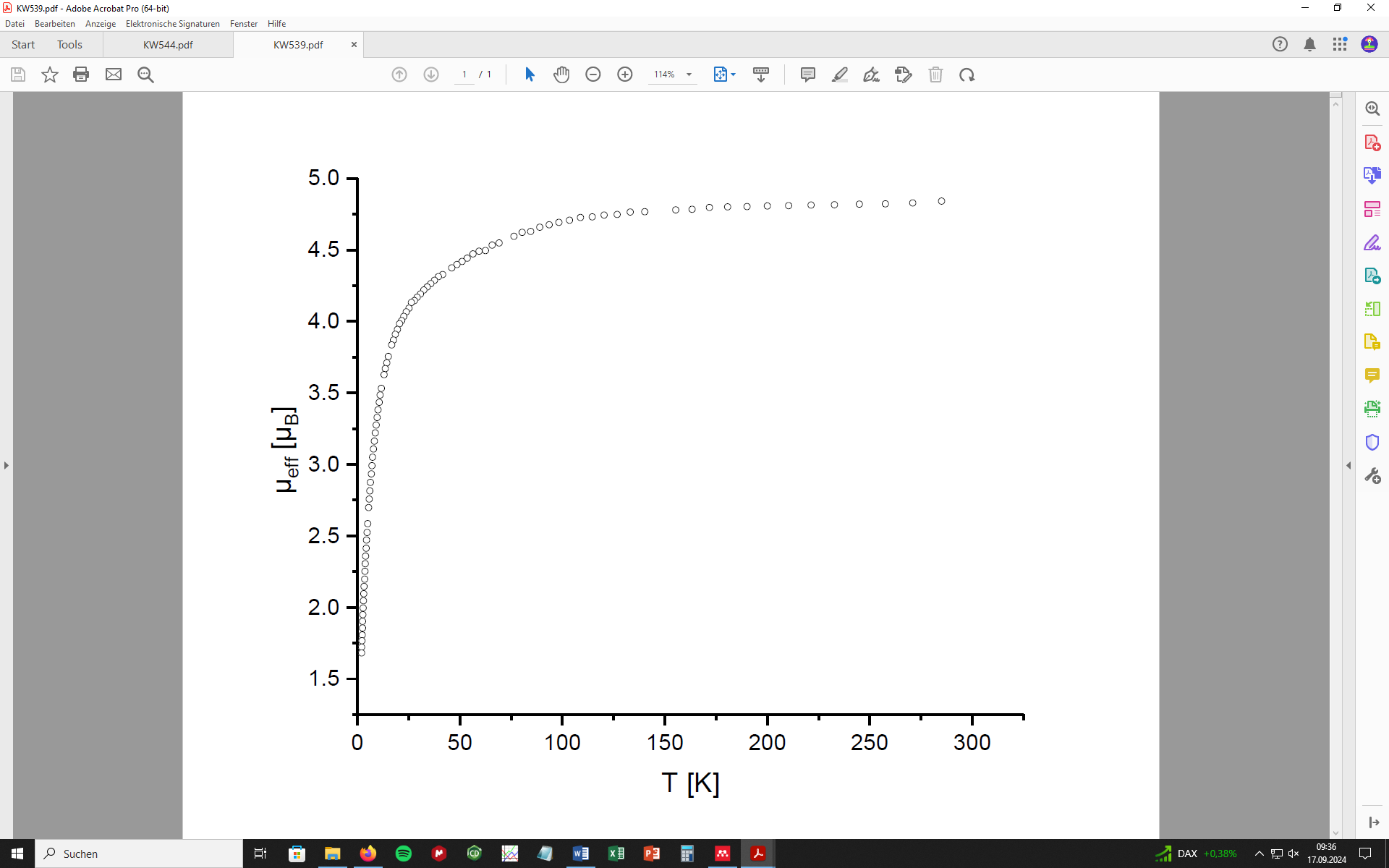


Figure S32. Experimental µ_eff_/µ_B_ *versus* T plot for [Tp^Mes^CoO_2_SeCysAm] (8).

Table S3. Experimental magnetic data for [Tp^Mes^CoO_2_SeCysAm] (8) obtained from the SQUID measurement.^[a]^

| Temperature [K] | Magnetic Field [Oe] | Magnetic Moment [emu] | χ_mol_ [cm^3^∙mol^-1^] | χ_p_ [cm^3^∙mol^-1^] | μ_eff_/µ_B_ [a.u.] |
| --- | --- | --- | --- | --- | --- |
| 299.999 | 69999.73 | 0.00232 | 0.00923 | 0.01038 | 4.80729 |
| 285.052 | 69999.73 | 0.00248 | 0.00988 | 0.01028 | 4.84039 |
| 271.027 | 69999.73 | 0.00260 | 0.01035 | 0.01075 | 4.82762 |
| 257.602 | 69999.73 | 0.00273 | 0.01089 | 0.01129 | 4.82204 |
| 244.889 | 69999.73 | 0.00288 | 0.01146 | 0.01186 | 4.82029 |
| 232.816 | 69999.73 | 0.00302 | 0.01206 | 0.01245 | 4.81548 |
| 221.303 | 69999.73 | 0.00318 | 0.01270 | 0.01309 | 4.81407 |
| 210.402 | 69999.73 | 0.00335 | 0.01335 | 0.01375 | 4.80994 |
| 200.023 | 69999.73 | 0.00352 | 0.01405 | 0.01445 | 4.80754 |
| 190.107 | 69999.73 | 0.00371 | 0.01477 | 0.01517 | 4.80303 |
| 180.696 | 69999.73 | 0.00390 | 0.01555 | 0.01595 | 4.80113 |
| 171.768 | 69999.73 | 0.00410 | 0.01635 | 0.01675 | 4.79624 |
| 163.311 | 69999.73 | 0.00430 | 0.01713 | 0.01753 | 4.78451 |
| 155.251 | 69999.73 | 0.00451 | 0.01800 | 0.01840 | 4.77946 |
| 140.242 | 69999.73 | 0.00498 | 0.01986 | 0.02026 | 4.76706 |
| 133.166 | 69999.73 | 0.00524 | 0.02091 | 0.02131 | 4.76358 |
| 126.750 | 69999.73 | 0.00548 | 0.02184 | 0.02224 | 4.74845 |
| 120.361 | 69999.73 | 0.00576 | 0.02297 | 0.02337 | 4.74332 |
| 114.558 | 69999.73 | 0.00602 | 0.02402 | 0.02442 | 4.72966 |
| 108.836 | 69999.73 | 0.00634 | 0.02526 | 0.02566 | 4.72628 |
| 103.482 | 69999.73 | 0.00662 | 0.02638 | 0.02678 | 4.70763 |
| 98.375 | 69999.73 | 0.00692 | 0.02759 | 0.02799 | 4.69259 |
| 93.531 | 69999.73 | 0.00723 | 0.02881 | 0.02921 | 4.67430 |
| 88.921 | 69999.73 | 0.00755 | 0.03011 | 0.03051 | 4.65776 |
| 84.557 | 69999.73 | 0.00784 | 0.03127 | 0.03167 | 4.62798 |
| 80.385 | 69999.73 | 0.00823 | 0.03282 | 0.03322 | 4.62122 |
| 76.438 | 69999.73 | 0.00856 | 0.03414 | 0.03454 | 4.59496 |
| 69.106 | 69999.73 | 0.00929 | 0.03704 | 0.03744 | 4.54878 |
| 65.703 | 69999.73 | 0.00971 | 0.03872 | 0.03912 | 4.53376 |
| 62.463 | 69999.73 | 0.01005 | 0.04006 | 0.04046 | 4.49568 |
| 59.357 | 69999.73 | 0.01055 | 0.04207 | 0.04247 | 4.48992 |
| 56.434 | 69999.73 | 0.01101 | 0.04391 | 0.04431 | 4.47215 |
| 53.665 | 69999.73 | 0.01143 | 0.04557 | 0.04597 | 4.44175 |
| 51.015 | 69999.73 | 0.01190 | 0.04745 | 0.04785 | 4.41841 |
| 48.493 | 69999.73 | 0.01240 | 0.04945 | 0.04985 | 4.39709 |
| 46.097 | 69999.73 | 0.01292 | 0.05151 | 0.05191 | 4.37478 |
| 41.657 | 69999.73 | 0.01400 | 0.05583 | 0.05623 | 4.32813 |
| 39.600 | 69999.73 | 0.01462 | 0.05832 | 0.05872 | 4.31221 |
| 37.665 | 69999.73 | 0.01519 | 0.06059 | 0.06099 | 4.28613 |
| 35.805 | 69999.73 | 0.01582 | 0.06307 | 0.06347 | 4.26332 |
| 34.038 | 69999.73 | 0.01647 | 0.06566 | 0.06606 | 4.24052 |
| 32.358 | 69999.73 | 0.01715 | 0.06837 | 0.06877 | 4.21862 |
| 30.760 | 69999.73 | 0.01782 | 0.07105 | 0.07145 | 4.19241 |
| 29.242 | 69999.73 | 0.01853 | 0.07390 | 0.07430 | 4.16859 |
| 27.799 | 69999.73 | 0.01927 | 0.07685 | 0.07725 | 4.14409 |
| 26.427 | 69999.73 | 0.02016 | 0.08039 | 0.08079 | 4.13218 |
| 25.123 | 69999.73 | 0.02080 | 0.08294 | 0.08334 | 4.09204 |
| 23.883 | 69999.73 | 0.02160 | 0.08613 | 0.08653 | 4.06533 |
| 22.704 | 69999.73 | 0.02239 | 0.08928 | 0.08967 | 4.03520 |
| 21.584 | 69999.73 | 0.02320 | 0.09249 | 0.09289 | 4.00436 |
| 20.518 | 69999.73 | 0.02414 | 0.09627 | 0.09667 | 3.98287 |
| 19.505 | 69999.73 | 0.02489 | 0.09924 | 0.09964 | 3.94256 |
| 18.543 | 69999.73 | 0.02573 | 0.10259 | 0.10299 | 3.90814 |
| 17.628 | 69999.73 | 0.02654 | 0.10583 | 0.10622 | 3.86984 |
| 16.758 | 69999.73 | 0.02741 | 0.10932 | 0.10971 | 3.83462 |
| 15.145 | 69999.73 | 0.02908 | 0.11595 | 0.11635 | 3.75399 |
| 14.397 | 69999.73 | 0.02987 | 0.11911 | 0.11951 | 3.70958 |
| 13.687 | 69999.73 | 0.03076 | 0.12267 | 0.12307 | 3.67032 |
| 13.011 | 69999.73 | 0.03159 | 0.12596 | 0.12636 | 3.62614 |
| 11.759 | 69999.73 | 0.03316 | 0.13224 | 0.13264 | 3.53180 |
| 11.178 | 69999.73 | 0.03396 | 0.13541 | 0.13581 | 3.48445 |
| 10.627 | 69999.73 | 0.03471 | 0.13839 | 0.13879 | 3.43437 |
| 10.102 | 69999.73 | 0.03540 | 0.14115 | 0.14155 | 3.38167 |
| 9.604 | 69999.73 | 0.03607 | 0.14382 | 0.14422 | 3.32821 |
| 9.130 | 69999.73 | 0.03673 | 0.14645 | 0.14684 | 3.27441 |
| 8.679 | 69999.73 | 0.03736 | 0.14896 | 0.14936 | 3.21984 |
| 8.251 | 69999.73 | 0.03788 | 0.15104 | 0.15144 | 3.16116 |
| 7.843 | 69999.73 | 0.03849 | 0.15349 | 0.15389 | 3.10693 |
| 7.456 | 69999.73 | 0.03899 | 0.15549 | 0.15589 | 3.04895 |
| 7.089 | 69999.73 | 0.03948 | 0.15741 | 0.15781 | 2.99107 |
| 6.739 | 69999.73 | 0.03991 | 0.15913 | 0.15953 | 2.93214 |
| 6.406 | 69999.73 | 0.04031 | 0.16075 | 0.16115 | 2.87333 |
| 6.085 | 69999.73 | 0.04071 | 0.16232 | 0.16272 | 2.81399 |
| 5.789 | 69999.73 | 0.04108 | 0.16380 | 0.16420 | 2.75731 |
| 5.504 | 69999.73 | 0.04134 | 0.16484 | 0.16523 | 2.69681 |
| 4.973 | 69999.73 | 0.04200 | 0.16747 | 0.16787 | 2.58403 |
| 4.726 | 69999.73 | 0.04216 | 0.16811 | 0.16851 | 2.52377 |
| 4.495 | 69999.73 | 0.04246 | 0.16932 | 0.16972 | 2.46999 |
| 4.273 | 69999.73 | 0.04265 | 0.17007 | 0.17047 | 2.41364 |
| 4.062 | 69999.73 | 0.04284 | 0.17083 | 0.17123 | 2.35846 |
| 3.862 | 69999.73 | 0.04304 | 0.17161 | 0.17201 | 2.30489 |
| 3.671 | 69999.73 | 0.04318 | 0.17219 | 0.17259 | 2.25104 |
| 3.490 | 69999.73 | 0.04329 | 0.17260 | 0.17300 | 2.19738 |
| 3.318 | 69999.73 | 0.04343 | 0.17317 | 0.17357 | 2.14606 |
| 3.154 | 69999.73 | 0.04354 | 0.17361 | 0.17401 | 2.09504 |
| 2.998 | 69999.73 | 0.04365 | 0.17407 | 0.17447 | 2.04546 |
| 2.850 | 69999.73 | 0.04373 | 0.17437 | 0.17477 | 1.99596 |
| 2.710 | 69999.73 | 0.04378 | 0.17457 | 0.17497 | 1.94726 |
| 2.575 | 69999.73 | 0.04389 | 0.17501 | 0.17541 | 1.90076 |
| 2.449 | 69999.73 | 0.04395 | 0.17524 | 0.17564 | 1.85464 |
| 2.327 | 69999.73 | 0.04389 | 0.17500 | 0.17539 | 1.80676 |
| 2.212 | 69999.73 | 0.04404 | 0.17562 | 0.17602 | 1.76479 |
| 2.104 | 69999.73 | 0.04408 | 0.17578 | 0.17618 | 1.72167 |
| 2.001 | 69999.73 | 0.04412 | 0.17592 | 0.17632 | 1.67993 |

[a] m = 2.8 mg, [b] χ_p_ molar magnetic susceptibility after correction for the diamagnetism of the sample (χ_dia_ = –3.988∙10^-4^ emu∙mol^-1^, calculated according to Bain *et al.*^[6]^).

**^18^O_2_-labelling experiment and ESI-QMS measurements**

A solution of Tp^Mes^CoSeCysAm (**7**) was dissolved in THF and the flask was subsequently connected to a vaccum line and an ^18^O_2_-cylinder (2.78 bar) *via* a T-piece. The system was evacuated, the solution degassed three times (freeze-pump-thaw) and the flask subsequently filled with ^18^O_2_. Following the addition of the gas, the solution was heated up to 40 °C and stirred for 3 h until the pink solution had turned colorless. An ESI-QMS spectrum was recorded from the crude reaction solution.

Scheme S1. Reaction of Tp^Mes^CoSeCysAm (7) with ^18^O_2_.


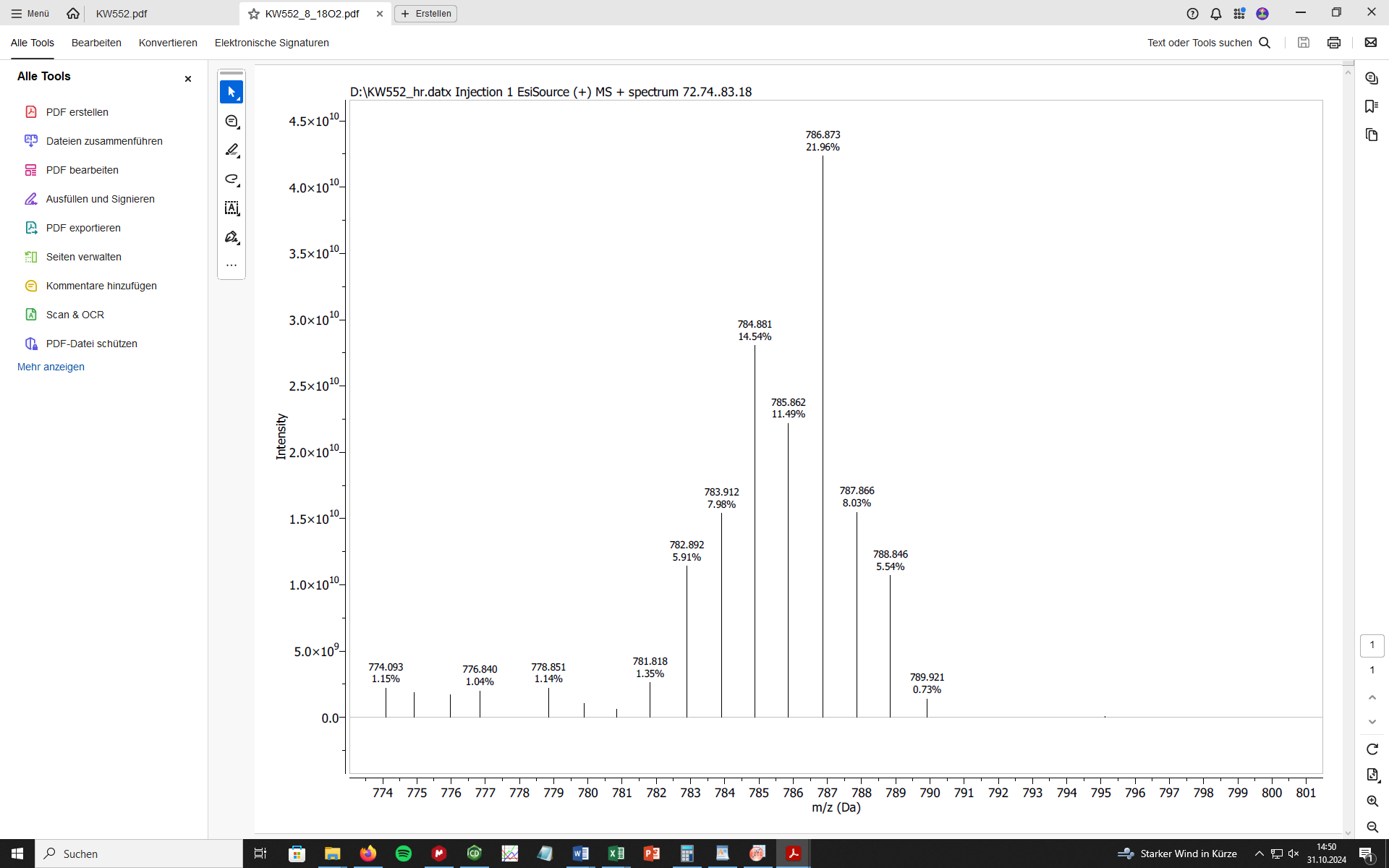


Figure S33. Section of the ESI-MS spectrum of [Tp^Mes^Co^18^O_2_SeCysAm] 8-[^18^O_2_], showing the isotopic pattern attributed to [C_38_H_46_BCoN_7_^18^O_2_Se]^+^.


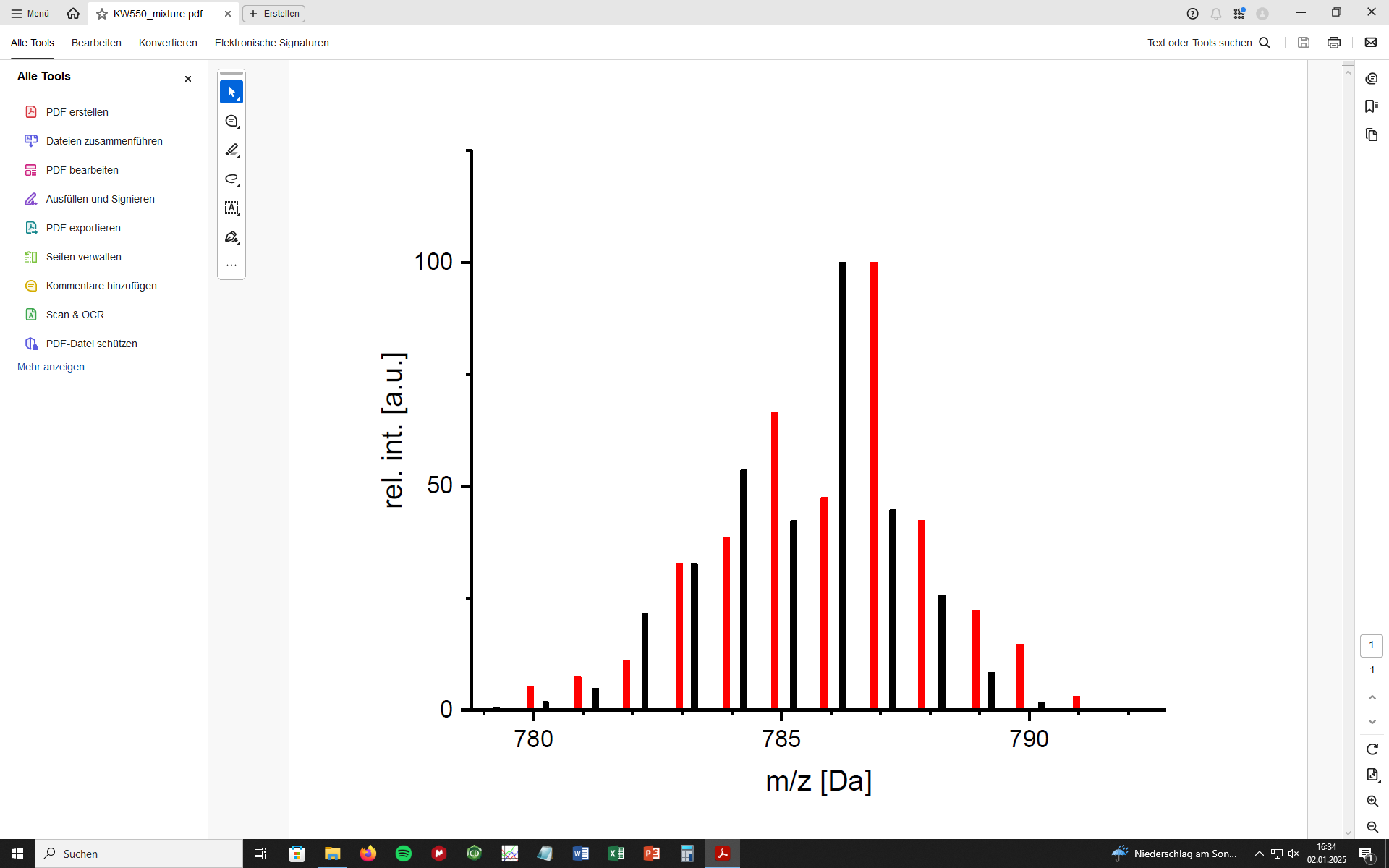


Figure S34. Isotope pattern of the ESI-MS product peak after the reaction shown in Scheme S1 (red [Tp^Mes^Co^18^O_2_SeCysAm]^+^) and the respective calculated spectrum (black). The measured data deviates from the calculate data by +0.633 Da. This discrepancy is attributed to instrumental error.

**^18^O_2_/^16^O_2_-labelling experiment and ESI-TOF measurements**

A mixture of ^18^O_2_ (200 mbar) and ^16^O_2_ (200 mbar) was prepared in a closed gas line that had been evacuated beforehand. The mixture was left to stand overnight to ensure a homogeneous blend. A flask equipped with Tp^Mes^CoSeCysAm (**7**) dissolved in benzene was degassed thrice *via* freeze-pump-thaw and connected to the line. The gas mixture was added to the evacuated flask and the solution was stirred for 3 h at 40 °C. An ESI-MS spectrum was recorded from the crude reaction solution.

Scheme S2. Reaction of TpMesCoSeCysAm (7) with a 1:1 mixture of ^16^O_2_ and ^18^O_2_.


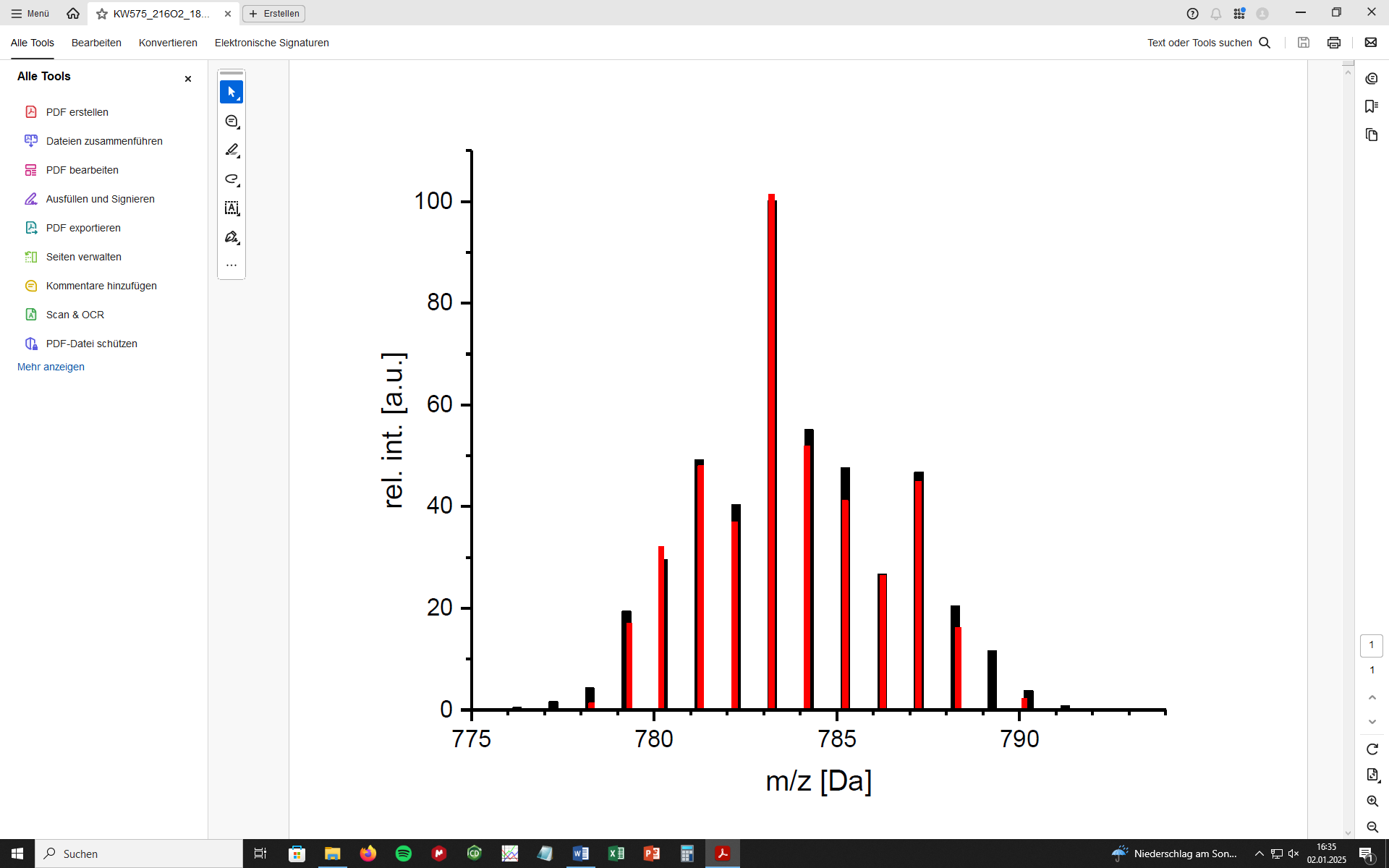


Figure S35. Isotope pattern of the ESI-MS product peaks after the reaction shown in Scheme S2 (red) and the respective spectrum calculated for a mixture of [TpMesCo^16^O_2_SeCysAm+H^+^] and [TpMesCo^18^O_2_SeCysAm+H^+^] in a ratio of 2:1 (black). Despite ensuring a homogenous 1:1 blend of the gases, the 2:1 mixture of the product complexes was reproducible.


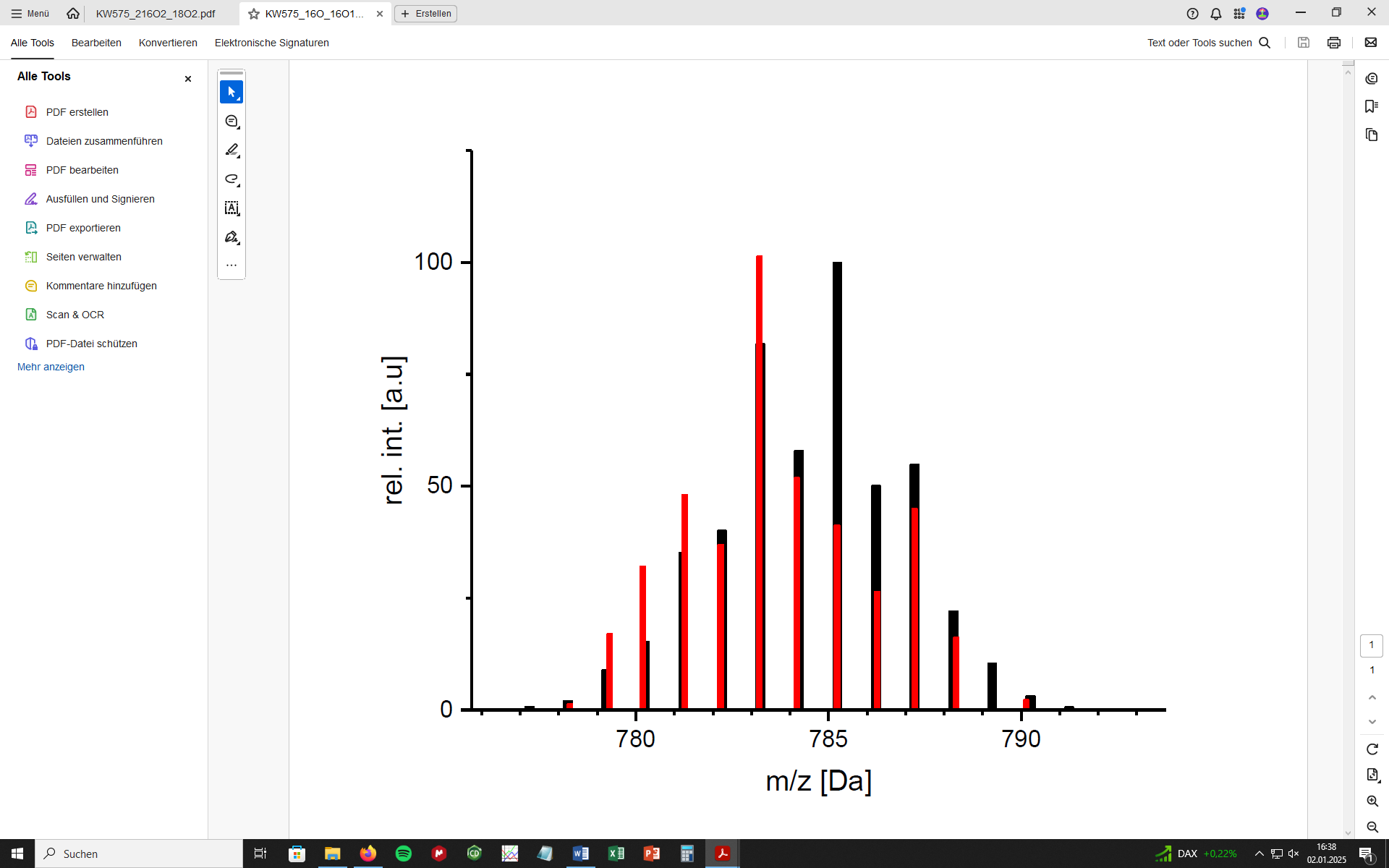


Figure S36. Isotope pattern of the ESI-MS product peaks after the reaction shown in Scheme S2 (red) and for comparison the spectrum calculated for a hypothetical statistical 1:2:1 mixture ([8-^16^O_2_]:[8-^16^O^18^O]:[8-^18^O_2_] as proton adducts) assuming O atom scrambling (black).

Single crystal X-ray crystallography

The data collections were performed with a BRUKER D8 VENTURE area detector with Mo-Kα radiation (λ = 0.71073 Å) at 100 K. Multi-scan absorption corrections implemented in SADABS^[8]^ were applied to the data. The structures were solved by intrinsic phasing method (SHELXT-2019)^[9,10]^ and refined by full matrix least square procedures based on F2 with all measured reflections (SHELXL-2014 in the graphical user interface SHELXle)^[11]^ with anisotropic temperature factors for all non-hydrogen atoms. All hydrogen atoms were added geometrically and refined by using a riding model except for the boron and amine hydrogens which were found in the Fourier residual electron density map and freely refined. For the complexes **4a** and **7** a squeeze refinement^[12]^ has been applied.

The data have been deposited with the CCDC (deposition numbers 2287742 (**4a**), 2287743 (**4b**), 2287744 (**5a**), 2287745 (**7**) and 2287746 (**8**)).

Data can be obtained free of charge from The Cambridge Crystallographic Data Centre via [www.ccdc.cam.ac.uk/data_request/cif](http://www.ccdc.cam.ac.uk/data_request/cif).


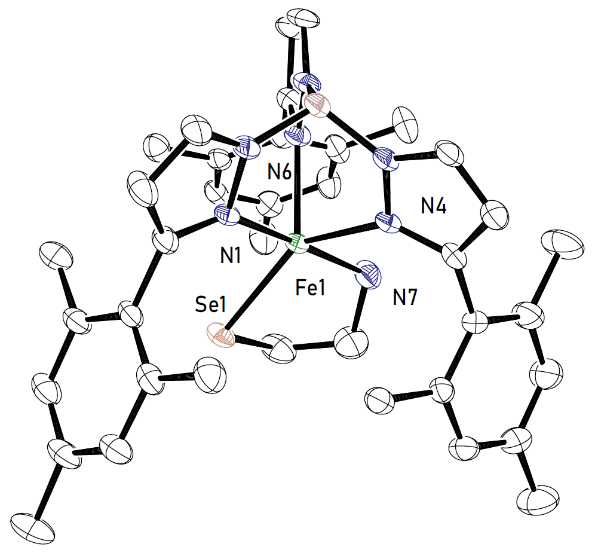


Table S4. Selected bond distances and angles of complex [TpMesFeSeCysAm] 4a.

| bond |  | distance [Å] |  | atoms | angle [°] |
| --- | --- | --- | --- | --- | --- |
| Fe1-Se1 |  | 2.4366(6) |  | Se1-Fe1-N1 | 96.37(9) |
| Fe1-N7 |  | 2.250(3) |  | Se1-Fe1-N4 | 143.58(8) |
| Fe1-N1 |  | 2.253(3) |  | Se1-Fe1-N6 | 128.55(8) |
| Fe1-N4 |  | 2.121(3) |  | Se1-Fe1-N7 | 84.67(9) |
| Fe1-N6 |  | 2.114(3) |  | N1-Fe1-N4 | 85.0(1) |
|  |  |  |  | N1-Fe1-N6- | 87.7(1) |
|  |  |  |  | N1-Fe1-N7 | 174.9(1) |
|  |  |  |  | N4-Fe1-N6 | 87.8(1) |
|  |  |  |  | N4-Fe1-N7 | 91.1(1) |
|  |  |  |  | N6-Fe1-N7 | 95.6(1) |

*
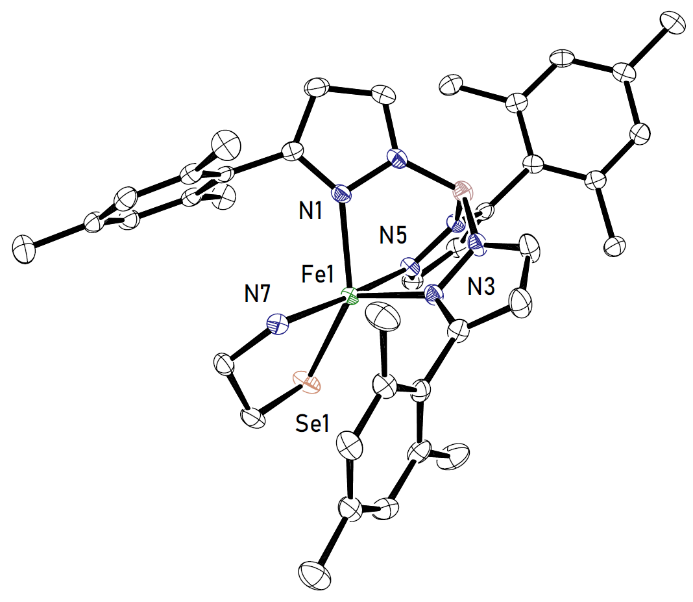
*

Table S5. Selected bond distances and angles of complex [TpMes*FeSeCysAm] 4b.

| bond |  | distance [Å] |  | atoms | angle [°] |
| --- | --- | --- | --- | --- | --- |
| Fe1-Se1 |  | 2.4510(5) |  | Se1-Fe1-N1 | 143.07(5) |
| Fe1-N7 |  | 2.213(1) |  | Se1-Fe1-N3 | 129.53(5) |
| Fe1-N1 |  | 2.142(2) |  | Se1-Fe1-N5 | 91.41(4) |
| Fe1-N3 |  | 2.104(3) |  | Se1-Fe1-N7 | 86.58(5) |
| Fe1-N5 |  | 2.177(1) |  | N1-Fe1-N3 | 87.23(6) |
|  |  |  |  | N1-Fe1-N5- | 85.02(6) |
|  |  |  |  | N1-Fe1-N7 | 93.67(6) |
|  |  |  |  | N3-Fe1-N5 | 88.91(6) |
|  |  |  |  | N3-Fe1-N7 | 96.09(6) |
|  |  |  |  | N5-Fe1-N7 | 174.74(6) |


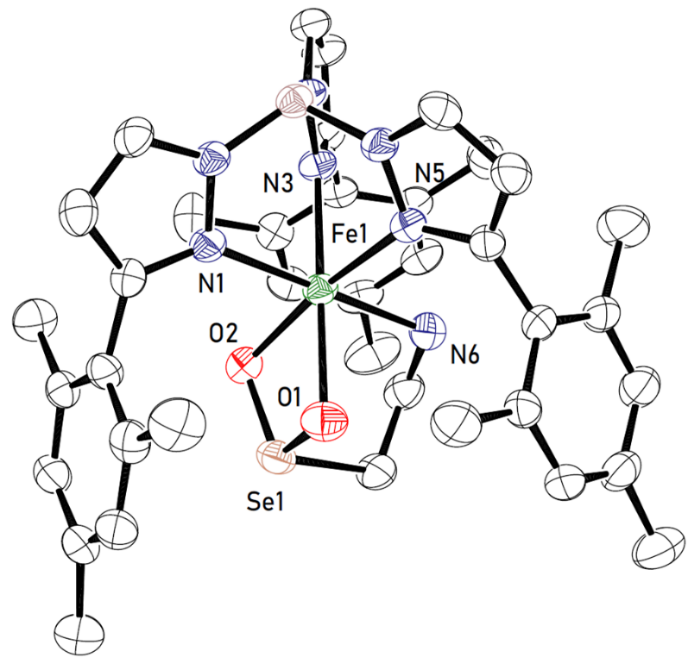


Table S6. Selected bond distances and angles of complex [TpMesFeO_2_SeCysAm] 5a.

| bond |  | distance [Å] |  | atoms | angle [°] |
| --- | --- | --- | --- | --- | --- |
| Fe1-O1 |  | 2.167(3) |  | O1-Fe1-O2 | 72.7(2) |
| Fe1-O2 |  | 2.177(3) |  | O1-Fe1-N6 | 84.2(2) |
| Fe1-N1 |  | 2.198(4) |  | O1-Fe1-N1 | 96.4(2) |
| Fe1-N3 |  | 2.200(4) |  | O1-Fe1-N3 | 175.6(2) |
| Fe1-N5 |  | 2.202(5) |  | O1-Fe1-N5 | 98.9(2) |
| Fe1-N6 |  | 2.257(4) |  | O2-Fe1-N6- | 85.2(2) |
| Se1-O1 |  | 1.691(5) |  | O2-Fe1-N1 | 94.1(2) |
| Se1-O2 |  | 1.685(4) |  | O2-Fe1-N3 | 104.0(2) |
|  |  |  |  | O2-Fe1-N5 | 171.5(2) |
|  |  |  |  | N6-Fe1-N1 | 178.9(2) |
|  |  |  |  | N6-Fe1-N3 | 92.7(2) |
|  |  |  |  | N6-Fe1-N5 | 93.2(2) |
|  |  |  |  | N1-Fe1-N3 | 86.7(2) |
|  |  |  |  | N1-Fe1-N5 | 87.7(2) |
|  |  |  |  | N3-Fe1-N5 | 84.4(2) |
|  |  |  |  | O1-Se1-O2 | 99.3(2) |


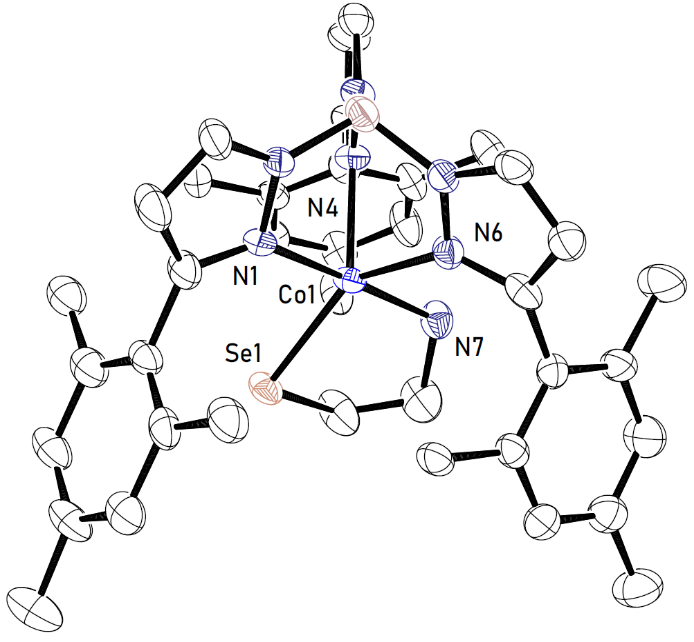


Table S7. Selected bond distances and angles of complex [TpMesCoSeCysAm] 7.

| bond |  | distance [Å] |  | atoms | angle [°] |
| --- | --- | --- | --- | --- | --- |
| Co1-Se1 |  | 2.389(1) |  | Se1-Co1-N1 | 95.5(2) |
| Co1-N7 |  | 2.195(7) |  | Se1-Co1-N4 | 129.3(2) |
| Co1-N1 |  | 2.238(7) |  | Se1-Co1-N6 | 142.8(2) |
| Co1-N4 |  | 2.085(7) |  | Se1-Co1-N7 | 85.2(2) |
| Co1-N6 |  | 2.086(7) |  | N1-Co1-N4 | 88.3(3) |
|  |  |  |  | N1-Co1-N6- | 84.9(3) |
|  |  |  |  | N1-Co1-N7 | 175.2(2) |
|  |  |  |  | N4-Co1-N6 | 87.9(3) |
|  |  |  |  | N4-Co1-N7 | 95.0(3) |
|  |  |  |  | N6-Co1-N7 | 91.7(3) |


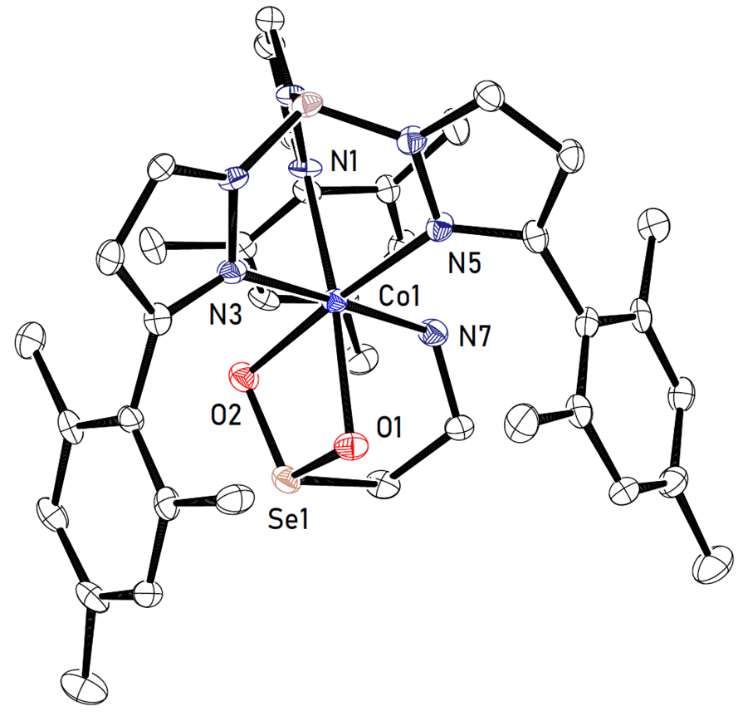


Table S8. Selected bond distances and angles of complex [TpMesCoO_2_SeCysAm] 8.

| bond |  | distance [Å] |  | atoms | angle [°] |
| --- | --- | --- | --- | --- | --- |
| Co1-O1 |  | 2.166(5) |  | O1-Co1-N1 | 171.8(2) |
| Co1-O2 |  | 2.137(4) |  | O1-Co1-N7 | 73.8(2) |
| Co1-N1 |  | 2.162(6) |  | O1-Co1-N1 | 92.7(2) |
| Co1-N3 |  | 2.155(6) |  | O1-Co1-N3 | 103.0(2) |
| Co1-N5 |  | 2.169(5) |  | O1-Co1-N5 | 85.6(2) |
| Co1-N7 |  | 2.203(6) |  | N1-Co1-O2- | 98.0(2) |
| Se1-O1 |  | 1.696(4) |  | N1-Co1-N3 | 88.3(2) |
| Se1-O2 |  | 1.698(5) |  | N1-Co1-N5 | 85.2(2) |
|  |  |  |  | N1-Co1-N7 | 93.4(2) |
|  |  |  |  | O2-Co1-N3 | 95.2(2) |
|  |  |  |  | O2-Co1-N5 | 175.9(2) |
|  |  |  |  | O2-Co1-N7 | 84.6(2) |
|  |  |  |  | N3-Co1-N5 | 87.4(2) |
|  |  |  |  | N3-Co1-N7 | 178.3(2) |
|  |  |  |  | N5-Co1-N7 | 92.7(2) |
|  |  |  |  | O1-Se1-O2 | 99.1(2) |

**Computational Data and Results**

**Methods**

All calculations were performed using the Gaussian-09 software package with the unrestricted B3LYP density functional theory approach.^[13–15]^ Geometry optimizations, analytical frequencies and constraint geometry scans were done with a split basis set that contains LACV3P+ with electron core potential on iron and 6-311+G* on the rest of the atoms, basis set BS2.^[16–18]^ The calculations also included the conductor-type polarized continuum model with a dielectric constant mimicking diethylether.^[19]^ Free energies were calculated from the frequency calculations and use the electronic energy supplemented with zero-point, thermal and entropic corrections at 273 K. The QTAIM calculations are performed using the AIM2000 package.^[20]^ The Mössbauer spectral parameters of the species were computed at the B3LYP/TZVP level of theory, using the ORCA software suite, incorporating COSMO solvation effects.^[21]^ The MB isomer shifts (IS) were calculated based on the calibration constants reported by Römelt *et al*.^[22]^

Scheme S3. Reaction mechanism and models explored in this work.

Figure S37. UB3LYP/BS2 optimized geometries of ^5,3,1^RC_Fe/S_ and ^5,3^RC_Fe/Se_ as obtained in Gaussian-09. Bond lengths are in Å.

Figure S38. UB3LYP/BS2 optimized geometries of ^5^TS1_Fe/S_ and ^5,3^IM1_Fe/S_ as obtained in Gaussian-09. Bond lengths are in Å and the imaginary frequency of the transition state in cm^−1^.

Figure S39. UB3LYP/BS2 optimized geometries of ^5,3^TS2_Fe/S_ and ^5,3^IM2_Fe/S_ as obtained in Gaussian-09. Bond lengths are in Å and the imaginary frequency of the transition state in cm^−1^.

Figure S40. UB3LYP/BS2 optimized geometries ^5,3^IM3_Fe/S_ and ^5^IM3’_Fe/S_ as obtained in Gaussian-09. Bond lengths are in Å. In IM3 the sulfinic acid group is bidentate bound and in IM3’ it is monodentate bound.

Figure S41. UB3LYP/BS2 calculated constraint geometry scan for the second oxygen atom transfer to sulfur starting from ^5^IM2_Fe/S_ on the right. As can be seen the barrier is smaller than 1 kcal mol^−1^.

Figure S42. UB3LYP/BS2 optimized geometries of ^5^TS1_Fe/Se_ and ^5^IM1_Fe/Se_ as obtained in Gaussian-09. Bond lengths are in Å and the imaginary frequency of the transition state in cm^−1^.

Figure S43. UB3LYP/BS2 optimized geometries of ^5,3^TS2_Fe/Se_ and ^5,3^IM2_Fe/Se_ as obtained in Gaussian-09. Bond lengths are in Å and the imaginary frequency of the transition state in cm^−1^.

Figure S44. UB3LYP/BS2 optimized geometries of ^5^IM3_Fe/Se_ and ^5^IM3’_Fe/Se_ as obtained in Gaussian-09. Bond lengths are in Å.

Figure S45. UB3LYP/BS2 optimized geometries of ^2^RC_Co/Se_ and ^2^TS1_Co/Se_ as obtained in Gaussian-09. Bond lengths are in Å and the imaginary frequency of the transition state in cm^−1^.

Figure S46. UB3LYP/BS2 optimized geometries of ^2^IM1_Co/Se_ and ^2^TS2_Co/Se_ as obtained in Gaussian-09. Bond lengths are in Å and the imaginary frequency of the transition state in cm^−1^.

Figure S47. UB3LYP/BS2 optimized geometries of ^2^IM2_Co/Se_ and ^2^IM3_Co/Se_ as obtained in Gaussian-09. Bond lengths are in Å.The right-hand-side shows an overlay of the crystal structure with the DFT optimized geometry.

Figure S48. Singly occupied molecular orbitals of ^5^RC_Fe/S_.

Figure S49. Singly occupied molecular orbitals of ^5^RC_Fe/Se_.

Figure S50. Singly occupied molecular orbitals of ^2^RC_Co/Se_.

Figure S51. Optimized geometry of ^5^4b structure and natural orbitals.

Figure S52. Optimized geometry of ^2^7 structure and natural orbital.

Figure S53. Constraint geometry scans from the Co/Se and Fe/S metal-superoxo complexes for proximal attack on the selenium/sulfur atom of the substrate. As can be seen, the barriers are considerably higher in energy than those for the stepwise mechanism reported above. Moreover, the metal-oxygen bond breaks.

Table S9. Absolute (in au) and relative (in kcal mol^−1^) relative (free) energies of optimized structures for the reaction mechanism of dioxygenation of cysteamine in ^5,3^RC_Fe/S_ as obtained in Gaussian-09.

|  | E [BS1, au] | ZPE [au] | G [BS1] | ΔE | ΔE+ZPE | ΔG |
| --- | --- | --- | --- | --- | --- | --- |
| ^5^**RC**_Fe/S_ | -2556.55090 | 0.78071 | -2555.85062 | 0.00 | 0.00 | 0.00 |
| ^5^**TS1**_Fe/S_ | -2556.53720 | 0.77885 | -2555.84014 | 8.60 | 7.43 | 6.58 |
| ^5^**IM1**_Fe/S_ | -2556.56751 | 0.78052 | -2555.86781 | -10.42 | -10.54 | -10.79 |
| ^5^**TS2**_Fe/S_ | -2556.55783 | 0.77970 | -2555.85766 | -4.35 | -4.99 | -4.42 |
| ^5^**IM2**_Fe/S_ | -2556.58556 | 0.78191 | -2555.88464 | -21.75 | -21.00 | -21.34 |
| ^5^**IM3**_Fe/S_ | -2556.65801 | 0.78178 | -2555.95264 | -67.21 | -66.54 | -64.02 |
| ^3^**RC**_Fe/S_ | -2556.54379 | 0.78343 | -2555.83764 | 4.46 | 6.17 | 8.15 |
| ^3^**IM1**_Fe/S_ | -2556.54598 | 0.78281 | -2555.84190 | 3.08 | 4.40 | 5.48 |
| ^3^**TS2**_Fe/S_ | -2556.54338 | 0.78178 | -2555.83912 | 4.72 | 5.39 | 7.22 |
| ^3^**IM2**_Fe/S_ | -2556.58645 | 0.78225 | -2555.88367 | -22.30 | -21.34 | -20.74 |
| ^3^**IM3**_Fe/S_ | -2556.63576 | 0.78424 | -2555.92925 | -53.25 | -51.04 | -49.34 |
| ^1^**RC**_Fe/S_ | -2556.54886 | 0.78357 | -2555.84041 | 1.28 | 3.08 | 6.41 |

Table S10. Group spin densities and charges of optimized structures for the reaction mechanism of dioxygenation of cysteamine in ^5,3^RC_Fe/S_ as obtained in Gaussian-09.

|  | Spin | | | | Charge | | | |
| --- | --- | --- | --- | --- | --- | --- | --- | --- |
|  | Fe | CysS | OO | Ligand | Fe | CysS | OO | Ligand |
| ^5^**RC**_Fe/S_ | 4.08 | 0.61 | -0.74 | 0.05 | -1.04 | 0.04 | 0.26 | 0.74 |
| ^5^**TS1**_Fe/S_ | 3.77 | 0.65 | -0.47 | 0.05 | -0.86 | 0.58 | -0.10 | 0.38 |
| ^5^**IM1**_Fe/S_ | 3.82 | 0.04 | 0.11 | 0.02 | -0.76 | 0.79 | -0.19 | 0.16 |
| ^5^**TS2**_Fe/S_ | 3.80 | 0.00 | 0.15 | 0.05 | -1.02 | 0.81 | -0.13 | 0.34 |
| ^5^**IM2**_Fe/S_ | 2.59 | 0.60 | 0.91 | -0.10 | -0.81 | 0.94 | -0.55 | 0.42 |
| ^5^**IM3**_Fe/S_ | 3.89 | 0.06 | 0.03 | 0.02 | -0.44 | 1.03 | -0.58 | 0.00 |
| ^3^**RC**_Fe/S_ | 0.97 | 0.11 | 1.00 | -0.08 | -3.58 | 0.93 | 0.46 | 2.19 |
| ^3^**IM1**_Fe/S_ | 1.97 | -0.02 | 0.05 | -0.01 | -1.31 | 0.74 | -0.01 | 0.59 |
| ^3^**TS2**_Fe/S_ | 2.07 | -0.09 | 0.04 | -0.01 | -1.45 | 0.80 | -0.03 | 0.67 |
| ^3^**IM2**_Fe/S_ | 2.43 | -0.50 | 0.09 | -0.02 | -1.18 | 1.02 | -0.51 | 0.67 |
| ^3^**IM3**_Fe/S_ | 1.89 | 0.01 | 0.03 | 0.08 | -1.56 | 1.36 | -0.68 | 0.89 |
| ^1^**RC**_Fe/S_ | -1.14 | -0.04 | 1.07 | 0.11 | -3.67 | 0.98 | 0.54 | 2.16 |

Table S11. Absolute (in au) and relative (in kcal mol^−1^) relative (free) energies of optimized structures for the reaction mechanism of dioxygenation of selenocysteamine in ^5,3^RC_Fe/Se_ as obtained in Gaussian-09.

|  | E [BS1, au] | ZPE [au] | G [BS1] | ΔE | ΔE+ZPE | ΔG |
| --- | --- | --- | --- | --- | --- | --- |
| ^5^**RC**_Fe/Se_ | -4559.88573 | 0.77993 | -4559.18698 | 0.00 | 0.00 | 0.00 |
| ^5^**TS1**_Fe/Se_ | -4559.87968 | 0.77853 | -4559.18239 | 3.80 | 2.92 | 2.88 |
| ^5^**IM1**_Fe/Se_ | -4559.90244 | 0.77940 | -4559.20438 | -10.49 | -10.82 | -10.92 |
| ^5^**TS2**_Fe/Se_ | -4559.88902 | 0.77862 | -4559.19140 | -2.06 | -2.89 | -2.78 |
| ^5^**IM2**_Fe/Se_ | -4559.91433 | 0.78061 | -4559.21620 | -17.95 | -17.52 | -18.34 |
| ^5^**IM3**_Fe/Se_ | -4559.97936 | 0.77953 | -4559.27854 | -58.75 | -59.01 | -57.45 |
| ^3^**RC**_Fe/Se_ | -4559.87655 | 0.78248 | -4559.17219 | 5.76 | 7.36 | 9.28 |

Table S12. Group spin densities and charges of optimized structures for the reaction mechanism of dioxygenation of selenocysteamine in ^5,3^RC_Fe/Se_ as obtained in Gaussian-09.

|  | Spin | | | | Charge | | | |
| --- | --- | --- | --- | --- | --- | --- | --- | --- |
|  | Fe | CysSe | OO | Ligand | Fe | CysSe | OO | Ligand |
| ^5^**RC**_Fe/Se_ | 4.17 | 0.60 | -0.77 | 0.00 | -1.22 | 0.47 | 0.23 | 0.52 |
| ^5^**TS1**_Fe/Se_ | 3.89 | 0.72 | -0.60 | 0.00 | -0.25 | 0.40 | -0.11 | -0.05 |
| ^5^**IM1**_Fe/Se_ | 3.80 | 0.06 | 0.12 | 0.03 | -0.60 | 0.91 | -0.21 | -0.10 |
| ^5^**TS2**_Fe/Se_ | 3.81 | 0.04 | 0.10 | 0.05 | -1.13 | 1.09 | -0.15 | 0.19 |
| ^5^**IM2**_Fe/Se_ | 2.52 | 0.60 | 0.95 | -0.07 | -1.18 | 1.31 | -0.67 | 0.54 |
| ^5^**IM3‘**_Fe/Se_ | 3.81 | 0.07 | 0.10 | 0.02 | -1.41 | 1.45 | -0.81 | 0.77 |
| ^5^**IM3**_Fe/Se_ | 3.91 | 0.05 | 0.04 | 0.00 | -0.41 | 1.31 | -0.68 | -0.22 |
| ^3^**RC**_Fe/Se_ | 1.02 | 0.05 | 0.99 | -0.06 | -2.04 | 0.63 | 0.33 | 1.07 |

**Table S13.** Absolute (in au) and relative (in kcal mol^−1^) relative (free) energies of optimized structures for the reaction mechanism of dioxygenation of selenocysteamine in ^2^RC_Co/Se_ as obtained in Gaussian-09.

|  | E [BS1, au] | ZPE [au] | G [BS1] | ΔE | ΔE+ZPE | ΔG |
| --- | --- | --- | --- | --- | --- | --- |
| ^2^**RC**_Co/Se_ | -4581.51775 | 0.78270 | -4580.81307 | 0.00 | 0.00 | 0.00 |
| ^2^**TS1**_Co/Se_ | -4581.49449 | 0.78204 | -4580.78984 | 14.60 | 14.19 | 14.57 |
| ^2^**IM1**_Co/Se_ | -4581.52000 | 0.78224 | -4580.81274 | -1.41 | -1.69 | 0.21 |
| ^2^**TS2**_Co/Se_ | -4581.49849 | 0.77983 | -4580.79874 | 12.09 | 10.29 | 8.99 |
| ^2^**IM2**_Co/Se_ | -4581.53223 | 0.78106 | -4580.83106 | -9.09 | -10.11 | -11.29 |
| ^2^**IM3**_Co/Se_ | -4581.59317 | 0.78175 | -4580.88594 | -47.33 | -47.92 | -45.73 |

Table S14. Group spin densities and charges of optimized structures for the reaction mechanism of dioxygenation of selenocysteamine in ^2^RC_Co/Se_ as obtained in Gaussian-09.

|  | Spin | | | | Charge | | | |
| --- | --- | --- | --- | --- | --- | --- | --- | --- |
|  | Co | CysSe | OO | Ligand | Co | CysSe | OO | Ligand |
| ^2^**RC**_Co/Se_ | -0.32 | 0.11 | 1.18 | 0.03 | -1.44 | 0.70 | 0.04 | 0.70 |
| ^2^**TS1**_Co/Se_ | 1.28 | -0.14 | -0.05 | -0.09 | -0.31 | 0.32 | -0.12 | 0.12 |
| ^2^**IM1**_Co/Se_ | 1.19 | -0.04 | -0.10 | -0.06 | -0.48 | 0.44 | 0.12 | -0.08 |
| ^2^**TS2**_Co/Se_ | 1.23 | -0.25 | 0.04 | -0.03 | -1.12 | 0.86 | 0.15 | 0.12 |
| ^2^**IM2**_Co/Se_ | 1.64 | -0.58 | -0.01 | -0.05 | -0.58 | 1.07 | -0.63 | 0.14 |
| ^2^**IM3**_Co/Se_ | 1.15 | -0.05 | -0.03 | -0.08 | -2.18 | 1.81 | -0.72 | 1.09 |

**Table S15.** Wiberg bond index, occupancies and natural hybrid orbital composition of the calculated natural bond orbitals (NBOs) for selenocysteamine and cysteamine.

|  | Bond | Computed bond length (Å) | WBI | Occupancy | Bond Orbital | EDX% | EDY% |
| --- | --- | --- | --- | --- | --- | --- | --- |
| [Tp^Mes^FeSeCysAm]^a^ | Se−Fe | 2.496 | 0.6859 | 0.993 | 0.8875 (s^25.55%^ p^74.45%^) Se +  0.4608 (s^31.04%^ p^15.64%^ d^53.32%^) Fe | 78.76 | 21.24 |
| [Tp^Mes^FeCysAm]^b^ | S−Fe | 2.375 | 0.6425 |  |  |  |  |
| [Tp^Mes*^FeSeCysAm]^a^ | Se−Fe | 2.513 | 0.6879 | 0.992 | 0.8867 (s^26.95%^ p^73.05%^) Se +  0.4623 (s ^30.74%^ p^23.43%^ d^45.83%^) Fe | 78.63 | 21.37 |
| [Tp^Mes*^FeCysAm]^b^ | S−Fe | 2.391 | 0.6511 | 0.993 | 0.8938 (s^30.24%^ p^69.76%^) S +  0.4484 (s^27.44%^ p^22.80%^ d^49.76%^) Fe | 79.89 | 20.11 |

Table S16. Topological parameters at the bond critical points of the selenocysteamine and cysteamine. All parameters are in a.u.

|  | Bond | *ρ*_(r)_ | $\nabla_{\rho_{(r)}}^{2}$ | G_(r)_ | H_(r)_ | L_(r)_ | *ε* | V_(r)_ | \|V_(r)_\|/G_(r)_ |
| --- | --- | --- | --- | --- | --- | --- | --- | --- | --- |
| [Tp^Mes^FeSeCysAm] | Fe−Se | 0.058 | 0.028 | 0.039 | -0.011 | -0.028 | 0.209 | 0.050 | 1.282 |
| [Tp^Mes^FeCysAm] | Fe−S | 0.065 | 0.040 | 0.051 | -0.011 | -0.040 | 0.203 | 0.062 | 1.216 |
| [Tp^Mes*^FeSeCysAm] | Fe−Se | 0.056 | 0.029 | 0.039 | -0.010 | -0.029 | 0.153 | 0.049 | 1.256 |
| [Tp^Mes*^FeCysAm] | Fe−S | 0.062 | 0.040 | 0.050 | -0.010 | -0.040 | 0.160 | 0.061 | 1.220 |
| [Tp^Mes^FeO_2_SeCysAm] | Fe−O1 | 0.059 | 0.081 | 0.079 | 0.002 | -0.081 | 0.249 | 0.077 | 0.975 |
|  | Fe−O2 | 0.051 | 0.067 | 0.065 | 0.002 | -0.067 | 0.318 | 0.062 | 0.954 |
| [Tp^Mes^FeO_2_CysAm] | Fe−O1 | 0.059 | 0.081 | 0.079 | 0.002 | -0.081 | 0.244 | 0.077 | 0.975 |
|  | Fe−O2 | 0.053 | 0.070 | 0.068 | 0.003 | -0.071 | 0.251 | 0.065 | 0.956 |
| [Tp^Mes*^FeO_2_SeCysAm] | Fe−O1 | 0.047 | 0.060 | 0.058 | 0.002 | -0.060 | 0.309 | 0.056 | 0.966 |
|  | Fe−O2 | 0.058 | 0.079 | 0.076 | 0.002 | -0.079 | 0.235 | 0.074 | 0.974 |
| [Tp^Mes*^FeO_2_CysAm] | Fe−O1 | 0.050 | 0.065 | 0.062 | 0.002 | -0.065 | 0.240 | 0.060 | 0.968 |
|  | Fe−O2 | 0.057 | 0.078 | 0.076 | 0.003 | -0.078 | 0.241 | 0.073 | 0.961 |


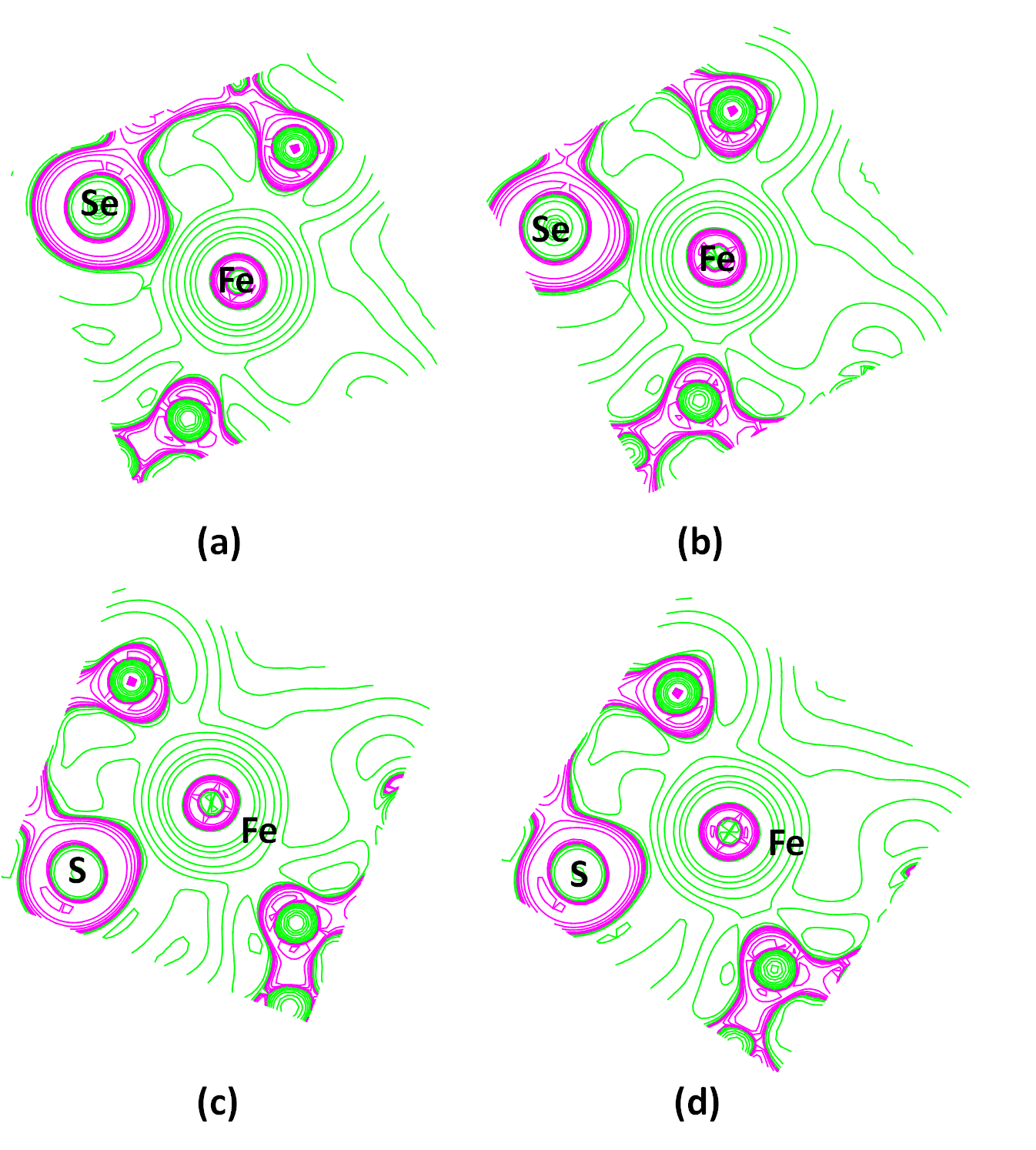


Figure S54. The contour line diagrams of the Laplacian of electron density along (a): the Fe−Se plane for [Tp^Mes^FeSeCysAm]; (b) the Fe−Se plane for [Tp^Mes^FeSeCysAm] (c) the Fe−S plane for [Tp^Mes*^FeCysAm]; (d) the Fe−S plane for [Tp^Mes*^FeCysAm]. Solid green lines indicate charge depletion [$\boldsymbol{\nabla}_{\boldsymbol{\rho}_{\boldsymbol{(r)}}}^{\boldsymbol{2}}$>0] and solid pink lines indicate charge concentration [$\boldsymbol{\nabla}_{\boldsymbol{\rho}_{\boldsymbol{(r)}}}^{\boldsymbol{2}}$<0].

Figure S55. UB3LYP/BS2 optimized geometries of ^2^RC_Co/S_ as obtained in Gaussian-09 with bond lengths in Å.

**
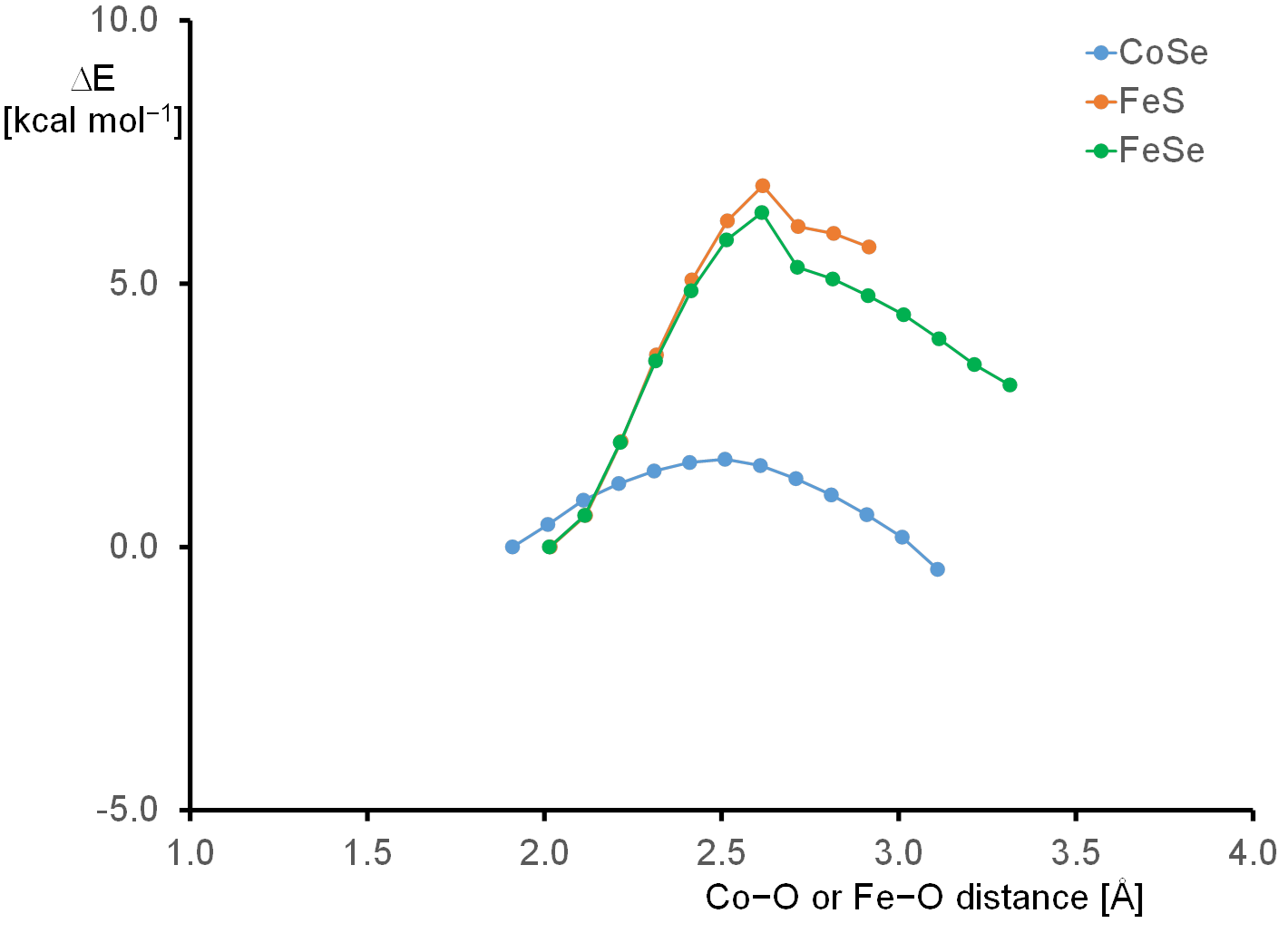
**

**Figure S56.** Constraint geometry scans from the Co/Se (in blue), Fe/S and Fe/Se metal-superoxo complexes for metal-dioxygen bond cleavage. As can be seen, the barriers are small for the Co system.

**Cartesian Coordinates of Optimized Geometries:**

**Reaction mechanism with cysteamine:**

**^5^RC_Fe/S_:**

26 -0.461211000 0.224970000 0.067043000

16 1.030407000 -1.374391000 -0.782418000

7 -1.851771000 2.006022000 0.697666000

7 -3.102051000 1.633489000 1.088950000

6 -3.771162000 2.689657000 1.591604000

6 -2.942895000 3.795252000 1.528110000

6 -1.745464000 3.325885000 0.958771000

1 -4.782882000 2.580272000 1.951250000

1 -3.164458000 4.804534000 1.838445000

7 -1.452545000 -0.977818000 1.614108000

7 -2.791992000 -0.783650000 1.787252000

6 -3.237723000 -1.536544000 2.808771000

6 -2.169898000 -2.249159000 3.324711000

6 -1.061444000 -1.872172000 2.549960000

1 -4.278087000 -1.511787000 3.094678000

1 -2.178391000 -2.948776000 4.145661000

7 -2.313723000 -0.260796000 -1.231648000

7 -3.505115000 -0.219733000 -0.567958000

6 -4.511278000 -0.582792000 -1.385703000

6 -3.977368000 -0.868272000 -2.628334000

6 -2.594402000 -0.656152000 -2.492483000

1 -5.528638000 -0.610873000 -1.026613000

1 -4.501633000 -1.185956000 -3.516039000

5 -3.616548000 0.190709000 0.914878000

1 -4.763381000 0.136580000 1.255364000

6 -0.551738000 4.152953000 0.629494000

6 -0.451724000 4.754010000 -0.641092000

6 0.666042000 5.544664000 -0.935052000

6 1.669797000 5.777165000 0.004560000

6 1.520279000 5.216245000 1.276360000

6 0.426907000 4.416270000 1.611833000

1 0.743889000 5.996627000 -1.920525000

1 2.278659000 5.407421000 2.031182000

6 0.325534000 -2.384657000 2.712682000

6 1.210790000 -1.748765000 3.604780000

6 2.490411000 -2.282620000 3.778675000

6 2.909664000 -3.433700000 3.108717000

6 1.999525000 -4.068202000 2.261417000

6 0.711350000 -3.568649000 2.054958000

1 3.175594000 -1.786468000 4.461144000

1 2.297919000 -4.977810000 1.746455000

6 -1.592646000 -0.825923000 -3.579575000

6 -1.039109000 -2.101158000 -3.828244000

6 -0.152235000 -2.256924000 -4.893066000

6 0.197227000 -1.192102000 -5.731216000

6 -0.389867000 0.048070000 -5.490360000

6 -1.292258000 0.247965000 -4.437163000

1 0.277867000 -3.238145000 -5.076749000

1 -0.154371000 0.884400000 -6.143651000

6 1.848593000 -0.381278000 -2.103673000

6 1.805479000 1.105485000 -1.794474000

7 0.413928000 1.512337000 -1.473273000

1 2.885356000 -0.713330000 -2.183866000

1 1.354159000 -0.603192000 -3.051515000

1 2.409224000 1.324445000 -0.915342000

1 -0.160534000 1.468442000 -2.310995000

1 0.398009000 2.479338000 -1.155793000

6 0.794065000 -0.528058000 4.389540000

1 -0.054666000 -0.746428000 5.045263000

1 1.614311000 -0.169873000 5.014736000

1 0.493316000 0.284565000 3.725648000

6 4.310992000 -3.966791000 3.282887000

1 4.715812000 -3.719312000 4.267203000

1 4.346227000 -5.052603000 3.164489000

1 4.989584000 -3.538075000 2.537050000

6 -0.248658000 -4.321707000 1.164625000

1 0.262395000 -5.125244000 0.631037000

1 -1.059865000 -4.771881000 1.746147000

1 -0.713568000 -3.669626000 0.422933000

6 -1.416329000 -3.294488000 -2.984831000

1 -2.480587000 -3.529835000 -3.082680000

1 -0.850349000 -4.178614000 -3.284107000

1 -1.221001000 -3.110911000 -1.926939000

6 1.185379000 -1.388346000 -6.854750000

1 2.206687000 -1.482428000 -6.470481000

1 0.972776000 -2.299666000 -7.420485000

1 1.172967000 -0.548042000 -7.552101000

6 -1.969819000 1.594360000 -4.306017000

1 -1.313974000 2.402692000 -4.637734000

1 -2.867982000 1.633880000 -4.932195000

1 -2.294562000 1.809421000 -3.287622000

6 2.887659000 6.600135000 -0.338031000

1 3.749694000 5.957669000 -0.548254000

1 2.719197000 7.220059000 -1.221371000

1 3.173115000 7.257247000 0.487654000

6 0.296146000 3.872084000 3.012952000

1 -0.586786000 4.279027000 3.515686000

1 0.196790000 2.785915000 3.002270000

1 1.170312000 4.130455000 3.613669000

6 -1.553442000 4.625378000 -1.669120000

1 -1.180744000 4.831865000 -2.674412000

1 -2.014586000 3.637107000 -1.669928000

1 -2.355370000 5.343246000 -1.465174000

1 2.190394000 1.683350000 -2.642740000

8 0.704347000 1.056935000 1.486531000

8 1.998638000 1.206211000 1.398329000

**^5^TS1_Fe/S_:**

26 -0.545128000 0.344091000 0.055509000

16 1.396341000 -1.256426000 -0.676171000

7 -1.864528000 2.028914000 0.718564000

7 -3.124069000 1.652411000 1.076607000

6 -3.822845000 2.718197000 1.513994000

6 -3.007002000 3.833525000 1.437027000

6 -1.784870000 3.358269000 0.928608000

1 -4.844953000 2.609653000 1.843397000

1 -3.252570000 4.849833000 1.703396000

7 -1.450549000 -0.930867000 1.653086000

7 -2.794103000 -0.754081000 1.812761000

6 -3.252653000 -1.539607000 2.806382000

6 -2.187357000 -2.256084000 3.319057000

6 -1.070584000 -1.846896000 2.567685000

1 -4.298017000 -1.532541000 3.074910000

1 -2.200952000 -2.975409000 4.123031000

7 -2.333508000 -0.286205000 -1.235839000

7 -3.517184000 -0.242124000 -0.559403000

6 -4.529784000 -0.626704000 -1.361746000

6 -4.007533000 -0.926937000 -2.606536000

6 -2.624626000 -0.699163000 -2.485638000

1 -5.543538000 -0.657750000 -0.992614000

1 -4.540068000 -1.262262000 -3.482952000

5 -3.620900000 0.197132000 0.921442000

1 -4.769405000 0.142646000 1.260287000

6 -0.581621000 4.171581000 0.603567000

6 -0.509798000 4.835024000 -0.637675000

6 0.616847000 5.612788000 -0.929040000

6 1.659736000 5.769263000 -0.015574000

6 1.545995000 5.135420000 1.224736000

6 0.445415000 4.342023000 1.554265000

1 0.671513000 6.116896000 -1.890447000

1 2.337002000 5.263291000 1.959289000

6 0.316393000 -2.355291000 2.747136000

6 1.197772000 -1.701962000 3.630431000

6 2.462856000 -2.250025000 3.853466000

6 2.880387000 -3.425898000 3.226297000

6 1.983554000 -4.065708000 2.369654000

6 0.704901000 -3.556406000 2.124202000

1 3.141117000 -1.742260000 4.534687000

1 2.282918000 -4.989252000 1.880508000

6 -1.613475000 -0.856680000 -3.566257000

6 -0.925476000 -2.079530000 -3.720257000

6 -0.016311000 -2.217444000 -4.769528000

6 0.230634000 -1.183032000 -5.678543000

6 -0.480530000 0.005810000 -5.524800000

6 -1.411459000 0.183972000 -4.493054000

1 0.513978000 -3.159606000 -4.882649000

1 -0.320015000 0.816334000 -6.230885000

6 2.027491000 -0.229656000 -2.058469000

6 1.748678000 1.258429000 -1.887652000

7 0.312801000 1.507530000 -1.625792000

1 3.101879000 -0.393381000 -2.157633000

1 1.552497000 -0.614156000 -2.966198000

1 2.310510000 1.647276000 -1.040046000

1 -0.222911000 1.312553000 -2.468087000

1 0.177804000 2.494032000 -1.418100000

6 0.793949000 -0.432118000 4.337963000

1 -0.133114000 -0.561197000 4.904129000

1 1.570671000 -0.107790000 5.033452000

1 0.624337000 0.373603000 3.619918000

6 4.268864000 -3.973919000 3.451284000

1 4.617639000 -3.780635000 4.468974000

1 4.308846000 -5.052077000 3.278525000

1 4.990494000 -3.509118000 2.770253000

6 -0.241360000 -4.319840000 1.227381000

1 0.285546000 -5.103959000 0.680138000

1 -1.038397000 -4.798500000 1.805914000

1 -0.728965000 -3.668983000 0.499234000

6 -1.176456000 -3.238844000 -2.786807000

1 -2.236022000 -3.508856000 -2.761168000

1 -0.611538000 -4.119788000 -3.096871000

1 -0.883865000 -2.996904000 -1.762762000

6 1.236004000 -1.360084000 -6.790192000

1 1.237786000 -0.505903000 -7.470362000

1 2.250380000 -1.471230000 -6.393646000

1 1.025183000 -2.257470000 -7.379412000

6 -2.213670000 1.466020000 -4.441502000

1 -1.690130000 2.277731000 -4.950863000

1 -3.179549000 1.338141000 -4.941944000

1 -2.435266000 1.786680000 -3.422257000

6 2.880724000 6.589200000 -0.354858000

1 2.684509000 7.279558000 -1.178117000

1 3.223203000 7.173909000 0.503033000

1 3.714790000 5.946828000 -0.657750000

6 0.365064000 3.695503000 2.914872000

1 -0.579422000 3.925851000 3.415563000

1 0.434618000 2.609210000 2.832755000

1 1.179519000 4.036371000 3.557135000

6 -1.642635000 4.769156000 -1.638179000

1 -1.312709000 5.096779000 -2.626012000

1 -2.060450000 3.764911000 -1.732913000

1 -2.468934000 5.420757000 -1.335547000

1 2.080847000 1.792306000 -2.785810000

8 1.107689000 0.965893000 1.190502000

8 2.127708000 0.156347000 0.995877000

**^5^IM1_Fe/S_:**

26 -0.761680000 0.421180000 0.168374000

16 1.644618000 -0.958060000 -0.333169000

7 -2.084366000 2.068652000 0.811839000

7 -3.341876000 1.693413000 1.179375000

6 -4.042793000 2.764955000 1.598411000

6 -3.230006000 3.881484000 1.501012000

6 -2.007669000 3.401151000 0.997834000

1 -5.064462000 2.660093000 1.930532000

1 -3.477889000 4.901194000 1.751892000

7 -1.694984000 -0.956831000 1.713639000

7 -3.021731000 -0.719527000 1.923756000

6 -3.478233000 -1.476592000 2.941918000

6 -2.427565000 -2.237962000 3.417860000

6 -1.323982000 -1.884848000 2.617137000

1 -4.511821000 -1.422405000 3.248430000

1 -2.443226000 -2.954599000 4.224378000

7 -2.536632000 -0.243413000 -1.113403000

7 -3.725700000 -0.209232000 -0.448906000

6 -4.726158000 -0.604333000 -1.261556000

6 -4.188037000 -0.902465000 -2.500684000

6 -2.808386000 -0.660788000 -2.365496000

1 -5.743717000 -0.643542000 -0.903752000

1 -4.709274000 -1.247038000 -3.380309000

5 -3.839759000 0.236081000 1.031773000

1 -4.991168000 0.188378000 1.363293000

6 -0.805757000 4.208873000 0.655562000

6 -0.749861000 4.871934000 -0.586286000

6 0.373342000 5.648414000 -0.893106000

6 1.429023000 5.802920000 0.006073000

6 1.333941000 5.164544000 1.245488000

6 0.238453000 4.369449000 1.589053000

1 0.415785000 6.152689000 -1.855142000

1 2.137336000 5.287533000 1.967513000

6 0.035344000 -2.483714000 2.717168000

6 1.019445000 -1.894108000 3.533819000

6 2.252351000 -2.536513000 3.678374000

6 2.540174000 -3.741487000 3.035359000

6 1.543814000 -4.316071000 2.243643000

6 0.292607000 -3.714874000 2.081431000

1 3.009500000 -2.077721000 4.309237000

1 1.740268000 -5.262227000 1.745131000

6 -1.771964000 -0.817481000 -3.421257000

6 -0.930171000 -1.948610000 -3.429841000

6 0.012817000 -2.086043000 -4.451326000

6 0.146051000 -1.138519000 -5.469479000

6 -0.712028000 -0.037899000 -5.454689000

6 -1.675534000 0.137262000 -4.455305000

1 0.655028000 -2.962928000 -4.455828000

1 -0.639109000 0.703533000 -6.246130000

6 2.125146000 0.084177000 -1.762821000

6 1.529946000 1.485944000 -1.733377000

7 0.054529000 1.467618000 -1.646933000

1 3.214121000 0.146677000 -1.802651000

1 1.788914000 -0.485087000 -2.636479000

1 1.902667000 2.020162000 -0.860461000

1 -0.338271000 1.091605000 -2.507239000

1 -0.276421000 2.426013000 -1.576517000

6 0.768030000 -0.585656000 4.239669000

1 -0.175569000 -0.597123000 4.792657000

1 1.570913000 -0.361884000 4.945770000

1 0.713741000 0.225704000 3.508634000

6 3.895309000 -4.392807000 3.172024000

1 4.370028000 -4.137118000 4.122393000

1 3.826932000 -5.481828000 3.109482000

1 4.571747000 -4.065549000 2.374415000

6 -0.768526000 -4.420645000 1.268915000

1 -0.326895000 -5.192884000 0.635559000

1 -1.502648000 -4.909212000 1.918662000

1 -1.324531000 -3.732625000 0.630501000

6 -1.046770000 -3.016674000 -2.370197000

1 -2.077122000 -3.368148000 -2.267259000

1 -0.418603000 -3.876517000 -2.610006000

1 -0.741710000 -2.642224000 -1.390720000

6 1.195148000 -1.295227000 -6.542964000

1 2.150848000 -0.862533000 -6.226988000

1 1.379283000 -2.347475000 -6.772648000

1 0.902450000 -0.792299000 -7.467469000

6 -2.605511000 1.327895000 -4.525632000

1 -2.234659000 2.070173000 -5.235007000

1 -3.605589000 1.029326000 -4.854925000

1 -2.731595000 1.815870000 -3.556369000

6 2.645671000 6.622526000 -0.350184000

1 3.478555000 5.979678000 -0.655679000

1 2.441713000 7.306932000 -1.176721000

1 2.994407000 7.213756000 0.500644000

6 0.193517000 3.693166000 2.936585000

1 -0.773497000 3.829886000 3.427927000

1 0.360441000 2.618898000 2.823950000

1 0.968217000 4.088628000 3.597133000

6 -1.893997000 4.803924000 -1.574403000

1 -1.576189000 5.135732000 -2.564910000

1 -2.310129000 3.798215000 -1.668256000

1 -2.720763000 5.450316000 -1.262126000

1 1.868967000 2.023712000 -2.627085000

8 0.834353000 0.924289000 1.259025000

8 1.995167000 0.046953000 0.954700000

**^5^TS2_Fe/S_:**

26 -0.750457000 0.425159000 0.207572000

16 1.783493000 -0.910347000 -0.443411000

7 -2.082689000 2.075126000 0.817559000

7 -3.345949000 1.701257000 1.168470000

6 -4.044292000 2.773718000 1.589930000

6 -3.226244000 3.886731000 1.507055000

6 -2.000245000 3.405970000 1.014055000

1 -5.068748000 2.670274000 1.913407000

1 -3.472185000 4.906196000 1.760382000

7 -1.723628000 -0.961557000 1.697838000

7 -3.046149000 -0.711992000 1.916422000

6 -3.494322000 -1.447849000 2.952599000

6 -2.442631000 -2.208954000 3.428485000

6 -1.345149000 -1.876609000 2.612112000

1 -4.523630000 -1.381094000 3.270710000

1 -2.454649000 -2.914261000 4.244861000

7 -2.527740000 -0.252811000 -1.100141000

7 -3.727243000 -0.203932000 -0.454863000

6 -4.719179000 -0.596049000 -1.278947000

6 -4.164877000 -0.908220000 -2.506841000

6 -2.785151000 -0.677954000 -2.353238000

1 -5.742088000 -0.623879000 -0.935699000

1 -4.675100000 -1.255999000 -3.391568000

5 -3.855710000 0.246606000 1.020075000

1 -5.008624000 0.208919000 1.344681000

6 -0.796656000 4.216186000 0.684748000

6 -0.740145000 4.897608000 -0.546774000

6 0.382228000 5.681131000 -0.839358000

6 1.435497000 5.824049000 0.064020000

6 1.339596000 5.166294000 1.293593000

6 0.245172000 4.364666000 1.623257000

1 0.425628000 6.199503000 -1.793815000

1 2.141917000 5.278869000 2.018420000

6 0.011579000 -2.482417000 2.700686000

6 1.008298000 -1.890759000 3.500214000

6 2.240716000 -2.536143000 3.631107000

6 2.516563000 -3.744740000 2.989332000

6 1.507447000 -4.321172000 2.215638000

6 0.255127000 -3.717889000 2.068733000

1 3.008610000 -2.075168000 4.247140000

1 1.694205000 -5.270484000 1.719494000

6 -1.741338000 -0.852829000 -3.398431000

6 -0.900633000 -1.982109000 -3.385435000

6 0.047021000 -2.136008000 -4.403164000

6 0.183174000 -1.206885000 -5.434876000

6 -0.673630000 -0.102712000 -5.438898000

6 -1.640660000 0.087123000 -4.448892000

1 0.690587000 -3.011499000 -4.389287000

1 -0.594062000 0.628065000 -6.239872000

6 2.125173000 0.172397000 -1.886136000

6 1.485635000 1.548315000 -1.763534000

7 0.013430000 1.469793000 -1.631559000

1 3.206478000 0.279573000 -1.994828000

1 1.749496000 -0.378721000 -2.754296000

1 1.866640000 2.048254000 -0.873877000

1 -0.389796000 1.079331000 -2.480431000

1 -0.352196000 2.415118000 -1.549866000

6 0.771077000 -0.575602000 4.197711000

1 -0.160411000 -0.581553000 4.771191000

1 1.588665000 -0.343689000 4.883969000

1 0.700662000 0.226086000 3.457635000

6 3.873046000 -4.396630000 3.108010000

1 4.351087000 -4.159253000 4.061532000

1 3.806217000 -5.484196000 3.023681000

1 4.545894000 -4.051524000 2.314929000

6 -0.820667000 -4.427419000 1.278909000

1 -0.390869000 -5.205213000 0.644277000

1 -1.544363000 -4.910359000 1.944451000

1 -1.386121000 -3.743874000 0.644042000

6 -1.011012000 -3.030908000 -2.306223000

1 -2.051601000 -3.305419000 -2.116336000

1 -0.466058000 -3.935332000 -2.583255000

1 -0.598349000 -2.673486000 -1.359738000

6 1.214000000 -1.385538000 -6.522817000

1 1.916369000 -0.546588000 -6.547346000

1 1.792444000 -2.300435000 -6.380688000

1 0.745214000 -1.436840000 -7.510359000

6 -2.570071000 1.276910000 -4.540850000

1 -2.198005000 2.006118000 -5.262999000

1 -3.569818000 0.972902000 -4.865911000

1 -2.697759000 1.782727000 -3.580912000

6 2.651181000 6.651379000 -0.276969000

1 3.489640000 6.014103000 -0.578675000

1 2.450991000 7.339580000 -1.101225000

1 2.989670000 7.239298000 0.580303000

6 0.196137000 3.667702000 2.959466000

1 -0.756783000 3.834384000 3.469567000

1 0.315923000 2.590084000 2.820839000

1 0.996472000 4.020126000 3.613637000

6 -1.881684000 4.845277000 -1.539181000

1 -1.552724000 5.162218000 -2.530944000

1 -2.320596000 3.848847000 -1.627428000

1 -2.694973000 5.512952000 -1.236025000

1 1.769580000 2.146186000 -2.637568000

8 0.665396000 0.919732000 1.216945000

8 2.180398000 0.010218000 0.806055000

**^5^IM2_Fe/S_:**

26 -0.865540000 0.265015000 0.240250000

16 2.285249000 -1.214605000 -1.091570000

7 -2.205014000 2.104588000 0.810479000

7 -3.458959000 1.746998000 1.193493000

6 -4.148753000 2.828530000 1.616063000

6 -3.324189000 3.933600000 1.504848000

6 -2.110497000 3.432722000 0.989899000

1 -5.168189000 2.735584000 1.959004000

1 -3.557040000 4.957135000 1.755325000

7 -1.789783000 -0.898201000 1.702210000

7 -3.108717000 -0.654376000 1.944630000

6 -3.532043000 -1.394498000 2.988006000

6 -2.466005000 -2.146016000 3.445263000

6 -1.384896000 -1.811163000 2.608607000

1 -4.556693000 -1.336821000 3.322066000

1 -2.457211000 -2.850734000 4.262001000

7 -2.549593000 -0.241066000 -1.019980000

7 -3.772007000 -0.166199000 -0.422395000

6 -4.736800000 -0.500348000 -1.298800000

6 -4.140750000 -0.801323000 -2.510910000

6 -2.763153000 -0.625504000 -2.296288000

1 -5.773995000 -0.499793000 -1.000615000

1 -4.623403000 -1.111888000 -3.424206000

5 -3.940982000 0.289722000 1.052247000

1 -5.096371000 0.216372000 1.361036000

6 -0.889951000 4.209207000 0.639724000

6 -0.852524000 4.945330000 -0.562099000

6 0.293052000 5.687811000 -0.869850000

6 1.394241000 5.733190000 -0.013651000

6 1.324442000 5.015343000 1.182946000

6 0.205267000 4.254049000 1.527354000

1 0.318779000 6.249952000 -1.799903000

1 2.165498000 5.048142000 1.870818000

6 -0.025396000 -2.412311000 2.674860000

6 0.983080000 -1.814106000 3.457338000

6 2.229994000 -2.434814000 3.542030000

6 2.508401000 -3.634095000 2.879881000

6 1.484287000 -4.226290000 2.142087000

6 0.215901000 -3.643365000 2.037735000

1 3.008252000 -1.965684000 4.138731000

1 1.669659000 -5.171418000 1.637706000

6 -1.686403000 -0.822734000 -3.303391000

6 -0.879875000 -1.980204000 -3.267548000

6 0.076121000 -2.172484000 -4.266838000

6 0.262402000 -1.251502000 -5.301926000

6 -0.553371000 -0.120090000 -5.324953000

6 -1.533869000 0.108131000 -4.352413000

1 0.688501000 -3.069857000 -4.238965000

1 -0.434649000 0.603572000 -6.126895000

6 2.091178000 0.286459000 -2.131737000

6 1.441972000 1.490787000 -1.458696000

7 -0.030896000 1.363590000 -1.329141000

1 3.105426000 0.541791000 -2.446466000

1 1.535550000 -0.035009000 -3.017329000

1 1.843102000 1.638624000 -0.457116000

1 -0.429177000 1.097888000 -2.227510000

1 -0.400364000 2.288677000 -1.121438000

6 0.734745000 -0.519574000 4.188112000

1 -0.148651000 -0.579898000 4.830981000

1 1.588866000 -0.251779000 4.813969000

1 0.565286000 0.281880000 3.465740000

6 3.881901000 -4.255926000 2.950775000

1 4.312469000 -4.167407000 3.951886000

1 3.857935000 -5.315465000 2.685579000

1 4.573044000 -3.761083000 2.259717000

6 -0.874068000 -4.381836000 1.294655000

1 -0.450406000 -5.102153000 0.591645000

1 -1.509964000 -4.941098000 1.989930000

1 -1.530537000 -3.710439000 0.740296000

6 -1.047211000 -3.017027000 -2.184500000

1 -2.097806000 -3.281234000 -2.039279000

1 -0.497901000 -3.928031000 -2.427802000

1 -0.676174000 -2.656825000 -1.221992000

6 1.325293000 -1.471582000 -6.350097000

1 2.313833000 -1.185252000 -5.974854000

1 1.386818000 -2.522811000 -6.642742000

1 1.133092000 -0.880127000 -7.247676000

6 -2.419747000 1.328033000 -4.474165000

1 -1.977027000 2.063750000 -5.148259000

1 -3.401798000 1.063356000 -4.878510000

1 -2.600928000 1.813089000 -3.512556000

6 2.632709000 6.516841000 -0.375000000

1 3.389498000 5.869565000 -0.831925000

1 2.411464000 7.312615000 -1.089929000

1 3.091062000 6.971534000 0.506839000

6 0.184336000 3.490268000 2.827551000

1 -0.727667000 3.689495000 3.397620000

1 0.222249000 2.415181000 2.633932000

1 1.039515000 3.757595000 3.451909000

6 -2.031017000 4.975167000 -1.509883000

1 -1.747351000 5.407559000 -2.471443000

1 -2.442769000 3.979613000 -1.692970000

1 -2.849734000 5.578966000 -1.106074000

1 1.676339000 2.374848000 -2.061381000

8 0.499885000 0.496055000 1.123141000

8 3.544618000 -0.974430000 -0.252847000

**^5^IM3’_Fe/S_:**

26 -0.447831000 0.286366000 -0.338330000

16 2.563695000 -0.713788000 -0.818544000

7 -1.633570000 1.938511000 0.487243000

7 -2.891938000 1.589611000 0.879716000

6 -3.524773000 2.662921000 1.391553000

6 -2.668045000 3.748645000 1.329588000

6 -1.487624000 3.252408000 0.748638000

1 -4.536461000 2.581092000 1.758529000

1 -2.861328000 4.758330000 1.656171000

7 -1.271677000 -0.930679000 1.404558000

7 -2.624422000 -0.833048000 1.543250000

6 -3.052293000 -1.622631000 2.550178000

6 -1.952680000 -2.260130000 3.092623000

6 -0.850294000 -1.793043000 2.348686000

1 -4.099234000 -1.667940000 2.809270000

1 -1.933787000 -2.958686000 3.914838000

7 -2.231059000 -0.241885000 -1.503142000

7 -3.399220000 -0.238523000 -0.801949000

6 -4.428988000 -0.539037000 -1.617963000

6 -3.929116000 -0.745914000 -2.892262000

6 -2.541649000 -0.543312000 -2.778336000

1 -5.438576000 -0.581210000 -1.238492000

1 -4.481000000 -1.006305000 -3.781967000

5 -3.453911000 0.150386000 0.697295000

1 -4.594789000 0.130089000 1.063086000

6 -0.257712000 4.016258000 0.410514000

6 -0.303449000 4.967544000 -0.634691000

6 0.846348000 5.701357000 -0.934435000

6 2.038350000 5.532601000 -0.224625000

6 2.055025000 4.600725000 0.812308000

6 0.931782000 3.835246000 1.143349000

1 0.808501000 6.428389000 -1.741640000

1 2.967277000 4.461695000 1.386394000

6 0.573887000 -2.149924000 2.592620000

6 1.299874000 -1.452510000 3.577239000

6 2.620512000 -1.827083000 3.841685000

6 3.237063000 -2.881497000 3.167143000

6 2.487646000 -3.577801000 2.216318000

6 1.167119000 -3.233421000 1.917524000

1 3.179834000 -1.279525000 4.596172000

1 2.942879000 -4.412719000 1.689402000

6 -1.527819000 -0.614732000 -3.864479000

6 -0.707735000 -1.753878000 -4.001377000

6 0.216256000 -1.800528000 -5.047542000

6 0.354116000 -0.754143000 -5.963370000

6 -0.478506000 0.356085000 -5.818813000

6 -1.424777000 0.442820000 -4.791242000

1 0.844402000 -2.681715000 -5.151233000

1 -0.398480000 1.176106000 -6.527640000

6 2.549630000 0.368598000 -2.331524000

6 1.904176000 1.721557000 -2.089343000

7 0.430900000 1.625769000 -1.929903000

1 3.601900000 0.498843000 -2.597495000

1 2.060987000 -0.204498000 -3.123690000

1 2.313085000 2.158049000 -1.176599000

1 0.023803000 1.305348000 -2.806239000

1 0.067918000 2.563399000 -1.778468000

6 0.671278000 -0.335621000 4.377978000

1 -0.025297000 -0.728189000 5.126473000

1 1.431980000 0.240653000 4.908465000

1 0.102933000 0.351256000 3.748977000

6 4.677463000 -3.242567000 3.438024000

1 4.965603000 -3.007022000 4.465525000

1 4.864474000 -4.305909000 3.268943000

1 5.352223000 -2.686517000 2.777651000

6 0.406068000 -4.015812000 0.876529000

1 0.962251000 -4.903997000 0.569026000

1 -0.571484000 -4.339517000 1.244435000

1 0.234335000 -3.399088000 -0.008059000

6 -0.806250000 -2.911134000 -3.038281000

1 -1.844953000 -3.184795000 -2.836740000

1 -0.293256000 -3.790806000 -3.432312000

1 -0.344681000 -2.653617000 -2.082024000

6 1.382496000 -0.823621000 -7.065886000

1 2.390478000 -0.640547000 -6.678222000

1 1.396920000 -1.809537000 -7.538217000

1 1.190406000 -0.080056000 -7.842358000

6 -2.326221000 1.655366000 -4.716726000

1 -1.947078000 2.461229000 -5.348083000

1 -3.338504000 1.418793000 -5.059173000

1 -2.424159000 2.038546000 -3.698223000

6 3.273594000 6.320632000 -0.586203000

1 3.809659000 5.851340000 -1.418499000

1 3.024689000 7.338293000 -0.897601000

1 3.969457000 6.382858000 0.253292000

6 1.032919000 2.839415000 2.273173000

1 0.085224000 2.721207000 2.800681000

1 1.333284000 1.854018000 1.903204000

1 1.788743000 3.152762000 2.996712000

6 -1.566013000 5.221161000 -1.428957000

1 -1.343637000 5.778029000 -2.341283000

1 -2.072156000 4.294889000 -1.711229000

1 -2.289274000 5.808716000 -0.855345000

1 2.160278000 2.391870000 -2.918326000

8 1.013568000 -1.019596000 -0.679244000

8 2.999766000 0.243985000 0.280792000

**^5^IM3_Fe/S_:**

26 0.110118000 0.857497000 0.364298000

16 2.230658000 -0.713958000 -0.566466000

7 -0.195991000 2.733159000 1.534790000

7 -1.342445000 2.744522000 2.271874000

6 -1.444388000 3.902357000 2.954299000

6 -0.340518000 4.681718000 2.655937000

6 0.419668000 3.910442000 1.757307000

1 -2.289764000 4.089702000 3.598781000

1 -0.109208000 5.667183000 3.029368000

7 -0.566786000 -0.250669000 2.193136000

7 -1.679594000 0.284122000 2.773384000

6 -2.042101000 -0.444312000 3.847416000

6 -1.146554000 -1.488872000 3.983699000

6 -0.231732000 -1.332108000 2.925395000

1 -2.904257000 -0.167995000 4.435117000

1 -1.142430000 -2.260897000 4.737493000

7 -2.028929000 1.089143000 -0.233942000

7 -2.869977000 1.358251000 0.803286000

6 -4.139877000 1.450758000 0.361810000

6 -4.139491000 1.245264000 -1.006851000

6 -2.791259000 1.024311000 -1.343015000

1 -4.948045000 1.658292000 1.046575000

1 -4.986431000 1.254674000 -1.675249000

5 -2.351553000 1.569885000 2.245951000

1 -3.280511000 1.841245000 2.953782000

6 1.695411000 4.308361000 1.103866000

6 1.670805000 5.245190000 0.049061000

6 2.876587000 5.644426000 -0.534214000

6 4.107927000 5.155951000 -0.091625000

6 4.109149000 4.248950000 0.969068000

6 2.927098000 3.813859000 1.576392000

1 2.852167000 6.362370000 -1.350256000

1 5.057334000 3.869146000 1.341012000

6 0.937749000 -2.214933000 2.662616000

6 2.137656000 -2.007393000 3.370650000

6 3.204791000 -2.886508000 3.169981000

6 3.112946000 -3.972896000 2.297131000

6 1.905274000 -4.173924000 1.627199000

6 0.813727000 -3.316178000 1.795268000

1 4.131461000 -2.719666000 3.713685000

1 1.805273000 -5.022856000 0.955473000

6 -2.244259000 0.798721000 -2.708748000

6 -1.993189000 -0.507055000 -3.175005000

6 -1.520326000 -0.680913000 -4.478826000

6 -1.288912000 0.398190000 -5.334250000

6 -1.565022000 1.681776000 -4.858999000

6 -2.048768000 1.902242000 -3.565510000

1 -1.330962000 -1.690169000 -4.835488000

1 -1.410428000 2.535713000 -5.513582000

6 2.654163000 0.362428000 -2.023267000

6 2.260051000 1.813948000 -1.804160000

7 0.823973000 1.969568000 -1.463606000

1 3.734534000 0.288223000 -2.172160000

1 2.154936000 -0.095230000 -2.880693000

1 2.832976000 2.226333000 -0.974485000

1 0.245266000 1.650572000 -2.238544000

1 0.628088000 2.960852000 -1.357244000

6 2.282321000 -0.871088000 4.355006000

1 1.645250000 -1.019264000 5.232761000

1 3.313050000 -0.787264000 4.705247000

1 1.995773000 0.085974000 3.914666000

6 4.291196000 -4.889355000 2.071689000

1 4.903902000 -4.985900000 2.971613000

1 3.970751000 -5.889927000 1.771381000

1 4.942614000 -4.506338000 1.278075000

6 -0.467880000 -3.579933000 1.044358000

1 -0.435836000 -4.549882000 0.543462000

1 -1.337414000 -3.569672000 1.707243000

1 -0.624899000 -2.810903000 0.285162000

6 -2.217082000 -1.711234000 -2.294983000

1 -3.157586000 -1.642445000 -1.742914000

1 -2.235938000 -2.628730000 -2.887086000

1 -1.411355000 -1.799947000 -1.562865000

6 -0.741439000 0.183906000 -6.724567000

1 0.353835000 0.154788000 -6.716775000

1 -1.084683000 -0.762387000 -7.149689000

1 -1.038619000 0.987620000 -7.402285000

6 -2.382100000 3.311475000 -3.129795000

1 -1.949238000 4.044324000 -3.813475000

1 -3.464456000 3.474429000 -3.115355000

1 -2.023072000 3.530992000 -2.121964000

6 5.395626000 5.582876000 -0.753685000

1 5.621806000 4.951912000 -1.620527000

1 5.341244000 6.613780000 -1.112206000

1 6.243464000 5.506412000 -0.069013000

6 2.996341000 2.823904000 2.712598000

1 2.279342000 3.060072000 3.502437000

1 2.772242000 1.816716000 2.352991000

1 3.995482000 2.807437000 3.153584000

6 0.376574000 5.852635000 -0.445747000

1 0.512153000 6.312482000 -1.426708000

1 -0.429042000 5.118615000 -0.521007000

1 0.019118000 6.632316000 0.234566000

1 2.515844000 2.386971000 -2.703245000

8 0.693177000 -0.918722000 -0.780359000

8 2.293124000 0.306215000 0.609503000

**^3^RC_Fe/S_:**

26 -0.642508000 0.271901000 0.043317000

16 0.644502000 -1.378668000 -0.703540000

7 -1.861000000 1.916755000 0.695834000

7 -3.120966000 1.591896000 1.089152000

6 -3.756257000 2.670638000 1.579304000

6 -2.890841000 3.747555000 1.507942000

6 -1.709083000 3.239122000 0.943003000

1 -4.772131000 2.596156000 1.935990000

1 -3.077971000 4.766488000 1.808758000

7 -1.463133000 -0.933786000 1.547818000

7 -2.805242000 -0.806362000 1.746483000

6 -3.211042000 -1.589189000 2.762891000

6 -2.107599000 -2.256866000 3.253961000

6 -1.022836000 -1.821718000 2.470212000

1 -4.248442000 -1.609855000 3.059497000

1 -2.069496000 -2.964300000 4.067391000

7 -2.316274000 -0.240147000 -1.215549000

7 -3.522349000 -0.224119000 -0.579906000

6 -4.509614000 -0.569766000 -1.424926000

6 -3.947825000 -0.820927000 -2.661432000

6 -2.569036000 -0.605448000 -2.495422000

1 -5.533339000 -0.609882000 -1.085873000

1 -4.449307000 -1.120207000 -3.568382000

5 -3.651767000 0.158352000 0.905769000

1 -4.798765000 0.099879000 1.244177000

6 -0.504822000 4.049867000 0.609620000

6 -0.431687000 4.695895000 -0.641327000

6 0.688117000 5.482030000 -0.937912000

6 1.719259000 5.669997000 -0.018303000

6 1.594026000 5.070632000 1.238467000

6 0.498940000 4.274158000 1.576391000

1 0.745051000 5.967300000 -1.908799000

1 2.371473000 5.230934000 1.980879000

6 0.373756000 -2.297242000 2.669663000

6 1.225190000 -1.616218000 3.560584000

6 2.507585000 -2.122342000 3.791610000

6 2.959833000 -3.295667000 3.186146000

6 2.075776000 -3.985052000 2.353560000

6 0.786484000 -3.514529000 2.093208000

1 3.166733000 -1.586829000 4.470231000

1 2.395506000 -4.917604000 1.895332000

6 -1.570660000 -0.759819000 -3.589066000

6 -1.058063000 -2.043414000 -3.887136000

6 -0.177264000 -2.187053000 -4.957760000

6 0.202641000 -1.104328000 -5.760040000

6 -0.354594000 0.140222000 -5.480581000

6 -1.251668000 0.329130000 -4.420007000

1 0.223266000 -3.173538000 -5.176743000

1 -0.103412000 0.990388000 -6.109898000

6 1.676337000 -0.492083000 -1.947535000

6 1.630208000 1.003163000 -1.699440000

7 0.233032000 1.406750000 -1.373982000

1 2.703538000 -0.855027000 -1.874493000

1 1.303722000 -0.741192000 -2.942356000

1 2.243476000 1.261220000 -0.839963000

1 -0.333162000 1.351045000 -2.215616000

1 0.228534000 2.382671000 -1.085550000

6 0.762316000 -0.387958000 4.305723000

1 -0.038360000 -0.634307000 5.011181000

1 1.582216000 0.053752000 4.875702000

1 0.374328000 0.370010000 3.625355000

6 4.364994000 -3.797575000 3.414184000

1 4.745943000 -3.496645000 4.393250000

1 4.419205000 -4.887248000 3.349623000

1 5.053088000 -3.395087000 2.662322000

6 -0.144565000 -4.336316000 1.232968000

1 0.386218000 -5.174034000 0.776071000

1 -0.969761000 -4.748832000 1.822582000

1 -0.588329000 -3.743536000 0.431081000

6 -1.475533000 -3.257467000 -3.093799000

1 -2.540635000 -3.471287000 -3.227376000

1 -0.917160000 -4.140522000 -3.410666000

1 -1.302337000 -3.111122000 -2.026522000

6 1.187008000 -1.289631000 -6.888570000

1 2.206056000 -1.413691000 -6.506737000

1 0.957199000 -2.181237000 -7.478464000

1 1.191481000 -0.430730000 -7.563032000

6 -1.904887000 1.685407000 -4.264313000

1 -1.194771000 2.495242000 -4.449593000

1 -2.714598000 1.801635000 -4.992959000

1 -2.349588000 1.834038000 -3.280462000

6 2.938642000 6.487860000 -0.366862000

1 3.776192000 5.841788000 -0.651867000

1 2.745660000 7.160581000 -1.205457000

1 3.273812000 7.090252000 0.481377000

6 0.385504000 3.705826000 2.968595000

1 -0.457850000 4.149791000 3.507437000

1 0.225887000 2.628255000 2.938602000

1 1.290402000 3.904233000 3.546082000

6 -1.562848000 4.621380000 -1.642299000

1 -1.211674000 4.845839000 -2.651366000

1 -2.049971000 3.645831000 -1.657509000

1 -2.340210000 5.353367000 -1.397701000

1 1.986446000 1.563628000 -2.570740000

8 0.594515000 0.979842000 1.328694000

8 1.887719000 0.848789000 1.312146000

**^3^IM1_Fe/S_:**

26 -0.868972000 0.186278000 0.110686000

16 2.349557000 -0.559946000 -0.601623000

7 -2.140485000 2.072306000 0.762921000

7 -3.408040000 1.738314000 1.121659000

6 -4.089758000 2.832623000 1.521967000

6 -3.246925000 3.925034000 1.418756000

6 -2.030577000 3.401616000 0.934046000

1 -5.116220000 2.757232000 1.848057000

1 -3.469436000 4.953574000 1.658096000

7 -1.745205000 -0.890273000 1.678597000

7 -3.059658000 -0.635252000 1.934262000

6 -3.473225000 -1.330337000 3.012181000

6 -2.405055000 -2.069681000 3.480945000

6 -1.334991000 -1.774237000 2.614455000

1 -4.492396000 -1.252410000 3.358642000

1 -2.386913000 -2.745155000 4.322029000

7 -2.549500000 -0.310410000 -1.053199000

7 -3.759619000 -0.227418000 -0.434465000

6 -4.740497000 -0.588138000 -1.282383000

6 -4.167225000 -0.914152000 -2.498591000

6 -2.786543000 -0.721328000 -2.319340000

1 -5.771574000 -0.583485000 -0.963851000

1 -4.667115000 -1.247826000 -3.394226000

5 -3.899487000 0.279454000 1.025244000

1 -5.051570000 0.224404000 1.352716000

6 -0.791106000 4.159094000 0.610661000

6 -0.730356000 4.923945000 -0.571891000

6 0.430090000 5.652375000 -0.853026000

6 1.526765000 5.654848000 0.010174000

6 1.435846000 4.907239000 1.186555000

6 0.299250000 4.159060000 1.504535000

1 0.473258000 6.236688000 -1.768740000

1 2.272237000 4.906809000 1.880943000

6 0.007608000 -2.414364000 2.693120000

6 1.000978000 -1.892898000 3.547078000

6 2.221952000 -2.559994000 3.652362000

6 2.490338000 -3.734434000 2.942400000

6 1.479484000 -4.253358000 2.135203000

6 0.235259000 -3.622944000 2.010976000

1 2.988306000 -2.148598000 4.304797000

1 1.653302000 -5.180205000 1.593888000

6 -1.731911000 -0.901518000 -3.353691000

6 -0.828121000 -1.980661000 -3.288666000

6 0.127868000 -2.130209000 -4.298927000

6 0.216003000 -1.245086000 -5.372902000

6 -0.700961000 -0.191819000 -5.429005000

6 -1.678003000 -0.006599000 -4.448841000

1 0.816084000 -2.969474000 -4.244468000

1 -0.659921000 0.503037000 -6.263884000

6 2.248459000 0.721370000 -1.918012000

6 1.223548000 1.798054000 -1.607210000

7 -0.152759000 1.257760000 -1.488874000

1 3.226465000 1.189749000 -2.042501000

1 2.008261000 0.187158000 -2.842636000

1 1.471010000 2.296513000 -0.671950000

1 -0.376605000 0.749746000 -2.343726000

1 -0.793357000 2.047823000 -1.471732000

6 0.766466000 -0.629422000 4.335810000

1 -0.124623000 -0.703502000 4.966097000

1 1.617495000 -0.407236000 4.983427000

1 0.618997000 0.211538000 3.655821000

6 3.838436000 -4.406997000 3.039746000

1 4.228249000 -4.381145000 4.061008000

1 3.790527000 -5.451525000 2.723039000

1 4.576398000 -3.906510000 2.402991000

6 -0.851803000 -4.296021000 1.204603000

1 -0.428233000 -4.994724000 0.480035000

1 -1.519825000 -4.869069000 1.857628000

1 -1.473924000 -3.582036000 0.665668000

6 -0.867415000 -2.979636000 -2.160139000

1 -1.888730000 -3.190633000 -1.838238000

1 -0.402026000 -3.921354000 -2.458292000

1 -0.322206000 -2.611221000 -1.286739000

6 1.272449000 -1.406736000 -6.438190000

1 2.088778000 -0.690417000 -6.295638000

1 1.709155000 -2.407361000 -6.421580000

1 0.864578000 -1.231344000 -7.437177000

6 -2.655624000 1.138040000 -4.595934000

1 -2.295830000 1.861577000 -5.329926000

1 -3.633549000 0.784775000 -4.936971000

1 -2.826934000 1.664409000 -3.654413000

6 2.781454000 6.424714000 -0.324101000

1 3.522744000 5.779944000 -0.809189000

1 2.575904000 7.252760000 -1.006436000

1 3.251939000 6.834404000 0.573211000

6 0.257680000 3.369931000 2.790121000

1 -0.669805000 3.546898000 3.341829000

1 0.315786000 2.297825000 2.586063000

1 1.094248000 3.638369000 3.438703000

6 -1.897933000 4.997245000 -1.531073000

1 -1.594350000 5.441561000 -2.481057000

1 -2.330642000 4.015208000 -1.739370000

1 -2.709068000 5.609962000 -1.125505000

1 1.261085000 2.547144000 -2.405339000

8 0.594630000 0.232608000 1.222847000

8 2.013790000 0.366244000 0.743336000

**^3^TS2_Fe/S_:**

26 -0.849434000 0.208830000 0.132409000

16 2.326644000 -0.608319000 -0.715969000

7 -2.108920000 2.094221000 0.776952000

7 -3.380649000 1.778578000 1.138712000

6 -4.042690000 2.881309000 1.548254000

6 -3.182567000 3.960209000 1.448695000

6 -1.976701000 3.420928000 0.955902000

1 -5.069260000 2.820537000 1.876933000

1 -3.387085000 4.990886000 1.694615000

7 -1.769481000 -0.883189000 1.674558000

7 -3.079422000 -0.608398000 1.930083000

6 -3.506671000 -1.310511000 2.998011000

6 -2.451782000 -2.073327000 3.459596000

6 -1.374767000 -1.784006000 2.600056000

1 -4.525242000 -1.220208000 3.343214000

1 -2.446421000 -2.758960000 4.292552000

7 -2.553983000 -0.268171000 -1.047312000

7 -3.764941000 -0.166022000 -0.433535000

6 -4.747427000 -0.516964000 -1.283043000

6 -4.174438000 -0.855478000 -2.496127000

6 -2.792125000 -0.681797000 -2.312650000

1 -5.779304000 -0.498264000 -0.967640000

1 -4.675658000 -1.185255000 -3.392437000

5 -3.900595000 0.331156000 1.029487000

1 -5.053896000 0.295232000 1.354133000

6 -0.730390000 4.166330000 0.630330000

6 -0.663251000 4.925832000 -0.554904000

6 0.504532000 5.641609000 -0.839762000

6 1.599498000 5.640418000 0.024941000

6 1.504514000 4.893136000 1.201512000

6 0.360975000 4.158122000 1.523241000

1 0.555574000 6.216618000 -1.760911000

1 2.344752000 4.880373000 1.891182000

6 -0.039251000 -2.437921000 2.675761000

6 0.964456000 -1.919889000 3.519379000

6 2.178133000 -2.600364000 3.624341000

6 2.429343000 -3.783331000 2.922722000

6 1.409005000 -4.297033000 2.123869000

6 0.171520000 -3.653817000 2.000723000

1 2.952534000 -2.192365000 4.269283000

1 1.570051000 -5.229726000 1.588689000

6 -1.738099000 -0.881578000 -3.344114000

6 -0.852179000 -1.976236000 -3.274664000

6 0.103402000 -2.142843000 -4.281217000

6 0.207392000 -1.261955000 -5.358342000

6 -0.686509000 -0.190203000 -5.415009000

6 -1.664407000 0.012310000 -4.437820000

1 0.781617000 -2.989873000 -4.220478000

1 -0.625550000 0.508032000 -6.245648000

6 2.230916000 0.721015000 -1.983296000

6 1.232777000 1.797727000 -1.598373000

7 -0.149076000 1.266466000 -1.476885000

1 3.216246000 1.175458000 -2.106251000

1 1.961866000 0.230522000 -2.923883000

1 1.507043000 2.242245000 -0.643746000

1 -0.378025000 0.756993000 -2.329578000

1 -0.785841000 2.059384000 -1.457460000

6 0.749292000 -0.645769000 4.295971000

1 -0.149242000 -0.695567000 4.918016000

1 1.598894000 -0.436033000 4.949533000

1 0.626263000 0.192073000 3.607066000

6 3.770031000 -4.470604000 3.018575000

1 4.506713000 -3.991432000 2.364177000

1 4.171344000 -4.429924000 4.034821000

1 3.706029000 -5.520308000 2.722415000

6 -0.926323000 -4.320190000 1.203392000

1 -0.512967000 -5.022149000 0.476082000

1 -1.593123000 -4.888553000 1.861718000

1 -1.547829000 -3.602516000 0.668558000

6 -0.909207000 -2.969720000 -2.142077000

1 -1.934694000 -3.164986000 -1.823417000

1 -0.456374000 -3.919134000 -2.434887000

1 -0.361489000 -2.605836000 -1.268247000

6 1.229375000 -1.479301000 -6.447108000

1 0.812968000 -2.085407000 -7.259236000

1 1.556536000 -0.533971000 -6.886344000

1 2.111224000 -2.003707000 -6.072494000

6 -2.618176000 1.176664000 -4.586187000

1 -2.238572000 1.897467000 -5.312884000

1 -3.599424000 0.843749000 -4.938150000

1 -2.788379000 1.700902000 -3.643345000

6 2.840011000 6.443793000 -0.283229000

1 2.914795000 6.676115000 -1.347987000

1 2.837599000 7.396212000 0.258310000

1 3.747549000 5.910852000 0.011906000

6 0.313723000 3.367924000 2.807705000

1 -0.601664000 3.570243000 3.371128000

1 0.338691000 2.295475000 2.599756000

1 1.165196000 3.611581000 3.446517000

6 -1.829177000 5.004439000 -1.515745000

1 -1.519508000 5.435482000 -2.469855000

1 -2.276043000 4.026846000 -1.714718000

1 -2.631812000 5.632332000 -1.116369000

1 1.258767000 2.586806000 -2.356989000

8 0.526733000 0.268612000 1.199122000

8 2.151635000 0.237230000 0.651675000

**^3^IM2_Fe/S_:**

26 -0.885784000 0.253314000 0.210213000

16 2.612843000 -0.840778000 -0.425001000

7 -2.197045000 2.091155000 0.804960000

7 -3.455016000 1.749293000 1.189876000

6 -4.134107000 2.841489000 1.601604000

6 -3.298558000 3.937286000 1.480954000

6 -2.089557000 3.420356000 0.971360000

1 -5.154681000 2.761906000 1.944507000

1 -3.521343000 4.965144000 1.722515000

7 -1.802788000 -0.902781000 1.685954000

7 -3.117992000 -0.651179000 1.941074000

6 -3.536119000 -1.388582000 2.988221000

6 -2.470589000 -2.146680000 3.435352000

6 -1.395331000 -1.818740000 2.588635000

1 -4.557237000 -1.324504000 3.331751000

1 -2.458275000 -2.851387000 4.252043000

7 -2.583193000 -0.236072000 -1.026719000

7 -3.800185000 -0.165230000 -0.419108000

6 -4.769057000 -0.518670000 -1.283321000

6 -4.180475000 -0.827307000 -2.497472000

6 -2.802831000 -0.635682000 -2.297500000

1 -5.803685000 -0.524648000 -0.976382000

1 -4.668854000 -1.151442000 -3.402926000

5 -3.952883000 0.296677000 1.055629000

1 -5.105640000 0.233661000 1.375601000

6 -0.862624000 4.181857000 0.611627000

6 -0.830736000 4.925417000 -0.585949000

6 0.321312000 5.652936000 -0.904309000

6 1.434734000 5.676649000 -0.063074000

6 1.370776000 4.951702000 1.129396000

6 0.245224000 4.204414000 1.483914000

1 0.343020000 6.220223000 -1.831345000

1 2.221569000 4.967012000 1.805701000

6 -0.040261000 -2.430569000 2.645471000

6 0.976037000 -1.843679000 3.426345000

6 2.217581000 -2.475498000 3.505243000

6 2.483151000 -3.675496000 2.838616000

6 1.452000000 -4.255237000 2.100878000

6 0.188354000 -3.660707000 2.002536000

1 3.002041000 -2.014778000 4.100326000

1 1.627457000 -5.199946000 1.592194000

6 -1.727270000 -0.817390000 -3.308539000

6 -0.864788000 -1.931450000 -3.248065000

6 0.102922000 -2.095819000 -4.242684000

6 0.246927000 -1.188504000 -5.294461000

6 -0.625206000 -0.099491000 -5.342475000

6 -1.616645000 0.100206000 -4.376984000

1 0.757796000 -2.961958000 -4.195888000

1 -0.540307000 0.612617000 -6.158984000

6 2.140503000 0.244187000 -1.830788000

6 1.394320000 1.526332000 -1.472046000

7 -0.073647000 1.331865000 -1.370430000

1 3.086490000 0.489045000 -2.319223000

1 1.566018000 -0.384070000 -2.518235000

1 1.743198000 1.923009000 -0.520251000

1 -0.424401000 0.981754000 -2.260236000

1 -0.497438000 2.248340000 -1.243701000

6 0.742887000 -0.547377000 4.158886000

1 -0.140652000 -0.597829000 4.802261000

1 1.600488000 -0.289390000 4.783926000

1 0.582188000 0.257582000 3.438703000

6 3.850663000 -4.310998000 2.906715000

1 4.267944000 -4.259996000 3.916131000

1 3.821659000 -5.360781000 2.605880000

1 4.556086000 -3.799087000 2.243111000

6 -0.910280000 -4.385894000 1.259078000

1 -0.495024000 -5.108374000 0.553272000

1 -1.550846000 -4.940699000 1.953613000

1 -1.560111000 -3.705975000 0.707343000

6 -0.968247000 -2.950444000 -2.140477000

1 -2.007406000 -3.177370000 -1.892989000

1 -0.473489000 -3.882105000 -2.420969000

1 -0.493033000 -2.594602000 -1.221856000

6 1.323884000 -1.370746000 -6.335239000

1 2.259830000 -0.897142000 -6.019079000

1 1.540281000 -2.427626000 -6.507382000

1 1.040566000 -0.921749000 -7.289838000

6 -2.553248000 1.279442000 -4.517480000

1 -2.160956000 2.002456000 -5.235050000

1 -3.537854000 0.963749000 -4.875653000

1 -2.718557000 1.796815000 -3.569683000

6 2.679453000 6.444232000 -0.437025000

1 3.414066000 5.791702000 -0.921773000

1 2.457786000 7.256725000 -1.132815000

1 3.164869000 6.874347000 0.442579000

6 0.232222000 3.432368000 2.779389000

1 -0.676348000 3.627033000 3.356272000

1 0.269960000 2.358516000 2.580010000

1 1.091270000 3.695508000 3.400057000

6 -2.020340000 4.979196000 -1.519158000

1 -1.739948000 5.408257000 -2.483154000

1 -2.454203000 3.992505000 -1.700113000

1 -2.822559000 5.597754000 -1.104730000

1 1.598312000 2.272440000 -2.247257000

8 0.515580000 0.414650000 1.049003000

8 3.800884000 -0.138930000 0.243486000

**^3^IM3’_Fe/S_:**

26 -0.457420000 0.359943000 -0.197702000

16 2.422455000 -0.819127000 -0.847174000

7 -1.629236000 1.912579000 0.527286000

7 -2.903183000 1.596598000 0.886484000

6 -3.537966000 2.690145000 1.348022000

6 -2.662196000 3.759948000 1.288461000

6 -1.470755000 3.234165000 0.758333000

1 -4.563504000 2.631586000 1.679001000

1 -2.847553000 4.779725000 1.586707000

7 -1.229727000 -0.846994000 1.315997000

7 -2.585785000 -0.815180000 1.464863000

6 -2.976508000 -1.689530000 2.412199000

6 -1.850304000 -2.313935000 2.909863000

6 -0.767913000 -1.755461000 2.203317000

1 -4.020438000 -1.796288000 2.664301000

1 -1.796691000 -3.061295000 3.686054000

7 -2.269247000 -0.206450000 -1.517697000

7 -3.440984000 -0.183350000 -0.829230000

6 -4.475021000 -0.461849000 -1.649858000

6 -3.971026000 -0.671024000 -2.921839000

6 -2.578593000 -0.497744000 -2.793908000

1 -5.486856000 -0.491125000 -1.274547000

1 -4.521446000 -0.917215000 -3.816768000

5 -3.466487000 0.167987000 0.674838000

1 -4.594004000 0.121008000 1.078248000

6 -0.228814000 3.989505000 0.443673000

6 -0.245180000 4.925484000 -0.617304000

6 0.912412000 5.655538000 -0.895284000

6 2.083486000 5.499960000 -0.148935000

6 2.069590000 4.588238000 0.905847000

6 0.937310000 3.828525000 1.217656000

1 0.897292000 6.369364000 -1.714866000

1 2.963133000 4.463867000 1.511714000

6 0.658674000 -2.082143000 2.476051000

6 1.344484000 -1.376962000 3.482299000

6 2.659806000 -1.739130000 3.792198000

6 3.305173000 -2.793657000 3.147676000

6 2.588046000 -3.508797000 2.184735000

6 1.275945000 -3.178098000 1.841542000

1 3.188759000 -1.182148000 4.561610000

1 3.063799000 -4.348834000 1.684718000

6 -1.554678000 -0.594638000 -3.869071000

6 -0.764918000 -1.757129000 -3.995535000

6 0.168762000 -1.832069000 -5.030869000

6 0.347018000 -0.790607000 -5.946352000

6 -0.454298000 0.343109000 -5.812056000

6 -1.410500000 0.457619000 -4.795988000

1 0.773471000 -2.730477000 -5.125860000

1 -0.342142000 1.159981000 -6.520198000

6 2.337478000 0.278962000 -2.340081000

6 1.738455000 1.630777000 -2.008974000

7 0.284433000 1.539695000 -1.710757000

1 3.370336000 0.395352000 -2.676578000

1 1.778472000 -0.260657000 -3.108386000

1 2.236441000 2.051483000 -1.134650000

1 -0.193471000 1.205652000 -2.546434000

1 -0.056633000 2.485249000 -1.559048000

6 0.675808000 -0.278760000 4.276576000

1 0.025695000 -0.697884000 5.052640000

1 1.416071000 0.349424000 4.776403000

1 0.051179000 0.362380000 3.654493000

6 4.739950000 -3.140652000 3.462774000

1 5.013272000 -2.842012000 4.477686000

1 4.925908000 -4.213166000 3.363495000

1 5.426457000 -2.629629000 2.778557000

6 0.542932000 -3.992683000 0.805674000

1 1.151069000 -4.833464000 0.464874000

1 -0.396315000 -4.394673000 1.196963000

1 0.298380000 -3.371570000 -0.057522000

6 -0.912389000 -2.909262000 -3.032499000

1 -1.960004000 -3.191585000 -2.898264000

1 -0.367063000 -3.786334000 -3.386705000

1 -0.520659000 -2.641712000 -2.048489000

6 1.386835000 -0.890554000 -7.035682000

1 2.395192000 -0.742598000 -6.634243000

1 1.374602000 -1.874775000 -7.511655000

1 1.229822000 -0.138317000 -7.811723000

6 -2.282213000 1.692452000 -4.736678000

1 -1.866699000 2.493032000 -5.351728000

1 -3.290292000 1.482888000 -5.108230000

1 -2.400088000 2.070547000 -3.718402000

6 3.327779000 6.283316000 -0.488147000

1 3.876909000 5.811680000 -1.310452000

1 3.087990000 7.301622000 -0.804647000

1 4.008277000 6.343700000 0.363915000

6 0.997304000 2.869231000 2.380702000

1 0.037073000 2.788926000 2.892786000

1 1.279503000 1.864080000 2.054171000

1 1.745532000 3.190769000 3.108197000

6 -1.484113000 5.170584000 -1.450943000

1 -1.231739000 5.701349000 -2.370892000

1 -1.995566000 4.244241000 -1.723117000

1 -2.214514000 5.780868000 -0.911197000

1 1.897820000 2.316072000 -2.848015000

8 0.879952000 -1.015015000 -0.539404000

8 3.059945000 0.093058000 0.190424000

**^1^RC_Fe/S_:**

26 -0.606417000 0.294254000 0.073527000

16 0.749937000 -1.351057000 -0.664161000

7 -1.864623000 1.939973000 0.693434000

7 -3.131020000 1.609167000 1.055974000

6 -3.796316000 2.691961000 1.495931000

6 -2.942830000 3.777709000 1.423618000

6 -1.736390000 3.269402000 0.912295000

1 -4.821810000 2.612456000 1.822667000

1 -3.150020000 4.801038000 1.694904000

7 -1.443446000 -0.902788000 1.571929000

7 -2.784296000 -0.768552000 1.768147000

6 -3.189775000 -1.527876000 2.802554000

6 -2.086659000 -2.186835000 3.306818000

6 -1.002988000 -1.774027000 2.509887000

1 -4.227319000 -1.542681000 3.098916000

1 -2.049980000 -2.880449000 4.132115000

7 -2.280426000 -0.245921000 -1.225341000

7 -3.485306000 -0.243649000 -0.582421000

6 -4.469396000 -0.628488000 -1.413693000

6 -3.910129000 -0.894163000 -2.647756000

6 -2.535611000 -0.646321000 -2.494565000

1 -5.490065000 -0.683167000 -1.067561000

1 -4.410061000 -1.223779000 -3.545019000

5 -3.633873000 0.165070000 0.895125000

1 -4.783680000 0.093829000 1.221517000

6 -0.520812000 4.081530000 0.629095000

6 -0.385672000 4.726428000 -0.616833000

6 0.743876000 5.518165000 -0.854153000

6 1.724002000 5.712604000 0.118881000

6 1.538543000 5.110533000 1.366554000

6 0.432536000 4.306448000 1.645064000

1 0.850247000 6.002188000 -1.821480000

1 2.277477000 5.271202000 2.147217000

6 0.388574000 -2.273606000 2.687301000

6 1.271139000 -1.620399000 3.569070000

6 2.550069000 -2.150832000 3.762226000

6 2.968251000 -3.323273000 3.130683000

6 2.053229000 -3.987125000 2.311414000

6 0.767148000 -3.490607000 2.086093000

1 3.233410000 -1.636897000 4.433543000

1 2.346031000 -4.919131000 1.834535000

6 -1.550124000 -0.810732000 -3.597652000

6 -1.047563000 -2.099492000 -3.891871000

6 -0.194420000 -2.258836000 -4.982268000

6 0.170798000 -1.186394000 -5.805051000

6 -0.370533000 0.064462000 -5.523018000

6 -1.241408000 0.268694000 -4.443729000

1 0.198277000 -3.248992000 -5.198812000

1 -0.127278000 0.907746000 -6.164668000

6 1.601833000 -0.497040000 -2.057629000

6 1.607602000 0.997041000 -1.813326000

7 0.258011000 1.434392000 -1.347603000

1 2.626734000 -0.871108000 -2.112906000

1 1.104656000 -0.745174000 -2.994592000

1 2.303075000 1.250593000 -1.018566000

1 -0.378359000 1.460780000 -2.138993000

1 0.326123000 2.391229000 -1.006182000

6 0.847757000 -0.396648000 4.345362000

1 0.062790000 -0.641852000 5.068620000

1 1.689227000 0.022720000 4.900771000

1 0.453680000 0.376615000 3.686307000

6 4.369271000 -3.852025000 3.320285000

1 4.777618000 -3.569500000 4.293747000

1 4.402627000 -4.941583000 3.241722000

1 5.046776000 -3.452491000 2.557350000

6 -0.198151000 -4.284767000 1.237883000

1 0.306458000 -5.125985000 0.758410000

1 -1.016550000 -4.689660000 1.842128000

1 -0.649364000 -3.673681000 0.454353000

6 -1.430999000 -3.298924000 -3.060072000

1 -2.506000000 -3.495592000 -3.112869000

1 -0.910816000 -4.194319000 -3.405984000

1 -1.178018000 -3.143321000 -2.009430000

6 1.125467000 -1.388989000 -6.955914000

1 2.150676000 -1.531108000 -6.597738000

1 0.866436000 -2.276474000 -7.540024000

1 1.129298000 -0.530416000 -7.630782000

6 -1.874104000 1.633534000 -4.280743000

1 -1.148211000 2.434068000 -4.446109000

1 -2.670796000 1.773893000 -5.019287000

1 -2.330813000 1.776800000 -3.301358000

6 2.955751000 6.536488000 -0.166169000

1 3.810244000 5.894104000 -0.405478000

1 2.803965000 7.206943000 -1.014931000

1 3.242715000 7.141783000 0.697554000

6 0.250608000 3.732788000 3.028331000

1 -0.591946000 4.207509000 3.541945000

1 0.047399000 2.662454000 2.993422000

1 1.142824000 3.891318000 3.636677000

6 -1.459162000 4.640344000 -1.678093000

1 -1.054673000 4.865208000 -2.666932000

1 -1.936457000 3.660614000 -1.717395000

1 -2.255516000 5.365758000 -1.479398000

1 1.883222000 1.551326000 -2.717309000

8 0.574605000 0.899123000 1.406171000

8 1.755777000 1.365531000 1.166127000

**Reaction mechanism with selenocysteamine:**

**^5^RC_Fe/Se_:**

26 -0.447468000 0.218066000 0.083396000

34 1.149456000 -1.475724000 -0.760078000

7 -1.846638000 2.003998000 0.703811000

7 -3.095760000 1.628682000 1.095553000

6 -3.768051000 2.683599000 1.596949000

6 -2.943096000 3.791522000 1.531522000

6 -1.744484000 3.324623000 0.962306000

1 -4.779408000 2.571779000 1.956878000

1 -3.167845000 4.800788000 1.839673000

7 -1.445684000 -0.986757000 1.628380000

7 -2.784984000 -0.787637000 1.797923000

6 -3.237328000 -1.540641000 2.816434000

6 -2.174506000 -2.259066000 3.334205000

6 -1.062293000 -1.885853000 2.563256000

1 -4.278558000 -1.512203000 3.098752000

1 -2.188367000 -2.960106000 4.153864000

7 -2.303732000 -0.266716000 -1.228054000

7 -3.493692000 -0.227189000 -0.561117000

6 -4.502862000 -0.583061000 -1.378602000

6 -3.973032000 -0.862320000 -2.624269000

6 -2.589409000 -0.653428000 -2.490552000

1 -5.519386000 -0.610559000 -1.017140000

1 -4.500279000 -1.173573000 -3.512479000

5 -3.606832000 0.185100000 0.921465000

1 -4.754437000 0.129836000 1.259457000

6 -0.555055000 4.154759000 0.625178000

6 -0.464183000 4.751683000 -0.648199000

6 0.649484000 5.544458000 -0.951573000

6 1.658161000 5.782960000 -0.018786000

6 1.517544000 5.226362000 1.255892000

6 0.428307000 4.424680000 1.600824000

1 0.719918000 5.993720000 -1.938861000

1 2.279603000 5.422780000 2.005645000

6 0.320051000 -2.409615000 2.729443000

6 1.216976000 -1.766886000 3.605211000

6 2.490531000 -2.312958000 3.784648000

6 2.892540000 -3.483260000 3.137631000

6 1.970495000 -4.124744000 2.308899000

6 0.687586000 -3.613503000 2.097011000

1 3.184765000 -1.811458000 4.453904000

1 2.255145000 -5.048884000 1.812448000

6 -1.590293000 -0.818115000 -3.580962000

6 -1.036197000 -2.091691000 -3.835199000

6 -0.148726000 -2.242777000 -4.900421000

6 0.197841000 -1.175085000 -5.735688000

6 -0.392781000 0.063131000 -5.491876000

6 -1.293627000 0.258661000 -4.436873000

1 0.282950000 -3.222769000 -5.087011000

1 -0.160464000 0.901373000 -6.143878000

6 1.944796000 -0.319143000 -2.171199000

6 1.815751000 1.151225000 -1.819837000

7 0.408816000 1.487263000 -1.481925000

1 2.995792000 -0.593672000 -2.265643000

1 1.443820000 -0.560702000 -3.109435000

1 2.415181000 1.384280000 -0.941185000

1 -0.170658000 1.414191000 -2.314302000

1 0.354230000 2.455777000 -1.172329000

6 0.817350000 -0.527496000 4.369093000

1 -0.036414000 -0.722989000 5.025515000

1 1.641402000 -0.172502000 4.991097000

1 0.531156000 0.279381000 3.692012000

6 4.288246000 -4.029299000 3.316399000

1 4.699889000 -3.768164000 4.294320000

1 4.310533000 -5.117426000 3.218116000

1 4.968543000 -3.622250000 2.560062000

6 -0.285587000 -4.376444000 1.228614000

1 0.216198000 -5.191924000 0.704527000

1 -1.092838000 -4.812493000 1.826112000

1 -0.756676000 -3.736734000 0.479588000

6 -1.425750000 -3.292685000 -3.008294000

1 -2.458676000 -3.592643000 -3.213731000

1 -0.782333000 -4.145964000 -3.230481000

1 -1.355544000 -3.085712000 -1.939920000

6 1.188205000 -1.364530000 -6.858401000

1 2.213400000 -1.413490000 -6.475803000

1 1.005565000 -2.295936000 -7.401073000

1 1.145175000 -0.541895000 -7.575407000

6 -1.975277000 1.602801000 -4.302881000

1 -1.318541000 2.414752000 -4.623759000

1 -2.867878000 1.643853000 -4.936858000

1 -2.309249000 1.810606000 -3.286095000

6 2.871582000 6.608051000 -0.371927000

1 3.730268000 5.966857000 -0.599191000

1 2.691212000 7.234621000 -1.248245000

1 3.168803000 7.258765000 0.454605000

6 0.307287000 3.885424000 3.004675000

1 -0.578955000 4.284367000 3.507966000

1 0.220627000 2.798071000 2.998587000

1 1.179974000 4.156544000 3.601949000

6 -1.572496000 4.618168000 -1.668590000

1 -1.206927000 4.823397000 -2.676732000

1 -2.031386000 3.628876000 -1.664351000

1 -2.374682000 5.334622000 -1.460724000

1 2.162884000 1.770444000 -2.656090000

8 0.721135000 1.073118000 1.483112000

8 2.017133000 1.217078000 1.391293000

**^5^TS1_Fe/Se_:**

26 -0.548639000 0.343771000 0.071278000

34 1.563483000 -1.308706000 -0.615529000

7 -1.871174000 2.037789000 0.717164000

7 -3.135205000 1.664765000 1.061173000

6 -3.834783000 2.731926000 1.494497000

6 -3.014439000 3.844647000 1.428055000

6 -1.788469000 3.365973000 0.931830000

1 -4.860054000 2.625917000 1.814790000

1 -3.259196000 4.861211000 1.694249000

7 -1.458956000 -0.928447000 1.650503000

7 -2.799936000 -0.735060000 1.816008000

6 -3.258292000 -1.498295000 2.826434000

6 -2.196937000 -2.218110000 3.343089000

6 -1.081968000 -1.834629000 2.576699000

1 -4.301684000 -1.476900000 3.101675000

1 -2.212878000 -2.926797000 4.156375000

7 -2.334936000 -0.279482000 -1.239438000

7 -3.518998000 -0.242710000 -0.563072000

6 -4.529585000 -0.632367000 -1.365688000

6 -4.005603000 -0.929205000 -2.610689000

6 -2.624187000 -0.692993000 -2.489539000

1 -5.543354000 -0.668332000 -0.997013000

1 -4.536633000 -1.266647000 -3.487221000

5 -3.627246000 0.207388000 0.913762000

1 -4.776075000 0.151412000 1.251267000

6 -0.576117000 4.172076000 0.622955000

6 -0.479827000 4.828327000 -0.620401000

6 0.657686000 5.594576000 -0.899179000

6 1.688247000 5.746785000 0.028840000

6 1.549387000 5.122529000 1.271455000

6 0.436631000 4.341106000 1.589531000

1 0.731114000 6.092749000 -1.862443000

1 2.329573000 5.249251000 2.017696000

6 0.295121000 -2.376359000 2.737054000

6 1.197684000 -1.773170000 3.634426000

6 2.450037000 -2.361154000 3.830866000

6 2.832497000 -3.529232000 3.168679000

6 1.912854000 -4.121869000 2.301856000

6 0.647222000 -3.572338000 2.080331000

1 3.145308000 -1.891144000 4.521782000

1 2.183038000 -5.040474000 1.787131000

6 -1.610126000 -0.840675000 -3.568807000

6 -0.904086000 -2.053660000 -3.717184000

6 0.014422000 -2.179654000 -4.759708000

6 0.249289000 -1.144273000 -5.670814000

6 -0.481024000 0.033888000 -5.524157000

6 -1.418891000 0.201066000 -4.496859000

1 0.561274000 -3.113097000 -4.866223000

1 -0.328801000 0.845225000 -6.231093000

6 2.137689000 -0.121628000 -2.095489000

6 1.750436000 1.334471000 -1.891529000

7 0.299909000 1.488544000 -1.636474000

1 3.219389000 -0.209503000 -2.198891000

1 1.679101000 -0.535605000 -2.996985000

1 2.280986000 1.746024000 -1.035076000

1 -0.216790000 1.240419000 -2.476911000

1 0.102472000 2.470418000 -1.458386000

6 0.838179000 -0.511461000 4.379727000

1 -0.117349000 -0.607090000 4.902905000

1 1.603392000 -0.263515000 5.118219000

1 0.748818000 0.328352000 3.686337000

6 4.207057000 -4.120852000 3.366327000

1 4.915630000 -3.722113000 2.631752000

1 4.605581000 -3.889697000 4.357160000

1 4.198289000 -5.207335000 3.249060000

6 -0.329118000 -4.292011000 1.179595000

1 0.175113000 -5.060914000 0.591017000

1 -1.115434000 -4.783746000 1.762102000

1 -0.829709000 -3.610654000 0.489562000

6 -1.158171000 -3.219907000 -2.793477000

1 -2.181397000 -3.593479000 -2.902020000

1 -0.477066000 -4.045448000 -3.007221000

1 -1.031311000 -2.939947000 -1.746575000

6 1.260826000 -1.309914000 -6.778687000

1 2.269671000 -1.451476000 -6.378203000

1 1.037251000 -2.186811000 -7.393781000

1 1.283377000 -0.438225000 -7.435862000

6 -2.238376000 1.472092000 -4.448367000

1 -1.738705000 2.281784000 -4.983989000

1 -3.214419000 1.321729000 -4.921907000

1 -2.438904000 1.807603000 -3.429437000

6 2.922440000 6.551543000 -0.298835000

1 2.744574000 7.241249000 -1.126920000

1 3.261186000 7.134912000 0.561273000

1 3.752532000 5.898483000 -0.589782000

6 0.330084000 3.706246000 2.954360000

1 -0.632410000 3.923378000 3.425417000

1 0.423972000 2.620524000 2.885580000

1 1.119222000 4.070680000 3.615215000

6 -1.598972000 4.769491000 -1.636960000

1 -1.250985000 5.086813000 -2.621955000

1 -2.028804000 3.770283000 -1.731955000

1 -2.421274000 5.433387000 -1.350420000

1 2.048569000 1.906505000 -2.779326000

8 1.037592000 0.912914000 1.342158000

8 2.186482000 0.376320000 0.980865000

**^5^IM1_Fe/Se_:**

26 -0.750885000 0.388689000 0.161506000

34 1.633852000 -1.168680000 -0.438193000

7 -2.069289000 2.038505000 0.814620000

7 -3.326124000 1.663757000 1.186644000

6 -4.023736000 2.735423000 1.611168000

6 -3.210110000 3.851070000 1.513420000

6 -1.990751000 3.370371000 1.003270000

1 -5.044480000 2.631212000 1.946297000

1 -3.455699000 4.870376000 1.768148000

7 -1.692786000 -0.977324000 1.713185000

7 -3.025032000 -0.759260000 1.905372000

6 -3.486632000 -1.528968000 2.911791000

6 -2.432542000 -2.278280000 3.399118000

6 -1.321513000 -1.902931000 2.618788000

1 -4.524906000 -1.489366000 3.204356000

1 -2.449336000 -2.997822000 4.203039000

7 -2.543846000 -0.250278000 -1.131332000

7 -3.729856000 -0.216923000 -0.461304000

6 -4.736327000 -0.598659000 -1.272786000

6 -4.205683000 -0.887658000 -2.517322000

6 -2.824470000 -0.654412000 -2.386152000

1 -5.752326000 -0.635678000 -0.910376000

1 -4.732534000 -1.219736000 -3.398434000

5 -3.835883000 0.211580000 1.024496000

1 -4.987164000 0.169788000 1.357454000

6 -0.790769000 4.178380000 0.655230000

6 -0.751957000 4.859081000 -0.578051000

6 0.367550000 5.638608000 -0.889895000

6 1.436484000 5.779053000 -0.004050000

6 1.359329000 5.121900000 1.226605000

6 0.267813000 4.323170000 1.574919000

1 0.396313000 6.157036000 -1.844849000

1 2.173264000 5.233601000 1.938573000

6 0.049892000 -2.465956000 2.756274000

6 0.984862000 -1.856946000 3.615972000

6 2.226792000 -2.467913000 3.807457000

6 2.573260000 -3.659070000 3.167175000

6 1.627352000 -4.250848000 2.328129000

6 0.367122000 -3.682213000 2.120187000

1 2.944934000 -1.994684000 4.472450000

1 1.869940000 -5.186131000 1.829534000

6 -1.796262000 -0.806467000 -3.451127000

6 -1.008023000 -1.974460000 -3.515606000

6 -0.071430000 -2.105947000 -4.542665000

6 0.105100000 -1.117612000 -5.515801000

6 -0.703479000 0.016990000 -5.449223000

6 -1.659913000 0.187620000 -4.440966000

1 0.533244000 -3.008169000 -4.587596000

1 -0.596864000 0.790644000 -6.205124000

6 2.109139000 0.038407000 -1.936751000

6 1.554595000 1.444598000 -1.767638000

7 0.081370000 1.461565000 -1.637532000

1 3.195829000 0.069718000 -2.013845000

1 1.714604000 -0.457753000 -2.828710000

1 1.967290000 1.897268000 -0.866912000

1 -0.343419000 1.127713000 -2.499899000

1 -0.219092000 2.426313000 -1.525443000

6 0.670591000 -0.559163000 4.316184000

1 -0.284610000 -0.603771000 4.847085000

1 1.448460000 -0.306585000 5.040167000

1 0.604490000 0.249244000 3.583190000

6 3.938631000 -4.274647000 3.357128000

1 4.345182000 -4.052293000 4.346826000

1 3.910996000 -5.360621000 3.238229000

1 4.651547000 -3.886375000 2.621045000

6 -0.640813000 -4.405778000 1.257775000

1 -0.156769000 -5.171900000 0.648460000

1 -1.399877000 -4.903422000 1.870776000

1 -1.173569000 -3.725904000 0.591226000

6 -1.185337000 -3.088498000 -2.513140000

1 -2.206118000 -3.482084000 -2.532559000

1 -0.503301000 -3.915289000 -2.719402000

1 -1.000241000 -2.743429000 -1.494410000

6 1.149605000 -1.272420000 -6.594031000

1 2.148881000 -1.037319000 -6.211637000

1 1.185564000 -2.297040000 -6.973139000

1 0.958201000 -0.605895000 -7.437722000

6 -2.544392000 1.414320000 -4.460343000

1 -2.127583000 2.186177000 -5.110196000

1 -3.543170000 1.172083000 -4.837425000

1 -2.684522000 1.843893000 -3.466095000

6 2.647189000 6.605269000 -0.365042000

1 3.479936000 5.967696000 -0.681574000

1 2.433572000 7.294410000 -1.185157000

1 3.000650000 7.192273000 0.486798000

6 0.242784000 3.627167000 2.912842000

1 -0.723452000 3.739742000 3.411559000

1 0.427646000 2.557483000 2.784030000

1 1.014532000 4.026624000 3.574465000

6 -1.909308000 4.803218000 -1.551195000

1 -1.612329000 5.174533000 -2.534040000

1 -2.305768000 3.792639000 -1.674099000

1 -2.744212000 5.420983000 -1.205142000

1 1.879579000 2.046877000 -2.625633000

8 0.858527000 0.825246000 1.256198000

8 2.018987000 -0.018986000 0.948589000

**^5^TS2_Fe/Se_:**

26 -0.723701000 0.438176000 0.204128000

34 1.914952000 -0.907032000 -0.667977000

7 -2.053080000 2.092925000 0.832469000

7 -3.319485000 1.725644000 1.178762000

6 -4.013486000 2.801340000 1.598896000

6 -3.189477000 3.910006000 1.520275000

6 -1.964260000 3.423880000 1.031005000

1 -5.039540000 2.702872000 1.918748000

1 -3.430596000 4.930325000 1.774716000

7 -1.709304000 -0.929162000 1.721231000

7 -3.034740000 -0.686257000 1.928432000

6 -3.487643000 -1.419024000 2.964722000

6 -2.435778000 -2.171966000 3.452810000

6 -1.333548000 -1.837025000 2.643992000

1 -4.519603000 -1.355209000 3.274738000

1 -2.450040000 -2.872648000 4.273115000

7 -2.516659000 -0.246270000 -1.087513000

7 -3.715582000 -0.179414000 -0.443109000

6 -4.712638000 -0.558923000 -1.266723000

6 -4.162321000 -0.881798000 -2.493615000

6 -2.779674000 -0.670744000 -2.340166000

1 -5.735940000 -0.571419000 -0.923816000

1 -4.676905000 -1.224763000 -3.377678000

5 -3.838891000 0.274108000 1.030965000

1 -4.991877000 0.244970000 1.356066000

6 -0.755500000 4.230726000 0.711463000

6 -0.706650000 4.952686000 -0.497262000

6 0.422214000 5.730876000 -0.779464000

6 1.489515000 5.830451000 0.112923000

6 1.400677000 5.134946000 1.322066000

6 0.300245000 4.337365000 1.640606000

1 0.459971000 6.278574000 -1.717637000

1 2.213410000 5.213872000 2.039668000

6 0.027945000 -2.427671000 2.755308000

6 0.999358000 -1.823768000 3.577836000

6 2.238867000 -2.448864000 3.730872000

6 2.546344000 -3.651211000 3.089987000

6 1.562524000 -4.239863000 2.294362000

6 0.302877000 -3.656200000 2.124814000

1 2.986898000 -1.978625000 4.364088000

1 1.773698000 -5.184695000 1.799519000

6 -1.739549000 -0.868586000 -3.385082000

6 -0.911984000 -2.010903000 -3.358349000

6 0.028998000 -2.191399000 -4.373635000

6 0.172142000 -1.278897000 -5.422885000

6 -0.667328000 -0.165030000 -5.439508000

6 -1.630387000 0.052858000 -4.447061000

1 0.664388000 -3.072915000 -4.347371000

1 -0.578661000 0.554884000 -6.248847000

6 2.105006000 0.417605000 -2.135897000

6 1.401695000 1.728301000 -1.828933000

7 -0.054548000 1.549557000 -1.627769000

1 3.169453000 0.592822000 -2.293791000

1 1.699674000 -0.073702000 -3.025102000

1 1.807868000 2.161674000 -0.915543000

1 -0.468017000 1.145085000 -2.464946000

1 -0.476255000 2.467964000 -1.517890000

6 0.723931000 -0.516062000 4.275913000

1 -0.202241000 -0.552375000 4.856883000

1 1.538495000 -0.255300000 4.955106000

1 0.620796000 0.282687000 3.537051000

6 3.910026000 -4.281972000 3.236466000

1 3.870831000 -5.364064000 3.089691000

1 4.611256000 -3.879101000 2.497149000

1 4.337997000 -4.089091000 4.223401000

6 -0.747601000 -4.385366000 1.319083000

1 -0.290276000 -5.120042000 0.653006000

1 -1.439661000 -4.924738000 1.975225000

1 -1.351495000 -3.707215000 0.715295000

6 -1.043242000 -3.043807000 -2.266468000

1 -2.079251000 -3.374019000 -2.150369000

1 -0.430678000 -3.921129000 -2.481917000

1 -0.726503000 -2.645033000 -1.300773000

6 1.183099000 -1.513986000 -6.518519000

1 2.123337000 -1.904091000 -6.120424000

1 0.813192000 -2.247655000 -7.243016000

1 1.402310000 -0.595808000 -7.067574000

6 -2.542957000 1.254368000 -4.557054000

1 -2.144580000 1.981941000 -5.266855000

1 -3.537091000 0.962836000 -4.910081000

1 -2.690790000 1.758359000 -3.599219000

6 2.711852000 6.652222000 -0.217179000

1 3.545440000 6.012062000 -0.525963000

1 2.517514000 7.352437000 -1.032579000

1 3.054522000 7.226553000 0.647718000

6 0.259321000 3.602403000 2.956319000

1 -0.694241000 3.746120000 3.472022000

1 0.388326000 2.530398000 2.786633000

1 1.057798000 3.942943000 3.618984000

6 -1.860407000 4.950341000 -1.477145000

1 -1.533699000 5.277486000 -2.466374000

1 -2.329186000 3.969041000 -1.580398000

1 -2.651147000 5.634532000 -1.152014000

1 1.594884000 2.428469000 -2.651019000

8 0.702495000 0.865983000 1.196616000

8 2.300539000 0.128913000 0.708457000

**^5^IM2_Fe/Se_:**

26 -0.879923000 0.247133000 0.233160000

34 2.534779000 -1.152433000 -0.651733000

7 -2.202332000 2.090558000 0.802908000

7 -3.458895000 1.744124000 1.188749000

6 -4.140724000 2.833370000 1.604041000

6 -3.308495000 3.931822000 1.485117000

6 -2.098513000 3.419428000 0.973062000

1 -5.160683000 2.749968000 1.947880000

1 -3.533998000 4.958563000 1.728993000

7 -1.804955000 -0.904527000 1.703345000

7 -3.121637000 -0.654276000 1.950530000

6 -3.543957000 -1.389119000 2.997831000

6 -2.479320000 -2.143982000 3.453083000

6 -1.400296000 -1.816529000 2.611150000

1 -4.566977000 -1.325855000 3.335830000

1 -2.470192000 -2.846101000 4.272042000

7 -2.571331000 -0.248073000 -1.020015000

7 -3.790577000 -0.175380000 -0.415902000

6 -4.758925000 -0.514754000 -1.286287000

6 -4.168555000 -0.816303000 -2.501085000

6 -2.790479000 -0.634617000 -2.295108000

1 -5.794543000 -0.516177000 -0.982682000

1 -4.655696000 -1.129421000 -3.411086000

5 -3.952058000 0.289639000 1.056791000

1 -5.106614000 0.225343000 1.370161000

6 -0.873626000 4.185895000 0.616626000

6 -0.836504000 4.920227000 -0.586265000

6 0.312983000 5.653833000 -0.900215000

6 1.418456000 5.692408000 -0.049126000

6 1.348756000 4.976986000 1.148839000

6 0.225668000 4.224117000 1.499211000

1 0.338701000 6.214311000 -1.831287000

1 2.193157000 5.004449000 1.832793000

6 -0.043160000 -2.422994000 2.672671000

6 0.969365000 -1.830469000 3.454829000

6 2.215761000 -2.452436000 3.530124000

6 2.490461000 -3.647993000 2.858701000

6 1.463188000 -4.233752000 2.120660000

6 0.194312000 -3.649211000 2.025962000

1 2.997580000 -1.987048000 4.125112000

1 1.646094000 -5.174778000 1.607858000

6 -1.716815000 -0.817200000 -3.308090000

6 -0.874946000 -1.948668000 -3.264094000

6 0.087690000 -2.117894000 -4.261160000

6 0.245657000 -1.199730000 -5.303055000

6 -0.606426000 -0.095586000 -5.335903000

6 -1.592630000 0.110427000 -4.364699000

1 0.727701000 -2.995640000 -4.226441000

1 -0.510571000 0.624968000 -6.143627000

6 2.168152000 0.294425000 -1.965302000

6 1.422598000 1.513582000 -1.441008000

7 -0.045869000 1.312066000 -1.351166000

1 3.166643000 0.582350000 -2.297756000

1 1.653506000 -0.179552000 -2.805700000

1 1.774682000 1.790762000 -0.448917000

1 -0.393046000 0.970445000 -2.245522000

1 -0.471823000 2.226488000 -1.217578000

6 0.726351000 -0.538275000 4.191464000

1 -0.156452000 -0.597850000 4.835077000

1 1.582170000 -0.275808000 4.817140000

1 0.559024000 0.267153000 3.473105000

6 3.863007000 -4.272512000 2.926006000

1 4.246586000 -4.289883000 3.950139000

1 3.856505000 -5.298454000 2.551142000

1 4.582845000 -3.706500000 2.325197000

6 -0.899100000 -4.380342000 1.280566000

1 -0.478856000 -5.100456000 0.575295000

1 -1.537718000 -4.938747000 1.974000000

1 -1.552237000 -3.704050000 0.728091000

6 -1.005584000 -2.981423000 -2.172087000

1 -2.049114000 -3.254682000 -1.997417000

1 -0.454107000 -3.888654000 -2.424680000

1 -0.613294000 -2.613675000 -1.220309000

6 1.315730000 -1.394411000 -6.348820000

1 2.297943000 -1.095031000 -5.967433000

1 1.395606000 -2.442262000 -6.649356000

1 1.117118000 -0.799393000 -7.242600000

6 -2.510256000 1.305933000 -4.491720000

1 -2.098247000 2.038655000 -5.188122000

1 -3.493723000 1.011432000 -4.870846000

1 -2.682495000 1.804956000 -3.535579000

6 2.660970000 6.466207000 -0.417724000

1 3.406803000 5.814421000 -0.886081000

1 2.440630000 7.268499000 -1.125650000

1 3.132368000 6.910886000 0.462296000

6 0.206143000 3.461788000 2.800336000

1 -0.705589000 3.660444000 3.370957000

1 0.245259000 2.386518000 2.608080000

1 1.061601000 3.730345000 3.423743000

6 -2.019172000 4.959036000 -1.528763000

1 -1.734841000 5.383267000 -2.493743000

1 -2.444571000 3.968001000 -1.705468000

1 -2.828665000 5.574792000 -1.124347000

1 1.623600000 2.348216000 -2.122431000

8 0.502496000 0.449442000 1.103779000

8 3.966452000 -0.613068000 0.065356000

**^5^IM3’_Fe/Se_:**

26 -0.433206000 0.262424000 -0.335067000

34 2.696948000 -0.689747000 -0.781344000

7 -1.621463000 1.932123000 0.486663000

7 -2.882502000 1.587244000 0.875666000

6 -3.518200000 2.664351000 1.376054000

6 -2.661287000 3.749195000 1.310430000

6 -1.477893000 3.248332000 0.739305000

1 -4.531774000 2.585051000 1.738328000

1 -2.855452000 4.761307000 1.628927000

7 -1.276443000 -0.935733000 1.421572000

7 -2.628383000 -0.831579000 1.556988000

6 -3.062685000 -1.616593000 2.564987000

6 -1.967208000 -2.258283000 3.111166000

6 -0.861260000 -1.798865000 2.367379000

1 -4.110431000 -1.656293000 2.821808000

1 -1.953538000 -2.955295000 3.934841000

7 -2.227791000 -0.252999000 -1.498471000

7 -3.394696000 -0.248452000 -0.795454000

6 -4.425698000 -0.553244000 -1.608624000

6 -3.927820000 -0.764220000 -2.882898000

6 -2.540258000 -0.559415000 -2.771874000

1 -5.434632000 -0.594573000 -1.227329000

1 -4.480961000 -1.028013000 -3.770845000

5 -3.449852000 0.149760000 0.701566000

1 -4.592042000 0.137278000 1.064185000

6 -0.248533000 4.015515000 0.406708000

6 -0.289939000 4.962446000 -0.642567000

6 0.857691000 5.703293000 -0.933132000

6 2.042859000 5.546404000 -0.209315000

6 2.054780000 4.619520000 0.832085000

6 0.933714000 3.846869000 1.153763000

1 0.823463000 6.426522000 -1.743959000

1 2.961666000 4.489361000 1.416647000

6 0.560797000 -2.166461000 2.607813000

6 1.301144000 -1.466196000 3.579321000

6 2.620430000 -1.850584000 3.837268000

6 3.221995000 -2.916666000 3.167681000

6 2.458629000 -3.615020000 2.229156000

6 1.138338000 -3.262327000 1.938940000

1 3.190689000 -1.301121000 4.582068000

1 2.901717000 -4.459807000 1.707531000

6 -1.529152000 -0.632112000 -3.860561000

6 -0.707848000 -1.770421000 -3.997380000

6 0.212949000 -1.818056000 -5.046394000

6 0.345999000 -0.773903000 -5.965517000

6 -0.487726000 0.335503000 -5.820869000

6 -1.430620000 0.423295000 -4.790276000

1 0.841591000 -2.698901000 -5.150496000

1 -0.411505000 1.153795000 -6.532124000

6 2.609050000 0.500954000 -2.389991000

6 1.891677000 1.805884000 -2.099970000

7 0.427431000 1.621400000 -1.937252000

1 3.650460000 0.687152000 -2.663173000

1 2.137940000 -0.085631000 -3.182566000

1 2.281061000 2.238805000 -1.176841000

1 0.039316000 1.273741000 -2.811912000

1 0.010978000 2.537059000 -1.790669000

6 0.688935000 -0.335303000 4.372781000

1 -0.013553000 -0.712904000 5.123465000

1 1.457828000 0.233519000 4.899388000

1 0.130885000 0.355372000 3.738796000

6 4.660728000 -3.289471000 3.431830000

1 4.957012000 -3.051078000 4.456348000

1 4.836884000 -4.355449000 3.267657000

1 5.337111000 -2.743285000 2.764885000

6 0.358493000 -4.051563000 0.916959000

1 0.917332000 -4.931573000 0.591163000

1 -0.603389000 -4.389910000 1.312421000

1 0.152053000 -3.435225000 0.039613000

6 -0.801855000 -2.925436000 -3.031233000

1 -1.839713000 -3.198488000 -2.824507000

1 -0.290890000 -3.806150000 -3.425823000

1 -0.335676000 -2.664385000 -2.077887000

6 1.370065000 -0.844958000 -7.072038000

1 2.379692000 -0.660987000 -6.689066000

1 1.382747000 -1.831631000 -7.542888000

1 1.174560000 -0.102781000 -7.848988000

6 -2.333584000 1.634667000 -4.715832000

1 -1.957422000 2.439725000 -5.350016000

1 -3.346517000 1.395879000 -5.054797000

1 -2.429051000 2.019754000 -3.697856000

6 3.276663000 6.341147000 -0.561216000

1 3.825054000 5.871384000 -1.385215000

1 3.024536000 7.355635000 -0.880371000

1 3.962872000 6.412106000 0.285534000

6 1.029486000 2.854676000 2.286781000

1 0.076791000 2.730112000 2.803816000

1 1.343291000 1.872863000 1.918531000

1 1.774961000 3.175136000 3.018050000

6 -1.546302000 5.206194000 -1.449830000

1 -1.317492000 5.755860000 -2.364974000

1 -2.048961000 4.276914000 -1.728458000

1 -2.275457000 5.797166000 -0.887235000

1 2.102803000 2.513814000 -2.911044000

8 0.991430000 -1.061646000 -0.666429000

8 3.044473000 0.434334000 0.418806000

**^5^IM3_Fe/Se_:**

26 0.161518000 0.848943000 0.410821000

34 2.396263000 -0.766171000 -0.418880000

7 -0.170669000 2.751132000 1.562245000

7 -1.312754000 2.746941000 2.307221000

6 -1.423774000 3.900141000 2.996208000

6 -0.331360000 4.693564000 2.694498000

6 0.430503000 3.936064000 1.785646000

1 -2.268386000 4.075115000 3.645138000

1 -0.109714000 5.681125000 3.068383000

7 -0.575813000 -0.268409000 2.214884000

7 -1.671750000 0.285195000 2.805172000

6 -2.049624000 -0.448383000 3.870951000

6 -1.180185000 -1.517471000 3.988737000

6 -0.266470000 -1.368827000 2.927481000

1 -2.903408000 -0.159407000 4.464825000

1 -1.193213000 -2.300303000 4.731339000

7 -1.979559000 1.090740000 -0.210966000

7 -2.827465000 1.366395000 0.819676000

6 -4.093529000 1.465670000 0.367857000

6 -4.083917000 1.257599000 -1.000185000

6 -2.734052000 1.027942000 -1.325161000

1 -4.905805000 1.678995000 1.045977000

1 -4.925849000 1.269866000 -1.674908000

5 -2.325376000 1.575730000 2.269562000

1 -3.261818000 1.853973000 2.965294000

6 1.684159000 4.370845000 1.111843000

6 1.606799000 5.264015000 0.023590000

6 2.788281000 5.704773000 -0.580999000

6 4.044480000 5.301256000 -0.125462000

6 4.097232000 4.438277000 0.971157000

6 2.942212000 3.964022000 1.599359000

1 2.722943000 6.388537000 -1.423658000

1 5.065662000 4.125755000 1.353714000

6 0.869556000 -2.283638000 2.629260000

6 2.120292000 -2.073470000 3.240550000

6 3.152014000 -2.987027000 3.006980000

6 2.977334000 -4.105403000 2.189338000

6 1.723098000 -4.302933000 1.608753000

6 0.663808000 -3.414653000 1.816741000

1 4.116958000 -2.820297000 3.479470000

1 1.560625000 -5.173657000 0.978335000

6 -2.180394000 0.789715000 -2.686186000

6 -1.921922000 -0.520758000 -3.135683000

6 -1.445987000 -0.708750000 -4.436395000

6 -1.218945000 0.360565000 -5.305320000

6 -1.501480000 1.648585000 -4.846507000

6 -1.988019000 1.882852000 -3.556389000

1 -1.251074000 -1.721551000 -4.779903000

1 -1.349943000 2.495088000 -5.511430000

6 2.798239000 0.414486000 -1.985917000

6 2.353648000 1.845838000 -1.739346000

7 0.906058000 1.951510000 -1.429057000

1 3.875768000 0.371510000 -2.160979000

1 2.286709000 -0.056914000 -2.828319000

1 2.895527000 2.260259000 -0.889730000

1 0.355495000 1.605739000 -2.212967000

1 0.674283000 2.936671000 -1.338866000

6 2.359537000 -0.882410000 4.135255000

1 1.658284000 -0.858091000 4.974476000

1 3.371928000 -0.896410000 4.544293000

1 2.231151000 0.048414000 3.578853000

6 4.117020000 -5.059608000 1.924743000

1 4.797291000 -5.119457000 2.777984000

1 3.755669000 -6.067910000 1.708299000

1 4.710219000 -4.736648000 1.061675000

6 -0.674029000 -3.677091000 1.170207000

1 -0.661296000 -4.613809000 0.609093000

1 -1.476823000 -3.738929000 1.910548000

1 -0.931754000 -2.871278000 0.479779000

6 -2.139724000 -1.713932000 -2.239576000

1 -3.085299000 -1.646783000 -1.695736000

1 -2.144464000 -2.640448000 -2.817971000

1 -1.336864000 -1.780376000 -1.501393000

6 -0.669332000 0.130981000 -6.692393000

1 0.425490000 0.088667000 -6.680927000

1 -1.022387000 -0.813892000 -7.112861000

1 -0.954903000 0.933739000 -7.376176000

6 -2.327320000 3.295900000 -3.138647000

1 -1.897088000 4.022148000 -3.831013000

1 -3.410270000 3.454973000 -3.126112000

1 -1.969048000 3.528459000 -2.133656000

6 5.307312000 5.768290000 -0.808172000

1 5.631889000 5.051738000 -1.571011000

1 5.162656000 6.728928000 -1.308064000

1 6.131224000 5.876379000 -0.098500000

6 3.062596000 3.023202000 2.772025000

1 2.363571000 3.278735000 3.572218000

1 2.845104000 1.999322000 2.457403000

1 4.073776000 3.041897000 3.184637000

6 0.280515000 5.786792000 -0.482779000

1 0.381690000 6.201157000 -1.487891000

1 -0.491891000 5.014923000 -0.506416000

1 -0.101900000 6.584301000 0.162672000

1 2.609360000 2.445817000 -2.621715000

8 0.705395000 -0.966749000 -0.685243000

8 2.315177000 0.427326000 0.815310000

**^2^RC_Co/Se_:**

27 -0.619315000 0.295543000 0.085986000

34 0.905202000 -1.427618000 -0.641550000

7 -1.853353000 1.952948000 0.679111000

7 -3.126607000 1.621079000 1.015019000

6 -3.805995000 2.704619000 1.432664000

6 -2.956108000 3.792763000 1.369356000

6 -1.735803000 3.284150000 0.890433000

1 -4.837232000 2.622312000 1.740012000

1 -3.172386000 4.817918000 1.626328000

7 -1.418179000 -0.882904000 1.569816000

7 -2.756658000 -0.734561000 1.774184000

6 -3.157062000 -1.462998000 2.831179000

6 -2.054353000 -2.116380000 3.342994000

6 -0.975102000 -1.735154000 2.526512000

1 -4.192406000 -1.463719000 3.135234000

1 -2.015258000 -2.791589000 4.183191000

7 -2.263478000 -0.285383000 -1.225114000

7 -3.467608000 -0.273549000 -0.582989000

6 -4.453266000 -0.671555000 -1.407656000

6 -3.894809000 -0.953467000 -2.637441000

6 -2.519539000 -0.700576000 -2.488647000

1 -5.473466000 -0.721308000 -1.059443000

1 -4.394795000 -1.293236000 -3.530898000

5 -3.616992000 0.171035000 0.881445000

1 -4.764734000 0.096287000 1.213862000

6 -0.519532000 4.102375000 0.628613000

6 -0.362090000 4.745867000 -0.614928000

6 0.767361000 5.545057000 -0.828864000

6 1.722156000 5.751353000 0.165864000

6 1.513251000 5.150961000 1.411105000

6 0.409549000 4.336904000 1.665346000

1 0.892150000 6.026760000 -1.795196000

1 2.232930000 5.320000000 2.207877000

6 0.404736000 -2.268187000 2.696572000

6 1.315572000 -1.634691000 3.564465000

6 2.580893000 -2.200066000 3.744750000

6 2.956870000 -3.390009000 3.118835000

6 2.011125000 -4.036929000 2.321922000

6 0.737420000 -3.504777000 2.107548000

1 3.287631000 -1.700414000 4.402385000

1 2.269578000 -4.982908000 1.852881000

6 -1.540452000 -0.857445000 -3.598709000

6 -1.035532000 -2.142817000 -3.903999000

6 -0.186913000 -2.292482000 -4.998873000

6 0.166207000 -1.214712000 -5.820392000

6 -0.380820000 0.031473000 -5.530441000

6 -1.243260000 0.227152000 -4.442551000

1 0.209713000 -3.279580000 -5.222333000

1 -0.146818000 0.878700000 -6.170371000

6 1.648410000 -0.389145000 -2.162057000

6 1.559399000 1.081189000 -1.828422000

7 0.194730000 1.421055000 -1.320539000

1 2.691063000 -0.686687000 -2.285734000

1 1.109500000 -0.636393000 -3.073437000

1 2.254217000 1.338182000 -1.033531000

1 -0.460501000 1.421743000 -2.096873000

1 0.219536000 2.374801000 -0.964490000

6 0.936159000 -0.398807000 4.343991000

1 0.156761000 -0.623047000 5.079973000

1 1.796608000 -0.001143000 4.886137000

1 0.551962000 0.383660000 3.690402000

6 4.345252000 -3.955724000 3.293454000

1 4.775377000 -3.675856000 4.258229000

1 4.347284000 -5.046380000 3.224405000

1 5.021910000 -3.581584000 2.517073000

6 -0.265410000 -4.287506000 1.291535000

1 0.220629000 -5.109381000 0.762115000

1 -1.041438000 -4.719763000 1.932268000

1 -0.770960000 -3.665266000 0.551434000

6 -1.423302000 -3.350794000 -3.086773000

1 -2.486396000 -3.584205000 -3.203319000

1 -0.854562000 -4.229541000 -3.397342000

1 -1.237750000 -3.184328000 -2.024357000

6 1.112802000 -1.408554000 -6.979388000

1 2.135750000 -1.581918000 -6.628867000

1 0.832763000 -2.275428000 -7.584663000

1 1.132389000 -0.533790000 -7.632824000

6 -1.874726000 1.591465000 -4.268070000

1 -1.139241000 2.391730000 -4.387581000

1 -2.642629000 1.755415000 -5.031607000

1 -2.365849000 1.713597000 -3.302484000

6 2.952293000 6.586589000 -0.091497000

1 3.826941000 5.951903000 -0.270600000

1 2.827953000 7.226761000 -0.967580000

1 3.190405000 7.224503000 0.763741000

6 0.201434000 3.763105000 3.044967000

1 -0.648787000 4.240060000 3.543805000

1 -0.004737000 2.693313000 3.008292000

1 1.083136000 3.919139000 3.669032000

6 -1.410191000 4.654875000 -1.701579000

1 -0.970860000 4.818564000 -2.687773000

1 -1.928743000 3.695712000 -1.711899000

1 -2.178807000 5.422109000 -1.557850000

1 1.777087000 1.708794000 -2.700921000

8 0.607070000 0.867884000 1.433656000

8 1.680229000 1.513939000 1.144788000

**^2^TS1_Co/Se_:**

27 -0.757263000 0.224912000 0.097158000

34 2.966231000 -0.656873000 -0.645486000

7 -1.945495000 2.057576000 0.731126000

7 -3.218720000 1.740288000 1.087007000

6 -3.878391000 2.839133000 1.510663000

6 -3.014934000 3.916331000 1.427310000

6 -1.809354000 3.380714000 0.929692000

1 -4.905654000 2.776102000 1.836636000

1 -3.216505000 4.944828000 1.684359000

7 -1.590550000 -0.886435000 1.598033000

7 -2.905964000 -0.648525000 1.854578000

6 -3.307642000 -1.355024000 2.927862000

6 -2.228075000 -2.082895000 3.392142000

6 -1.162525000 -1.767120000 2.529070000

1 -4.327061000 -1.292391000 3.276666000

1 -2.201009000 -2.763283000 4.228833000

7 -2.353685000 -0.263030000 -1.108217000

7 -3.572930000 -0.183855000 -0.506099000

6 -4.541918000 -0.547149000 -1.366451000

6 -3.952081000 -0.869414000 -2.574206000

6 -2.573462000 -0.676254000 -2.377358000

1 -5.576335000 -0.548413000 -1.059192000

1 -4.437788000 -1.203112000 -3.477524000

5 -3.733261000 0.293510000 0.959082000

1 -4.888274000 0.243438000 1.273502000

6 -0.560796000 4.126972000 0.614609000

6 -0.451027000 4.816247000 -0.608848000

6 0.719719000 5.531747000 -0.884457000

6 1.775143000 5.596521000 0.025241000

6 1.629926000 4.932385000 1.246417000

6 0.482123000 4.202378000 1.560899000

1 0.802481000 6.056301000 -1.832961000

1 2.432732000 4.985299000 1.977361000

6 0.199402000 -2.364769000 2.596192000

6 1.176671000 -1.819078000 3.455395000

6 2.428518000 -2.430713000 3.529372000

6 2.739609000 -3.577202000 2.791698000

6 1.741078000 -4.128790000 1.991103000

6 0.469247000 -3.552079000 1.892070000

1 3.183439000 -1.999312000 4.181925000

1 1.948694000 -5.037265000 1.431327000

6 -1.519952000 -0.867245000 -3.411477000

6 -0.641262000 -1.969509000 -3.355072000

6 0.295290000 -2.145653000 -4.375763000

6 0.389788000 -1.265277000 -5.455635000

6 -0.492982000 -0.185214000 -5.497318000

6 -1.454780000 0.026219000 -4.504601000

1 0.968863000 -2.996957000 -4.324292000

1 -0.438077000 0.513405000 -6.327865000

6 2.377707000 0.468661000 -2.181004000

6 1.462600000 1.627311000 -1.810116000

7 0.059994000 1.226315000 -1.493716000

1 3.277699000 0.879284000 -2.638847000

1 1.899814000 -0.190737000 -2.908208000

1 1.855155000 2.186865000 -0.966554000

1 -0.302683000 0.749284000 -2.316950000

1 -0.479201000 2.086684000 -1.432723000

6 0.882254000 -0.612968000 4.312279000

1 0.104230000 -0.829342000 5.051378000

1 1.774073000 -0.291929000 4.854529000

1 0.531957000 0.220971000 3.703325000

6 4.119058000 -4.186430000 2.855884000

1 4.511346000 -4.187473000 3.876567000

1 4.121180000 -5.216276000 2.491716000

1 4.827632000 -3.620491000 2.241331000

6 -0.600554000 -4.265188000 1.097745000

1 -0.160097000 -4.918924000 0.342267000

1 -1.211954000 -4.893705000 1.755286000

1 -1.280937000 -3.576581000 0.597873000

6 -0.688809000 -2.955414000 -2.216248000

1 -1.714519000 -3.189283000 -1.923566000

1 -0.189599000 -3.887919000 -2.486013000

1 -0.182996000 -2.561283000 -1.331076000

6 1.397662000 -1.493945000 -6.554950000

1 2.344538000 -1.869770000 -6.159528000

1 1.032225000 -2.236252000 -7.272950000

1 1.601670000 -0.576446000 -7.110994000

6 -2.400633000 1.198498000 -4.639979000

1 -2.022652000 1.917789000 -5.368969000

1 -3.388102000 0.874220000 -4.982492000

1 -2.556391000 1.722457000 -3.694440000

6 3.043345000 6.347856000 -0.299721000

1 3.843419000 5.661127000 -0.596938000

1 2.894204000 7.051297000 -1.121845000

1 3.409486000 6.909435000 0.563867000

6 0.366611000 3.528119000 2.906333000

1 -0.545371000 3.831362000 3.428709000

1 0.332243000 2.441965000 2.801977000

1 1.218118000 3.779111000 3.541859000

6 -1.583502000 4.841243000 -1.611996000

1 -1.234282000 5.185516000 -2.587449000

1 -2.053158000 3.863626000 -1.744897000

1 -2.379092000 5.521060000 -1.289800000

1 1.426328000 2.303124000 -2.673302000

8 0.667887000 0.428977000 1.303117000

8 1.827485000 0.877817000 0.887061000

**^2^IM1_Co/Se_:**

27 -0.707579000 0.239039000 0.099195000

34 3.117519000 -0.049526000 -0.204999000

7 -1.937348000 2.089284000 0.708356000

7 -3.200919000 1.759420000 1.084446000

6 -3.873230000 2.854610000 1.498461000

6 -3.028021000 3.943360000 1.386761000

6 -1.820143000 3.417492000 0.882770000

1 -4.895336000 2.780878000 1.838162000

1 -3.242329000 4.972405000 1.631167000

7 -1.554014000 -0.886986000 1.616037000

7 -2.864309000 -0.621920000 1.879682000

6 -3.278300000 -1.323687000 2.952160000

6 -2.214452000 -2.077329000 3.409040000

6 -1.145714000 -1.783183000 2.541220000

1 -4.295478000 -1.241213000 3.303401000

1 -2.199078000 -2.762303000 4.242398000

7 -2.334346000 -0.253478000 -1.093168000

7 -3.549794000 -0.185063000 -0.485262000

6 -4.518713000 -0.577950000 -1.333923000

6 -3.930564000 -0.908489000 -2.540793000

6 -2.553754000 -0.689269000 -2.353232000

1 -5.551235000 -0.592382000 -1.020463000

1 -4.416585000 -1.262732000 -3.436214000

5 -3.700122000 0.306412000 0.976635000

1 -4.852870000 0.250937000 1.300646000

6 -0.588173000 4.180604000 0.544229000

6 -0.536519000 4.925913000 -0.650679000

6 0.614311000 5.666755000 -0.941778000

6 1.706246000 5.703502000 -0.073917000

6 1.622505000 4.976246000 1.116171000

6 0.498388000 4.214443000 1.442467000

1 0.651725000 6.234902000 -1.867796000

1 2.456724000 5.001122000 1.812752000

6 0.190593000 -2.438919000 2.599665000

6 1.202244000 -1.936590000 3.444171000

6 2.419764000 -2.614228000 3.517582000

6 2.667268000 -3.779551000 2.785273000

6 1.637273000 -4.282419000 1.992574000

6 0.395184000 -3.642263000 1.900814000

1 3.200299000 -2.218862000 4.163084000

1 1.793341000 -5.204501000 1.437920000

6 -1.492301000 -0.869858000 -3.381080000

6 -0.583117000 -1.947084000 -3.303928000

6 0.370549000 -2.105534000 -4.311130000

6 0.454429000 -1.231802000 -5.398087000

6 -0.458361000 -0.178982000 -5.461088000

6 -1.438134000 0.014453000 -4.481237000

1 1.064601000 -2.939622000 -4.245756000

1 -0.414114000 0.512111000 -6.298423000

6 2.390123000 0.545499000 -1.966595000

6 1.438680000 1.721576000 -1.809130000

7 0.047178000 1.297129000 -1.494457000

1 3.254255000 0.840786000 -2.562480000

1 1.905868000 -0.299513000 -2.456699000

1 1.769736000 2.398317000 -1.024138000

1 -0.313948000 0.820198000 -2.317914000

1 -0.512043000 2.142707000 -1.411384000

6 0.991055000 -0.685907000 4.259819000

1 0.102379000 -0.760306000 4.893452000

1 1.848782000 -0.490954000 4.907370000

1 0.852221000 0.170122000 3.596838000

6 4.013081000 -4.460597000 2.846388000

1 4.429075000 -4.439796000 3.857326000

1 3.951107000 -5.503843000 2.527889000

1 4.737068000 -3.962355000 2.192070000

6 -0.714007000 -4.304496000 1.115725000

1 -0.310938000 -4.980364000 0.358598000

1 -1.351323000 -4.901521000 1.777991000

1 -1.362981000 -3.584188000 0.618883000

6 -0.620298000 -2.929258000 -2.160573000

1 -1.643871000 -3.181236000 -1.876609000

1 -0.102454000 -3.853530000 -2.423963000

1 -0.132090000 -2.526149000 -1.268823000

6 1.489676000 -1.438056000 -6.476447000

1 2.484926000 -1.595219000 -6.051459000

1 1.258193000 -2.321156000 -7.081209000

1 1.542360000 -0.581644000 -7.151697000

6 -2.412931000 1.160313000 -4.637132000

1 -2.054290000 1.874430000 -5.380840000

1 -3.393373000 0.806008000 -4.969607000

1 -2.578029000 1.698261000 -3.700903000

6 2.949509000 6.489506000 -0.413545000

1 3.746884000 5.830957000 -0.774980000

1 2.756996000 7.228587000 -1.194402000

1 3.342400000 7.015710000 0.460553000

6 0.461956000 3.446073000 2.740380000

1 -0.460697000 3.637408000 3.295692000

1 0.513538000 2.371000000 2.553339000

1 1.304585000 3.720425000 3.378568000

6 -1.705474000 4.971215000 -1.610199000

1 -1.408845000 5.409196000 -2.565307000

1 -2.125490000 3.981508000 -1.807779000

1 -2.524293000 5.577457000 -1.210259000

1 1.425063000 2.285327000 -2.748741000

8 0.778337000 0.256601000 1.232644000

8 1.980922000 0.956697000 0.851797000

**^2^TS2_Co/Se_:**

27 -0.708757000 0.188413000 0.071346000

34 2.894195000 -0.435621000 -1.101677000

7 -1.935241000 2.045831000 0.688275000

7 -3.205255000 1.736507000 1.061234000

6 -3.847120000 2.834595000 1.512535000

6 -2.976186000 3.905090000 1.427469000

6 -1.784020000 3.366405000 0.900985000

1 -4.868453000 2.775659000 1.857310000

1 -3.163753000 4.931100000 1.703950000

7 -1.595188000 -0.901396000 1.607315000

7 -2.913096000 -0.647401000 1.842910000

6 -3.344145000 -1.356451000 2.903604000

6 -2.284311000 -2.102311000 3.381649000

6 -1.198667000 -1.793948000 2.540851000

1 -4.369027000 -1.281952000 3.233541000

1 -2.280022000 -2.786550000 4.215686000

7 -2.378468000 -0.286791000 -1.120600000

7 -3.594590000 -0.194892000 -0.518835000

6 -4.566355000 -0.580009000 -1.367378000

6 -3.978428000 -0.928102000 -2.569797000

6 -2.599360000 -0.727719000 -2.378727000

1 -5.600052000 -0.578063000 -1.057360000

1 -4.466888000 -1.281273000 -3.464296000

5 -3.734874000 0.294878000 0.942465000

1 -4.887507000 0.259765000 1.268919000

6 -0.540874000 4.117525000 0.577576000

6 -0.521008000 4.975581000 -0.541795000

6 0.637993000 5.707617000 -0.817270000

6 1.771532000 5.627739000 -0.006666000

6 1.725285000 4.780373000 1.102114000

6 0.591038000 4.024948000 1.412650000

1 0.652957000 6.358664000 -1.687785000

1 2.596302000 4.703458000 1.747929000

6 0.151333000 -2.410942000 2.651854000

6 1.118106000 -1.853875000 3.513416000

6 2.351075000 -2.491641000 3.649573000

6 2.655838000 -3.671063000 2.962941000

6 1.669515000 -4.225740000 2.148948000

6 0.414210000 -3.625212000 1.993845000

1 3.097605000 -2.052829000 4.306967000

1 1.872699000 -5.157283000 1.626290000

6 -1.536588000 -0.925938000 -3.401852000

6 -0.627390000 -1.999491000 -3.308064000

6 0.332110000 -2.168389000 -4.310683000

6 0.418527000 -1.309571000 -5.406910000

6 -0.496063000 -0.256746000 -5.486094000

6 -1.478558000 -0.053315000 -4.513838000

1 1.028263000 -2.999075000 -4.230612000

1 -0.447710000 0.425251000 -6.330953000

6 2.327095000 1.006207000 -2.354382000

6 1.295417000 1.921046000 -1.722027000

7 -0.030714000 1.270820000 -1.540739000

1 3.215079000 1.583438000 -2.614098000

1 1.956948000 0.516614000 -3.258580000

1 1.639003000 2.274134000 -0.752104000

1 -0.251358000 0.744666000 -2.384219000

1 -0.729299000 2.008251000 -1.497776000

6 0.838779000 -0.588219000 4.284210000

1 -0.035915000 -0.694479000 4.933100000

1 1.689647000 -0.315915000 4.912380000

1 0.639642000 0.237585000 3.599805000

6 4.016251000 -4.312458000 3.093127000

1 4.387935000 -4.262413000 4.120195000

1 3.996236000 -5.362460000 2.792040000

1 4.753775000 -3.805405000 2.461127000

6 -0.644532000 -4.333497000 1.180453000

1 -0.193729000 -5.023207000 0.463733000

1 -1.301884000 -4.922716000 1.829939000

1 -1.281929000 -3.640650000 0.631850000

6 -0.661270000 -2.967002000 -2.152469000

1 -1.682182000 -3.181954000 -1.831769000

1 -0.182008000 -3.910603000 -2.420872000

1 -0.127723000 -2.567648000 -1.285182000

6 1.444867000 -1.529557000 -6.491145000

1 2.327637000 -2.048071000 -6.110589000

1 1.033458000 -2.141941000 -7.301084000

1 1.769802000 -0.585628000 -6.935249000

6 -2.451337000 1.092243000 -4.683869000

1 -2.088115000 1.800257000 -5.431233000

1 -3.430536000 0.736735000 -5.018682000

1 -2.620901000 1.637309000 -3.752598000

6 2.999522000 6.455296000 -0.300556000

1 3.913436000 5.943703000 0.010866000

1 3.085046000 6.684972000 -1.365335000

1 2.967549000 7.410905000 0.234710000

6 0.602948000 3.122674000 2.621441000

1 -0.308354000 3.233071000 3.215719000

1 0.669784000 2.074092000 2.318078000

1 1.456519000 3.346493000 3.264879000

6 -1.724414000 5.135515000 -1.444535000

1 -1.447870000 5.632344000 -2.376716000

1 -2.188911000 4.178514000 -1.695247000

1 -2.502817000 5.740562000 -0.969228000

1 1.179147000 2.794808000 -2.371823000

8 0.752257000 0.051896000 1.023411000

8 2.555629000 0.369876000 0.398003000

**^2^IM2_Co/Se_:**

27 -0.787176000 0.279638000 0.109529000

34 2.677270000 -0.749302000 0.008616000

7 -2.026691000 2.061015000 0.736947000

7 -3.289723000 1.739593000 1.119655000

6 -3.950166000 2.840119000 1.536706000

6 -3.096045000 3.921994000 1.419909000

6 -1.895415000 3.388316000 0.908196000

1 -4.971445000 2.775381000 1.880595000

1 -3.301132000 4.952789000 1.664419000

7 -1.629104000 -0.881538000 1.591885000

7 -2.945286000 -0.652342000 1.858630000

6 -3.338369000 -1.384364000 2.917983000

6 -2.254508000 -2.119067000 3.360419000

6 -1.193904000 -1.780247000 2.500284000

1 -4.356368000 -1.333326000 3.272784000

1 -2.220780000 -2.817015000 4.182277000

7 -2.419713000 -0.220299000 -1.085617000

7 -3.641710000 -0.164958000 -0.489199000

6 -4.597637000 -0.547209000 -1.354787000

6 -3.993884000 -0.857208000 -2.561192000

6 -2.621303000 -0.638202000 -2.355381000

1 -5.633822000 -0.571541000 -1.054012000

1 -4.469354000 -1.199113000 -3.466942000

5 -3.795350000 0.291064000 0.984304000

1 -4.945506000 0.224910000 1.311998000

6 -0.662830000 4.140969000 0.548698000

6 -0.621564000 4.872793000 -0.655401000

6 0.531927000 5.598714000 -0.971325000

6 1.637591000 5.633910000 -0.120350000

6 1.563969000 4.921078000 1.078806000

6 0.436673000 4.174465000 1.430299000

1 0.560633000 6.156660000 -1.903816000

1 2.407995000 4.945869000 1.763362000

6 0.167790000 -2.377399000 2.551660000

6 1.174555000 -1.792904000 3.345531000

6 2.419593000 -2.418719000 3.426907000

6 2.695811000 -3.612105000 2.753611000

6 1.671312000 -4.192575000 2.006611000

6 0.406162000 -3.604109000 1.904069000

1 3.196803000 -1.959612000 4.032656000

1 1.854848000 -5.133162000 1.493289000

6 -1.540577000 -0.804399000 -3.364363000

6 -0.636136000 -1.883350000 -3.283668000

6 0.348539000 -2.019629000 -4.266197000

6 0.462688000 -1.124214000 -5.330701000

6 -0.449645000 -0.068927000 -5.398380000

6 -1.456238000 0.103712000 -4.444618000

1 1.042851000 -2.852658000 -4.196359000

1 -0.380407000 0.640369000 -6.218877000

6 2.394337000 0.435445000 -1.580201000

6 1.480868000 1.633061000 -1.412467000

7 0.039646000 1.278279000 -1.444148000

1 3.406218000 0.767917000 -1.814233000

1 2.066357000 -0.220793000 -2.390308000

1 1.672964000 2.154532000 -0.476733000

1 -0.168573000 0.799079000 -2.319006000

1 -0.496011000 2.142508000 -1.468792000

6 0.924885000 -0.512053000 4.099884000

1 0.068300000 -0.600636000 4.775194000

1 1.795235000 -0.232355000 4.697498000

1 0.710070000 0.295139000 3.396904000

6 4.066483000 -4.240133000 2.818133000

1 4.539409000 -4.075421000 3.789698000

1 4.024241000 -5.317274000 2.639831000

1 4.729238000 -3.810558000 2.059013000

6 -0.686313000 -4.335353000 1.157992000

1 -0.265362000 -5.037308000 0.435226000

1 -1.308060000 -4.914413000 1.850010000

1 -1.354597000 -3.658471000 0.625665000

6 -0.702626000 -2.890024000 -2.162659000

1 -1.732472000 -3.119823000 -1.882749000

1 -0.210613000 -3.821943000 -2.447368000

1 -0.199882000 -2.521289000 -1.263977000

6 1.518255000 -1.309260000 -6.392748000

1 2.408436000 -1.800173000 -5.993424000

1 1.143683000 -1.933708000 -7.211313000

1 1.822225000 -0.354742000 -6.828453000

6 -2.428305000 1.251396000 -4.602185000

1 -2.047987000 1.985583000 -5.314883000

1 -3.395964000 0.903261000 -4.976496000

1 -2.626091000 1.763988000 -3.658251000

6 2.883567000 6.401006000 -0.491192000

1 3.617518000 5.749352000 -0.978191000

1 2.663357000 7.215906000 -1.184627000

1 3.369063000 6.827852000 0.389900000

6 0.414148000 3.412045000 2.730844000

1 -0.496440000 3.613974000 3.302096000

1 0.447462000 2.337028000 2.535112000

1 1.271030000 3.675888000 3.354385000

6 -1.804616000 4.919730000 -1.597377000

1 -1.515926000 5.332690000 -2.565861000

1 -2.245880000 3.934159000 -1.766894000

1 -2.605294000 5.549349000 -1.196761000

1 1.680975000 2.331211000 -2.233932000

8 0.640799000 0.464941000 1.001978000

8 4.035260000 -1.587440000 -0.623663000

**^2^IM3_Co/Se_:**

27 0.125462000 0.909216000 0.520942000

34 2.255650000 -0.799025000 -0.509195000

7 -0.172514000 2.650885000 1.552227000

7 -1.327876000 2.724955000 2.270106000

6 -1.393644000 3.898764000 2.925908000

6 -0.254967000 4.624728000 2.630869000

6 0.489149000 3.810869000 1.757728000

1 -2.243803000 4.132711000 3.548199000

1 0.011975000 5.607855000 2.985547000

7 -0.544604000 -0.164278000 2.128211000

7 -1.699294000 0.289589000 2.693489000

6 -2.110821000 -0.549578000 3.662623000

6 -1.205479000 -1.588928000 3.742159000

6 -0.230953000 -1.315136000 2.763115000

1 -3.011126000 -0.344491000 4.221257000

1 -1.222969000 -2.431996000 4.415125000

7 -2.087540000 1.145133000 -0.232995000

7 -2.924892000 1.433495000 0.796755000

6 -4.191235000 1.583667000 0.351298000

6 -4.188447000 1.390202000 -1.018177000

6 -2.842187000 1.122058000 -1.344677000

1 -4.994803000 1.814934000 1.034261000

1 -5.030309000 1.434309000 -1.692101000

5 -2.380542000 1.591939000 2.231270000

1 -3.282508000 1.860434000 2.973997000

6 1.772723000 4.193068000 1.107320000

6 1.744779000 5.109838000 0.032798000

6 2.948428000 5.511069000 -0.551892000

6 4.182959000 5.050323000 -0.089189000

6 4.188036000 4.174681000 0.996493000

6 3.008229000 3.736914000 1.606921000

1 2.920108000 6.209739000 -1.384168000

1 5.138482000 3.821553000 1.388962000

6 0.965525000 -2.172757000 2.537208000

6 2.144349000 -1.922474000 3.265280000

6 3.227246000 -2.794567000 3.127976000

6 3.171281000 -3.917324000 2.300104000

6 1.980238000 -4.165638000 1.615633000

6 0.872057000 -3.319612000 1.725179000

1 4.136590000 -2.592812000 3.688717000

1 1.905564000 -5.045009000 0.980746000

6 -2.285088000 0.871417000 -2.702548000

6 -2.003726000 -0.443654000 -3.127918000

6 -1.517177000 -0.648238000 -4.421494000

6 -1.301199000 0.409032000 -5.308981000

6 -1.602996000 1.700386000 -4.874514000

6 -2.099274000 1.951229000 -3.590518000

1 -1.305938000 -1.664066000 -4.746154000

1 -1.456712000 2.537329000 -5.552641000

6 2.460880000 0.425329000 -2.076383000

6 2.073397000 1.836749000 -1.678870000

7 0.688458000 1.909730000 -1.139677000

1 3.504762000 0.384580000 -2.395288000

1 1.831318000 0.007214000 -2.864951000

1 2.745112000 2.204294000 -0.905234000

1 0.036626000 1.581735000 -1.851404000

1 0.462044000 2.887445000 -0.986501000

6 2.241966000 -0.754063000 4.214765000

1 1.525875000 -0.849371000 5.037363000

1 3.240978000 -0.685587000 4.650081000

1 2.026617000 0.189385000 3.712254000

6 4.366794000 -4.824688000 2.138010000

1 4.966933000 -4.866949000 3.050511000

1 4.066798000 -5.844062000 1.882963000

1 5.024764000 -4.471015000 1.336126000

6 -0.396296000 -3.650379000 0.978642000

1 -0.292818000 -4.587728000 0.427638000

1 -1.250365000 -3.752203000 1.654632000

1 -0.632979000 -2.856542000 0.268317000

6 -2.217514000 -1.621765000 -2.211238000

1 -3.200254000 -1.587886000 -1.732922000

1 -2.139758000 -2.562528000 -2.760819000

1 -1.465677000 -1.625756000 -1.418694000

6 -0.740180000 0.161140000 -6.688328000

1 0.353472000 0.098463000 -6.664166000

1 -1.105422000 -0.779865000 -7.107476000

1 -1.003491000 0.964575000 -7.380065000

6 -2.450396000 3.368690000 -3.198140000

1 -2.013592000 4.086594000 -3.895176000

1 -3.533935000 3.523845000 -3.202118000

1 -2.106462000 3.616328000 -2.191672000

6 5.470405000 5.474615000 -0.753116000

1 5.772147000 4.755301000 -1.522623000

1 5.370095000 6.447046000 -1.240812000

1 6.290965000 5.538344000 -0.034308000

6 3.100778000 2.782844000 2.771230000

1 2.247619000 2.871322000 3.445767000

1 3.130508000 1.753733000 2.403397000

1 4.011527000 2.962489000 3.347824000

6 0.450995000 5.705221000 -0.479022000

1 0.586154000 6.131553000 -1.474898000

1 -0.363685000 4.978941000 -0.528098000

1 0.101055000 6.509337000 0.176426000

1 2.165399000 2.494908000 -2.549977000

8 0.513456000 -0.767126000 -0.456109000

8 2.628392000 0.288700000 0.731887000

**References**

[1] L. Müller, S. Hoof, M. Keck, C. Herwig, C. Limberg, "Enhancing Tris(Pyrazolyl)Borate-Based Models of Cysteine/Cysteamine Dioxygenases through Steric Effects: Increased Reactivities, Full Product Characterization and Hints to Initial Superoxide Formation" *Chem. Eur. J.*, **2020**, *26*, 11851–11861.

[2] A. L. Rheingold, C. B. White, S. Trofimenko, "Hydrotris(3-Mesitylpyrazol-1-yl)Borate and Hydrobis(3-Mesitylpyrazol-1-yl)(5-Mesitylpyrazol-1-yl)Borate: Symmetric and Asymmetric Ligands with Rotationally Restricted Aryl Substituents" *Inorg. Chem.*, **1993**, *32*, 3471–3477.

[3] R. J. C. Dubey, R. J. Comito, Z. Wu, G. Zhang, A. J. Rieth, C. H. Hendon, J. T. Miller, M. Dincǎ, "Highly Stereoselective Heterogeneous Diene Polymerization by Co-MFU-4l: A Single-Site Catalyst Prepared by Cation Exchange" *J. Am. Chem. Soc.*, **2017**, *139*, 12664–12669.

[4] H. Myamoto, Y. Yampolski, C. L. Young, "IUPAC-NIST Solubility Data Series. 103. Oxygen and Ozone in Water, Aqueous Solutions, and Organic Liquids" *J. Phys. Chem. Ref. Data*, **2014**, *43*, 1–209.

[5] D. Kass, T. Corona, K. Warm, B. Braun-Cula, U. Kuhlmann, E. Bill, S. Mebs, M. Swart, H. Dau, M. Haumann, P. Hildebrandt, K. Ray, "Stoichiometric Formation of an Oxoiron(IV) Complex by a Soluble Methane Monooxygenase Type Activation of O_2_ at an Iron(II)-Cyclam Center**”** *J. Am. Chem. Soc.*, **2020**, *142*, 5924–5928.

[6] G. A. Bain, J. F. Berry, "Diamagnetic Corrections and Pascal's Constants" *J. Chem. Educ.*, **2008**, *85*, 532–536.

[7] B. N. Figgis, "Magnetic Properties of Spin-Free Transition Series Complexes" *Nature*, **1958**, *182*, 1568–1570.

[8] G. M. Sheldrick, SADABS, Program for Empirical Absorption Correction of Area Detector Data, University of Göttingen, Göttingen (Germany) **1996**.

[9] G. M. Sheldrick, "SHELXT - Integrated Space-Group and Crystal-Structure Determination" *Acta Crystallogr. A*, **2015**, *71*, 3–8.

[10] G. M. Sheldrick, "Crystal Structure Refinement with SHELXL" *Acta Crystallogr. C Struct. Chem.*, **2015**, *71*, 3–8.

[11] C. B. Hübschle, G. M. Sheldrick, B. Dittrich, "ShelXle: A Qt Graphical User Interface for SHELXL" *J. Appl. Crystallogr.*, **2011**, *44*, 1281–1284.

[12] A. L. Spek, "PLATON SQUEEZE: A Tool for the Calculation of the Disordered Solvent Contribution to the Calculated Structure Factors" *Acta Crystallogr. C Struct. Chem.*, **2015**, *71*, 9–18.

[13] M. J. Frisch, G. W. Trucks, H. B. Schlegel, G. E. Scuseria, M. A. Robb, J. R. Cheeseman, G. Scalmani, V. Barone, B. Mennucci, G. A. Petersson, H. Nakatsuji, M. Caricato, X. Li, H. P. Hratchian, A. F. Izmaylov, Y. Honda, O. Kitao, H. Nakai, T. Vreven, J. A. Montgomery, Jr., J. E. Peralta, F. Ogliaro, M. Bearpark, J. J. Heyd, E. Brothers, K. N. Kudin, V. N. Staroverov, R. Kobayashi, J. Normand, K. Raghavachari, A. Rendell, J. C. Burant, S. S. Iyengar, J. Tomasi, M. Cossi, N. Rega, J. M. Millam, M. Klene, J. E. Knox, J. B. Cross, V. Bakken, C. Adamo, J. Jaramillo, R. Gomperts, R. E. Stratmann, O. Yazyev, A. J. Austin, R. Cammi, C. Pomelli, J. W. Ochterski, R. L. Martin, K. Morokuma, V. G. Zakrzewski, G. A. Voth, P. Salvador, J. J. Dannenberg, S. Dapprich, A. D. Daniels, Ö. Farkas, J. B. Foresman, J. V. Ortiz, J. Cioslowski, D. J. Fox, Gaussian 09, Gaussian Inc., Wallingford CT (USA) **2009**.

[14] A. D. Becke, "Density‐functional Thermochemistry. III. The Role of Exact Exchange" *J. Chem. Phys.*, **1993**, *98*, 5648–5652.

[15] C. Lee, W. Yang, R. G. Parr, "Development of the Colle-Salvetti Correlation-Energy Formula into a Functional of the Electron Density" *Phys. Rev. B*, **1988**, *37*, 785.

[16] P. J. Hay, W. R. Wadt, "Ab Initio Effective Core Potentials for Molecular Calculations. Potentials for the Transition Metal Atoms Sc to Hg" *J. Chem. Phys.*, **1985**, *82*, 270–283.

[17] R. Ditchfield, W. J. Hehre, J. A. Pople, "Self‐Consistent Molecular‐Orbital Methods. IX. An Extended Gaussian‐Type Basis for Molecular‐Orbital Studies of Organic Molecules" *J. Chem. Phys.*, **1971**, *54*, 724–728.

[18] M. M. Francl, W. J. Pietro, W. J. Hehre, J. S. Binkley, M. S. Gordon, D. J. DeFrees, J. A. Pople, "Self-Consistent Molecular Orbital Methods. XXIII. A Polarization-Type Basis Set for Second-Row Elements" *J. Chem. Phys.*, **1982**, *77*, 3654–3665.

[19] J. Tomasi, B. Mennucci, R. Cammi, "Quantum Mechanical Continuum Solvation Models" *Chem. Rev.*, **2005**, *105*, 2999–3093.

[20] F. Biegler‐König, J. Schönbohm, "Update of the AIM2000-Program for Atoms in Molecules" *J. Comput. Chem.* **2002**, *23*, 1489–1494.

[21] F. Neese, "The ORCA program system" *WIREs Comput. Mol. Sci.* **2012**, *2*, 73–78.

[22] M. Römelt, S. Ye, F. Neese, "Calibration of Modern Density Functional Theory Methods for the Prediction of ^57^Fe-Mössbauer Isomer Shifts: Meta-GGA and Double-Hybrid Functionals" *Inorg. Chem.* **2009**, *48*, 784–785.
